# Enantioselective propargylic [1,3]-rearrangements: copper-catalyzed *O*-to-*N* migrations toward C–N bond formation†

**DOI:** 10.1039/c7sc01042g

**Published:** 2017-03-31

**Authors:** Li-Jie Cheng, Alexander P. N. Brown, Christopher J. Cordier

**Affiliations:** a Department of Chemistry, Imperial College London South Kensington London SW7 2AZ UK ccordier@imperial.ac.uk

## Abstract

We have identified an enantioselective copper-catalyzed *O*-to-*N* formal [1,3]-rearrangement to form *N*-propargylic-2-pyridones. This enantioconvergent *O*-to-*N* propargylic rearrangement occurs rapidly at ambient temperature and high enantioselectivity is observed for a range of 3-alkyl-substituted substrates. Stereochemical features include a mild kinetic enantioenrichment of the substrate and a non-linear relationship between product and ligand enantiopurity. Based on kinetic analyses and cross-over experiments, we put forward a mechanistic proposal involving Cu-acetylides as well as bimetallic intermediates in which coordination to the pyridyl nitrogen is likely a crucial binding interaction.

## Abstract 2

We have identified an enantioselective copper-catalyzed *O*-to-*N* formal [1,3]-rearrangement to form *N*-propargylic-2-pyridones.

## Graphical abstract

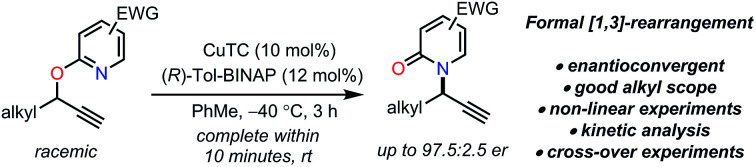

## Introduction

Thermal suprafacial *O*-to-*N* [3,3]-sigmatropic rearrangements^1^ are well documented for allylic acetimidates,^2^ phosphorimidates^3^ and cyanates.^4^ Transition metal catalyzed formal [3,3]-sigmatropic rearrangements of this type can be performed at ambient temperatures,^5^ and chiral metal–ligand complexes have allowed enantioselective processes to be developed.^6^ Thermal *O*-to-*N* [3,3]-rearrangements of 2-allyloxypyridines are also documented, providing *N*-allyl-2-pyridones,^7^ and Pd catalysts with chiral ligands have enabled enantioselective processes from achiral substrates (eqn (1)).^8^1
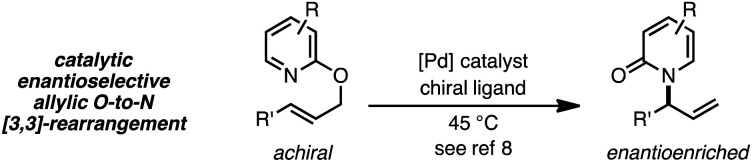
2
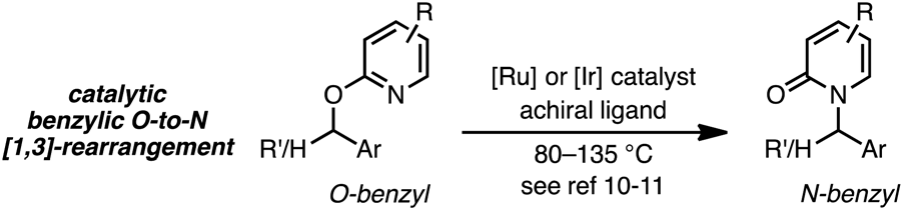
3
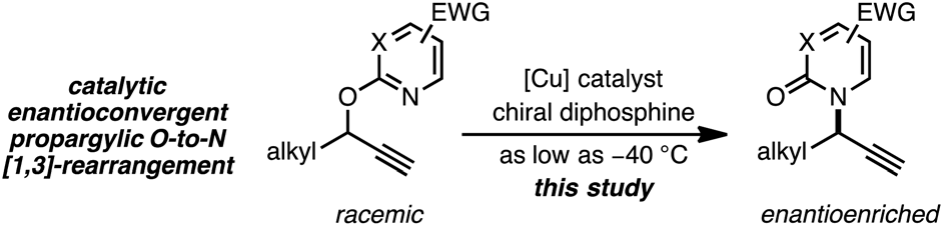


As a juxtaposition, suprafacial [1,3]-sigmatropic rearrangements are thermally disallowed^9^ but Ru^10^ and Ir^11^ catalysts can promote *formal* sigmatropic [1,3]-rearrangements of 2-benzyloxypyridine derivatives at elevated temperatures (eqn (2)).^12^ To our knowledge, enantioselective methods for *O*-to-*N* rearrangements yielding propargylic products have not been reported^13^ and, moreover, enantioselective *O*-to-*N* [1,3]-rearrangements are extremely rare.^14^ Here we report that a chiral Cu–diphosphine complex can promote the formal [1,3]-rearrangement of 2-propargyloxypyridines to enantioenriched *N*-propargylic-2-pyridones at temperatures as low as −40 °C (eqn (3)).4
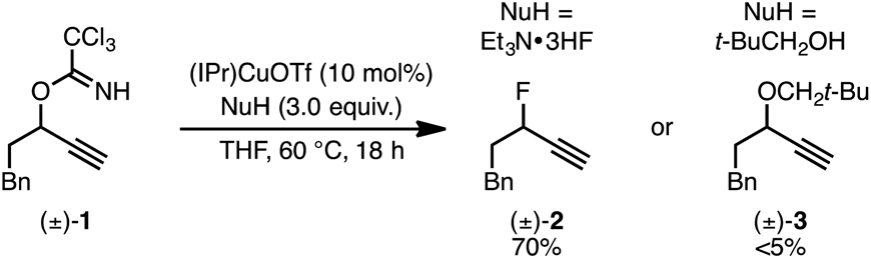
5
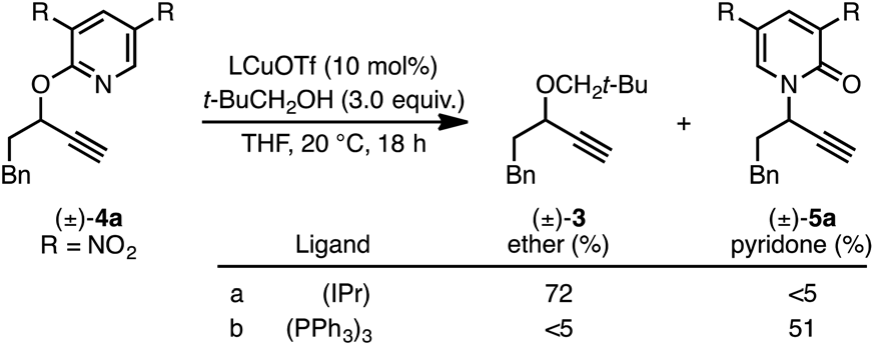


We recently reported a Cu-catalyzed fluorination protocol for the preparation of secondary and tertiary propargylic fluorides from the corresponding sulfonate esters and trichloroacetimidates.^15^ An *N*-heterocyclic carbene ligand was crucial to expanding the breadth of propargylic substitutions^16^ from *N*-,^17^*C*-,^18^ and *O*-nucleophiles^19^ to include fluoride. We decided to explore NHC–Cu complexes as catalysts for propargylic etherification of trichloroacetimidate (±)-1 (eqn (4)). Under the same conditions used for propargylic fluorination, direct replacement of Et_3_N·3HF with neopentyl alcohol resulted in trace (±)-3 (eqn (4)). A broader survey of imidate-like leaving groups led us to 2-propargyloxypyridine derivatives. We found that (IPr)CuOTf catalyzed etherification of (±)-4a at room temperature (eqn (5a)). A control experiment designed to contrast the influence of this NHC ligand with phosphine ligands led to a different result. Using catalytic (PPh_3_)_3_CuOTf, pyridone (±)-5a was formed in 51% yield, constituting a formal [1,3]-sigmatropic rearrangement (eqn (5b)). Furthermore, etherification of enantioenriched (*S*)-4a (98 : 2 er) led to racemic product under the same conditions. This observation is indicative of achiral intermediates^20^ and presented the possibility to develop an *enantioconvergent O*-to-*N* [1,3]-rearrangement by employing chiral phosphine ligands.

## Results and discussion

Investigation of a range of parameters^21^ showed that a complex derived from CuTC and (*R*)-L1 can catalyze the formation of (*R*)-5a at −40 °C in just 3 hours (Table 1, entry 2).

**Table 1:** Impact of reaction parameters on catalytic enantioconvergent formal [1,3]-rearrangement^a^

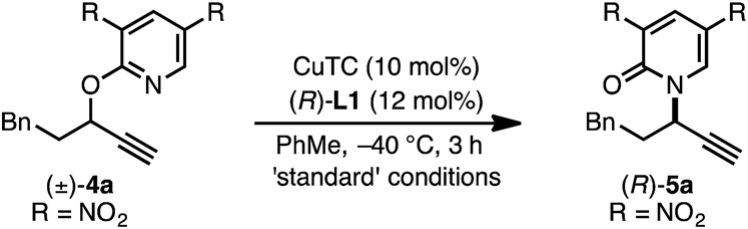				
Entry	Variation from ‘standard’ conditions	Conv.^b^ (%)	Yield^b^ (%)	er^c^
1	R = H	0	—	—
2	None	**>98**	**90**	**97.5 : 2.5**
3	No CuTC	0	—	—
4	No (*R*)-L1	0	—	—
5	CuOTf·0.5PhH, instead of CuTC	66	50	77 : 23
6	CuI, instead of CuTC	88	50	47 : 53
7	Cu(MeCN)_4_BF_4_, instead CuTC	72	60	46 : 54
8	(*R*)-L2, instead of (*R*)-L1	>98	94	96.5 : 3.5
9	(*R*)-L4, instead of (*R*)-L1	>98	97	95.5 : 4.5
10	(*R*)-L5, instead of (*R*)-L1	>98	95	96.5 : 3.5
11	(*R*)-L6, instead of (*R*)-L1	>98	92	96.5 : 3.5
12	(*R*)-L3, instead of (*R*)-L1	39	24	19 : 81
13	(*R*)-L7, instead of (*R*)-L1	80	40	50 : 50
14	(*R*)-L8, instead of (*R*)-L1	<2	—	—
15	THF, instead of PhMe	>98	80	96 : 4
16	Addition of *i*-Pr_2_NEt (2.0 equiv.)	>98	82	96.5 : 3.5
17	5 mol% CuTC, 6 mol% (*R*)-L1 (7 h)	>98	85	97.5 : 2.5
18	−20 °C (1 h), instead of −40 °C	>98	96	96 : 4
19	20 °C (10 min), instead of −40 °C	**>98**	**87**	**94 : 6**
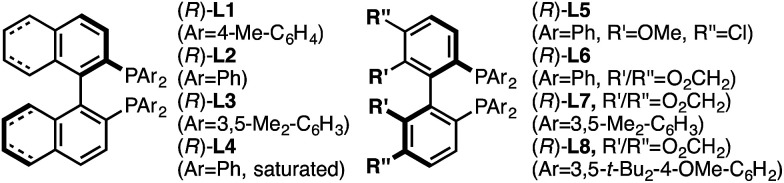				

a All data are the average of two experiments performed using 0.1 mmol substrate. For entries in which incomplete conversion was observed, reaction times were 24 h.

b Determined by analysis of the crude reaction mixtures by ^1^H NMR using CH_2_Br_2_ as an internal standard.

c Determined by chiral stationary phase HPLC. CuTC = copper(i)-thiophenecarboxylate. Bn = benzyl.

Under our standard protocol, nitro-substituents on the pyridine were found to be required (entry 1).^21*b*^ However, substrate (±)-4a underwent an efficient enantioconvergent *O*-to-*N* rearrangement, providing pyridone (*R*)-5a in 90% yield and 97.5 : 2.5 er (entry 2). In the absence of CuTC or (*R*)-L1, no conversion of (±)-4a was observed (entries 3 and 4). The copper source had a strong influence on both conversion and enantioselectivity (entries 5–7). Other ligand architectures faired similarly well in terms of yield and enantioselectivity compared with L1 (entries 8–11). Effects of the phosphine aryl-substituents were more pronounced; diphenyl- and di-4-methylphenyl-phosphines (L1–L2, L4–L6) resulted in high conversion and stereoinduction. However, under the same conditions bis-3,5-dimethylphenyl analogues led to low product enantiomeric ratios (entries 12 and 13) and the highly bulky ligand L8 resulted in no conversion (entry 14). Performing the transformation in THF (entry 15) offered similar results to toluene. Since related propargylic *substitutions* normally require base, we note that base is not required for this process (entry 16). Employing less catalyst did not impact enantioselectivity but did influence yield and reaction time (entry 17). If the transformation is performed at higher temperature, enantioselectivity is affected minimally (entry 18). Significantly, the rearrangement is complete in less than 10 minutes at ambient temperature (entry 19).

The *rearrangement* approach toward enantioselective propargylic C–N bond formation complements Cu-catalyzed *substitution* methods.^17^ In particular 3-alkyl substrates typically require longer reaction times during substitutions with *N*-nucleophiles than 3-aryl substrates require when using Cu–diphosphine or Cu–pybox catalyst systems; the current method occurs far faster and occurs at remarkably lower temperatures.^22^ To examine the efficacy of our conditions toward propargylic *substitutions*, we decided to use 2-(1*H*)-pyridones 6a and 6b in reaction with mesylate (±)-7 (eqn (6) and (7)).^23^6
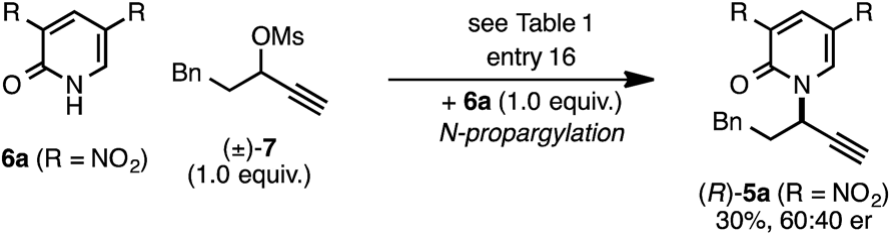
7
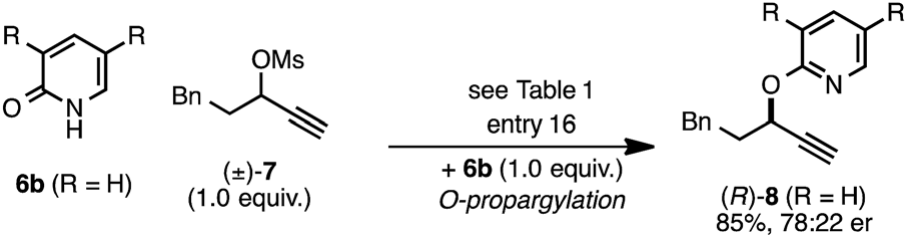


*N*-propargylated product (*R*)-5a was formed but with substantially reduced enantioselectivity (eqn (6)).^24^ Reaction of unsubstituted pyridone 6a led exclusively to *O*-propargylation, forming (*R*)-8 in good yield and with modest enantioselectivity (eqn (7)).^25^ The observed *O*- *vs. N*-alkylation selectivity may arise from initial *O*-alkylation of 7 with 6a or 6b but with only intermediate 4a undergoing the rearrangement process. The sense of stereoinduction during these substitutions matches that for the rearrangement process, indicating a similar pathway for enantiocontrol but with markedly reduced efficacy under these conditions.

**Scheme 1:**
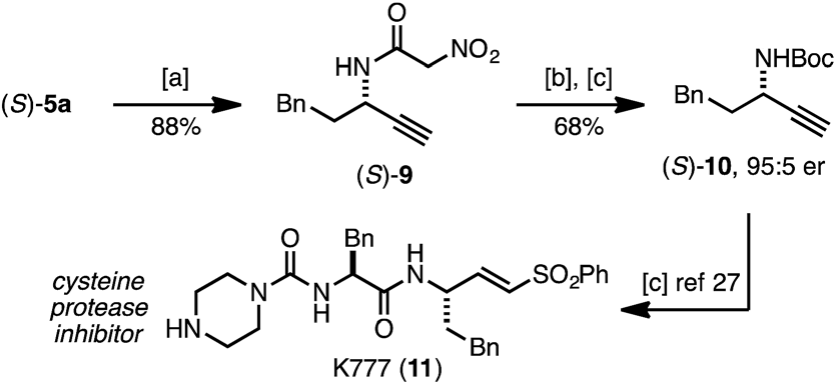
Synthetic utility of pyridone (*S*)-5a. (a) NH_3_ in MeOH (7 M), cyclohexane (2.0 equiv.), 65 °C, 3 h; (b) aq. HCL, 90 °C, 24 h; (c) Boc_2_O (1.5 equiv.), NaHCO_3_ (2.0 equiv.), DCM, RT, 18 h.

Following established protocols for modifying the pyridone core^26^ we demonstrated that (*S*)-5a serves as a surrogate for α-nitroacetanilide (*S*)-9, and can be readily converted into amine derivative (*S*)-10 (Scheme 1). While related substitution methods are not suitable for the preparation of propargylic amides or primary amines, the rearrangement approach to (*S*)-5a represents a precursor to (*S*)-10, an intermediate in synthesis of the potent cysteine protease inhibitor K777 (11).^27^

Having established a useful protocol for an enantioselective C–N bond formation, we elected to move forward to explore the influence of 3-substituents on enantioselectivity in this transformation (Table 2). Preparative scale reaction of substrate (±)-4a proceeded smoothly (entry 1) and a reaction performed on 5 mmol scale provided gram quantities of (*R*)-5a (81%). Rearrangements were successful using substrates containing an unfunctionalized alkyl chain, an alkene, and a primary alkyl chloride (entries 2–4). A benzyl ether and silyl ether were both tolerated (entries 5 and 6) but an unprotected alcohol negatively affected both conversion and stereoinduction (entry 7). The presence of an acetal, an unprotected aldehyde and a methyl ester had minimal effects on enantioselectivity (entries 8–10). Assessing the influence of bulkier 3-substituents, a 3-benzyl group was tolerated very poorly while a 3-cyclohexyl appendage was accommodated smoothly (entries 11 and 12). Reaction of a highly sterically hindered 3-(1-adamantyl) substrate suffered from low conversion and poor enantioselectivity (entry 13). A benzylic substrate was compatible with our standard conditions but led to product with mediocre er (entry 14). Furthermore, attempts to conduct this rearrangement using a substrate bearing an *internal* alkyne failed, and recovered starting material was observed even after prolonged heating.

**Table 2:** Scope of the catalytic enantioconvergent formal [1,3]-rearrangement with respect to propargylic substituents^a^

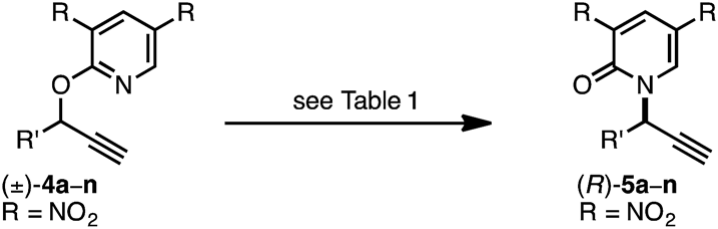			
Entry	R′	Yield^b^ (%)	er^c^
1 (4a)	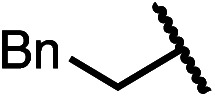	90	97.5 : 2.5
		81^d^	97.5 : 2.5^d^
2 (4b)	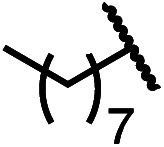	76	97 : 3
3 (4c)	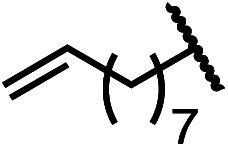	88	95.5 : 4.5
4 (4d)	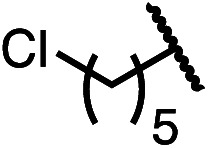	92	97 : 3
5 (4e)	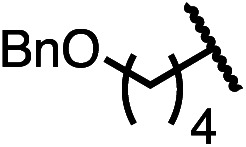	90	96.5 : 3.5
6 (4f)	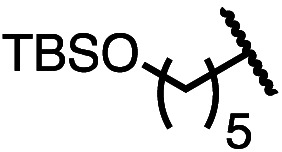	75	96.5 : 3.5
7 (4g)	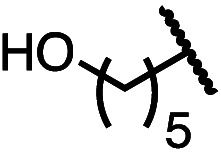	52	63 : 37
8 (4h)	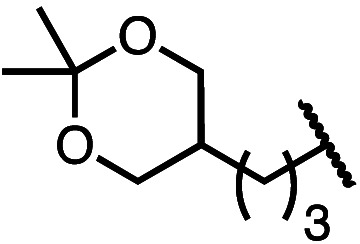	88	97 : 3
9 (4i)	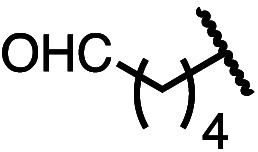	89	96.5 : 3.5
10 (4j)	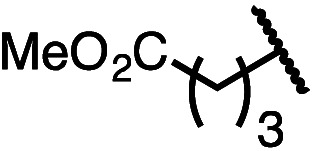	90	96 : 4
11 (4k)	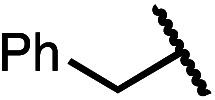	49	62 : 38
12 (4l)	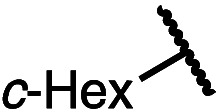	80	95.5 : 4.5
13 (4m)	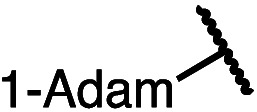	30	55 : 45
14^e^ (4n)	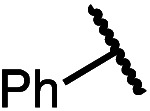	70	85 : 15

a All data are the average of two experiments performed using 0.4 mmol substrate, unless otherwise stated.

b Isolated yields.

c Determined by chiral stationary phase HPLC.

d Performed using 5.0 mmol of substrate.

e The Cahn–Ingold–Prelog terminology for product 5n denotes the (*S*)-configuration. Bn = benzyl; TBS = *tert*-butyldimethylsilyl; 1-Adam = 1-adamantyl.

**Fig. 1:**
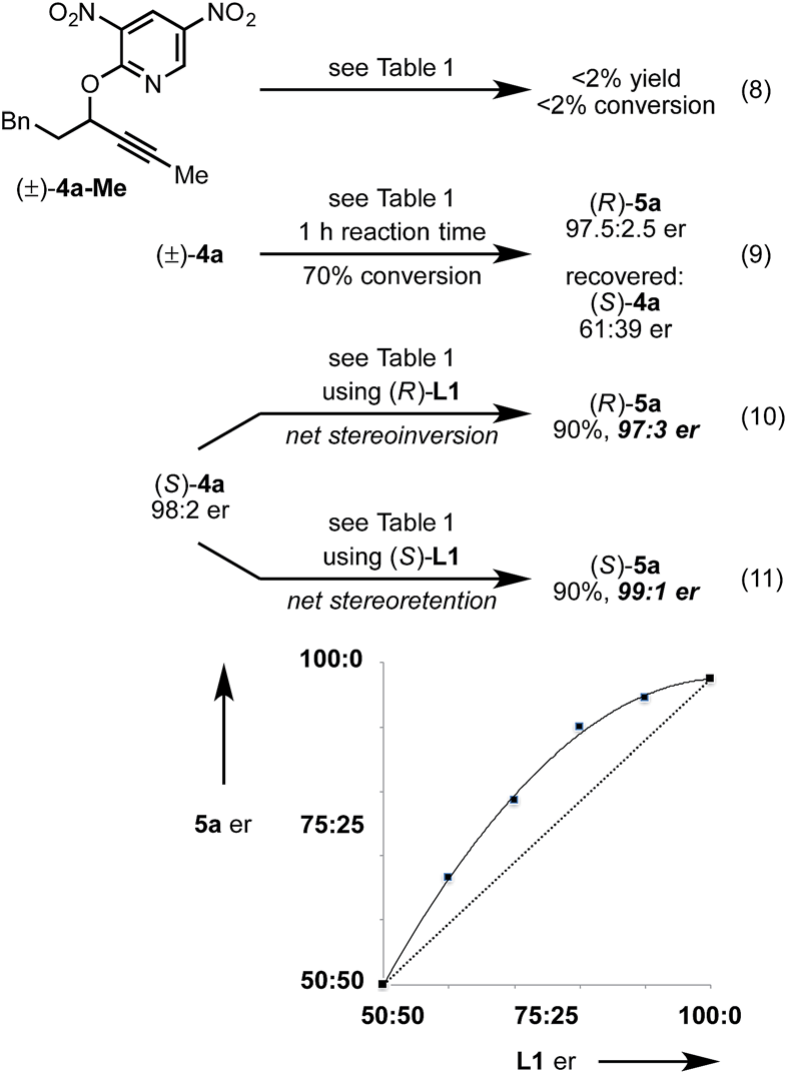
Non-linear er relationship between L1 and 5a under standard conditions using (±)-4a.

Next, we conducted a range of experiments designed to reveal stereochemical features about this transformation. Quenching our standard reaction of (±)-4a after 1 h (70% conversion) led to recovered starting material (*S*)-4a with 61 : 39 er, indicating a mild kinetic resolution^28^ (*s* factor = 2.7) associated with C–O bond cleavage (eqn (8)).^29^ We note that the enantiomeric ratio of (*R*)-5a is constant throughout the course of the reaction. To examine the extent of enantioconvergence during our rearrangement, we subjected enantioenriched substrate (*S*)-4a to our standard rearrangement conditions using (*R*)-L1 (eqn (9)) and (*S*)-L1 (eqn (10)). The stereochemistry of the pyridone product was dependent primarily on the stereochemistry of the ligand rather than of the starting propargyloxypyridine. However, when employing (*S*)-L1 net stereoretention was observed to occur with a small but measurable enhancement in product er (99 : 1 *cf.* 97 : 3), indicating a non-zero degree of stereochemical transfer during this transformation.^28^ A positive non-linear effect was observed when employing non-enantiomerically pure L1 (Fig. 1); this observation may indicate dinuclear, or higher order, species involved in the stereochemistry determining step^30^ or may be a kinetic consequence off-cycle polynuclear species.^31^

**Fig. 2:**
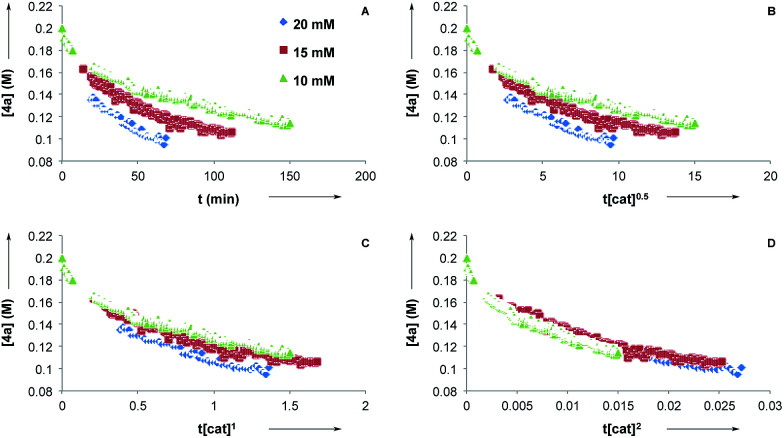
(a) Reaction profiles for initial conditions: [CuTC–Tol-BINAP] = 20, 15, 10 mM. Determinations of catalyst order using time-normalization profiles: (b) half order in catalyst; (c) first order in catalyst; (d) second order in catalyst.

Next, we conducted reaction progress kinetic analysis to elucidate some kinetic features of this transformation,^32^ using the rearrangement of 4a as a representative substrate. Concentration profiles of 4a were generated by following the rearrangement using ^1^H NMR in *d*_8_-PhMe at [CuTC–Tol-BINAP] = 10, 15, and 20 mM (Fig. 2A). Applying the Burés method^33^ for variable time normalization analysis, using *t*[cat]_T_^*n*^, shows that the transformation is far from half- or first-order (Fig. 2B and C, respectively) but more closely approximate to second order (Fig. 2D)^34^ in catalyst for [CuTC–Tol-BINAP] between the 10–20 mM range examined. These data are consistent with the results obtained following nonlinear experiments and imply that bimetallic species are involved in the turnover-limiting and stereochemistry-determining steps.
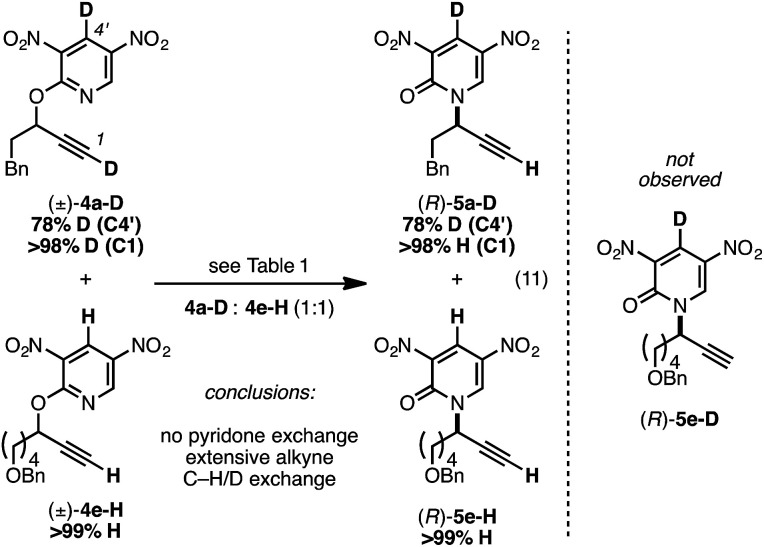


We used ^31^P NMR to identify catalyst species present in the reaction mixture: non-complexed ligand, Tol-BINAP, showed a signal at −16.47 ppm; the CuTC–Tol-BINAP adduct showed a signal at −3.93 ppm, and addition of the product led to a signal at −4.30 ppm. At *ca.* 20% conversion, ^31^P NMR of the reaction mixture showed signals corresponding to the non-complexed ligand, the CuTC–Tol-BINAP adduct, and the presence of two additional signals at −3.56 and −0.94 ppm. We assign these signals to potential bimetallic species of the catalyst resting state.

We prepared the deuterium-labeled substrate 4a-D in order to examine the extent of pyridone–alkyne dissociation during the rearrangement process (eqn (11)). Treatment of a 1 : 1 mixture of 4a-D and 4e-H to our standard conditions led to rearranged products 5a-D and 5e-H; the ‘cross-over’ adduct 5e-D was not observed. These results are consistent with a mechanism that involves no dissociation between the alkyne component of 4a-D and the deuterium-labeled pyridone moiety. Alternatively solvent-cage effects may kinetically favor C–N bond formation over diffusion-based separation of the pyridone and alkyne species. During our synthetic preparation of 4a-D, the terminal alkyne was also labeled with a deuteron. Performing the rearrangement of 4a-D, either in the presence or absence 4e-H, led to 5a-D bearing a terminal proton; performing this rearrangement in *d*_8_-toluene and monitoring progress by ^1^H NMR demonstrated that terminal deuteron/proton exchange was rapid and the source of the proton in eqn (11) was likely adventitious moisture.^35^

**Scheme 2:**
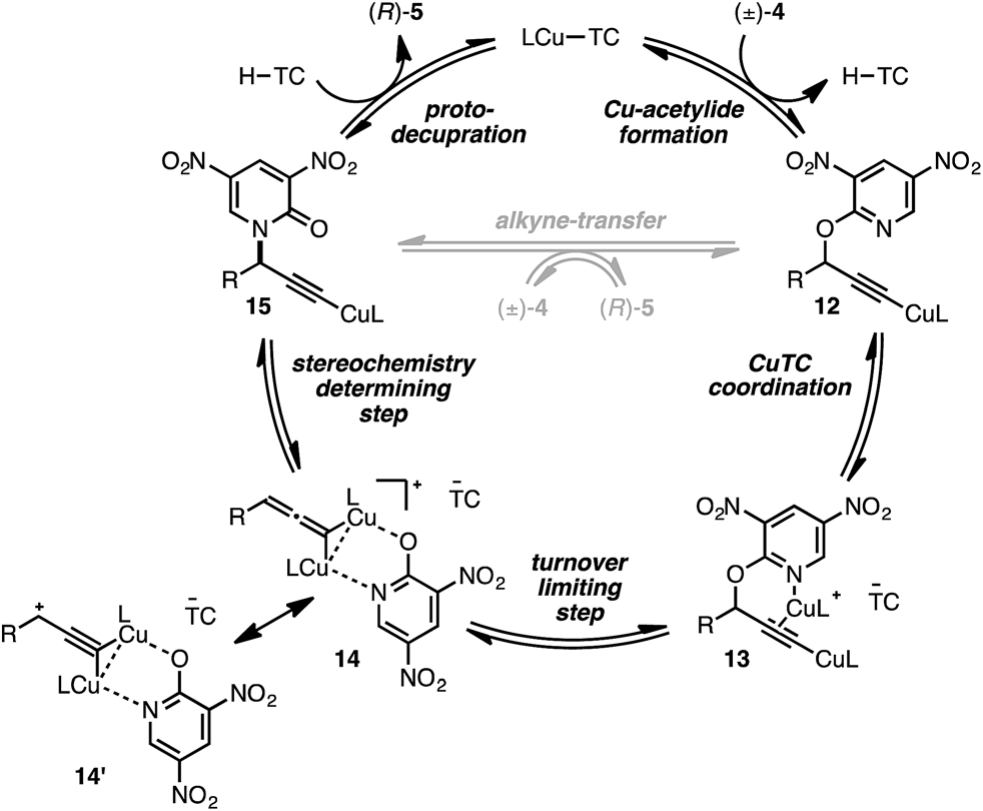
Proposed mechanistic pathway.

We conclude from the data presented that bimetallic intermediates are likely involved and speculate that Cu-coordination to the pyridyl nitrogen of (±)-4a may be an important binding interaction during C–O bond cleavage (Scheme 2). Thus, complexation of a second catalyst to Cu-acetylide 12 leads to bimetallic intermediate 13 (the putative resting state). We assign the heterolytic C–O bond cleavage as the turnover-limiting state, leading to bimetallic copper-pyridone intermediate 14/14′, similar to that proposed by Nishibayashi during propargylic etherifications;^36^ collapse of this intermediate permits C–N bond formation leading to Cu-acetylide 15. The cycle may close by means of protodecupration or, alternatively, alkyne exchange with another substrate molecule.

## Conclusions

In summary, we have developed the first enantioconvergent *O*-to-*N* [1,3]-rearrangement of a propargylic substrate, specifically, the enantioselective Cu-catalyzed rearrangement of electron-deficient 2-propargyloxy-pyridines. High enantioselectivity is observed with a range of 3-alkyl substituents, and short reaction times were noted in all cases. The pathway for stereoconvergence in the present method likely involves heterolytic C–O bond cleavage promoted by bimetallic Cu-species. Additional investigations to elucidate the mechanism of this transformation are underway, and methods that expand upon this [1,3]-rearrangement concept to include other propargylic bond formations will be reported in due course.

## Supplementary Material

SC-008-C7SC01042G-s001

SC-008-C7SC01042G-s002
