# Supplementary material for: Enantioselective propargylic [1,3]-rearrangements: copper-catalyzed O-to-N migrations toward C–N bond formation
Source: Chem Sci. 2017 Mar 31;8(6):4299–305. doi: 10.1039/c7sc01042g (PMC5635726; doi:10.1039/c7sc01042g)
Supplement: SC-008-C7SC01042G-s002 [file SC-008-C7SC01042G-s002.pdf]

Electronic Supplementary Material (ESI) for Chemical Science.
This journal is © The Royal Society of Chemistry 2017

Enantioselective Propargylic [1,3]-Rearrangements: Copper-Catalyzed *O*-to-*N* Migrations Toward C–N Bond Formation

Li-Jie Cheng, Alexander P. N. Brown, Christopher J. Cordier*

Department of Chemistry, Imperial College London,
South Kensington, London SW7 2AZ, UK
*c.cordier@imperial.ac.uk

Supporting Information

Table of Contents

1.	General Information	S–2
2.	General Procedure for Optimization of Rearrangement	S–3
3.	Experimental Procedures for Substrate Syntheses	S–5
4.	General Procedure for Rearrangement	S–13
5.	Mechanistic studies	S–21
6.	Derivations of the Product **(*S*)-5a**	S–25
7.	X-Ray Crystallographic Data	S–28
8.	^{1}H, ^{13}C NMR Spectra of Unknown Compounds	S–31
9.	HPLC Spectra of Enantioenriched Products	S–67

1. General Information

General Procedures. Reactions requiring anhydrous conditions were conducted in oven-dried glassware under dry N_2. Screens were performed in 4 mL glass vials with a PTFE-lined cap, and all other reactions were performed in round-bottom flasks with rubber septa. Syringes were used to transfer air- and moisture-sensitive reagents. Thin layer chromatography (TLC) was conducted with E. Merck silica gel 60 F254 pre-coated plates (0.25 mm) and visualised initially by UV light (254 nm) or potassium permanganate staining. Purification of compounds was achieved by column chromatography using Merck Flash Silica Gel 60 (230-400 mesh). Organic solutions were concentrated under reduced pressure using a rotary evaporator.

Materials. Unless otherwise noted, materials obtained from commercial suppliers were used without further purification. THF was distilled from Na/benzophenone under an atmosphere of dry N_2. Et_2O, CH_2Cl_2, CH_3CN, hexane, toluene and were dried on a LC Technology Solutions Inc. SP-1 Solvent Purification System under N_2. CuTC, CuOTf, *rac*-BINAP, (*R*)-Tol-BINAP, (*S*)-Tol-BINAP, (*R*)-SEGPHOS, (*R*)-Cl-MeO-BIPHEP, (R)-*i*-Pr-Pybox were purchased from Sigma-Aldrich Company. Propargylic alcohols were synthesized according to literature.[1]

Instrumentation. Nuclear Magnetic Resonance (NMR) spectra were recorded on BRUKER AV (400 MHz) at 298 K. Chemical shifts (δ) are reported in ppm with the residual solvent signal as internal standard (chloroform at 7.26 and 77.00 ppm for ^1H and ^{13}C NMR spectroscopy, respectively). Abbreviations for signal coupling are as follows: s, singlet; d, doublet; t, triplet; q, quartet; quin., quintet; m, multiplet; br., broad. Coupling constants were taken from the spectra directly and are uncorrected and not averaged according to protons determined to exist in the same spin system according to COSY experiments. Signals with the appearance of a given multiplicity are preceded with the term 'app.' to denote an apparent splitting pattern when chemically distinct nuclei display very similar coupling constants. *^1H and ^{13}C NMR provided are taken directly using material for which the yield is quoted, without further purification, and are representative of purity*. Infrared (IR) spectra were recorded on a PERKIN ELMER Spectrum Two ATR-FT-IR as neat compounds. Absorptions are given in wavenumbers (cm^{-1}). Mass spectra (MS) were obtained on Agilent 5975C spectrometer using electron impact (EI) or Agilent 6220 (for high resolution MS) using electrospray ionization time-of-flight (ESI-TOF). Optical rotation was measured by the Perkin Elmer 341 polarimeter. HPLC analyses were carried out on an Agilent 1260 Infinity Series system, employing Daicel Chiracel columns.

1 Cheng, L.-J.; Cordier, C. J. *Angew. Chem. Int. Ed.* **2015**, *54*, 13734–13738.

2. Selected Optimization Experiments

General Procedure for Optimization of Rearrangement:

CuX_n (1.9 mg, 0.01 mmol) and chiral ligand (0.012 mmol) were added in turn to a 4 mL vial with a 1 cm stir bar. The vial was capped with a PTFE-lined cap and then it was evacuated and refilled with N_2 three times. Then solvent (0.5 mL) was added via 1 mL syringe. The mixture was stirred at room temperature for 1 h. The vial was then cooled down to –40 °C using a cooling bath and a solution of propargyloxypyridine (0.1 mmol) in solvent (0.5 mL) was added dropwise. After the addition is complete, the reaction mixture was stirred at –40 °C for 18 h. The reaction mixture was filtered through a pad of silica gel (a pipette with about 5 cm silica gel) and washed with ethyl acetate (10 mL). The filtrate was concentrated under reduced pressure. The residue was dissolved in $CDCl_3$ and CH_2Br_2 (0.1 mmol) was added as internal standard for ^{1}H NMR analysis. The er was determined by Chiral HPLC.

Table S1. Investigation of Ligands

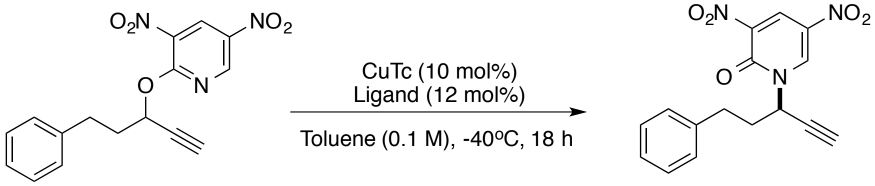

entry	ligand	yield (%)	er (%)
1	(*R*)-Tol-BINAP	90	**97.5:2.5**
2	(*R*)-BINAP	94	96.5:3.5
3	(*R*)-DM-BINAP	24 (71% SM)	19:81
4	(*R*)-SEGPHOS	92	96.5:3.5
5	(*R*)-DM-SEGPHOS	40 (20% SM)	50:50
6	(*R*)-DTBM-SEGPHOS	N.R.	--
7	(*R*)-Cl-MeO-BIPHEP	95	96.5:3.5
8	(*S,S*)-^{i}Pr-Pybox	50 (26% SM)	76:24
9	(*R*)-H8-BINAP	97	95.5:4.5

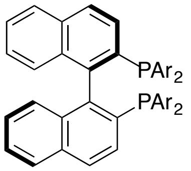

(*R*)-BINAP, Ar = Ph
(*R*)-Tol-BINAP, Ar = 4-MeC_6H_4
(*R*)-DM-BINAP, Ar = 3,5-$Me_2C_6H_3$

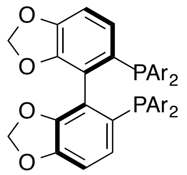

(*R*)-SEGPHOS, Ar = Ph
(*R*)-DM-SEGPHOS, Ar = 3,5-$Me_2C_6H_3$
(*R*)-DTBM-SEGPHOS, Ar = 3,5-^{t}Bu2-4-$MeOC_6H_2$

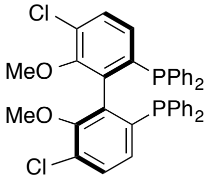

(*R*)-Cl-MeO-BIPHEP

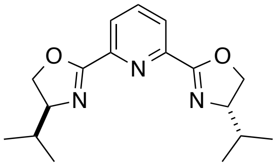

(*S,S*)-^{i}Pr-Pybox

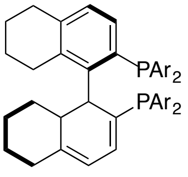

(*R*)-H8-BINAP, Ar = Ph

Table S2. Evaluation of Other Functional Group[2]

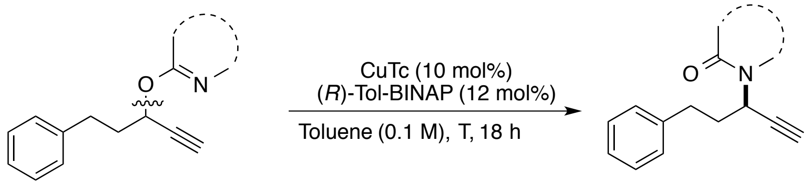

Entry	Functional group	Temp. (°C)	Conv. (%)	Yield (%)
1	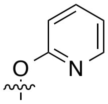	50	0	0
2	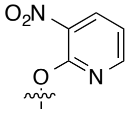	50	100	36[a]
3	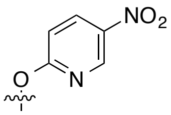	100	100	19[b]
4	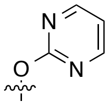	70	100	33[b]
5	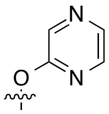	70	57	0
6	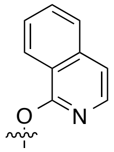	70	70	0
7	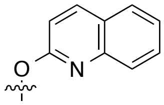	50	100	0
8	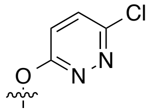	100	36	0

[a] 53:47 er was obtained. [b] <52:48 er was obtained.

[2] For all the substrates, no reaction was observed under room temperature.

3. Experimental Procedures for Substrate Syntheses

General Procedure for Synthesis of Propargyloxylpyridines

NaH (2.0 eq of a 60% dispersion in mineral oil) was added into the oven-dried flask with a stir bar. The flask was evacuated and refilled with N_2 three times. THF was transferred to the flask via syringe and the reaction was cooled to 0 °C. In another flask, propargylic alcohol (1.2 eq) was dissolved in THF (1.0 M) and added dropwise to the above suspension at 0 °C. After 10 min, the cooling bath was removed to reach room temperature and the mixture was stirred for a further 20 min. Then a solution of 2-chloro-3,5-dinitropyridine (1.0 eq) in THF (1.0 M) was added dropwise to the above solution at 0 °C. After addition is complete, the brown solution was allowed to room temperature and continue to stir for 2 h. Then the mixture was poured into ice/water in a beaker. The mixture was extracted with EtOAc three times. The combined organic layers were washed with a saturated aqueous sodium chloride solution, dried over magnesium sulphate, filtered and concentrated under reduced pressure. The residue was purified by silica gel chromatography (Hexane/ethyl acetate as eluent) to give the corresponding propargyloxypyridine.

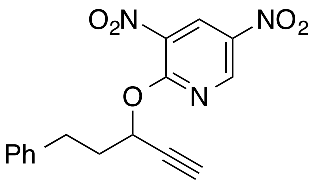

3,5-Dinitro-2-(1-phenethyl-2-propynyloxy)pyridine (4a). The title compound was prepared according to the general procedure using NaH (60% dispersion in mineral oil, 1.6 g, 40.0 mmol), 5-phenyl-1-pentyn-3-ol (3.84 g, 24.0 mmol), 2-chloro-3,5-dinitropyridine (4.04 g, 20.0 mmol). The crude material was purified by silica gel chromatography (hexanes/ethyl acetate = 10:1) to afford the title compound as a light yellow solid (4.8 g, 73% yield).

Mp: 85–86 °C.

^{1}H NMR (400 MHz, $CDCl_3$) δ 9.29 (d, J = 2.6 Hz, 1H), 9.09 (d, J = 2.6 Hz, 1H), 7.38 – 7.16 (m, 5H), 5.87 (app. td, J = 6.5, 2.2 Hz, 1H), 2.97 (t, J = 7.7 Hz, 2H), 2.61 (d, J = 2.1 Hz, 1H), 2.52 – 2.33 (m, 2H).

^{13}C NMR (101 MHz, $CDCl_3$) δ 157.7, 147.4, 140.1, 138.5, 132.7, 130.9, 128.6, 128.4, 126.3, 79.8, 75.5, 68.5, 36.2, 30.9.

IR (neat) 3285, 2923, 2120, 1708, 1602, 1331, 1230, 705, 696 cm^{-1}.

HRMS (CI) Calcd. for $C_{16}H_{14}N_3O_5$ ($[M+H]^+$): 328.0928; Found: 328.0929.

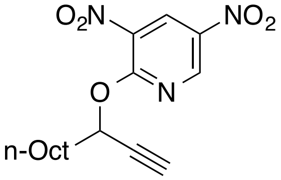

3,5-Dinitro-2-(1-octyl-2-propynyloxy)pyridine (4b). The title compound was prepared according to the

general procedure using NaH (60% dispersion in mineral oil, 320 mg, 8.0 mmol), 1-undecyn-3-ol (801 mg, 4.8 mmol), 2-chloro-3, 5-dinitropyridine (816 mg, 4.0 mmol). The crude material was purified by silica gel chromatography (hexanes/ethyl acetate = 10:1) to afford the title compound as a light yellow solid (1.4 g, >99% yield).

^{1}H NMR (400 MHz, $CDCl_3$) δ 9.29 (d, *J* = 2.6 Hz, 1H), 9.06 (d, *J* = 2.5 Hz, 1H), 5.88 (app. td, *J* = 6.6, 2.1 Hz, 1H), 2.52 (d, *J* = 2.1 Hz, 1H), 2.10 – 1.95 (m, 2H), 1.64 – 1.49 (m, 2H), 1.41 – 1.18 (m, 10H), 0.93 – 0.82 (m, 3H).

^{13}C NMR (101 MHz, $CDCl_3$) δ 157.9, 147.4, 138.4, 132.8, 130.8, 80.1, 75.1, 69.4, 34.6, 31.8, 29.3, 29.1, 29.0, 24.8, 22.6, 14.1.

IR (neat) 3284, 2924, 2855, 2122, 1600, 1591, 1540, 1347, 1330, 963, 716 cm^{-1}.

HRMS (CI) Calcd. for $C_{16}H_{22}N_3O_5$ ($[M+H]^+$): 336.1554; Found: 336.1554.

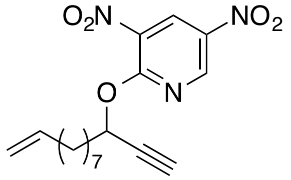

2-(1-Ethynyl-9-decenyloxy)-3,5-dinitropyridine (4c). The title compound was prepared according to the general procedure using NaH (60% dispersion in mineral oil, 240 mg, 6.0 mmol), propargyic alcohol (648 mg, 3.6 mmol), 2-chloro-3,5-dinitropyridine (612 mg, 3.0 mmol). The crude material was purified by silica gel chromatography (hexanes/ethyl acetate = 10:1) to afford the title compound as a light yellow oil (660 mg, 62% yield).

^{1}H NMR (400 MHz, $CDCl_3$) δ 9.29 (d, *J* = 2.6 Hz, 1H), 9.06 (d, *J* = 2.6 Hz, 1H), 5.87 (app. td, *J* = 6.6, 2.2 Hz, 1H), 5.80 (ddt, *J* = 17.0, 10.1, 6.7 Hz, 1H), 5.05 – 4.86 (m, 2H), 2.52 (d, *J* = 2.2 Hz, 1H), 2.11– 2.02 (m, 4H), 1.64 – 1.52 (m, 2H), 1.44 – 1.25 (m, 8H).

^{13}C NMR (101 MHz, $CDCl_3$) δ 157.9, 147.4, 139.1, 138.4, 132.7, 130.8, 114.2, 80.1, 75.1, 69.3, 34.5, 33.7, 29.2, 28.9, 28.8, 24.7.

IR (neat) 3284, 2925, 2120, 1720, 1598, 1591, 1348, 1329, 1301, 965, 709, 653 cm^{-1}.

HRMS (CI) Calcd. for $C_{17}H_{22}N_3O_5$ ($[M+H]^+$): 348.1554; Found: 348.1555.

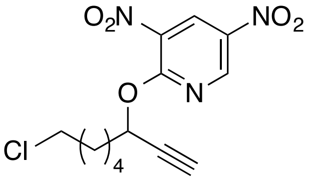

2-[1-(5-Chloropentyl)-2-propynyloxy]-3,5-dinitropyridine (4d). The title compound was prepared according to the general procedure using NaH (60% dispersion in mineral oil, 320 mg, 8.0 mmol), propargyic alcohol (768 mg, 4.8 mmol), 2-chloro-3,5-dinitropyridine (816 mg, 4.0 mmol). The crude material was purified by

silica gel chromatography (hexanes/ethyl acetate = 10:1) to afford the title compound as a light yellow solid (1.2 g, 92% yield).

^{1}H NMR (400 MHz, $CDCl_3$) δ 9.29 (d, J = 2.6 Hz, 1H), 9.06 (d, J = 2.6 Hz, 1H), 5.89 (app. td, J = 6.4, 2.1 Hz, 1H), 3.55 (t, J = 6.6 Hz, 2H), 2.53 (d, J = 2.1 Hz, 1H), 2.10 – 2.00 (m, 2H), 1.87 – 1.76 (m, 2H), 1.68 – 1.49 (m, 4H).

^{13}C NMR (101 MHz, $CDCl_3$) δ 157.8, 147.4, 138.4, 132.7, 130.8, 79.9, 75.3, 69.0, 44.8, 34.3, 32.2, 26.2, 24.0.

IR (neat) 3290, 2943, 2123, 1602, 1590, 1335, 1075, 957, 830, 715 cm^{-1}.

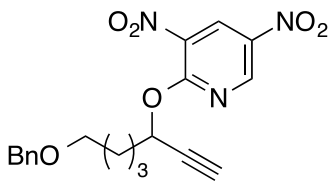

2-{1-[4-(Benzyloxy)butyl]-2-propynyloxy}-3,5-dinitropyridine (4e). The title compound was prepared according to the general procedure using NaH (60% dispersion in mineral oil, 320 mg, 8.0 mmol), propargyic alcohol (1.05 g, 4.8 mmol), 2-chloro-3, 5-dinitropyridine (816 mg, 4.0 mmol). The crude material was purified by silica gel chromatography (hexanes/ethyl acetate = 10:1) to afford the title compound as a light yellow solid (700 mg, 45% yield).

Mp: 63–64 °C.

^{1}H NMR (400 MHz, $CDCl_3$) δ 9.30 (d, J = 2.6 Hz, 1H), 9.07 (d, J = 2.6 Hz, 1H), 7.43 – 7.20 (m, 5H), 5.93 (app. td, J = 6.5, 2.1 Hz, 1H), 4.53 (s, 2H), 3.61 – 3.50 (m, 2H), 2.56 (d, J = 2.1 Hz, 1H), 2.12-2.07 (m, 2H), 1.75-1.73 (m, 4H).

^{13}C NMR (101 MHz, $CDCl_3$) δ 157.8, 147.3, 138.4 (2C), 132.7, 130.8, 128.3, 127.6, 127.5, 80.0, 75.2, 72.9, 69.9, 69.2, 34.3, 29.1, 21.7.

IR (neat) 3285, 3081, 2861, 2123, 1603, 1590, 1344, 1077, 705 cm^{-1}.

HRMS (CI) Calcd. for $C_{19}H_{20}N_3O_6$ ($[M+H]^+$): 386.1347; Found: 386.1346.

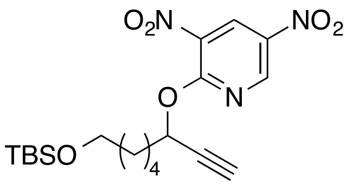

2-[1-(5-tert-Butyldimethylsilyloxypentyl)-2-propynyloxy]-3,5-dinitropyridine (4f). The title compound was prepared according to the general procedure using NaH (60% dispersion in mineral oil, 800 mg, 20 mmol), propargyic alcohol (3.07 g, 12 mmol), 2-chloro-3, 5-dinitropyridine (2.04 g, 10 mmol). The crude material was purified by silica gel chromatography (hexanes/ethyl acetate = 10:1) to afford the title compound as a yellow oil (3.6 g, 85% yield).

^{1}H NMR (400 MHz, $CDCl_3$) δ 9.29 (d, J = 2.6 Hz, 1H), 9.06 (d, J = 2.6 Hz, 1H), 5.88 (app. td, J = 6.5, 2.1 Hz, 1H), 3.63-3.58 (m, 2H), 2.52 (d, J = 2.1 Hz, 1H), 2.08-2.01 (m, 2H), 1.67 – 1.40 (m, 6H), 0.88 (s, 9H), 0.04 (s, 6H).

^{13}C NMR (101 MHz, $CDCl_3$) δ 157.9, 147.4, 138.4, 132.7, 130.8, 80.1, 75.1, 69.3, 62.9, 34.6, 32.5, 25.9, 25.3, 24.6, 18.3, -5.3.

IR (neat) 3287, 2929, 2122, 1603, 1590, 1343, 1076, 830, 775 cm^{-1}.

HRMS (CI) Calcd. for $C_{19}H_{30}N_3O_6Si$ ($[M]^+$): 424.1898; Found: 424.1897.

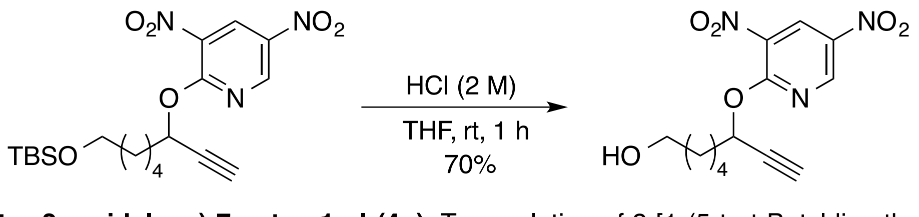

6-(3,5-Dinitro-2-pyridyloxy)-7-octyn-1-ol (4g). To a solution of 2-[1-(5-tert-Butyldimethylsilyloxypentyl)-2-propynyloxy]-3,5-dinitropyridine (2.54 g, 6.0 mmol) in THF (18 mL) was added aqueous HCl (2 M, 9 mL) dropwise via pipette at 0 °C. The reaction mixture was then stirred at room temperature for 4 h. H_2O (20 mL) and EtOAc (60 mL) were added to dilute the mixture. After the organic phase was separated, the aqueous phase was extracted twice with EtOAc (60 mL). The combined organic phases were washed with brine, dried over $MgSO_4$, filtered and then concentrated under reduced pressure. The crude oil was purified by silica gel chromatography (hexane/EtOAc = 2:1) to afford the product as a colorless oil (600 mg, 70% yield).

^{1}H NMR (400 MHz, $CDCl_3$) δ 9.29 (d, J = 2.6 Hz, 1H), 9.06 (d, J = 2.6 Hz, 1H), 5.88 (app. td, J = 6.4, 2.1 Hz, 1H), 3.65 (t, J = 6.4 Hz, 2H), 2.52 (d, J = 2.1 Hz, 1H), 2.12 – 1.97 (m, 2H), 1.66 – 1.53 (m, 5H), 1.52 – 1.41 (m, 2H).

^{13}C NMR (101 MHz, $CDCl_3$) δ 157.8, 147.4, 138.4, 132.7, 130.8, 80.0, 75.2, 69.2, 62.6, 34.4, 32.4, 25.2, 24.5.

IR (neat) 3504, 3290, 2936, 2121, 1602, 1590, 1335, 1305, 955, 830 cm^{-1}.

HRMS (CI) Calcd. for $C_{13}H_{16}N_3O_6$ ($[M]^+$): 310.1034; Found: 310.1034.

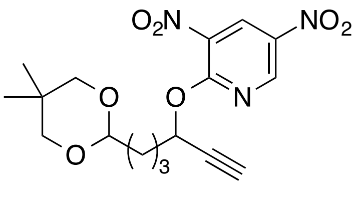

2-[4-(3,5-Dinitro-2-pyridyloxy)-5-hexynyl]-5,5-dimethyl-1,3-dioxane (4h). The title compound was prepared according to the general procedure using NaH (60% dispersion in mineral oil, 188 mg, 4.7 mmol), propargyic alcohol (600 mg, 2.8 mmol), 2-chloro-3,5-dinitropyridine (478 mg, 2.3 mmol). The crude material was purified by silica gel chromatography (hexanes/ethyl acetate = 6:1) to afford the title compound as a light yellow oil (500 mg, 56% yield).

^{1}H NMR (400 MHz, $CDCl_3$) δ 9.27 (d, *J* = 2.6 Hz, 1H), 9.05 (d, *J* = 2.6 Hz, 1H), 5.88 (app. td, *J* = 6.6, 2.1 Hz, 1H), 4.46 – 4.42 (m, 1H), 3.65 – 3.52 (m, 2H), 3.41 (dd, *J* = 11.2, 2.7 Hz, 2H), 2.52 (d, *J* = 2.1 Hz, 1H), 2.08-2.05 (m, 2H), 1.72-1.69 (m, 4H), 1.16 (s, 3H), 0.70 (s, 3H).

^{13}C NMR (101 MHz, $CDCl_3$) δ 157.8, 147.4, 138.4, 132.7, 130.8, 101.7, 79.9, 77.1, 75.2, 69.2, 34.5, 34.0, 30.1, 22.9, 21.8, 19.4.

IR (neat) 3291, 2954, 2122, 1603, 1591, 1341, 1307, 960, 830, 715 cm^{-1}.

HRMS (CI) Calcd. for $C_{17}H_{22}N_3O_7$ ($[M+H]^+$): 380.1452; Found: 380.1454.

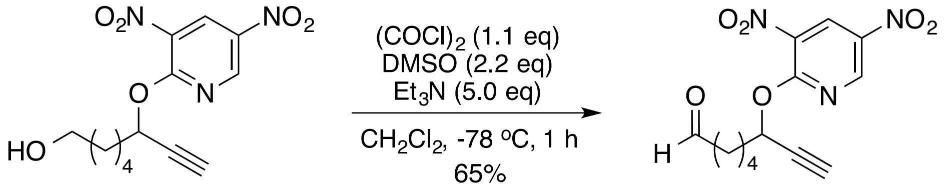

6-(3,5-Dinitro-2-pyridyloxy)-7-octynal (4i). A 100 mL round-bottom flask with a 1.5 cm stir bar was evacuated and refilled with N_2 three times. $(COCl)_2$ (0.28 mL, 3.3 mmol) and CH_2Cl_2 (5 mL) was added via syringe. The flask was cooled to –78 °C. A solution of DMSO (0.47 mL, 6.6 mmol) in CH_2Cl_2 (5 mL) was added to the flask by syringe at –78 °C. After addition, the mixture was stirred at this temperature for 10 min before a solution of alcohol (927 mg, 3.0 mmol) in CH_2Cl_2 (5 mL) was added dropwise. The mixture was stirred at –78 °C for further 0.5 h. Then Et_3N (2.0 mL, 15.6 mmol) was added once. After that, the cooling bath was removed and the mixture was allowed to reach room temperature. H_2O (20 mL) was added and the aqueous phase was extracted twice with CH_2Cl_2 (60 mL). The combined organic phases were washed with brine, dried over $MgSO_4$, filtered and then concentrated under reduced pressure. The crude oil was purified by silica gel chromatography (hexane/EtOAc = 3:1) to afford the product as a colorless oil (600 mg, 65% yield).

^{1}H NMR (400 MHz, $CDCl_3$) δ 9.78 (t, *J* = 1.7 Hz, 1H), 9.29 (d, *J* = 2.5 Hz, 1H), 9.06 (d, *J* = 2.6 Hz, 1H), 5.89 (app. td, *J* = 6.4, 2.1 Hz, 1H), 2.58 – 2.46 (m, 3H), 2.13 – 2.00 (m, 2H), 1.78 – 1.61 (m, 4H).

^{13}C NMR (101 MHz, $CDCl_3$) δ 202.0, 157.7, 147.4, 138.5, 132.7, 130.9, 79.7, 75.4, 68.9, 43.6, 34.2, 24.3, 21.4.

IR (neat) 3287, 2939, 2122, 1720, 1602, 1590, 1335, 955, 830 cm^{-1}.

HRMS (CI) Calcd. for $C_{13}H_{13}N_3O_6$ ($[M+H]^+$): 308.0877; Found: 308.0872.

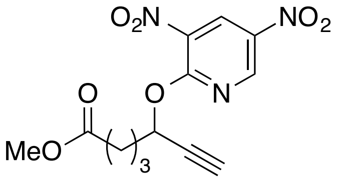

Methyl 5-(3,5-dinitro-2-pyridyloxy)-6-heptynoate (4j). The title compound was prepared according to the general procedure using NaH (60% dispersion in mineral oil, 330 mg, 8.0 mmol), propargyic alcohol (667 mg, 4.8 mmol), 2-chloro-3, 5-dinitropyridine (816 mg, 4.0 mmol). The crude material was purified by silica gel

chromatography (hexanes/ethyl acetate = 6:1) to afford the title compound as a light yellow oil (918 mg, 75% yield).

^{1}H NMR (400 MHz, $CDCl_3$) δ 9.29 (d, J = 2.6 Hz, 1H), 9.07 (d, J = 2.6 Hz, 1H), 5.90 (app. td, J = 6.2, 2.1 Hz, 1H), 3.67 (s, 3H), 2.54 (d, J = 2.1 Hz, 1H), 2.43 (t, J = 7.3 Hz, 2H), 2.15 – 1.87 (m, 4H).

^{13}C NMR (101 MHz, $CDCl_3$) δ 173.3, 157.7, 147.4, 138.5, 132.7, 130.9, 79. 6, 75.5, 68.8, 51.6, 33.8, 33.2, 20.2.

IR (neat) 3289, 2955, 2122, 1730, 1603, 1590, 1336, 956, 830 cm^{-1}.

HRMS (CI) Calcd. for $C_{13}H_{13}N_3O_7$ ([M+H]$^+$): 324.0826; Found: 324.0829.

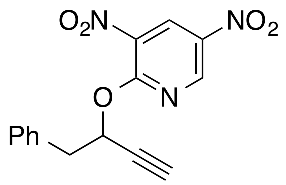

2-(1-Benzyl-2-propynyloxy)-3,5-dinitropyridine (4k). The title compound was prepared according to the general procedure using NaH (60% dispersion in mineral oil, 240 mg, 6.0 mmol), propargyic alcohol (522 mg, 3.6 mmol), 2-chloro-3, 5-dinitropyridine (612 mg, 3.0 mmol). The crude material was purified by silica gel chromatography (hexanes/ethyl acetate = 15:1) to afford the title compound as a light yellow solid (750 mg, 80% yield).

Mp: 108–109 ºC.

^{1}H NMR (400 MHz, $CDCl_3$) δ 9.26 (d, J = 2.6 Hz, 1H), 9.06 (d, J = 2.7 Hz, 1H), 7.41 – 7.25 (m, 5H), 6.07 (ddd, J = 7.7, 5.6, 2.1 Hz, 1H), 3.44 – 3.32 (m, 2H), 2.58 (d, J = 2.0 Hz, 1H).

^{13}C NMR (101 MHz, $CDCl_3$) δ 157.7, 147.3, 138.5, 135.1, 132.6, 130.9, 129.8, 128.5, 127.3, 79.6, 75.9, 69.8, 41.1.

IR (neat) 3284, 2927, 2124, 1603, 1590, 1348, 1282, 980, 728, 718 cm^{-1}.

HRMS (CI) Calcd. for $C_{15}H_{12}N_3O_5$([M+H]$^+$): 314.0771; Found: 314.0772.

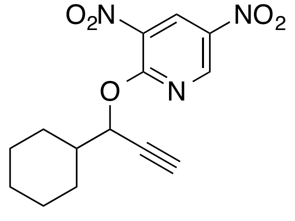

2-(1-Cyclohexyl-2-propynyloxy)-3,5-dinitropyridine (4l). The title compound was prepared according to the general procedure using NaH (60% dispersion in mineral oil, 240 mg, 6.0 mmol), propargyic alcohol (493 mg, 4.8 mmol), 2-chloro-3, 5-dinitropyridine (612 mg, 3.0 mmol). The crude material was purified by silica gel chromatography (hexanes/ethyl acetate = 20:1) to afford the title compound as a light yellow oil (800 mg, 88% yield).

^{1}H NMR (400 MHz, $CDCl_3$) δ 9.29 (d, *J* = 2.6 Hz, 1H), 9.06 (d, *J* = 2.6 Hz, 1H), 5.71 (dd, *J* = 5.5, 2.2 Hz, 1H), 2.51 (d, *J* = 2.1 Hz, 1H), 2.02 – 1.91 (m, 3H), 1.84-1.80 (m, 2H), 1.75 – 1.67 (m, 1H), 1.39 – 1.17 (m, 6H).

^{13}C NMR (101 MHz, $CDCl_3$) δ 158.1, 147.4, 138.3, 132.7, 130.8, 79.1, 75.7, 73.6, 41.9, 28.2, 28.0, 26.1, 25.7, 25.6.

IR (neat) 3292, 2930, 2123, 1603, 1590, 1343, 1330, 1232, 962, 829 cm^{-1}.

HRMS (CI) Calcd. for $C_{14}H_{16}N_3O_5$ ($[M+H]^+$): 306.1084; Found: 306.1085.

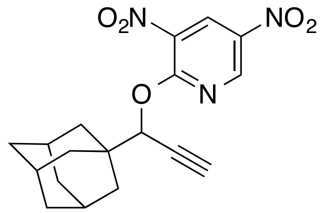

3,5-Dinitro-2-(1-adamantyl-2-propynyloxy)pyridine (4m). The title compound was prepared according to the general procedure using NaH (60% dispersion in mineral oil, 160 mg, 4.0 mmol), propargyic alcohol (412 mg, 2.4 mmol), 2-chloro-3, 5-dinitropyridine (408 mg, 2.0 mmol). The reaction was stirred at room temperature for 12 h. The crude material was purified by silica gel chromatography (hexanes/ethyl acetate = 10:1) to afford the title compound as a white solid (470 mg, 69% yield).

Mp: 95–96 °C.

^{1}H NMR (400 MHz, $CDCl_3$) δ 9.29 (d, *J* = 2.5 Hz, 1H), 9.08 (d, *J* = 2.6 Hz, 1H), 5.47 (d, *J* = 2.1 Hz, 1H), 2.49 (d, *J* = 2.2 Hz, 1H), 2.07-2.05 (m, 3H), 1.84 – 1.66 (m, 12H).

^{13}C NMR (101 MHz, $CDCl_3$) δ 158.4, 147.5, 138.3, 132.6, 130.9, 77.6, 76.1, 69.3, 37.7, 37.2, 36.7, 28.0.

IR (neat) 3296, 2904, 2850, 2114, 1607, 1588, 1346, 1328, 960, 751cm^{-1}.

HRMS (CI) Calcd. for $C_{18}H_{20}N_3O_5$ ($[M+H]^+$): 358.1397; Found: 358.1400.

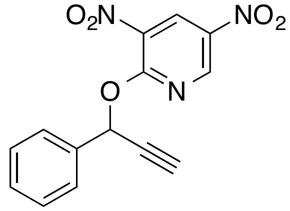

3,5-Dinitro-2-(1-phenyl-2-propynyloxy)pyridine (4n). The title compound was prepared according to the general procedure using NaH (60% dispersion in mineral oil, 160 mg, 4.0 mmol), 1-Phenyl-2-propyn-1-ol (317 mg, 2.4 mmol), 2-chloro-3, 5-dinitropyridine (408 mg, 2.0 mmol). The crude material was purified by silica gel chromatography (hexanes/ethyl acetate = 5:1) to afford the title compound as a yellow solid (570 mg, 95% yield).

Mp: 122–123 °C.

^{1}H NMR (400 MHz, CDCl3) δ 9.32 (d, *J* = 2.6 Hz, 1H), 9.07 (d, *J* = 2.6 Hz, 1H), 7.70 – 7.62 (m, 2H), 7.48 – 7.38 (m, 3H), 7.02 (d, *J* = 2.3 Hz, 1H), 2.74 (d, *J* = 2.2 Hz, 1H).

^{13}C NMR (101 MHz, CDCl3) δ 157.4, 147.3, 138.6, 135.2, 132.8, 131.0, 129.6, 128.9, 127.6, 79.3, 77.1, 70.4.

IR (neat) 3286, 2922, 2122, 1603, 1589, 1517, 1344, 1233, 758, 702 cm^{-1}.

HRMS (CI) Calcd. for $C_{14}H_{10}N_3O_7$ ([M+H]$^+$): 300.0615; Found: 300.0610.

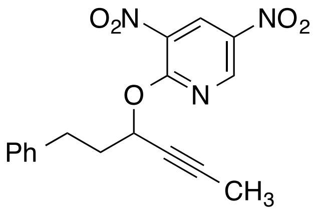

3,5-Dinitro-2-(1-phenethyl-2-butynyloxy)pyridine (4a-Me). The title compound was prepared according to the general procedure using NaH (60% dispersion in mineral oil, 80 mg, 2.0 mmol), propargyic alcohol (209 mg, 1.2 mmol), 2-chloro-3,5-dinitropyridine (204 mg, 1.0 mmol). The crude material was purified by silica gel chromatography (hexanes/ethyl acetate = 10:1) to afford the title compound as a light yellow solid (200 mg, 59% yield).

Mp: 81–82 °C.

^{1}H NMR (400 MHz, $CDCl_3$) δ 9.29 (d, *J* = 2.7 Hz, 1H), 9.08 (d, *J* = 2.6 Hz, 1H), 7.34 – 7.28 (m, 2H), 7.24-7.21 (m, 3H), 5.93 – 5.77 (m, 1H), 2.99 – 2.90 (m, 2H), 2.43 – 2.28 (m, 2H), 1.88 (d, *J* = 2.1 Hz, 3H).

^{13}C NMR (101 MHz, $CDCl_3$) δ 158.1, 147.5, 140.5, 138.1, 132.7, 130.7, 128.5, 128.4, 126.2, 84.0, 75.5, 69.5, 36.6, 31.1, 3.7.

IR (neat) 3086, 2929, 2249, 1601, 1587, 1344, 1330, 994, 829, 747, 703 cm^{-1}.

HRMS (CI) Calcd. for $C_{17}H_{16}N_3O_5$ ([M+H]$^+$): 342.1084; Found: 342.1067.

4. General Procedure for Rearrangement

CuTC (7.6 mg, 0.040 mmol) and (*R*)-Tol-BINAP (33.0 mg, 0.048 mmol) were added in turn to a 4 mL vial with a 1 cm stir bar. The vial was capped with a PTFE-lined cap and then it was evacuated and refilled with N_2 three times. Then toluene (1.0 mL) was added via 2 mL syringe. The mixture was stirred at room temperature for 1 h. The vial was then cooled down to –40 °C using a cooling bath and a solution of 3,5-dinitro-propargyloxypyridine (0.4 mmol) in toluene (1.0 mL) was added dropwise. After the addition is complete, the reaction mixture was stirred at –40 °C for 18 h. Then the mixture was transferred to a 25 mL round-bottom flask via pipette and solvent was removed under reduced pressure. The residue was purified by silica gel chromatography (hexane/ethyl acetate) to afford the corresponding pyridones.

A second run was performed using (*S*)-Tol-BINAP.

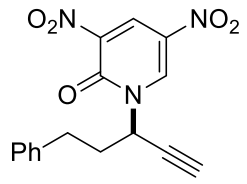

(*R*)-3,5-Dinitro-1-(1-phenethyl-2-propynyl)-1*H*-pyridin-2-one (5a). The title compound was prepared according to the general procedure. The crude material was purified by silica gel chromatography (hexanes/ethyl acetate = 10:1 to 5:1) to afford the title compound as a light yellow solid.

First run: 110 mg (85% yield, 97.5:2.5 er); Second run: 124 mg (95% yield, 97.5:2.5 er).

The er was determined on a Chiracel IB column (hexane/*i*-PrOH = 70:30; 35 °C; 1.0 mL/min); retention times for compound obtained using (*R*)-Tol-BINAP: t_R (major) = 18.7 min; t_R (minor) = 25.1 min.

Mp: 124–125 °C.

$[\alpha]^{24}_D$ = –104.5 (*c* = 1.0, $CHCl_3$); 97.5:2.5 er, from (*R*)-Tol-BINAP.

^{1}H NMR (400 MHz, $CDCl_3$) δ 9.24 (d, *J* = 3.0 Hz, 1H), 8.94 (d, *J* = 3.0 Hz, 1H), 7.31 – 7.25 (m, 2H), 7.21-7.18 (m, 3H), 5.88 (ddd, *J* = 7.9, 5.4, 2.5 Hz, 1H), 2.86 – 3.0 (m, 3H), 2.44 – 2.26 (m, 2H).

^{13}C NMR (101 MHz, $CDCl_3$) δ 152.3, 140.7, 138.7, 136.0, 131.9, 128.7, 128.1, 127.8, 126.6, 79.7, 77.5, 51.62, 35.5, 31.5.

IR (neat) 3255, 3098, 2935, 2123, 1691, 1526, 1340, 1245, 1235, 744 cm^{-1}.

HRMS (CI) Calcd. for $C_{16}H_{14}N_3O_5$ ($[M+H]^+$): 328.0928; Found: 328.0929.

Gram Scale: CuTC (95 mg, 0.5 mmol) and (*R*)-Tol-BINAP (407 mg, 0.6 mmol) were added in turn to a 100 mL flask with a 2.5 cm stir bar. The flask was evacuated and refilled with N_2 three times. Then toluene (12.5 mL) was added via syringe. The mixture was stirred at room temperature for 1 h. The vial was then cooled down to –40 °C using a cooling bath and a solution of 3,5-dinitro-2-(1-phenethyl-2-propynyloxy)pyridine (1.64 g, 5.0 mmol) in toluene (12.5 mL) was added dropwise by syringe. After the addition was complete, the reaction mixture was stirred at –40 °C for 18 h. Then the mixture was concentrated under reduced pressure. The residue was purified by silica gel chromatography (hexane/ethyl acetate = 5:1) to afford the title product as a yellow solid (1.3 g, 81% yield). The er of the product was determined to be 97.2:2.5.

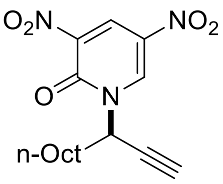

(*R*)-3,5-Dinitro-1-(1-octyl-2-propynyl)-1*H*-pyridin-2-one (5b). The title compound was prepared according to the general procedure. The crude material was purified by silica gel chromatography (hexanes/ethyl acetate = 10:1) to afford the title compound as a light yellow solid.

First run: 100 mg (75% yield, 96.5:3.5 er); Second run: 103 mg (77% yield, 97:3 er).

The er was determined on a Chiracel IB column (hexane/*i*-PrOH = 80:20; 35 °C; 1.0 mL/min); retention times for compound obtained using (*R*)-Tol-BINAP: t_R (minor) = 15.7 min; t_R (major) = 18.1 min.

Mp: 57–58 °C.

$[\alpha]^{24}_D$ = –73.1 (*c* = 1.0, $CHCl_3$); 96.5:3.5 er, from (*R*)-Tol-BINAP.

^{1}H NMR (400 MHz, $CDCl_3$) δ 9.33 (d, *J* = 3.0 Hz, 1H), 9.04 (d, *J* = 3.1 Hz, 1H), 5.83 (ddd, *J* = 8.2, 5.1, 2.4 Hz, 1H), 2.82 (d, *J* = 2.5 Hz, 1H), 2.03 – 1.75 (m, 2H), 1.56 – 1.18 (m, 12H), 0.87 (t, *J* = 6.6 Hz, 3H).

^{13}C NMR (101 MHz, CDCl3) δ 152.3, 140.6, 136.3, 132.0, 127.9, 78.9, 78.1, 51.7, 35.8, 31.7, 29.2, 29.1, 28.7, 25.4, 22.6, 14.1.

IR (neat) 3255, 2926, 2122, 1690, 1569, 1332, 713 cm^{-1}.

HRMS (CI) Calcd. for $C_{16}H_{22}N_3O_5$ ($[M+H]^+$): 336.1554; Found: 336.1552.

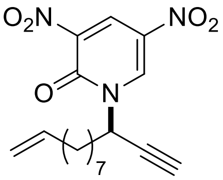

(*R*)-1-(1-Ethynyl-9-decenyl)-3,5-dinitro-1*H*-pyridin-2-one (5c). The title compound was prepared according to the general procedure. The crude material was purified by silica gel chromatography (hexanes/ethyl acetate = 10:1) to afford the title compound as a yellow oil.

First run: 123 mg (87% yield, 96.5:3.5 er); Second run: 127 mg (89% yield, 97:3 er).

The er was determined on a Chiracel IB column (hexane/*i*-PrOH = 70:30; 35 °C; 1.0 mL/min); retention times for compound obtained using (*R*)-Tol-BINAP: t_R (minor) = 12.7 min; t_R (major) = 14.8 min.

$[\alpha]^{24}_D$ = –75.5 (*c* = 1.0, $CHCl_3$); 96.5:3.5% er, from (*R*)-Tol-BINAP.

^{1}H NMR (400 MHz, $CDCl_3$) δ 9.33 (d, *J* = 3.1 Hz, 1H), 9.04 (d, *J* = 3.0 Hz, 1H), 5.98 – 5.62 (m, 2H), 5.21 – 4.76 (m, 2H), 2.83 (d, *J* = 2.4 Hz, 1H), 2.10 – 1.74 (m, 4H), 1.56 – 1.27 (m, 10H).

^{13}C NMR (101 MHz, $CDCl_3$) δ 152.3, 140.6, 139.0, 136.3, 132.0, 127.9, 114.3, 78.9, 78.1, 51.7, 35.8, 33.7, 29.1, 28.8, 28.7, 28.6, 25.4.

IR (neat) 3254, 2928, 2122, 1689, 1569, 1331, 1235, 713 cm^{-1}.

HRMS (CI) Calcd. for $C_{17}H_{22}N_3O_5$ ($[M+H]^+$): 348.1554; Found: 348.1555.

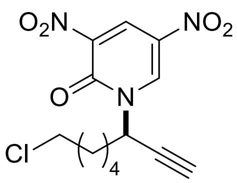

(*R*)-1-[1-(5-Chloropentyl)-2-propynyl]-3,5-dinitro-1H-pyridin-2-one (5d). The title compound was prepared according to the general procedure. The crude material was purified by silica gel chromatography (hexanes/ethyl acetate = 5:1) to afford the title compound as a yellow solid.

First run: 123 mg (95% yield, 95.5:4.5 er); Second run: 115 mg (88% yield, 95.5:4.5 er).

The er was determined on a Chiracel ID column (hexane/*i*-PrOH = 70:30; 35 °C; 1.0 mL/min); retention times for compound obtained using (*R*)-Tol-BINAP: t_R (minor) = 16.9 min; t_R (major) = 18.4 min.

Mp: 74–75 °C.

$[\alpha]^{24}_D$ = –73.2 (*c* = 1.0, $CHCl_3$); 95.5:4.5 er, from (*R*)-Tol-BINAP.

^{1}H NMR (400 MHz, $CDCl_3$) δ 9.33 (d, *J* = 3.1 Hz, 1H), 9.04 (d, *J* = 3.0 Hz, 1H), 5.82 (ddd, *J* = 9.0, 5.0, 2.4 Hz, 1H), 3.53 (t, *J* = 6.5 Hz, 2H), 2.86 (d, *J* = 2.4 Hz, 1H), 2.06 – 1.73 (m, 4H), 1.63 – 1.45 (m, 4H).

^{13}C NMR (101 MHz, $CDCl_3$) δ 152.3, 140.5, 136.2, 132.0, 128.0, 79.2, 77.8, 51.5, 44.6, 35.6, 32.0, 25.9, 24.8.

IR (neat) 3288, 2954, 2129, 1702, 1687, 1331, 1235, 714 cm^{-1}.

HRMS (CI) Calcd. for $C_{13}H_{15}ClN_3O_5$ ($[M+H]^+$): 328.0695; Found: 328.0694.

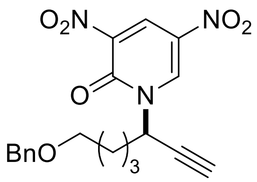

(*R*)-1-{1-[4-(Benzyloxy)butyl]-2-propynyl}-3,5-dinitro-1*H*-pyridin-2-one (5e). The title compound was prepared according to the general procedure. The crude material was purified by silica gel chromatography (hexanes/ethyl acetate = 5:1) to afford the title compound as a light yellow solid.

First run: 140 mg (91% yield, 96.5:3.5 er); Second run: 135 mg (88% yield, 96.5:3.5 er).

The er was determined on a Chiracel IF column (hexane/*i*-PrOH = 60:40; 35 °C; 1.0 mL/min); retention times for compound obtained using (*R*)-Tol-BINAP: t_R (minor) = 11.9 min; t_R (major) = 14.1 min.

Mp: 60–61 °C.

$[\alpha]^{24}_D$ = –57.8 (*c* = 1.0, $CHCl_3$); 96.5:3.5 er, from (*R*)-Tol-BINAP.

^{1}H NMR (400 MHz, $CDCl_3$) δ 9.33 (d, *J* = 3.1 Hz, 1H), 9.03 (d, *J* = 3.1 Hz, 1H), 7.41 – 7.26 (m, 5H), 5.85 (ddd, *J* = 8.6, 5.1, 2.5 Hz, 1H), 4.50 (s, 2H), 3.51 (t, *J* = 5.9 Hz, 2H), 2.86 (d, *J* = 2.4 Hz, 1H), 2.08 – 1.83 (m, 2H), 1.76 – 1.59 (m, 4H).

^{13}C NMR (101 MHz, $CDCl_3$) δ 152.3, 140.6, 138.3, 136.2, 132.0, 128.3, 127.9, 127.6, 79.0, 77.9, 72.9,

69.3, 51.6, 35.4, 28.7, 22.2.

IR (neat) 3236, 2863, 2121, 1697, 1563, 1535, 1336, 741, 713 cm^{-1}.

HRMS (CI) Calcd. for $C_{19}H_{20}N_3O_6$ ($[M]^+$): 386.1347; Found: 386.1342

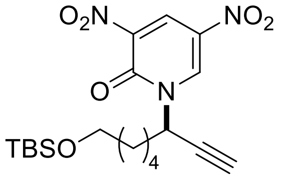

(*R*)-1-[1-(5-tert-butyldimethylsilyloxypentyl)-2-propynyl]-3,5-dinitro-1*H*-pyridin-2-one (5f). The title compound was prepared according to the general procedure. The crude material was purified by silica gel chromatography (hexanes/ethyl acetate = 8:1) to afford the title compound as a yellow solid.

First run: 125 mg (74% yield, 96.5:3.5 er); Second run: 130 mg (76% yield, 96.5:3.5 er).

The er was determined on a Chiracel ID column (hexane/*i*-PrOH = 70:30; 35 °C; 1.0 mL/min); retention times for compound obtained using (*R*)-Tol-BINAP: t_R (minor) = 10.2 min; t_R (major) = 10.9 min.

Mp: 51–52°C.

$[\alpha]^{24}_D$ = –61.6 (*c* = 1.0, $CHCl_3$); 96.5:3.5 er, from (*R*)-Tol-BINAP

^{1}**H** NMR (400 MHz, $CDCl_3$) δ 9.32 (d, *J* = 3.1 Hz, 1H), 9.03 (d, *J* = 3.0 Hz, 1H), 5.82 (ddd, *J* = 8.8, 5.0, 2.4 Hz, 1H), 3.59 (t, *J* = 6.1 Hz, 2H), 2.83 (d, *J* = 2.4 Hz, 1H), 2.05 – 1.77 (m, 2H), 1.59 – 1.35 (m, 6H), 0.87 (s, 9H), 0.03 (s, 6H).

^{13}**C** NMR (101 MHz, $CDCl_3$) δ 152.3, 140.6, 136.3, 132.0, 127.9, 78.9, 78.0, 62.7, 51.6, 35.8, 32.3, 25.9, 25.3, 25.1, 18.3, -5.3.

IR (neat) 3267, 2957, 2128, 1702, 1690, 1569, 1352, 1330, 1094, 714 cm^{-1}.

HRMS (CI) Calcd. for $C_{19}H_{30}N_3O_6Si$ ($[M+H]^+$): 424.1898; Found: 424.1901.

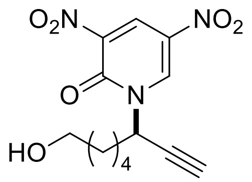

(*R*)-1-[1-(5-Hydroxypentyl)-2-propynyl]-3,5-dinitro-1*H*-pyridin-2-one (5g). The title compound was prepared according to the general procedure. The crude material was purified by silica gel chromatography (hexanes/ethyl acetate = 1:1) to afford the title compound as a yellow solid.

First run: 68 mg (55% yield, 57:43 er); Second run: 60 mg (48% yield, 69:31 er).

The er was determined on a Chiracel ID column (hexane/*i*-PrOH = 60:40; 35 °C; 1.0 mL/min); retention times for compound obtained using (*R*)-Tol-BINAP: t_R (minor) = 11.4 min; t_R (major) = 16.1 min.

MP: 85–86 °C.

^{1}**H** NMR (400 MHz, $CDCl_3$) δ 9.33 (d, *J* = 3.1 Hz, 1H), 9.04 (d, *J* = 3.0 Hz, 1H), 5.82 (ddd, *J* = 8.2, 5.0, 2.4 Hz, 1H), 3.64 (t, *J* = 6.3 Hz, 2H), 2.84 (d, *J* = 2.5 Hz, 1H), 2.06 – 1.77 (m, 2H), 1.63 – 1.43 (m, 7H).

^{13}**C** NMR (101 MHz, $CDCl_3$) δ 152.4, 140.6, 136.2, 132.1, 128.0, 79.0, 78.0, 62.4, 51.6, 35.7, 32.1, 25.2, 24.8.

IR (neat) 3561, 3270, 2932, 2120, 1706, 1697, 1570, 1330, 1235, 714 cm^{-1}.

HRMS (CI) Calcd. for $C_{13}H_{16}N_3O_6$ ($[M+H]^+$): 310.1034; Found: 310.1054.

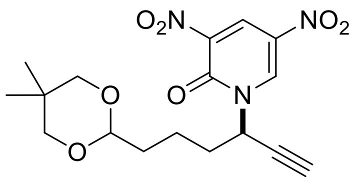

(*R*)-1-{1-[3-(5,5-Dimethyl-1,3-dioxan-2-yl)propyl]-2-propynyl}-3,5-dinitro-1*H*-pyridin-2-one (5h). The title compound was prepared according to the general procedure. The crude material was purified by silica gel chromatography (hexanes/ethyl acetate = 3:1) to afford the title compound as a yellow solid.

First run: 139 mg (91% yield, 96.5:3.5 er); Second run: 129 mg (85% yield, 97:3 er).

The er was determined on a Chiracel IB column (hexane/*i*-PrOH = 60:40; 35 °C; 1.0 mL/min); retention times for compound obtained using (*R*)-Tol-BINAP: t_R (major) = 15.7 min; t_R (minor) = 18.4 min.

Mp: 115–116 °C.

$[\alpha]^{24}_D$ = –71.1 (*c* = 1.0, $CHCl_3$); 96.5:3.5 er, from (*R*)-Tol-BINAP.

^{1}**H** NMR (400 MHz, $CDCl_3$) δ 9.31 (d, *J* = 3.1 Hz, 1H), 9.02 (d, *J* = 3.0 Hz, 1H), 5.83 (ddd, *J* = 8.0, 5.2, 2.4 Hz, 1H), 4.42 (t, *J* = 4.4 Hz, 1H), 3.61 – 3.51 (m, 2H), 3.39 (dd, *J* = 11.2, 2.4 Hz, 2H), 2.83 (d, *J* = 2.4 Hz, 1H), 2.06 – 1.84 (m, 2H), 1.72 – 1.55 (m, 4H), 1.14 (s, 3H), 0.70 (s, 3H).

^{13}**C** NMR (100 MHz, $CDCl_3$) δ 152.3, 140.7, 136.3, 132.0, 127.9, 101.2, 79.0, 77.9, 77.1, 51.6, 35.5, 33.6, 30.1, 22.9, 21.7, 19.8.

IR (neat) 3280, 3081, 2938, 2135, 1700, 1688, 1568, 1329, 1218, 698 cm^{-1}.

HRMS (CI) Calcd. for $C_{17}H_{22}N_3O_7$ ($[M+H]^+$): 380.1452; Found: 380.1454.

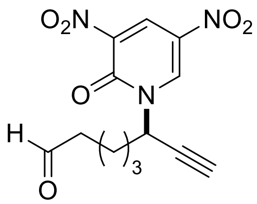

(*R*)-6-(3,5-Dinitro-2-oxo-1*H*-pyrid-1-yl)-7-octynal (5i). The title compound was prepared according to the general procedure. The crude material was purified by silica gel chromatography (hexanes/ethyl acetate = 2:1) to afford the title compound as a yellow solid.

First run: 110 mg (89% yield, 96.5:3.5 er); Second run: 105 mg (85% yield, 96.5:3.5 er).

The aldehyde product was further protected with ethylene glycol. The er of corresponding acetal was determined on a Chiracel IB column (hexane/*i*-PrOH = 60:40; 35 °C; 1.0 mL/min); retention times for compound obtained using (*R*)-Tol-BINAP: t_R (major) = 19.2 min; t_R (minor) = 23.6 min.

Mp: 86–87°C.

$[\alpha]^{24}_{D}$ = –87.1 (c = 1.0, $CHCl_3$); 96.5:3.5 er, from (R)-Tol-BINAP

^{1}H NMR (400 MHz, $CDCl_3$) δ 9.76 (t, J = 1.4 Hz, 1H), 9.33 (d, J = 3.0 Hz, 1H), 9.04 (d, J = 3.0 Hz, 1H), 5.81 (ddd, J = 9.0, 5.0, 2.4 Hz, 1H), 2.86 (d, J = 2.4 Hz, 1H), 2.50 (app. tt, J = 6.9, 1.3 Hz, 2H), 2.05 – 1.80 (m, 2H), 1.75 – 1.65 (m, 2H), 1.59 – 1.50 (m, 2H).

^{13}C NMR (101 MHz, CDCl3) δ 201.5, 152.4, 140.5, 136.3, 132.1, 128.0, 79.3, 77.7, 51.4, 43.2, 35.4, 24.9, 20.9.

IR (neat) 3244, 2944, 2122, 1702, 1686, 1574, 1329, 1313, 744, 714 cm^{-1}.

HRMS (CI) Calcd. for $C_{13}H_{14}N_3O_6$ ($[M+H]^+$): 308.0877; Found: 308.0875.

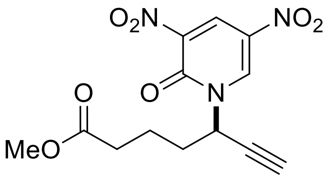

(R)-Methyl 5-(3,5-dinitro-2-oxo-1H-pyrid-1-yl)-6-heptynoate (5j). The title compound was prepared according to the general procedure. The crude material was purified by silica gel chromatography (hexanes/ethyl acetate = 3:1) to afford the title compound as a yellow solid.

First run: 115 mg (92% yield, 96:4 er); Second run: 110 mg (88% yield, 96:4 er).

The er was determined on a Chiracel ID column (hexane/i-PrOH = 60:40; 35 °C; 1.0 mL/min); retention times for compound obtained using (R)-Tol-BINAP: t_R (minor) = 20.3 min; t_R (major) = 26.2 min.

Mp: 111–112 °C.

$[\alpha]^{24}_{D}$ = –77.6 (c = 1.0, $CHCl_3$); 96:4 er, from (R)-Tol-BINAP.

^{1}H NMR (400 MHz, $CDCl_3$) δ 9.32 (d, J = 3.0 Hz, 1H), 9.03 (d, J = 3.0 Hz, 1H), 5.82 (ddd, J = 8.0, 5.1, 2.4 Hz, 1H), 3.65 (s, 3H), 2.88 (d, J = 2.4 Hz, 1H), 2.42 – 2.37 (m, 2H), 2.07 – 1.76 (m, 4H).

^{13}C NMR (101 MHz, $CDCl_3$) δ 172.9, 152.3, 140.6, 136.2, 132.1, 128.0, 79.4, 77.6, 51.7, 51.3, 34.8, 32.6, 20.7.

IR (neat) 3267, 2957, 2128, 1705, 1690, 1569, 1330, 1256, 714 cm^{-1}.

HRMS (CI) Calcd. for $C_{13}H_{14}N_3O_7$ ($[M+H]^+$): 324.0826; Found: 324.0822.

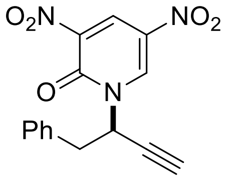

(R)-1-(1-Benzyl-2-propynyl)-3,5-dinitro-1H-pyridin-2-one (5k). The title compound was prepared according to the general procedure. The crude material was purified by silica gel chromatography (hexanes/ethyl acetate = 5:1) to afford the title compound as a light yellow solid.

Mp: 138–139 °C.

First run: 58 mg (46% yield, 62.5:37.5 er); Second run: 65 mg (52% yield, 62:38% er).

The er was determined on a Chiracel IF column (hexane/*i*-PrOH = 60:40; 35 °C; 1.0 mL/min); retention times for compound obtained using (*R*)-Tol-BINAP: t_R (minor) = 9.0 min; t_R (major) = 10.4 min.

^{1}H NMR (400 MHz, $CDCl_3$) δ 9.02 (d, *J* = 3.0 Hz, 1H), 8.80 (d, *J* = 3.1 Hz, 1H), 7.35 – 7.27 (m, 3H), 7.20 – 7.11 (m, 2H), 6.12 (ddd, *J* = 7.3, 3.8, 2.4 Hz, 1H), 3.35 – 3.12 (m, 2H), 2.87 (d, *J* = 2.5 Hz, 1H).

^{13}C NMR (101 MHz, $CDCl_3$) δ 152.3, 140.9, 135.9, 132.9, 132.2, 129.8, 128.9, 128.3, 127.2, 80.2, 77.4, 52.4, 40.1.

IR (neat) 3280, 3083, 2125, 1706, 1562, 1531, 1334, 699, 685 cm^{-1}.

HRMS (CI) Calcd. for $C_{15}H_{12}N_3O_5$ ($[M+H]^+$): 314.0771; Found: 314.0711.

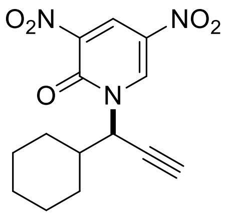

(*R*)-1-(1-Cyclohexyl-2-propynyl)-3,5-dinitro-1*H*-pyridin-2-one (5l). The title compound was prepared according to the general procedure. The crude material was purified by silica gel chromatography (hexanes/ethyl acetate = 5:1) to afford the title compound as a yellow solid.

First run: 100 mg (82% yield, 95.5:4.5 er); Second run: 105 mg (86% yield, 95:5 er).

The er was determined on a Chiracel ID column (hexane/*i*-PrOH = 80:20; 35 °C; 1.0 mL/min); retention times for compound obtained using (*R*)-Tol-BINAP: t_R (major) = 11.0 min; t_R (minor) = 13.4 min.

Mp: 156–157 °C.

$[\alpha]^{24}_D$ = –89.7 (*c* = 1.0, $CHCl_3$); 95.5:4.5 er, from (*R*)-Tol-BINAP

^{1}H NMR (400 MHz, $CDCl_3$) δ 9.24 (d, *J* = 3.1 Hz, 1H), 9.04 (d, *J* = 3.0 Hz, 1H), 5.74 (dd, *J* = 5.7, 2.5 Hz, 1H), 2.81 (d, *J* = 2.5 Hz, 1H), 1.94 – 1.53 (m, 6H), 1.36 – 1.10 (m, 5H).

^{13}C NMR (101 MHz, $CDCl_3$) δ 152.6, 141.0, 136.2, 132.0, 127.5, 79.4, 76.9, 56.5, 41.5, 29.7, 26.9, 25.6, 25.5, 25.2.

IR (neat) 3281, 3081, 2938, 2135, 1689, 1568, 1329, 697 cm^{-1}.

HRMS (CI) Calcd. for $C_{14}H_{16}N_3O_5$ ($[M+H]^+$): 306.1084; Found: 306.1083.

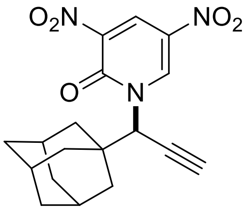

(*R*)-3,5-Dinitro-1-(1-adamantyl -2-propynyl)-1H-pyridin-2-one (5m). The title compound was prepared according to the general procedure. The crude material was purified by silica gel chromatography (hexanes/ethyl acetate = 10:1) to afford the title compound as a yellow solid.

First run: 125 mg (36% yield, 51:48 er); Second run: 30 mg (22% yield, 59:41 er).

The er was determined on a Chiracel ID column (hexane/*i*-PrOH = 70:30; 35 °C; 1.0 mL/min); retention times for compound obtained using (*R*)-Tol-BINAP: t_R (major) = 7.5 min; t_R (minor) = 8.6 min.

^{1}H NMR (400 MHz, $CDCl_3$) δ 9.10 (d, *J* = 3.0 Hz, 1H), 9.03 (d, *J* = 3.1 Hz, 1H), 5.82 (d, *J* = 2.6 Hz, 1H), 2.71 (d, *J* = 2.5 Hz, 1H), 2.12 – 2.03 (m, 3H), 1.77 – 1.51 (m, 12H).

^{13}C NMR (101 MHz, $CDCl_3$) δ 153.1, 141.8, 136.3, 131.9, 127.1, 78.2, 77.2, 59.2, 39.0, 38.2, 36.2, 27.9.

IR (neat) 3248, 2909, 2117, 1695, 1564, 1347, 1328, 1227, 712 cm^{-1}.

HRMS (CI) Calcd. for $C_{18}H_{20}N_3O_5$ ($[M+H]^+$): 358.1397; Found: 358.1403.

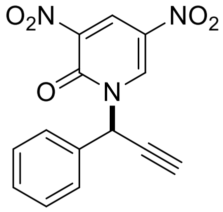

(*R*)-3,5-Dinitro-1-(1-phenyl-2-propynyl)-1*H*-pyridin-2-one (5n). The title compound was prepared according to the general procedure. The crude material was purified by silica gel chromatography (hexanes/ethyl acetate = 5:1) to afford the title compound as a yellow solid.

First run: 83 mg (69% yield, 85.5:14.5 er); Second run: 84 mg (70% yield, 84.5:15.5 er).

The er was determined on a Chiracel IE column (hexane/*i*-PrOH = 80:20; 35 °C; 1.0 mL/min); retention times for compound obtained using (*R*)-Tol-BINAP: t_R (minor) = 16.5 min; t_R (major) = 18.5 min.

Mp: 121–122 °C.

$[\alpha]^{24}_D$ = –107.0 (*c* = 1.0, $CHCl_3$); 85.5:14.5 er, from (*R*)-Tol-BINAP

^{1}H NMR (400 MHz, $CDCl_3$) δ 9.23 (d, *J* = 3.0 Hz, 1H), 8.99 (d, *J* = 3.1 Hz, 1H), 7.59 (dd, *J* = 6.6, 2.9 Hz, 2H), 7.45-7.43 (m, 3H), 7.15 (d, *J* = 2.5 Hz, 1H), 2.98 (d, *J* = 2.5 Hz, 1H).

^{13}C NMR (101 MHz, $CDCl_3$) δ 152.6, 140.6, 136.5, 134.0, 132.1, 130.2, 129.6, 128.3, 127.7, 79.5, 77.5, 53.1.

IR (neat) 3280, 2939, 2134, 1701, 1689, 1568, 1328, 1217, 698 cm^{-1}.

HRMS (CI) Calcd. for $C_{14}H_{10}N_3O_5$ ($[M+H]^+$): 300.0615; Found: 300.0613.

5. Mechanistic Studies

Non-linear Effect Experiments

CuTC (3.6 mg, 0.02 mmol), (*R*)-Tol-BINAP and (*S*)-Tol-BINAP (according to the bellowing ratio, total 0.024 mmol) were added in turn to a 4 mL vial with a 1 cm stir bar. The vial was capped with a PTFE-lined cap and then it was evacuated and refilled with N_2 three times. Then toluene (1.0 mL) was added via 2 mL syringe and the mixture was stirred at room temperature for 1 h. 0.5 mL of the above solution was transferred to another vial under the N_2 atmosphere and the vial was then cooled down to –40 °C using a cooling bath. A solution of 3,5-dinitro-2-(1-phenethyl-2-propynyloxy)pyridine (32.7 mg, 0.1 mmol) in toluene (0.5 mL) was added dropwise. After the addition is complete, the reaction mixture was stirred at –40 °C for 18 h. The reaction mixture was filtered through a pad of silica gel (a pipette with about 5 cm silica gel) and washed with ethyl acetate (10 mL). The filtrate was concentrated under reduced pressure. The residue was dissolved in $CDCl_3$ and CH_2Br_2 (0.1 mmol) was added as internal standard for ^1H NMR analysis. The er was determined by Chiral HPLC.

A second run was performed with the inverse ratio of (*R*)-Tol-BINAP and (*S*)-Tol-BINAP. The results are showed in the parenthesis.

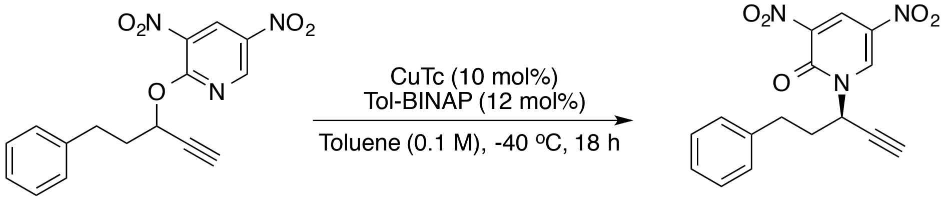

entry	ratio of (*R*) to (*S*)-Tol-BINAP[a]	Product er
1	100:0 (0:100)	97.5:2.5 (2.5:97.5)
2	90:10 (10:90)	95:5 (6:94)
3	80:20 (20:80)	91:9 (11:89)
4	70:30 (30:70)	78.5:21.5 (21.5:78.5)
5	60:40 (40:60)	66:34 (33:67)

Kinetic Resolution Experiments

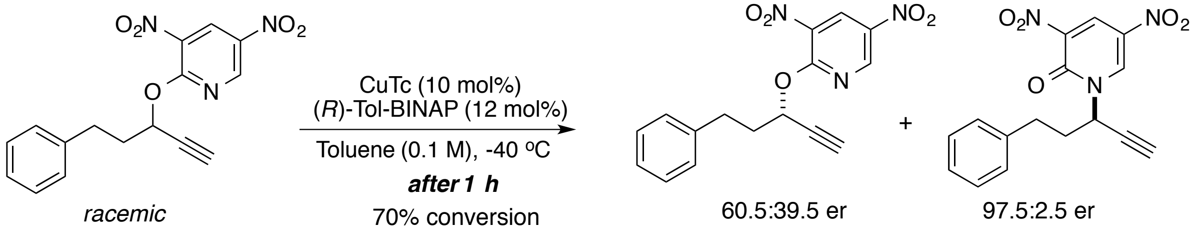

The reaction was performed according to the general procedure of rearrangement utilizing 3,5-dinitro-2-(1-phenethyl-2-propynyloxy)pyridine (32.7 mg, 0.1 mmol), CuTC (1.9 mg, 0.01 mmol) and (*R*)-Tol-BINAP (8.1 mg, 0.012 mmol). After 1 h, the reaction was quenched with AcOH (12 mg, 0.2 mmol) at –40 °C. The reaction mixture

was filtered through a pad of silica gel (a pipette with about 5 cm silica gel) and washed with ethyl acetate (10 mL). The filtrate was concentrated under reduced pressure. The residue was dissolved in $CDCl_3$ and CH_2Br_2 (0.1 mmol) was added as internal standard for ^1H NMR analysis to determine the reaction conversion (70%).

Both the er of product and starting material were determined on a Chiracel IB column (hexane/*i*-PrOH = 70:30; 35 °C; 1.0 mL/min); retention times for product: t_R (major) = 18.1 min; t_R (major) = 24.2 min; 97.5:2.5 er; retention times for starting material: t_R (minor) = 9.7 min; t_R (major) = 10.6 min; 60.5:39.5 er

Isotopic Labelling Experiments

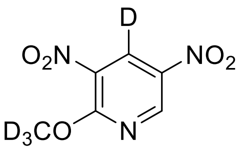

2-(Methoxy-*d*$_3$)-3,5-dinitropyridine-4-*d* (S-1). A 20 mL glass vial was charged with 2-chloro-3,5-dinitropyridine (1.02 g, 5.0 mmol, 1 eq.) and a 1.5 cm stir bar. The vial was fitted with a PTFE-lined cap and evacuated and refilled with N_2 three times through a needle. CD_3OD (5.0 mL) was added to the vial, which was stirred to give a suspension. A solution of NaO*t*-Bu (530 mg, 5.5 mmol, 1.1 eq.) in CD_3OD (5 mL, 1.1 M) was added via a 6 mL syringe and the resulting orange solution stirred for 48 h at ambient temperature. The mixture was transferred to a separating funnel with CH_2Cl_2 (10 mL) and washed sequentially with D_2O (8 mL) and phosphate buffer (20 mL, pH 7). The organic phase was separated, dried over $MgSO_4$ and concentrated under reduced pressure to afford the title compound as a pale yellow solid (955 mg, 94%, 97 atom% D at C4).

Mp: 94–95 °C

^{1}H NMR (400 MHz, $CDCl_3$) δ 9.28 (s, 1H).

^{13}C NMR (101 MHz, $CDCl_3$) δ 159.4, 147.7, 138.1, 132.5, 130.5 (t, $^1J_{CD}$ = 26.8 Hz), 56.7 – 55.1 (m).

IR (neat) 3071, 3035, 2302, 1580, 1513, 1337, 1317, 1201, 829, 679 cm^{-1}.

HRMS (EI) Calcd. for $C_6HD_4N_3O_5$ ($[M]^+$): 204.0480; Found: 204.0483.

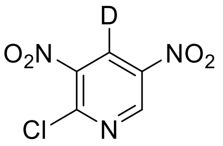

2-Chloro-3,5-dinitropyridine-4-*d* (S-2). A 4 mL glass vial was charged with 2-(methoxy-*d*$_3$)-3,5-dinitropyridine-4-*d* (216 mg, 1.06 mmol, 1 eq.) and a 1 cm stir bar. The vial was fitted with a PTFE-lined cap and evacuated and refilled with N_2 three times through a needle. DMF (1.25 mL) was added *via* a 2 mL syringe, followed by $POCl_3$ (250 µL, 2.66 mmol, 2.5 eq.). The mixture was heated to 80 °C and stirred for 12 h. The reaction was allowed to cool to room temperature, diluted with phosphate buffer (10 mL, pH 7) and extracted with EtOAc (2 x 10 mL). The combined organic extracts were washed with saturated aqueous NaOAc (10 mL), separated, dried over $MgSO_4$ and concentrated under reduced pressure to afford the title compound as a yellow

oil that solidified on standing (142 mg, 65%, 97 atom% D at C4). The product was used in the next step without further purification.

^{1}H NMR (400 MHz, $CDCl_3$) δ 9.46 (s, 1H).

^{13}C NMR (101 MHz, $CDCl_3$) δ 148.8, 147.0, 142.6, 129.3 (t, $^1J_{CD}$ = 27.3 Hz).

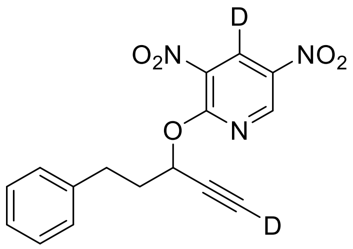

3,5-Dinitro-2-((5-phenylpent-1-yn-3-yl-1-*d*)oxy)pyridine-4-*d* (4a-D). A flame-dried 10 mL round-bottom flask was charged with NaH (55 mg, 1.36 mmol, 60% dispersion in mineral oil, 2 eq.) and a 1 cm stir bar. The flask was fitted with a rubber septum and evacuated and refilled with N_2 three times. THF (0.5 mL) was added to the flask *via* syringe and the resulting suspension cooled to 0 °C. A solution of 5-phenyl-1-pentyn-3-ol (131 mg, 0.82 mmol, 1.2 eq.) in THF (1.1 mL) was prepared in a separate flask and added dropwise to the above solution over 3 minutes. The mixture was stirred at 0 °C for 5 minutes and then allowed to warm to room temperature over 20 minutes. The flask was cooled to 0 °C and a solution of 2-chloro-3,5-dinitropyridine-4-*d* (139 mg, 0.68 mmol, 1 eq.) in THF (1.4 mL) was added dropwise over 3 minutes. The resulting solution was allowed to warm to room temperature and stirred for 2 hours. The reaction was quenched by the dropwise addition of D_2O (3 mL). The pH was neutralised with phosphate buffer (10 mL, pH 7) and the mixture was extracted with EtOAc (3 x 20 mL). The combined organic extracts were dried over $MgSO_4$ and concentrated under reduced pressure. The residue was purified by column chromatography (5–10% EtOAc in hexane) to afford the title compound as a pale yellow solid (124 mg, 55%, 78 atom% D at C4).

Mp: 89–90 °C

^{1}H NMR (400 MHz, $CDCl_3$) δ 9.29 (s, 1H), 7.35 – 7.20 (m, 5H), 5.85 (app. t, J = 6.5 Hz, 1H), 2.97 (t, J = 7.5 Hz, 1H), 2.51 – 2.31 (m, 2H).

^{13}C NMR (101 MHz, $CDCl_3$) δ 157.8, 147.4, 140.1, 138.5, 132.7, 131.4 – 130.4 (m), 128.6, 128.4, 126.4, 79.6 – 79.2 (m), 75.3 (t, $^1J_{CD}$ = 38.0 Hz), 36.2, 31.0.

IR (neat) 3086, 2925, 2306, 1980, 1581, 1341, 1194, 997, 705 cm^{-1}.

Crossover Experiment

CuTC (3.8 mg, 0.02 mmol, 0.2 eq.) and (*R*)-tol-BINAP (16.3 mg, 0.02 mmol, 0.2 eq.) were added in turn to a 4 mL vial equipped with a 1 cm stir bar. The vial was fitted with a PTFE-lined cap and evacuated and refilled with N_2 three times. Toluene (0.5 mL) was added *via* a 1 mL syringe and the mixture was stirred at room temperature for 1 h. The vial was cooled down to −40 °C using a cooling bath and a solution of 3,5-dinitro-2-((5-phenylpent-1-yn-3-yl-1-*d*)oxy)pyridine-4-*d* **4ad** (32.9 mg, 0.1 mmol, 1 eq.) and 2-(1-(4-(benzyloxy)butyl)-2-

propynyloxy)-3,5-dinitropyridine **4e** (38.5 mg, 0.1 mmol, 1 eq.) in toluene (0.5 mL) was added dropwise over 5 minutes. After the addition was complete, the reaction mixture was stirred at –40 °C for 18 h. The mixture was transferred to a 25 mL round-bottom flask *via* pipette and the solvent was removed under reduced pressure. The residue was purified by preparative TLC with 20% EtOAc in hexane to afford the following compounds.

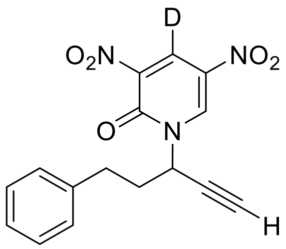

(*R*)-3,5-Dinitro-1-(5-phenylpent-1-yn-3-yl)pyridin-2(1H)-one-4-*d* (5a-D). (27 mg, 83%, 97:3 er, 78 atom% D at C4).

The er was determined on a CHIRALPAK IB column (hexane/*i*-PrOH = 70:30; 35 °C; 1.0 mL/min); retention times for compound obtained using (*R*)-Tol-BINAP: t_R (major) = 18.6 min; t_R (minor) = 23.8 min.

^{1}H NMR (400 MHz, $CDCl_3$) δ 9.24 (s, 1H), 7.31 – 7.14 (m, 5H), 5.87 (ddd, *J* = 7.9, 5.4, 2.5 Hz, 1H), 3.05 – 2.82 (m, 2H), 2.95 (d, *J* = 2.5 Hz, 1H), 2.44 – 2.23 (m, 2H).

^{13}C NMR (101 MHz, $CDCl_3$) δ 152.4, 140.7, 138.7, 136.1, 131.2 – 130.4 (m), 128.8, 128.2, 127.8, 126.7, 79.7, 77.6, 51.7, 35.6, 31.6.

IR (neat) 3286, 3079, 2924, 1701, 1685, 1572, 1327, 1228, 704 cm^{-1}.

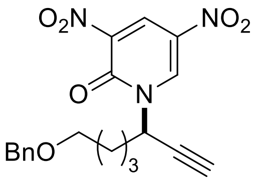

(*R*)-1-(1-(4-(Benzyloxy)butyl)-2-propynyl)-3,5-dinitro-1H-pyridin-2-one (5e) (31 mg, 80%, 97:3 er).

The er was determined on a CHIRALPAK IF column (hexane/*i*-PrOH = 60:40; 35 °C; 1.0 mL/min); retention times for compound obtained using (*R*)-Tol-BINAP: t_R (minor) = 12.1 min; t_R (major) = 15.2 min.

Spectroscopic data were identical to those obtained previously.

Additional information on the 'cross-over' experiments:

The rearrangement of deuterated substrate **4a-D** was performed in d_8-toluene and followed by ^{1}H NMR; product **5a-D** was observed, bearing a terminal proton and no terminal deuteron-contiaining products were observed. The rearrangement of non-deuterated substrate **4a** was performed in d_8-toluene and gave a comparable results with no alkyne deuteration observed. Under no circumstances was pyridone-exchange observed during 'cross-over' experiments, indicating unambiguously that pyridone exchange does not occur. Exchange of the alkyne deuteron was rationalized by the presence of adventurous moisture, despite repeated

efforts to remove moisture from the system – nevertheless, the alkyne–D/H exchange does not cange the conclusion relating to pyridone exchange.

Kinetics Experiments

CuTC (X mmol, Y eq.) and (*R*)-tol-BINAP (X mmol, Y eq.) were added in turn to a 4 mL vial equipped with a 1 cm stir bar. The vial was fitted with a PTFE-lined cap and evacuated and refilled with N_2 three times. Toluene-*d*8 (0.5 mL) was added *via* a 1 mL syringe and the mixture was stirred at room temperature for 1 h. In a separate vial 3,5-dinitro-2-(1-phenethyl-2-propynyloxy)pyridine **4a** (65.4 mg, 0.2 mmol, 1 eq.) and 1,3,5-trimethoxybenzene (11.1 mg, 0.066 mmol, 0.33 eq., internal standard) were dissolved in toluene-d_8 (0.5 mL). An NMR tube was equipped with a septum and evacuated and refilled with N_2 three times. The solution containing catalyst was transferred to the NMR tube *via* 1 mL syringe and cooled to –78 °C in a CO_2(s)/acetone bath. The solution of starting material 4**a** was then added dropwise down the side of the NMR tube over 2 minutes. The NMR tube was immediately transferred to an NMR spectrometer at –40 °C. ^{1}H NMR spectra were recorded each minute over 2 h. The disappearance of starting material was measured using the integral of the peak for the propargylic proton at 5.45 ppm, relative to the internal standard.

**NMR Parameter**	**Value**
Temperature (K)	233.2
Experiment	1D
Number of Scans	4
Relaxation Delay (s)	2.0200
Pulse Width (µs)	12.5000
Acquisition Time (s)	1.9792
Spectrometer Frequency (MHz)	400.13
Spectral Width (Hz)	8278.1
Lowest Frequency (Hz)	–1668.1
Nucleus	1H

6. Derivations of the Product

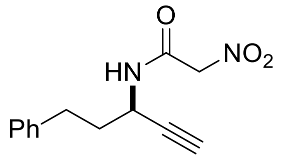

(*R*)-2-Nitro-1-(1-phenethyl-2-propynylamino)-1-ethanone, (*S*)-9.[3] In the air, to an oven-dried 40 mL vial with a 1.5 cm stir bar was added **(*R*)-5a** (310 mg, 0.95 mmol). The vial was closed with a PTFE septum cap, and then it was evacuated and back-filled with nitrogen three times. CH_3OH (15 mL) was added to the vial, followed by cyclohexanone (0.2 mL, 1.9 mmol) and ammonia (7 M in Methanol, 2.7 mL, 19 mmol). The resulting solution was then stirred at 65 °C for 3 h. After the reaction was cooled to room temperature, the solvent was removed under reduced pressure. The residue was diluted with EtOAc (20 mL) and aq. HCl (1 M, 20 mL). The organic phase was separated and aqueous phase was washed with EtOAc (10 mL × 3). The combined organic phase was washed with brine, dried over $NaSO_4$, concentrated. The crude material was purified by silica gel chromatography (hexanes/ethyl acetate = 2:1) to afford the title compound as a white solid (205 mg, 88% yield).

$[\alpha]^{24}_D$ = −0.8 (*c* = 1.0, $CHCl_3$);

Mp: 102−103 °C.

^{1}H NMR (400 MHz, $CDCl_3$) δ 7.33 – 7.27 (m, 2H), 7.24 – 7.17 (m, 3H), 6.79 (d, *J* = 8.2 Hz, 1H), 5.01 (s, 2H), 4.76 (app. qd, *J* = 7.0, 2.3 Hz, 1H), 2.88 – 2.71 (m, 2H), 2.39 (d, *J* = 2.3 Hz, 1H), 2.12 – 1.99 (m, 2H).

^{13}C NMR (101 MHz, $CDCl_3$) δ 159.2, 140.3, 128.6, 128.4, 126.3, 81.2, 77.4, 72.8, 41.9, 36.4, 31.7.

IR (neat) 3273, 3029, 1655, 1549, 683, 670 cm^{-1}.

HRMS (CI) Calcd. for $C_{13}H_{15}N_2O_3$ ($[M+H]^+$): 247.1077; Found: 247.1072.

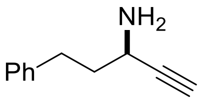

(*R*)-1-phenethylprop-2-ynylamine. In the air, to an oven-dried 50 mL flask with a 1.5 cm stir bar was added **(*R*)-2-Nitro-1-(1-phenethyl-2-propynylamino)-1-ethanone** (148 mg, 0.6 mmol). Then aq HCl (3 M, 10 mL) was added to the flask and the reaction was heated at 90 °C for 24 h. After the reaction was cooled to room temperature, K_2CO_3 was added as small portions to neutralize the acid until pH > 10. The product was extracted with EtOAc (20 mL × 3) and the combined organic phase was washed with brine, dried over $NaSO_4$, concentrated. The crude material was purified by silica gel chromatography (ethyl acetate/methanol = 95:5) to afford the title compound as a yellow oil (73 mg, 76% yield).

$[\alpha]^{24}_D$ = −41.5 (*c* = 1.0, $CHCl_3$);

---

(1) Tohda, Y.; Eiraku, M.; Nakagawa, T.; Usami, Y.; Ariga, M.; Kawashima, T.; Tani, K.; Watanabe, H.; Mori, Y. *Bull. Chem. Soc. Jpn.* **1990**, *63*, 2820–2827.

^{1}H NMR (400 MHz, $CDCl_3$) δ 7.32 – 7.26 (m, 2H), 7.24 – 7.17 (m, 3H), 3.54 (ddd, *J* = 8.4, 6.6, 2.2 Hz, 1H), 2.90 – 2.69 (m, 2H), 2.35 (d, *J* = 2.2 Hz, 1H), 2.00 – 1.86 (m, 2H), 1.55 (s, 2H).

^{13}C NMR (101 MHz, $CDCl_3$) δ 141.3, 128.4 (2C), 125.9, 87.4, 70.8, 42.8, 39.5, 32.2.

IR (neat) 3288, 2922, 2859, 1496, 1454, 744, 699 cm^{-1}.

HRMS (ES) Calcd. for $C_{11}H_{14}N$ ($[M]^+$): 160.1126; Found: 160.1121.

***(S)*-1-phenethylprop-2-ynylamine** was also synthesized using the same method.

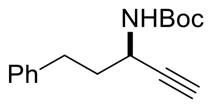

***(R)*-1-Phenethyl-2-propynylamino 2,2-dimethylpropionate, *(S)*-10.**[4] In the air, to an oven-dried 4 mL vial with a 1.0 cm stir bar was added (*R*)-1-phenethylprop-2-ynylamine (29 mg, 0.18 mmol). The vial was closed with a PTFE septum cap, and then it was evacuated and back-filled with nitrogen three times. DCM (1.0 mL) was added to the vial, followed by $NaHCO_3$ (30 mg, 0.36 mmol) and Boc_2O (59 mg, 0.27 mmol). The resulting solution was then stirred at room temperature over night. Then the solid was removed by filtration and filtrate was concentrated under reduced pressure. The crude material was purified by silica gel chromatography (hexanes/ethyl acetate = 12:1) to afford the title compound as a white solid (205 mg, 88% yield).

The er was determined on a Chiracel IF column (hexane/*i*-PrOH = 60:40; 35 °C; 1.0 mL/min); retention times: t_R (major) = 4.9 min; t_R (minor) = 6.4 min, 95:5 er;

$[\alpha]^{24}_D$ = 5.4 (*c* = 1.0, $CHCl_3$);

^{1}H NMR (400 MHz, $CDCl_3$) δ 7.31-7.27 (m, 2H), 7.22-7.18 (m, 3H), 4.78 (d, *J* = 8.7 Hz, 1H), 4.54 – 4.38 (m, 1H), 2.87 – 2.67 (m, 2H), 2.34 (d, *J* = 2.3 Hz, 1H), 2.11 – 1.88 (m, 2H), 1.45 (s, 9H).

^{13}C NMR (101 MHz, $CDCl_3$) δ 154.7, 140.9, 128.4 (2C), 126.0, 83.2, 79.9, 71.5, 42.5, 37.7, 31.9, 28.3.

IR (neat) 3355, 3284, 2984, 2954, 2909, 1685, 1516, 1248, 1165, 702 cm^{-1}.

***(S)*-1-Phenethyl-2-propynylamino 2,2-dimethylpropionate** was also synthesized form (*S*)-1-phenethylprop-2-ynylamine using the same method. The er of this compound was determined to be 95:5 er.

(2) Kiemele, E. R.; Wathier, M.; Bichler, P. A.; Love, J. A. *Org. Lett.* **2016**, *18*, 492–495.

7. X-Ray Crystallographic Data

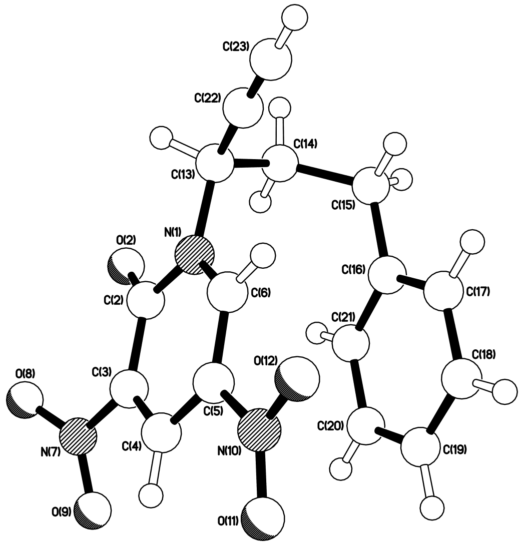

Table 1. Crystal data and structure refinement for CJC1601.

Identification code	CJC1601	
Formula	C16 H13 N3 O5	
Formula weight	327.29	
Temperature	173(2) K	
Diffractometer, wavelength	Agilent Xcalibur PX Ultra A, 1.54184 Å	
Crystal system, space group	Monoclinic, $P2_1$	
Unit cell dimensions	a = 7.24510(14) Å	a = 90°
	b = 9.38179(17) Å	b = 90.4932(16)°
	c = 10.91846(19) Å	g = 90°
Volume, Z	742.12(2) $Å^3$, 2	
Density (calculated)	1.465 Mg/m^3	
Absorption coefficient	0.939 mm^{-1}	
F(000)	340	
Crystal colour / morphology	Colourless platy needles	
Crystal size	0.31 x 0.15 x 0.03 mm^3	
q range for data collection	4.049 to 73.852°	
Index ranges	-9<=h<=8, -11<=k<=11, -13<=l<=13	
Reflns collected / unique	11235 / 2930 [R(int) = 0.0338]	
Reflns observed [F>4s(F)]	2705	
Absorption correction	Analytical	

Max. and min. transmission	0.975 and 0.817
Refinement method	Full-matrix least-squares on F^2
Data / restraints / parameters	2930 / 1 / 218
Goodness-of-fit on F^2	1.093
Final R indices [F>4s(F)]	R1 = 0.0293, wR2 = 0.0686
R indices (all data)	R1 = 0.0341, wR2 = 0.0727
Absolute structure parameter	-0.09(10)
Largest diff. peak, hole	0.153, -0.148 eÅ^{-3}
Mean and maximum shift/error	0.000 and 0.000

Table 2. Bond lengths [Å] and angles [°] for CJC1601.

N(1)-C(6)	1.340(3)
N(1)-C(2)	1.422(3)
N(1)-C(13)	1.505(3)
C(2)-O(2)	1.214(3)
C(2)-C(3)	1.450(3)
C(3)-C(4)	1.355(3)
C(3)-N(7)	1.465(3)
C(4)-C(5)	1.404(3)
C(5)-C(6)	1.356(3)
C(5)-N(10)	1.451(3)
N(7)-O(9)	1.221(3)
N(7)-O(8)	1.223(3)
N(10)-O(11)	1.221(3)
N(10)-O(12)	1.225(3)
C(13)-C(22)	1.466(3)
C(13)-C(14)	1.543(3)
C(14)-C(15)	1.533(3)
C(15)-C(16)	1.514(4)
C(16)-C(17)	1.392(4)
C(16)-C(21)	1.399(3)
C(17)-C(18)	1.386(4)
C(18)-C(19)	1.384(4)
C(19)-C(20)	1.388(4)
C(20)-C(21)	1.383(4)
C(22)-C(23)	1.173(4)
C(6)-N(1)-C(2)	123.35(19)
C(6)-N(1)-C(13)	121.54(18)
C(2)-N(1)-C(13)	115.01(18)
O(2)-C(2)-N(1)	120.2(2)
O(2)-C(2)-C(3)	127.1(2)
N(1)-C(2)-C(3)	112.67(19)
C(4)-C(3)-C(2)	124.37(19)
C(4)-C(3)-N(7)	117.48(19)
C(2)-C(3)-N(7)	118.14(19)
C(3)-C(4)-C(5)	117.4(2)
C(6)-C(5)-C(4)	121.1(2)
C(6)-C(5)-N(10)	119.05(19)
C(4)-C(5)-N(10)	119.9(2)
N(1)-C(6)-C(5)	120.98(19)

O(9)-N(7)-O(8)	123.95(19)
O(9)-N(7)-C(3)	117.70(18)
O(8)-N(7)-C(3)	118.33(19)
O(11)-N(10)-O(12)	124.3(2)
O(11)-N(10)-C(5)	117.86(18)
O(12)-N(10)-C(5)	117.9(2)
C(22)-C(13)-N(1)	109.9(2)
C(22)-C(13)-C(14)	112.4(2)
N(1)-C(13)-C(14)	111.42(18)
C(15)-C(14)-C(13)	116.6(2)
C(16)-C(15)-C(14)	116.42(19)
C(17)-C(16)-C(21)	117.9(2)
C(17)-C(16)-C(15)	120.3(2)
C(21)-C(16)-C(15)	121.8(2)
C(18)-C(17)-C(16)	121.3(2)
C(19)-C(18)-C(17)	120.2(2)
C(18)-C(19)-C(20)	119.3(2)
C(21)-C(20)-C(19)	120.5(2)
C(20)-C(21)-C(16)	120.8(2)
C(23)-C(22)-C(13)	177.3(3)

8. ^{1}H, ^{13}C NMR Spectra of Unknown Compounds

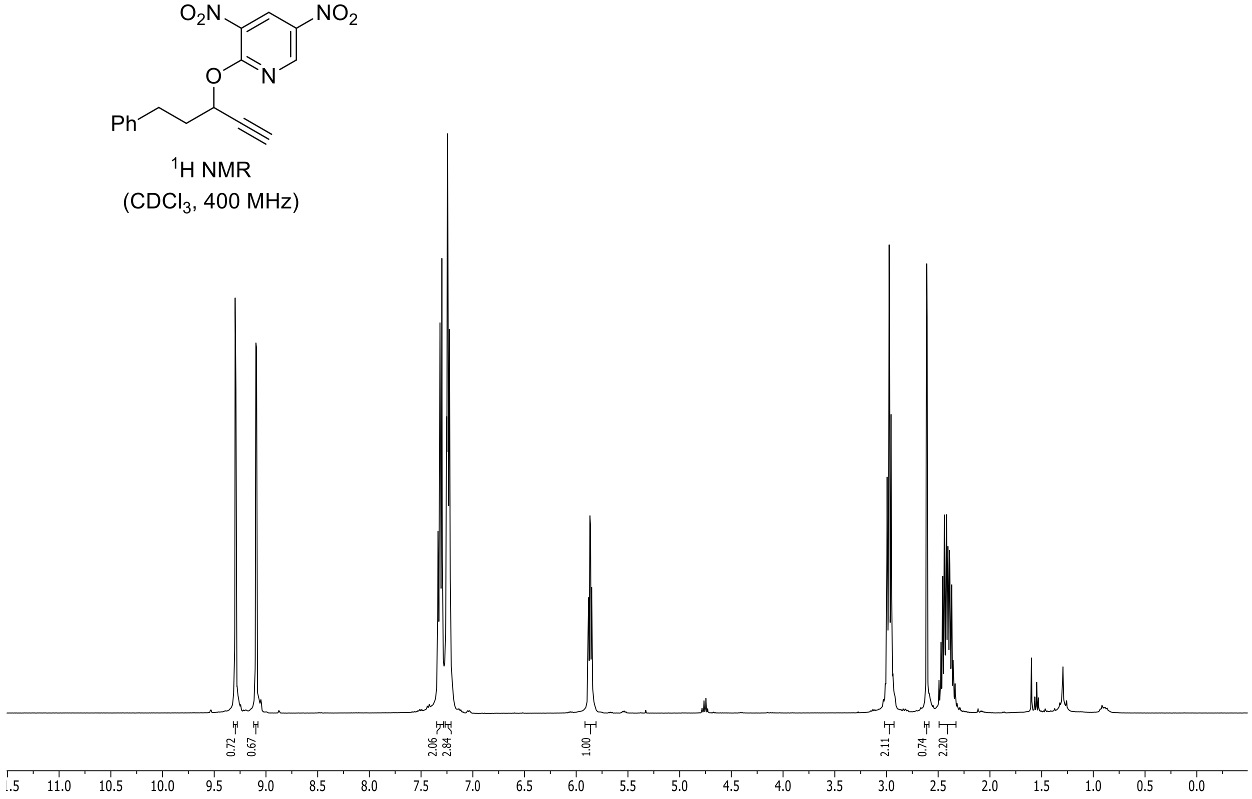

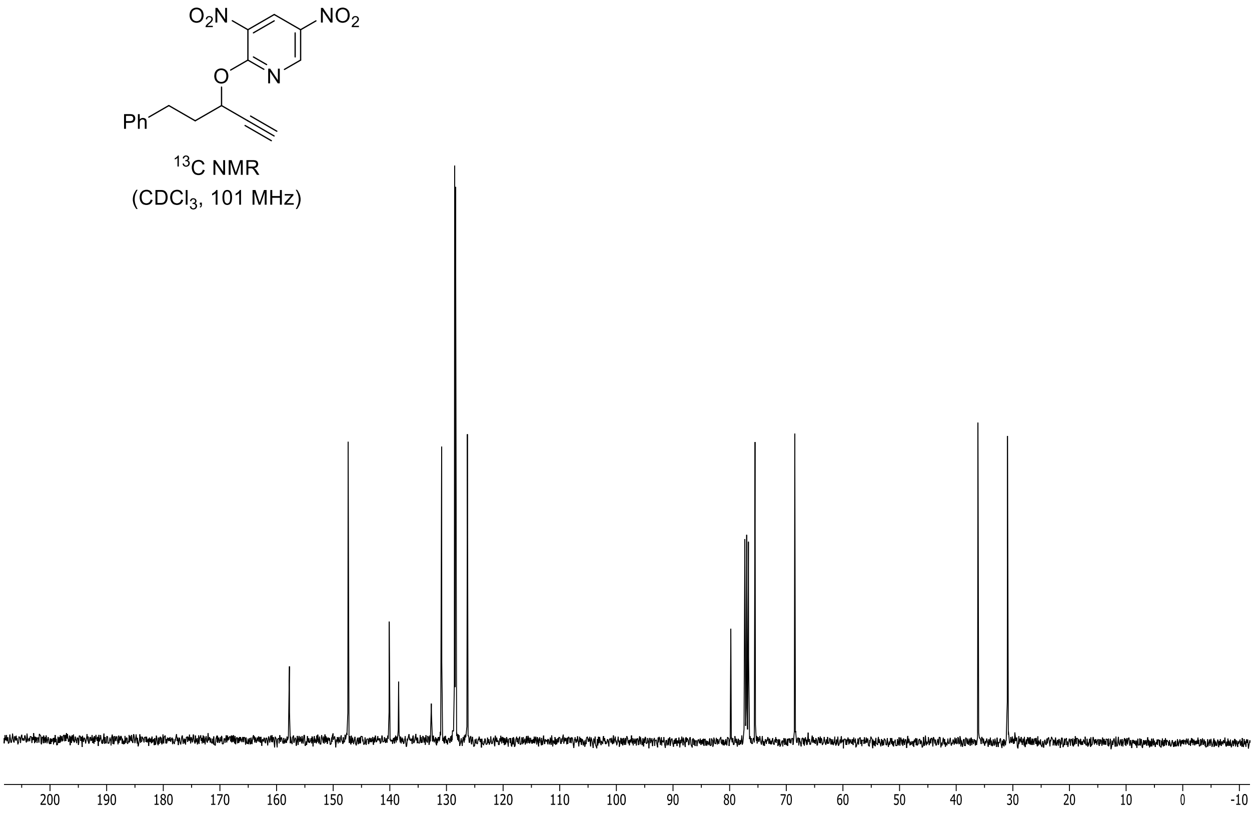

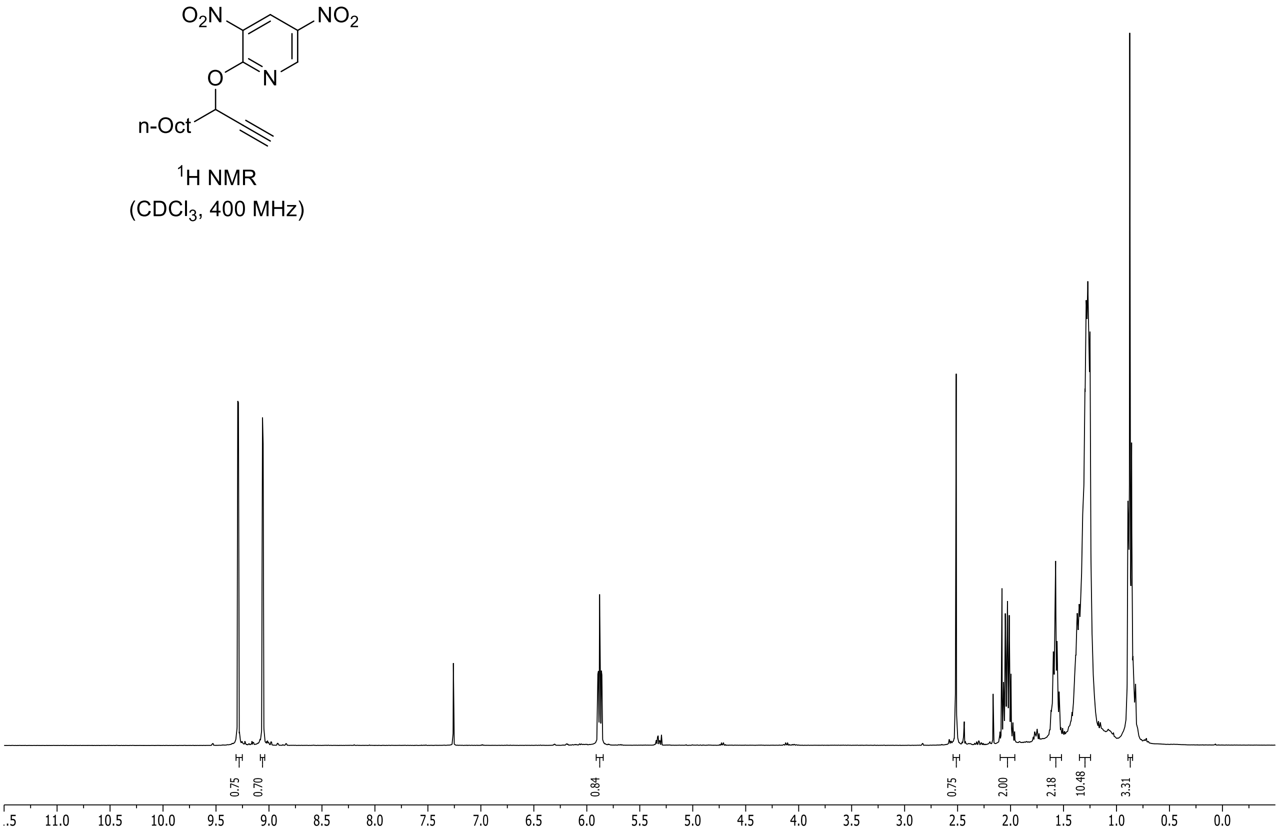
O2N
NO2
O
N
n-Oct
1H NMR
(CDCl3, 400 MHz)
0.75
0.70
0.84
0.75
2.00
2.18
10.48
3.31
11.0 10.5 10.0 9.5 9.0 8.5 8.0 7.5 7.0 6.5 6.0 5.5 5.0 4.5 4.0 3.5 3.0 2.5 2.0 1.5 1.0 0.5 0.0

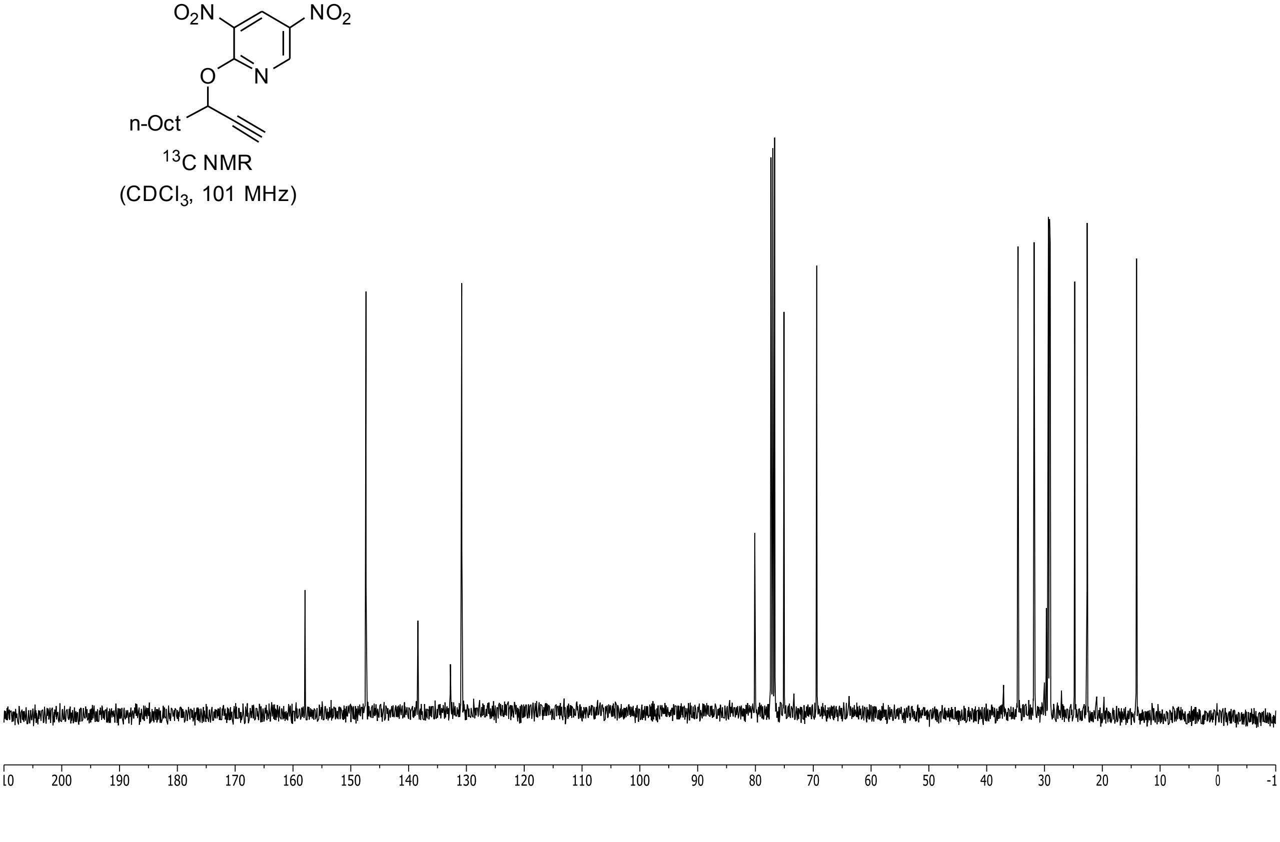
O2N
NO2
O
N
n-Oct
13C NMR
(CDCl3, 101 MHz)
200 190 180 170 160 150 140 130 120 110 100 90 80 70 60 50 40 30 20 10 0

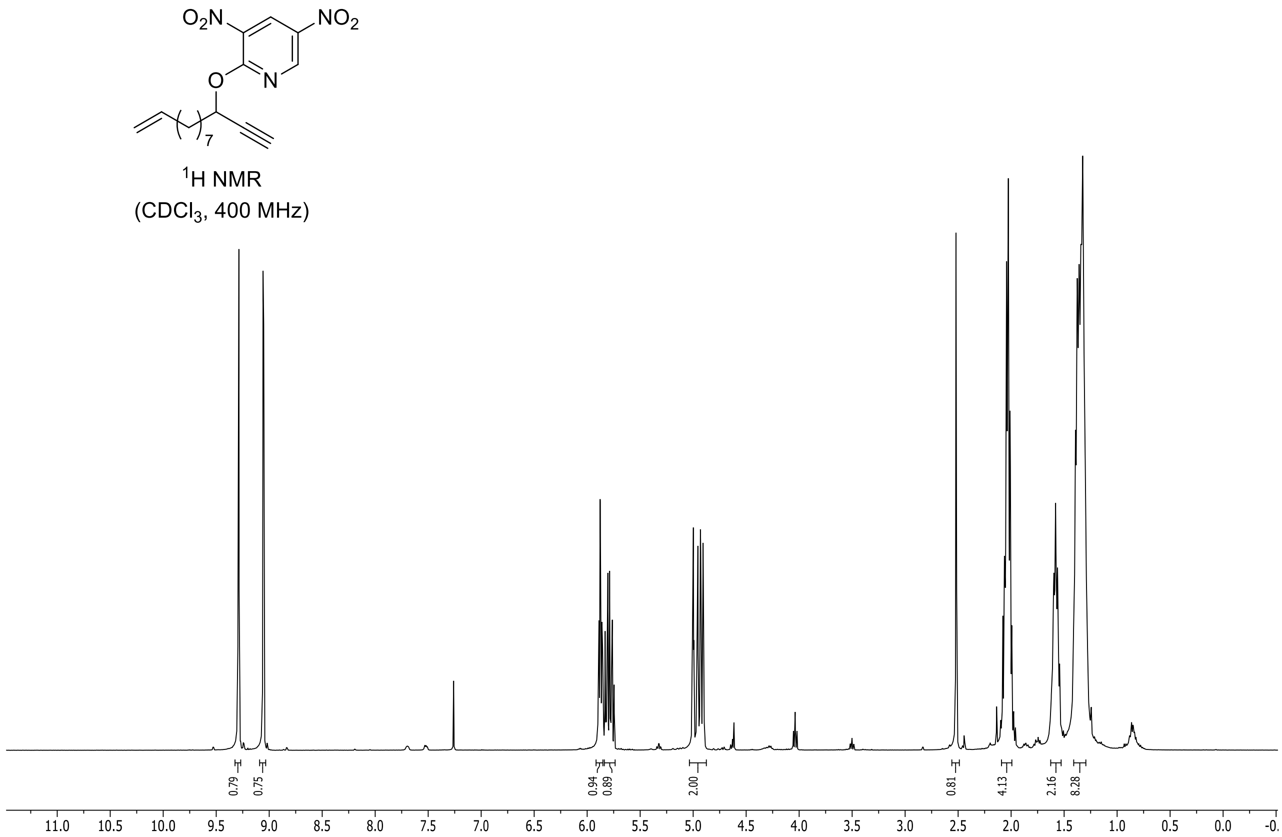

O2N
NO2
O
N
7
1H NMR
(CDCl3, 400 MHz)
0.79
0.75
0.94
0.89
2.00
0.81
4.13
2.16
8.28
11.0 10.5 10.0 9.5 9.0 8.5 8.0 7.5 7.0 6.5 6.0 5.5 5.0 4.5 4.0 3.5 3.0 2.5 2.0 1.5 1.0 0.5 0.0

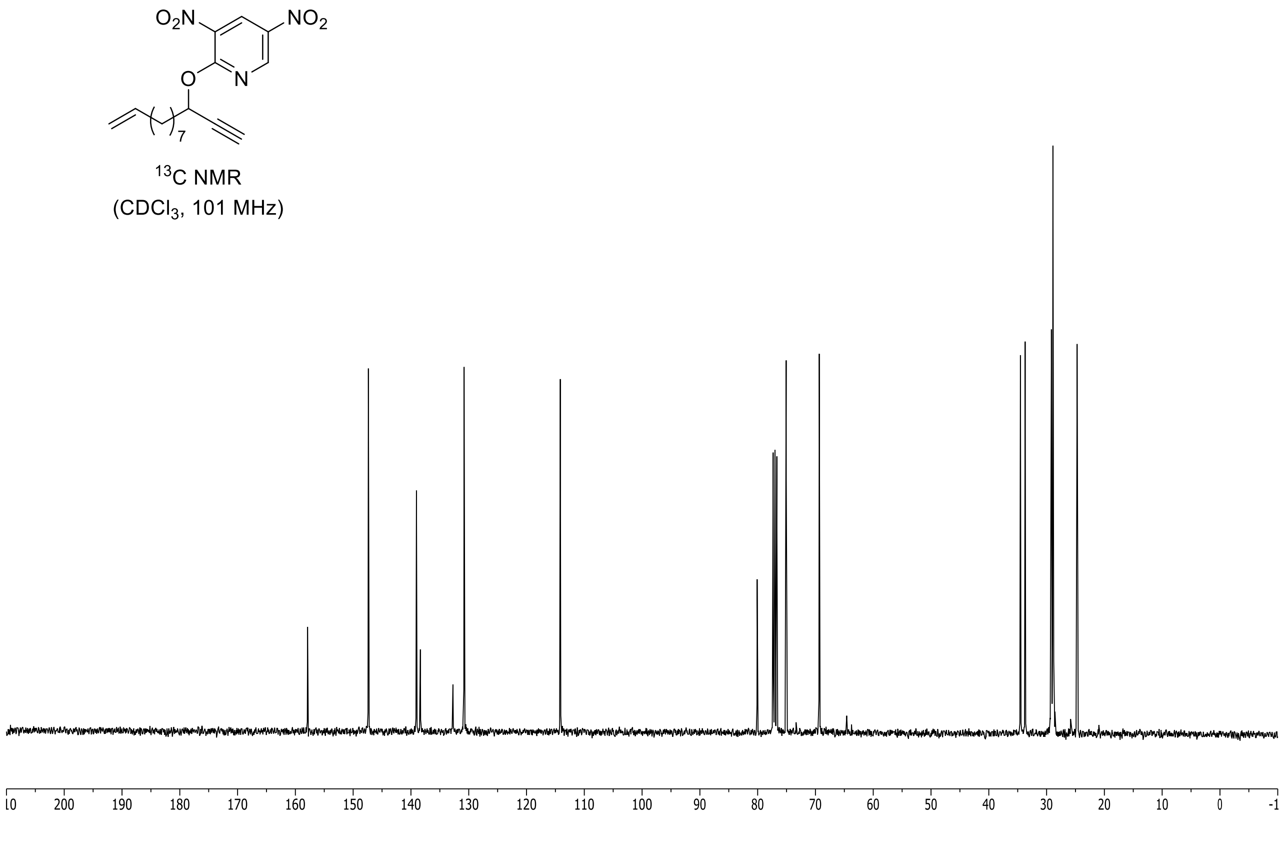

O2N
NO2
O
N
7
13C NMR
(CDCl3, 101 MHz)
200 190 180 170 160 150 140 130 120 110 100 90 80 70 60 50 40 30 20 10 0

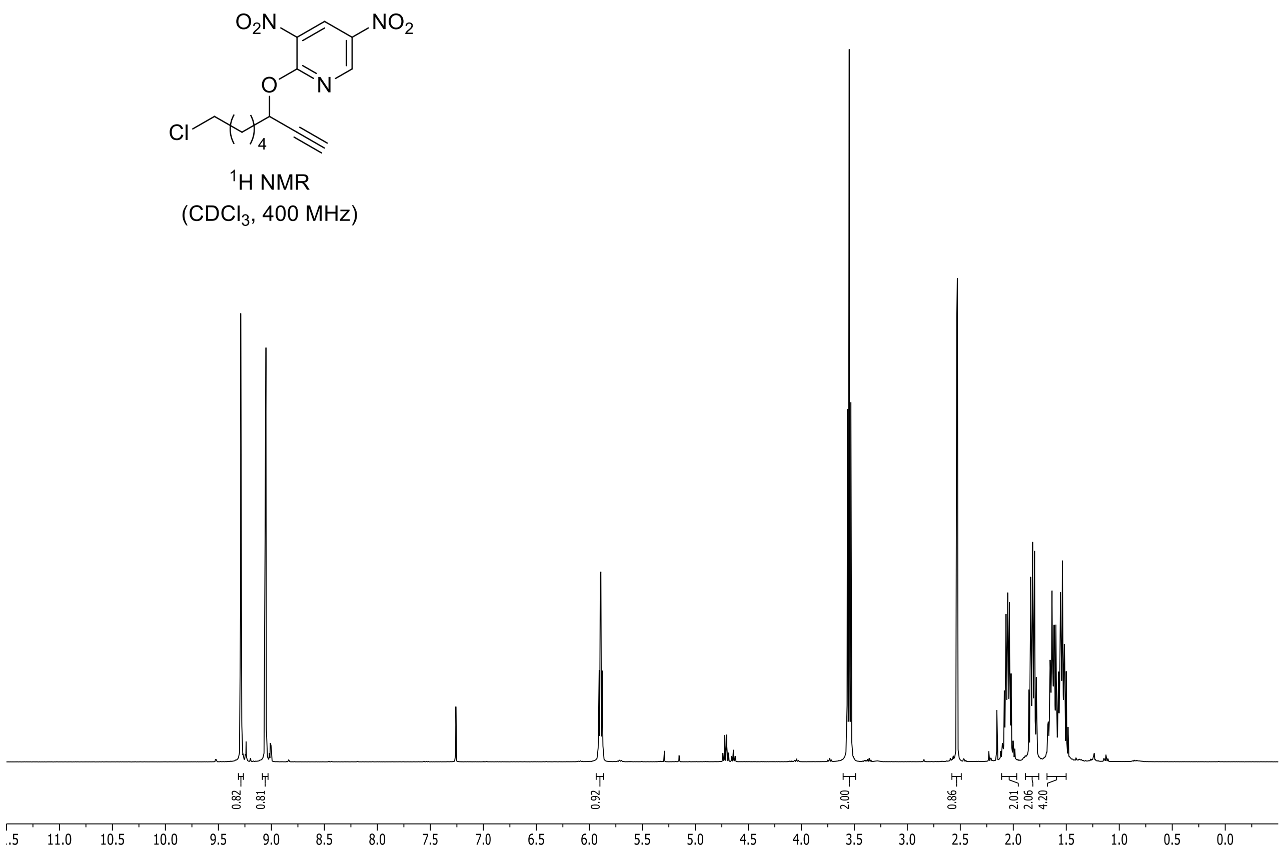

O_2N
NO_2
N
O
Cl
4
^1H NMR
($CDCl_3$, 400 MHz)
0.82
0.81
0.92
2.00
0.86
2.01
2.06
4.20

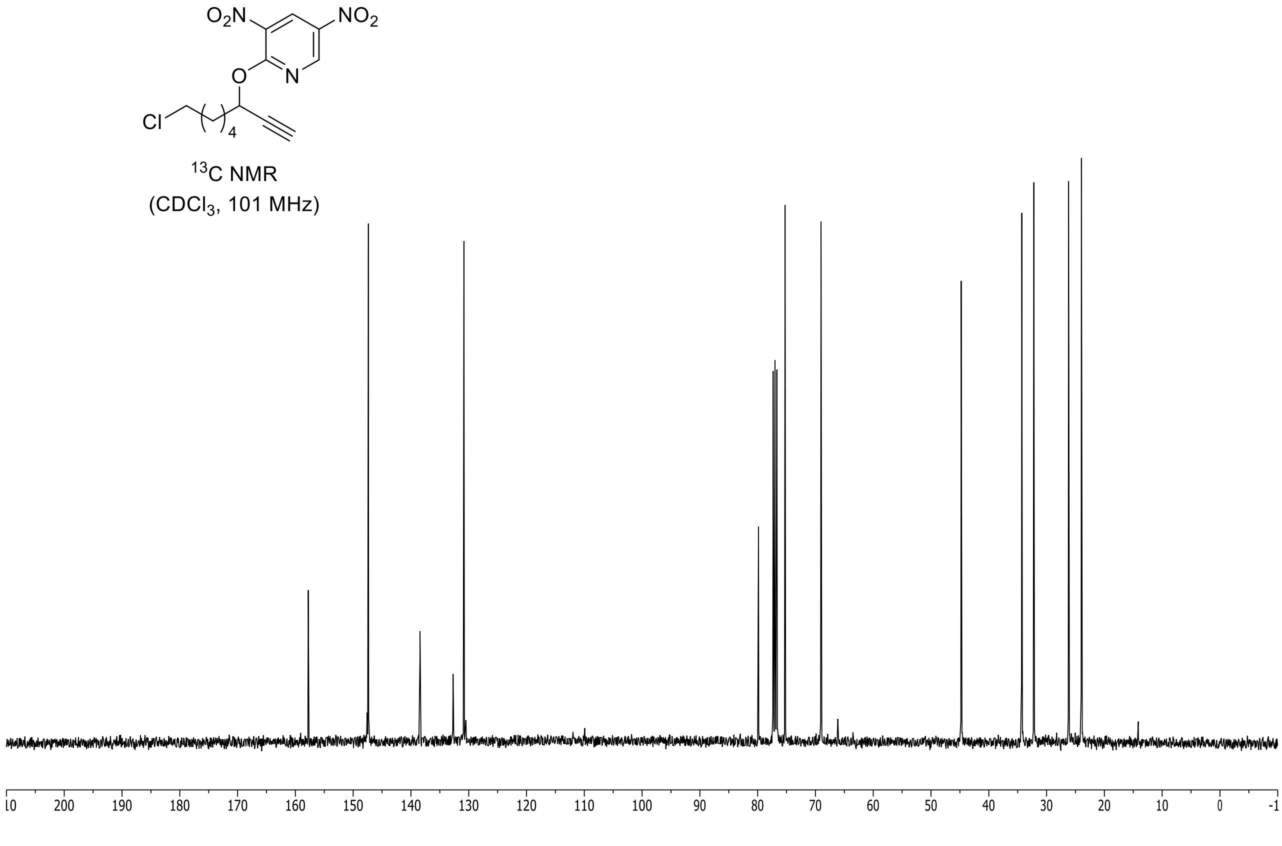

O_2N
NO_2
N
O
Cl
4
^{13}C NMR
($CDCl_3$, 101 MHz)

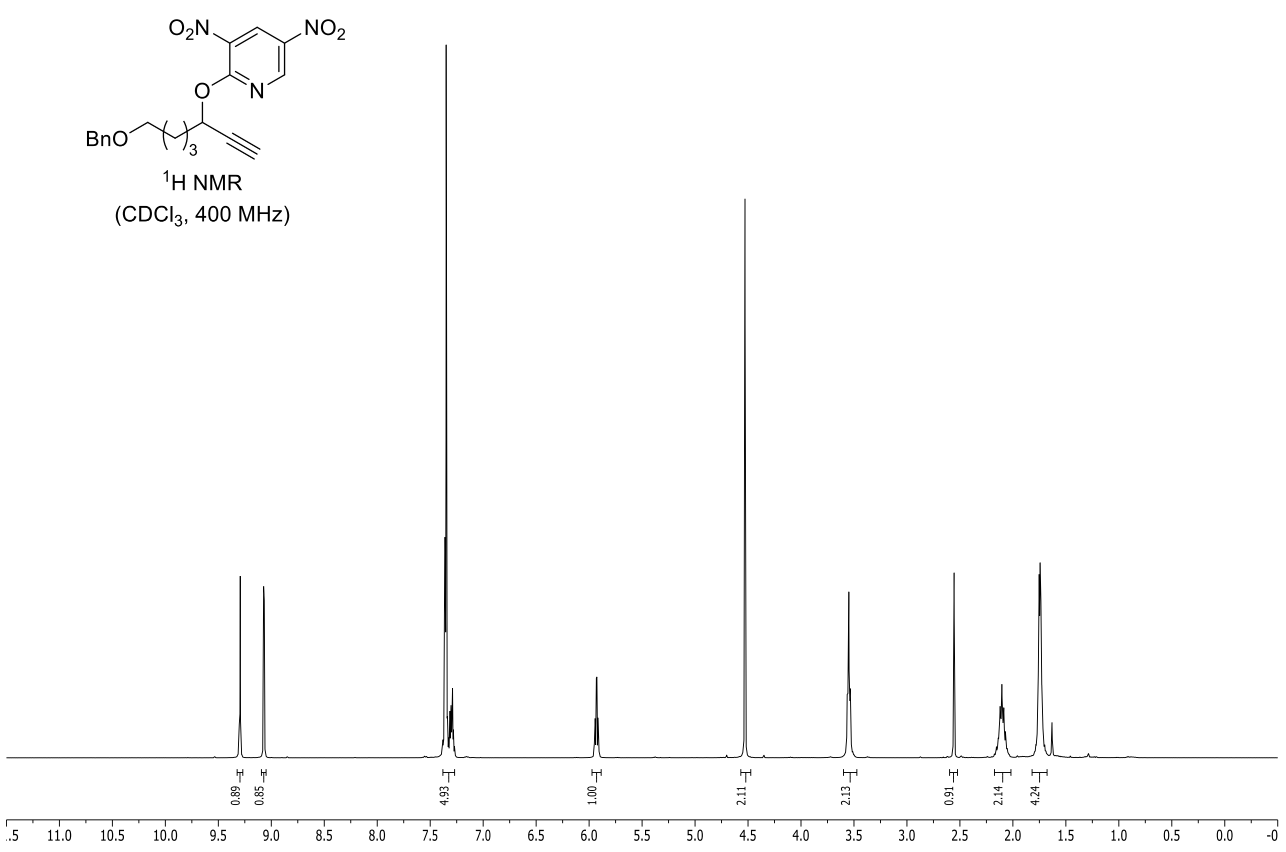
O2N
NO2
O
N
BnO
3
1H NMR
(CDCl3, 400 MHz)
0.89
0.85
4.93
1.00
2.11
2.13
0.91
2.14
4.24

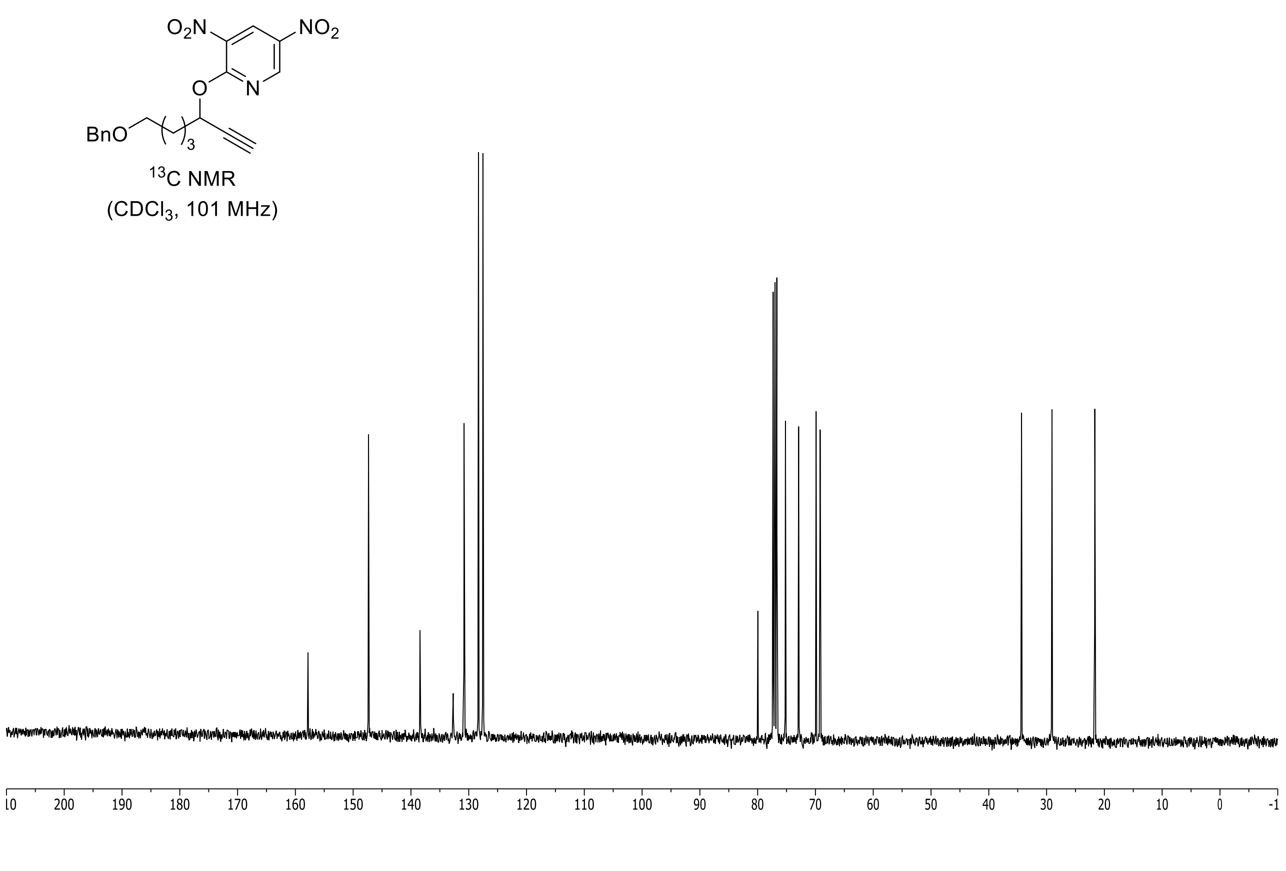
O2N
NO2
O
N
BnO
3
13C NMR
(CDCl3, 101 MHz)

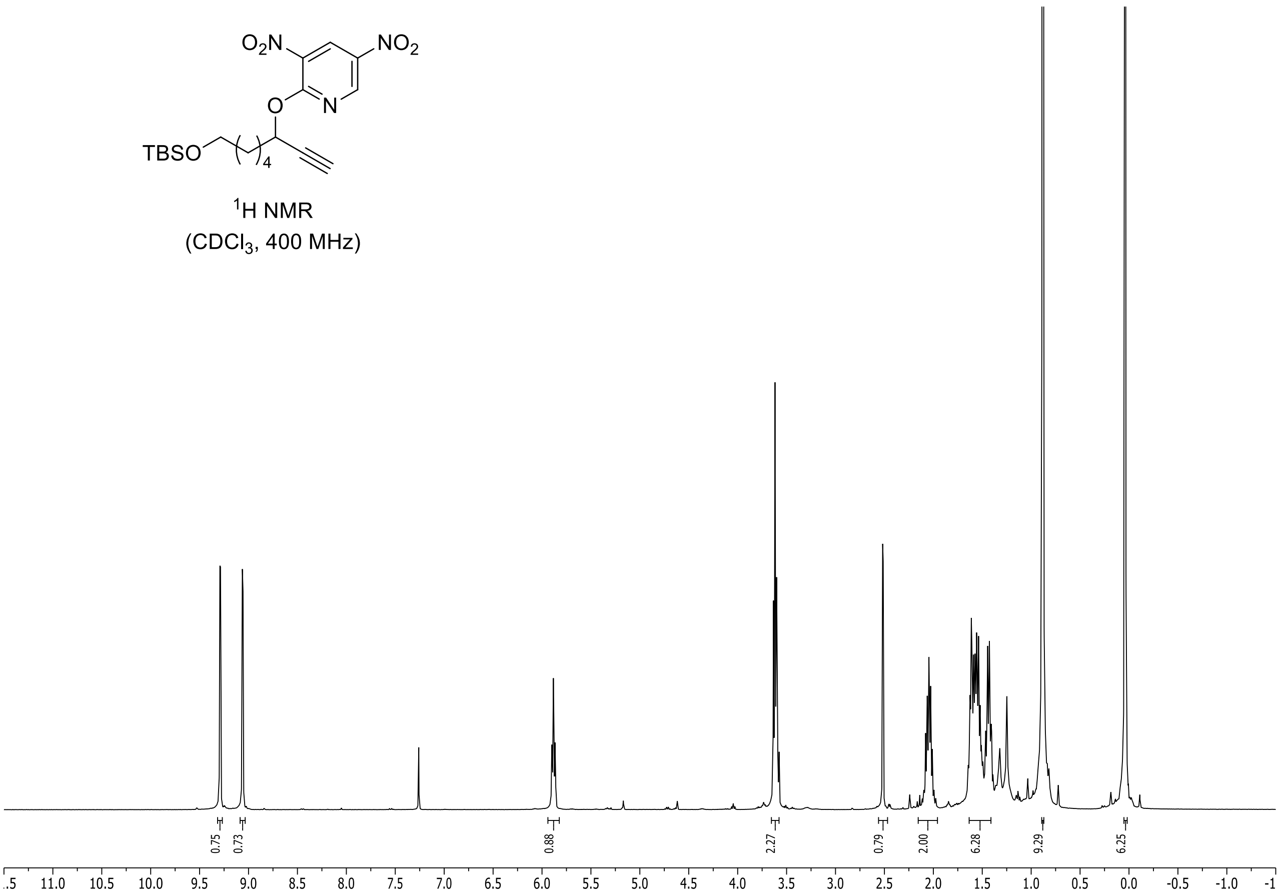
O2N
NO2
O
N
TBSO
4
1H NMR
(CDCl3, 400 MHz)
0.75
0.73
0.88
2.27
0.79
2.00
6.28
9.29
6.25
11.0 10.5 10.0 9.5 9.0 8.5 8.0 7.5 7.0 6.5 6.0 5.5 5.0 4.5 4.0 3.5 3.0 2.5 2.0 1.5 1.0 0.5 0.0 -0.5 -1.0

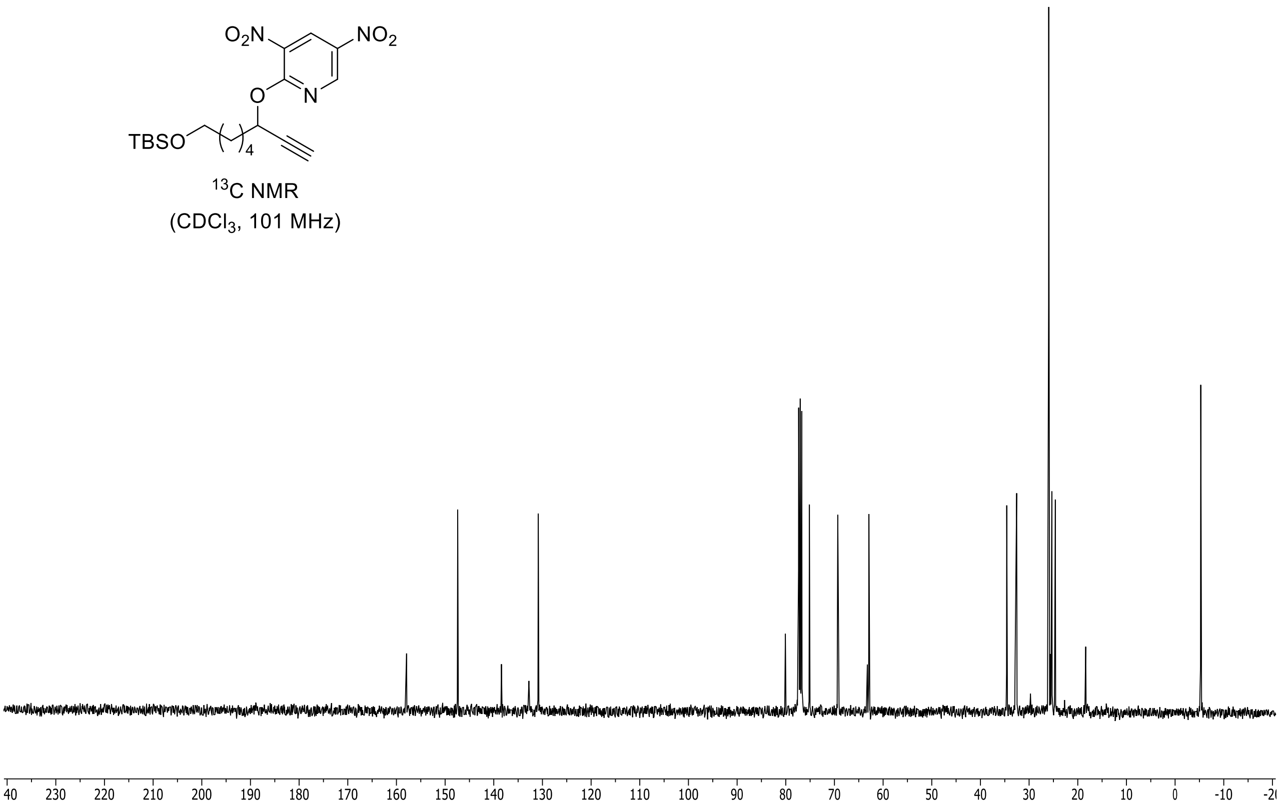
O2N
NO2
O
N
TBSO
4
13C NMR
(CDCl3, 101 MHz)
230 220 210 200 190 180 170 160 150 140 130 120 110 100 90 80 70 60 50 40 30 20 10 0 -10

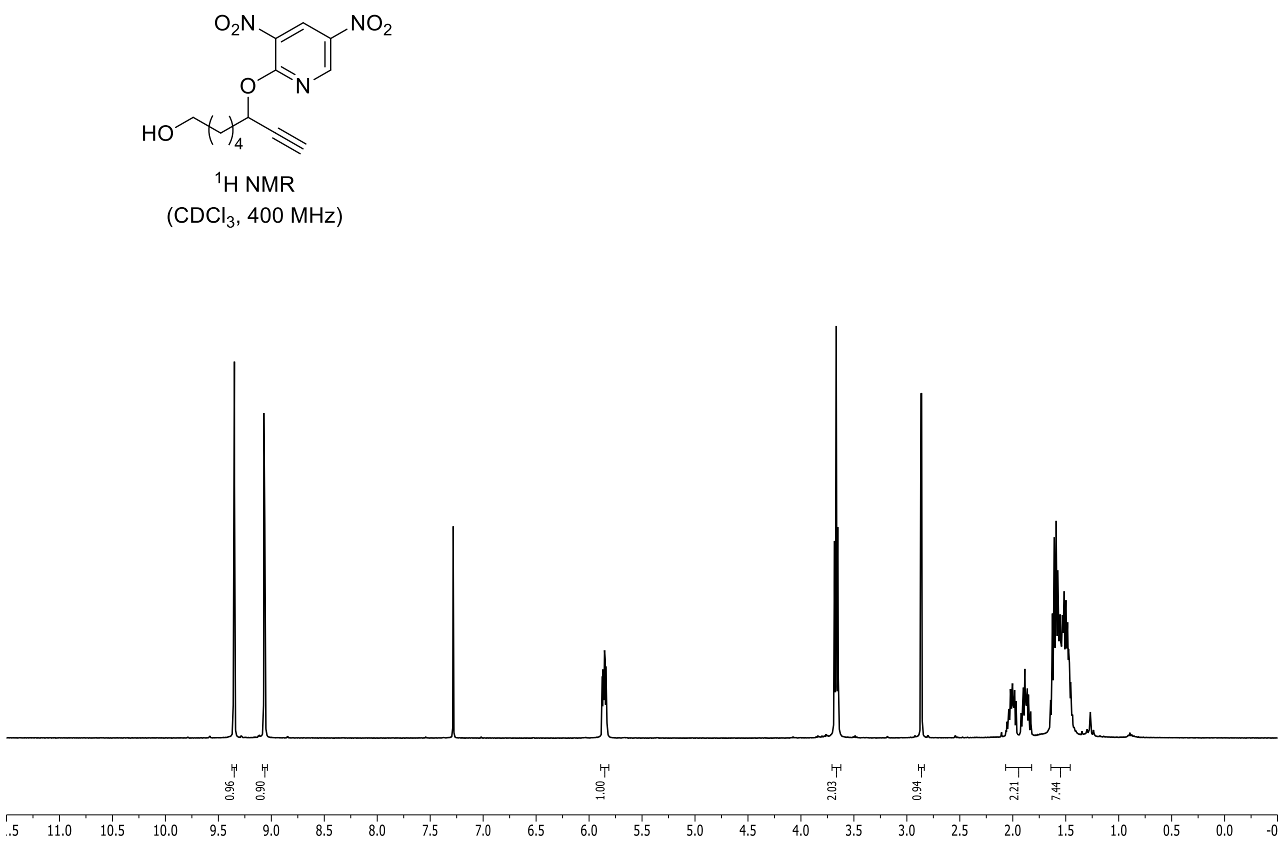
O2N
NO2
N
O
HO
4
1H NMR
(CDCl3, 400 MHz)
0.96
0.90
1.00
2.03
0.94
2.21
7.44

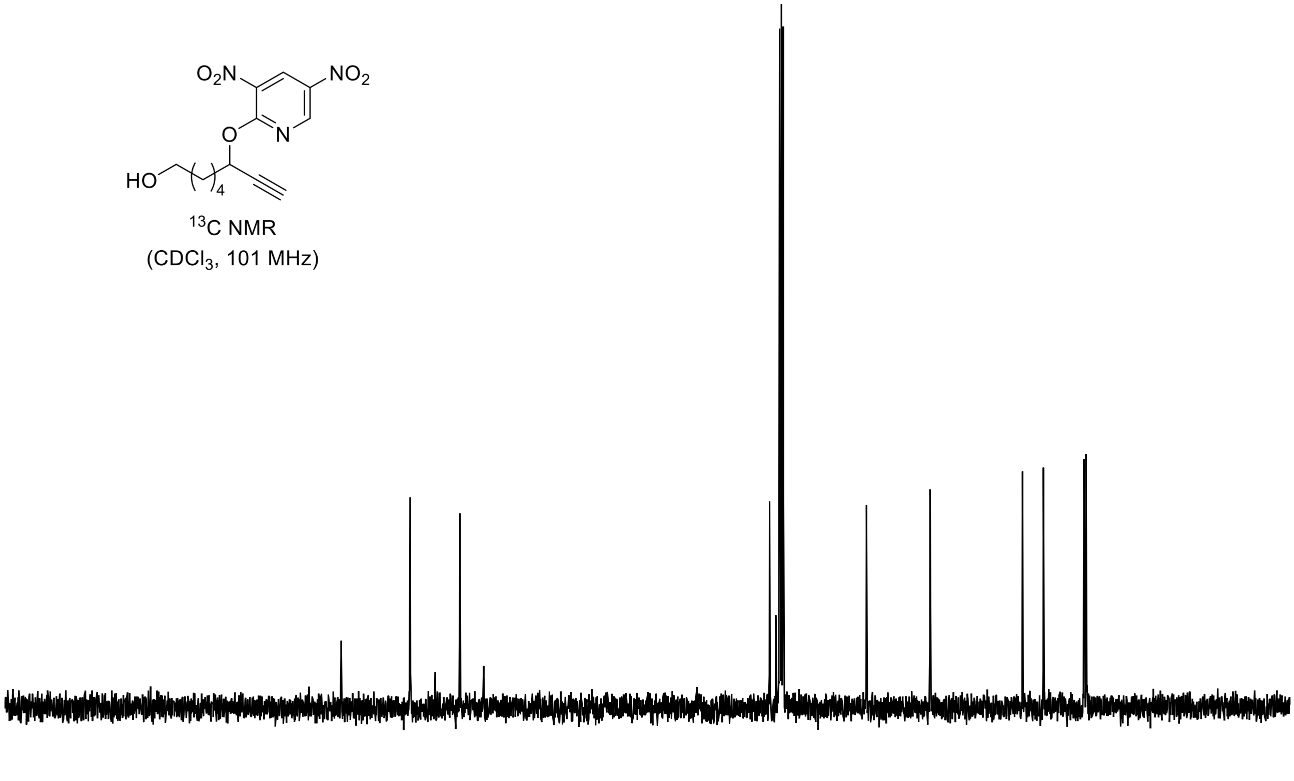
O2N
NO2
N
O
HO
4
13C NMR
(CDCl3, 101 MHz)

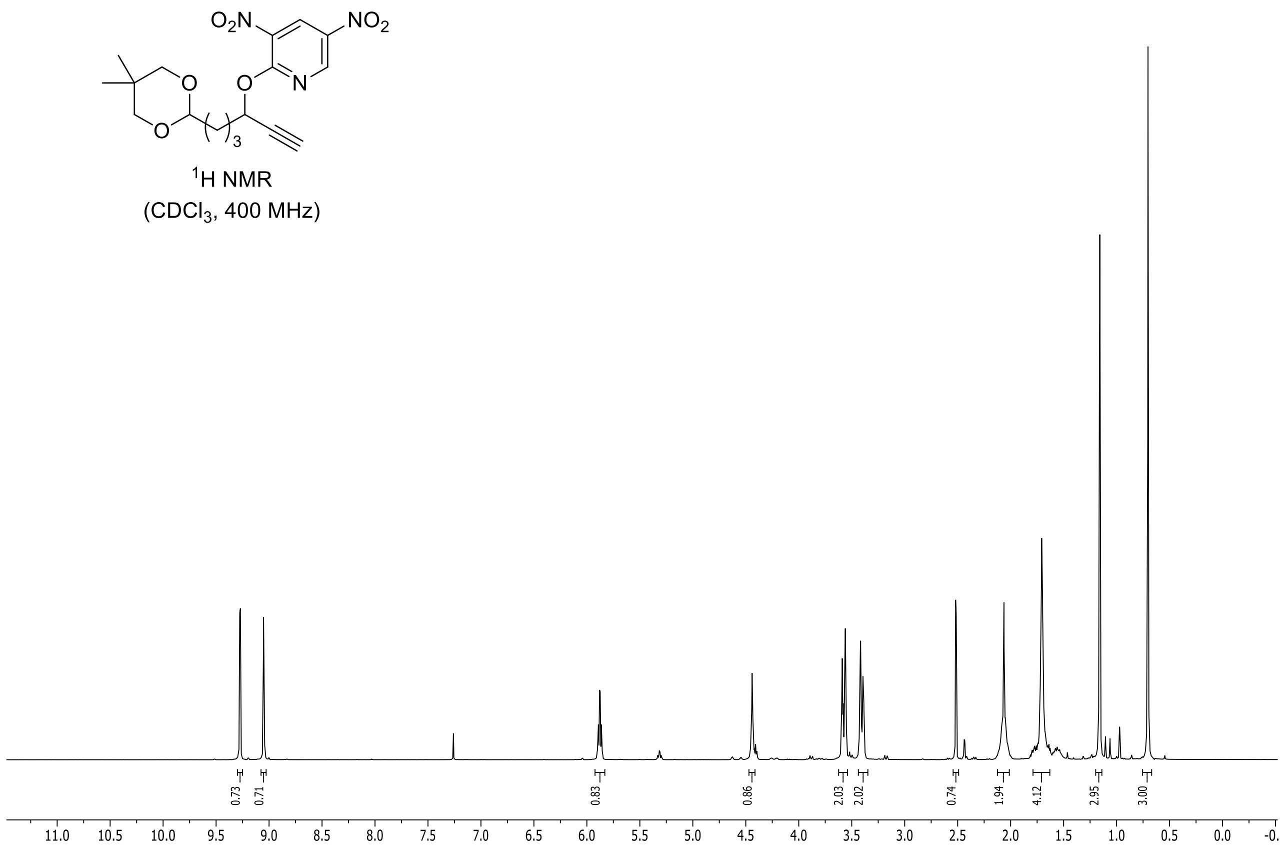
O2N
NO2
O
N
O
O
3
1H NMR
(CDCl3, 400 MHz)
0.73 0.71 0.83 0.86 2.03 2.02 0.74 1.94 4.12 2.95 3.00
11.0 10.5 10.0 9.5 9.0 8.5 8.0 7.5 7.0 6.5 6.0 5.5 5.0 4.5 4.0 3.5 3.0 2.5 2.0 1.5 1.0 0.5 0.0

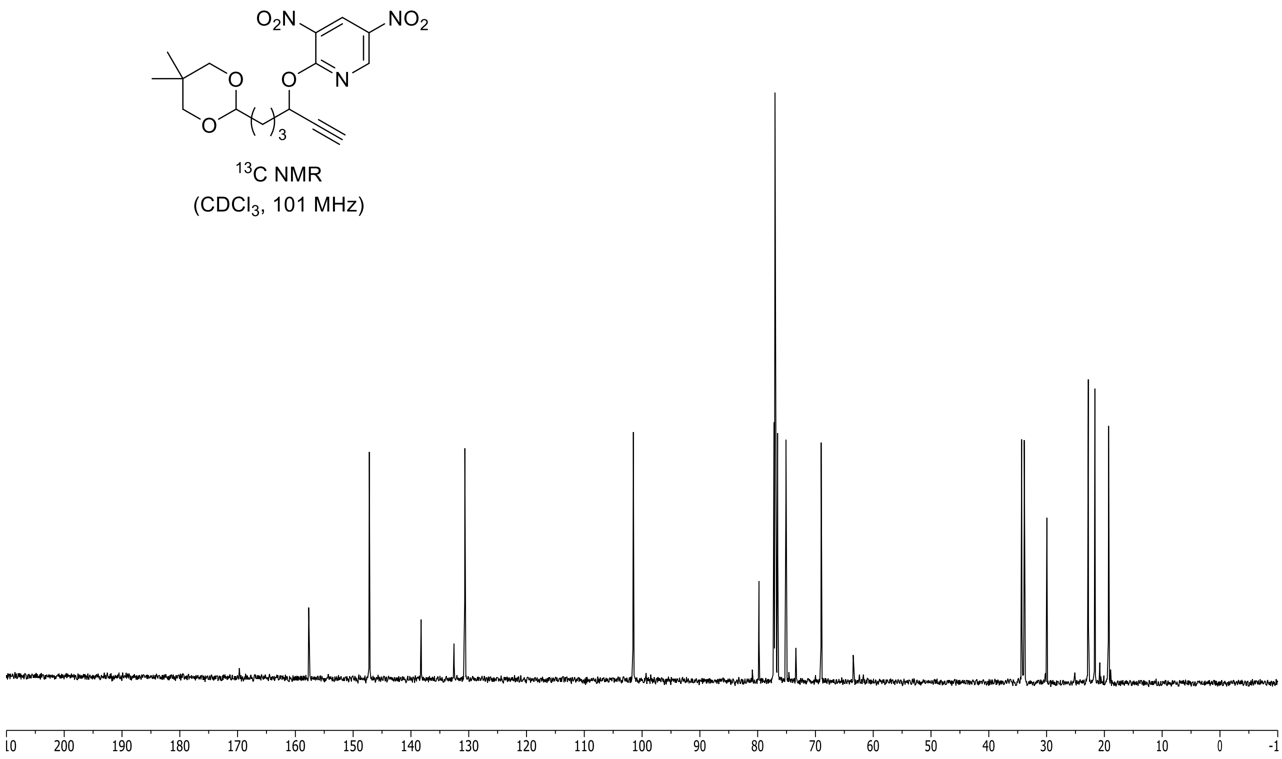
O2N
NO2
O
N
O
O
3
13C NMR
(CDCl3, 101 MHz)
200 190 180 170 160 150 140 130 120 110 100 90 80 70 60 50 40 30 20 10 0

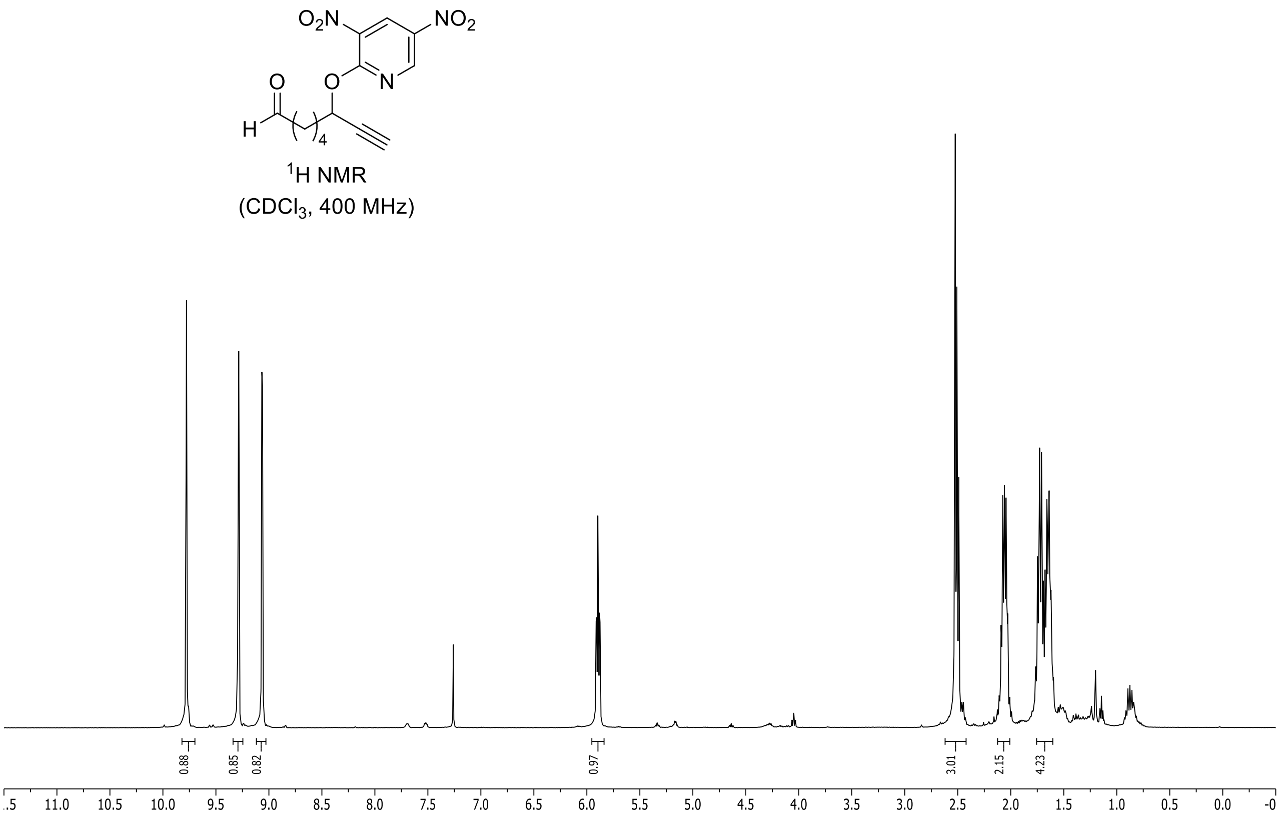

O2N
NO2
N
O
O
H
4
1H NMR
(CDCl3, 400 MHz)

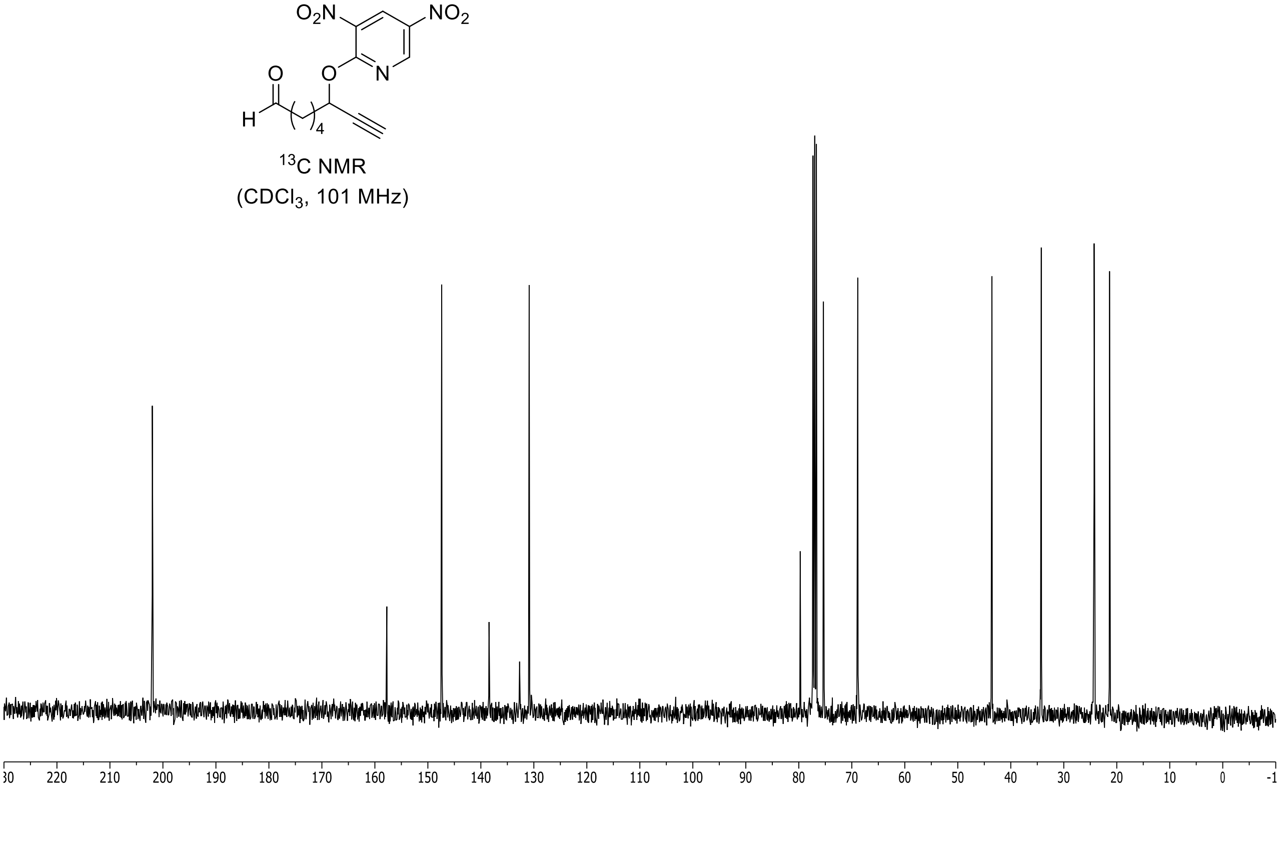

O2N
NO2
N
O
O
H
4
13C NMR
(CDCl3, 101 MHz)

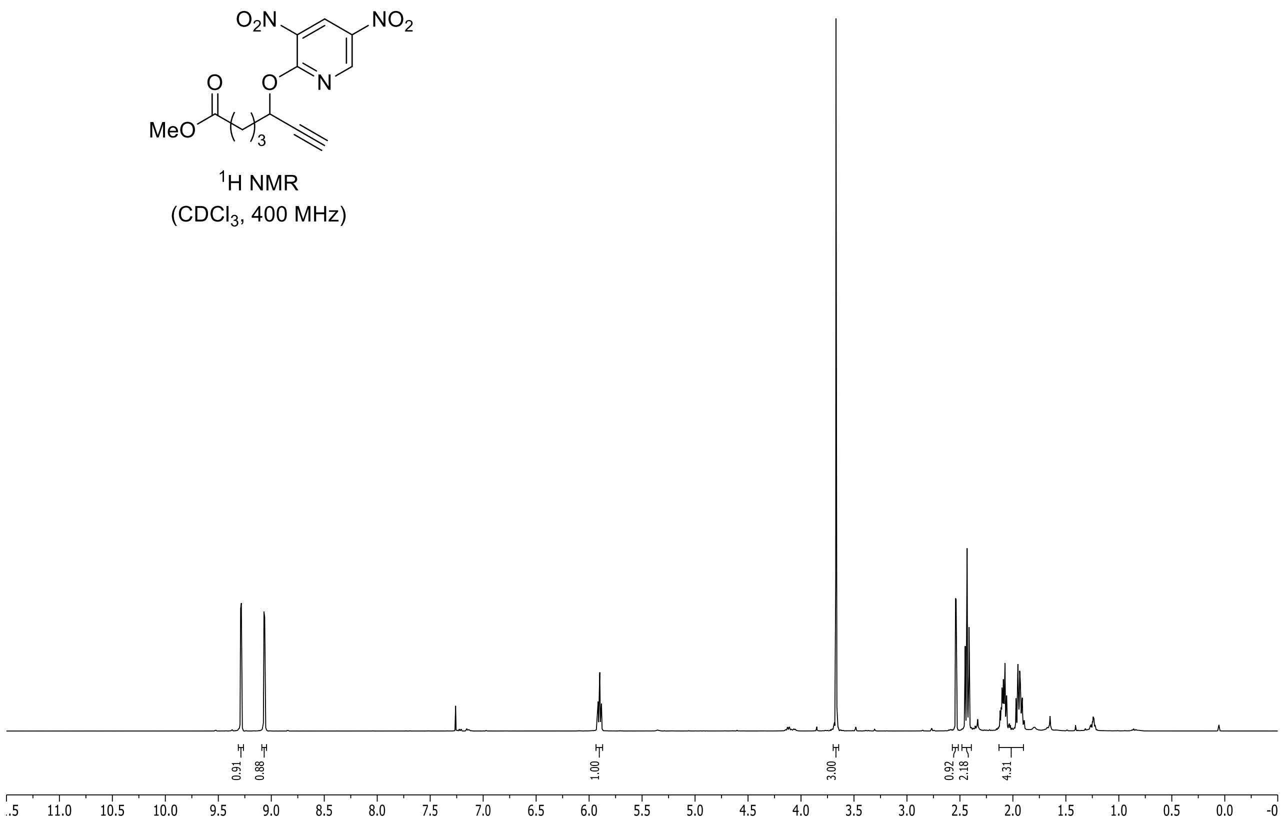
O2N
NO2
O
N
O
MeO
3
1H NMR
(CDCl3, 400 MHz)
0.91
0.88
1.00
3.00
0.92
2.18
4.31
11.0 10.5 10.0 9.5 9.0 8.5 8.0 7.5 7.0 6.5 6.0 5.5 5.0 4.5 4.0 3.5 3.0 2.5 2.0 1.5 1.0 0.5 0.0

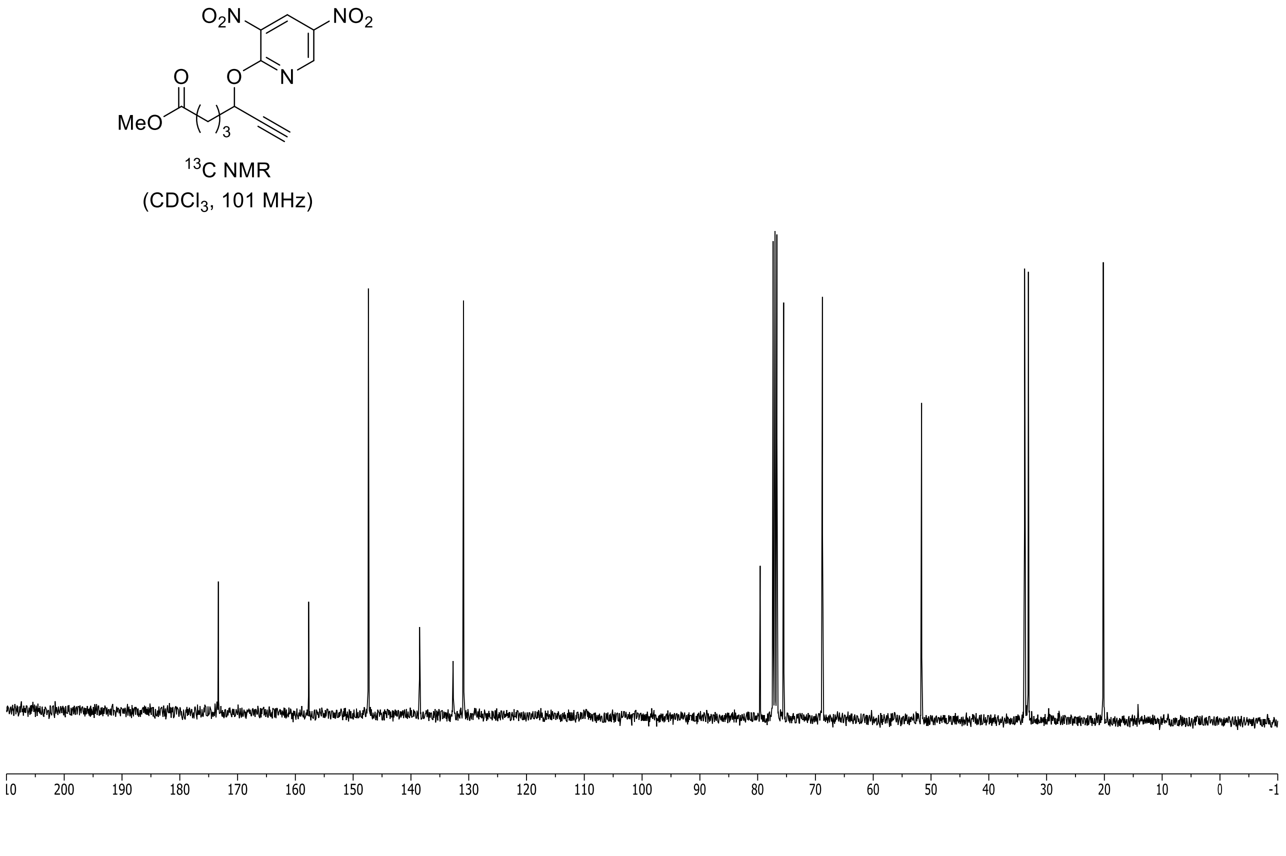
O2N
NO2
O
N
O
MeO
3
13C NMR
(CDCl3, 101 MHz)
200 190 180 170 160 150 140 130 120 110 100 90 80 70 60 50 40 30 20 10 0

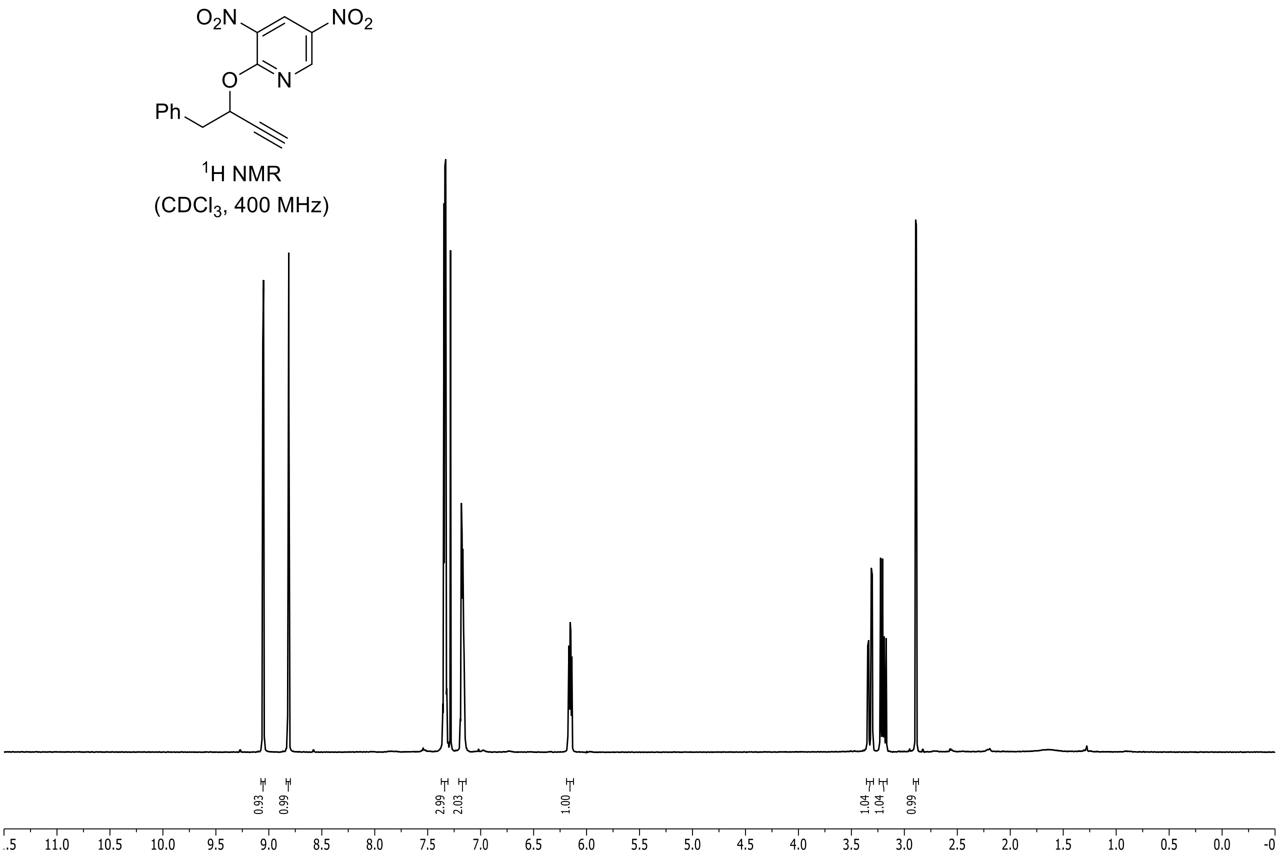
O2N
NO2
O
N
Ph
1H NMR
(CDCl3, 400 MHz)
0.93
0.99
2.99
2.03
1.00
1.04
1.04
0.99
11.0 10.5 10.0 9.5 9.0 8.5 8.0 7.5 7.0 6.5 6.0 5.5 5.0 4.5 4.0 3.5 3.0 2.5 2.0 1.5 1.0 0.5 0.0

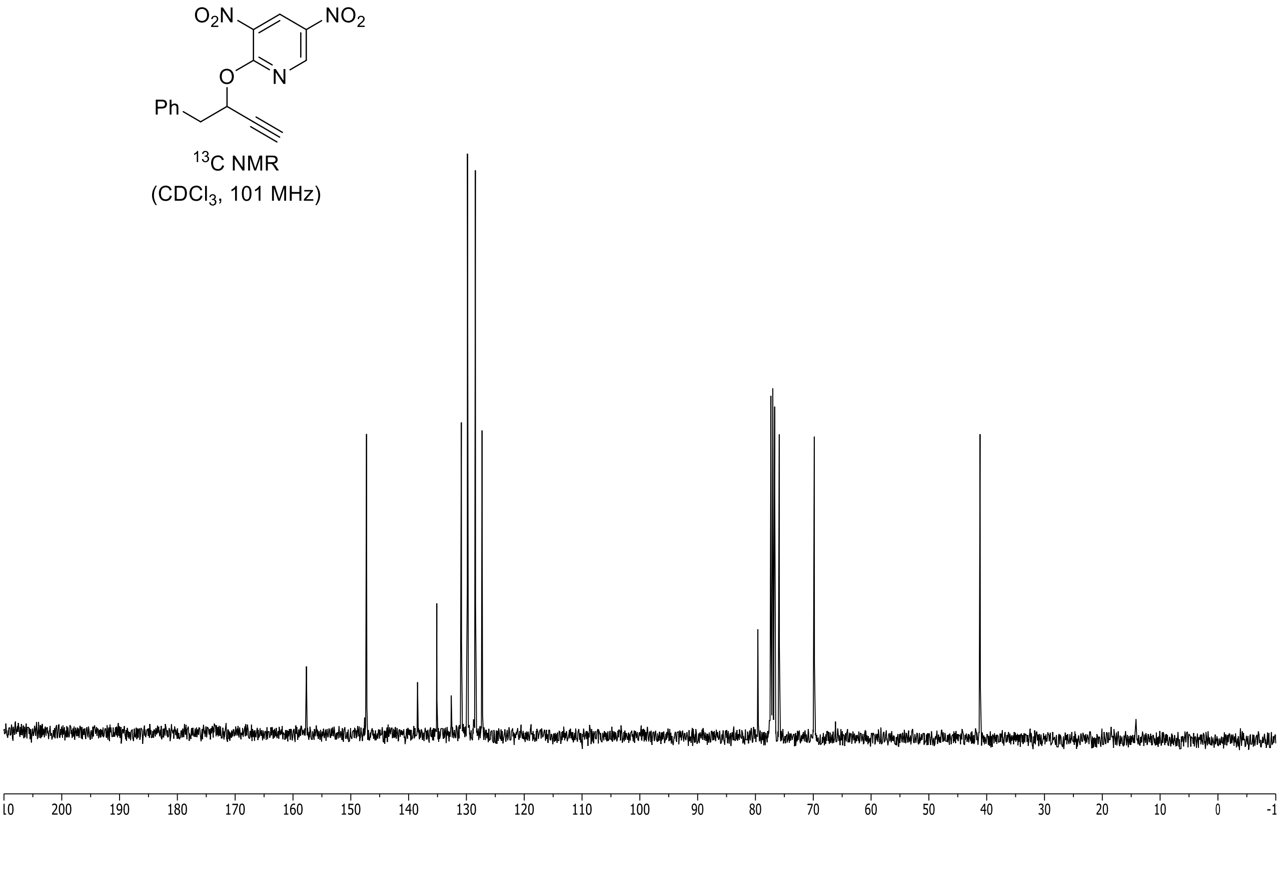
O2N
NO2
O
N
Ph
13C NMR
(CDCl3, 101 MHz)
200 190 180 170 160 150 140 130 120 110 100 90 80 70 60 50 40 30 20 10 0

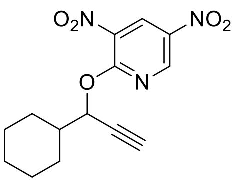

^{1}H NMR
($CDCl_3$, 400 MHz)

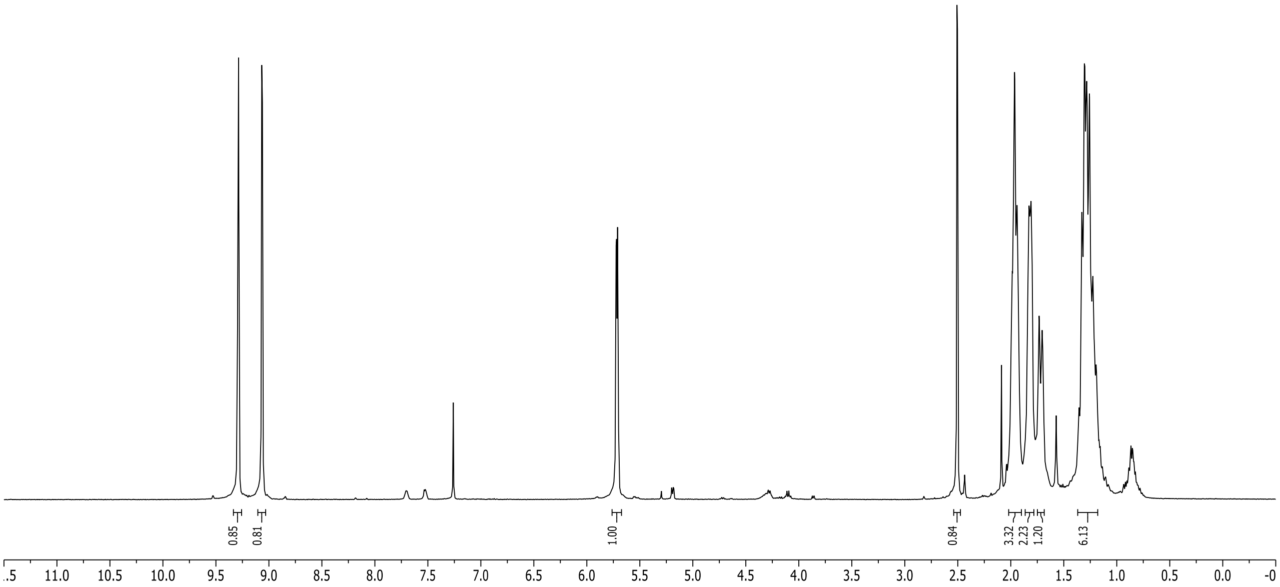

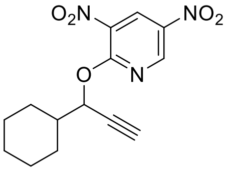

^{13}C NMR
($CDCl_3$, 101 MHz)

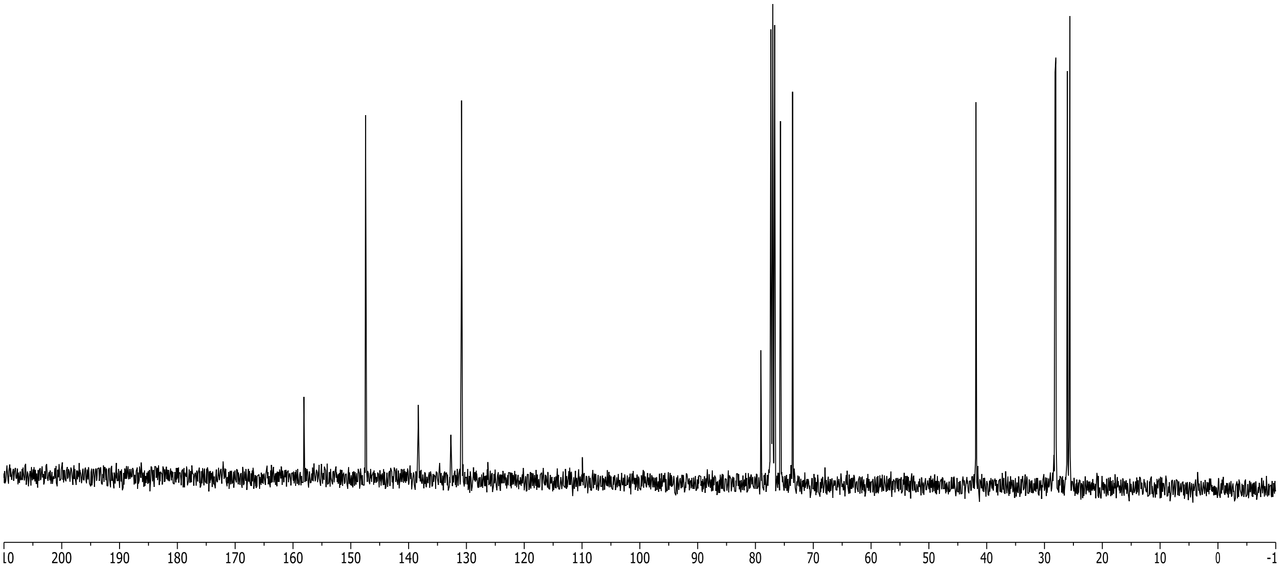

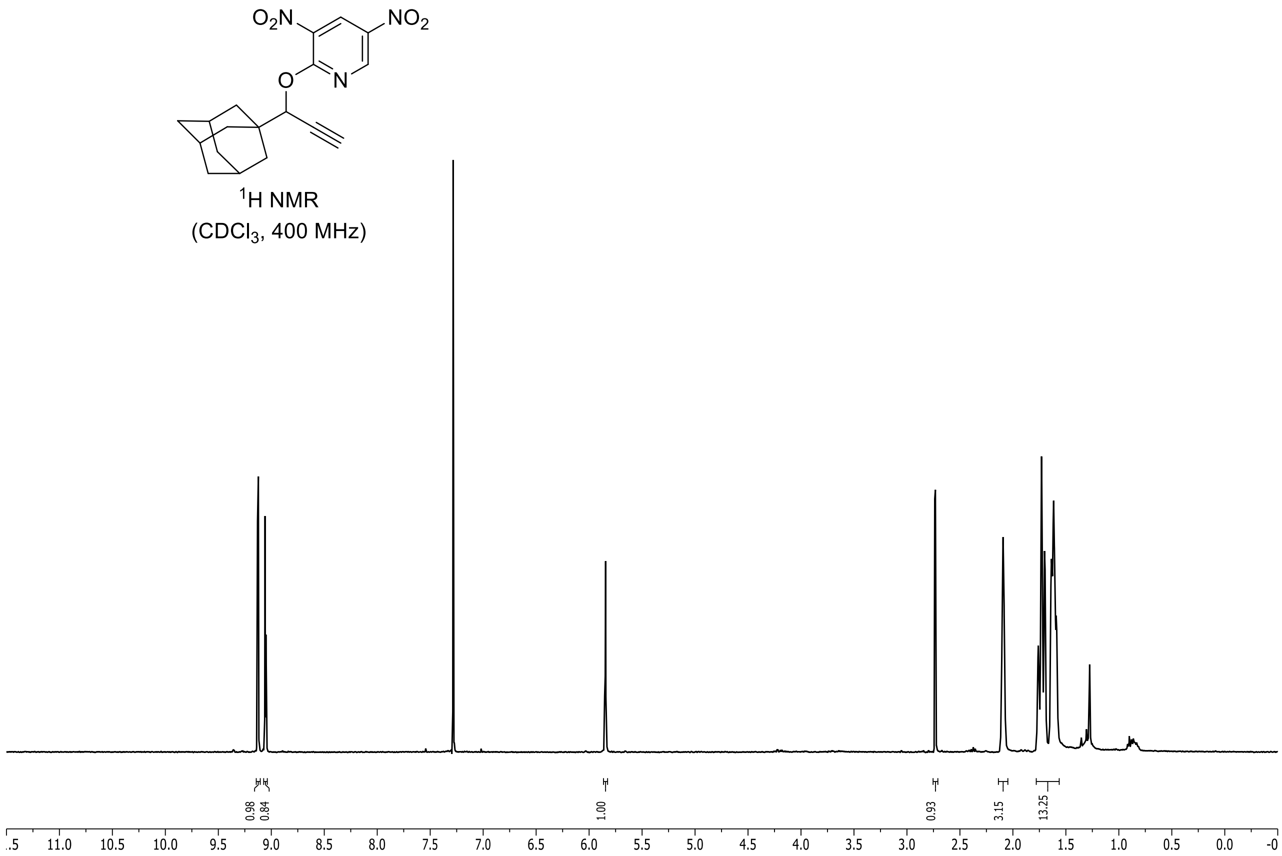

O_2N
NO_2
O
N
^1H NMR
($CDCl_3$, 400 MHz)

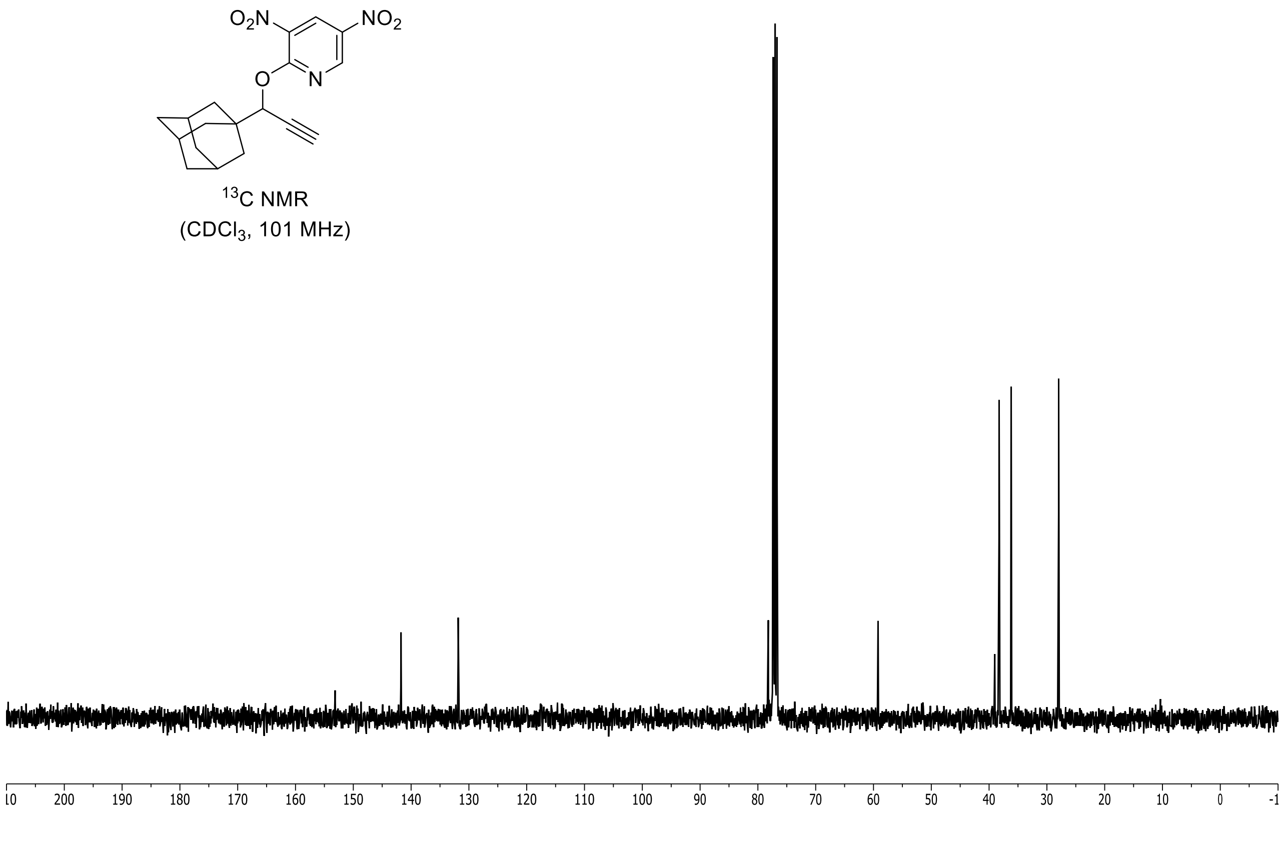

O_2N
NO_2
O
N
^{13}C NMR
($CDCl_3$, 101 MHz)

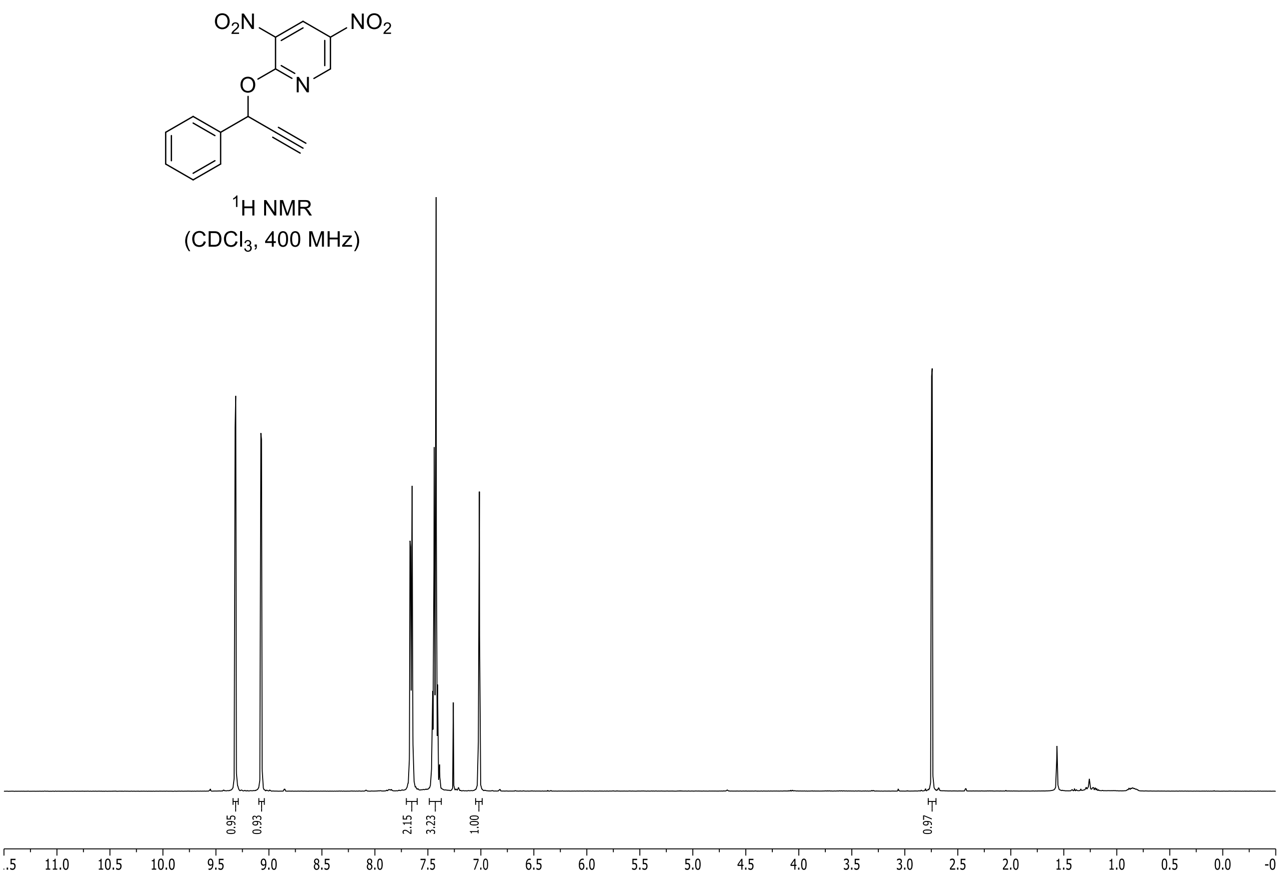
O2N
NO2
O
N
1H NMR
(CDCl3, 400 MHz)
0.95
0.93
2.15
3.23
1.00
0.97
11.0 10.5 10.0 9.5 9.0 8.5 8.0 7.5 7.0 6.5 6.0 5.5 5.0 4.5 4.0 3.5 3.0 2.5 2.0 1.5 1.0 0.5 0.0

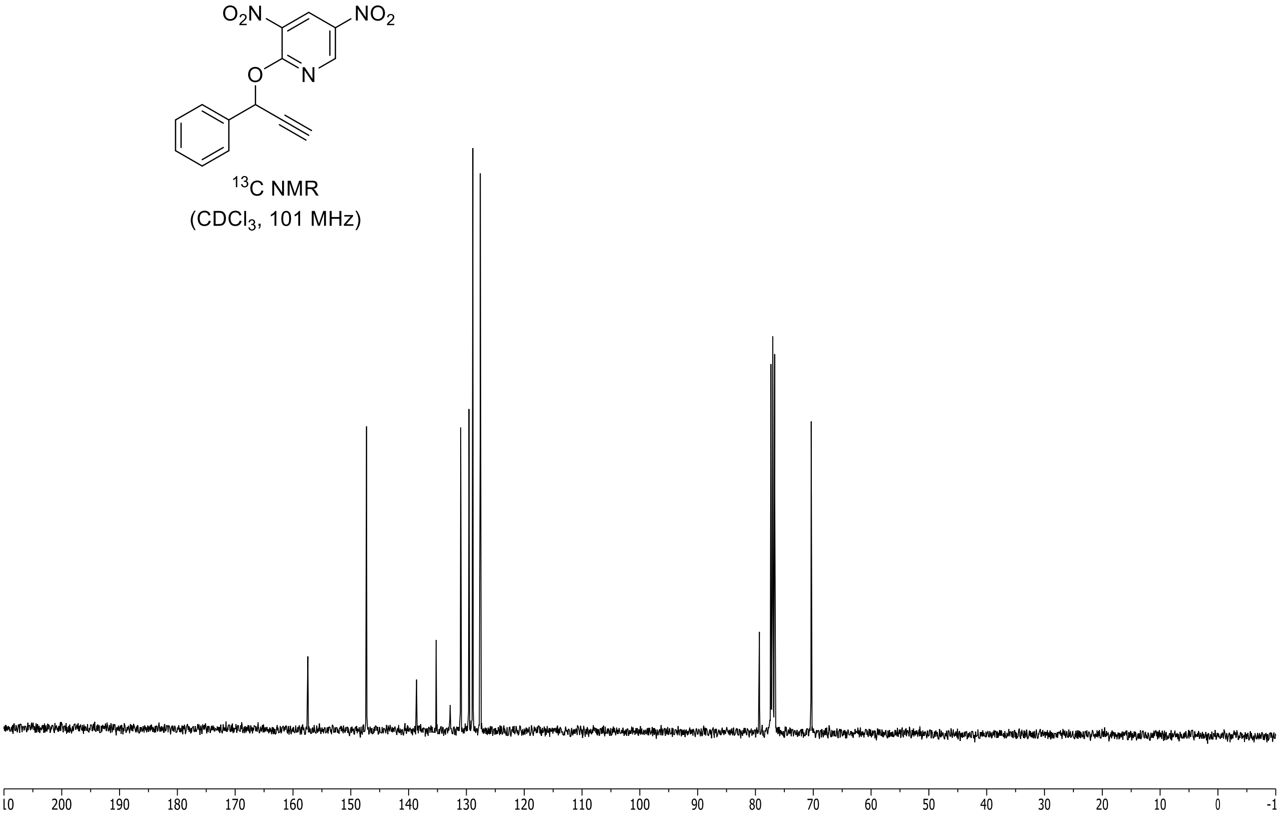
O2N
NO2
O
N
13C NMR
(CDCl3, 101 MHz)
200 190 180 170 160 150 140 130 120 110 100 90 80 70 60 50 40 30 20 10 0

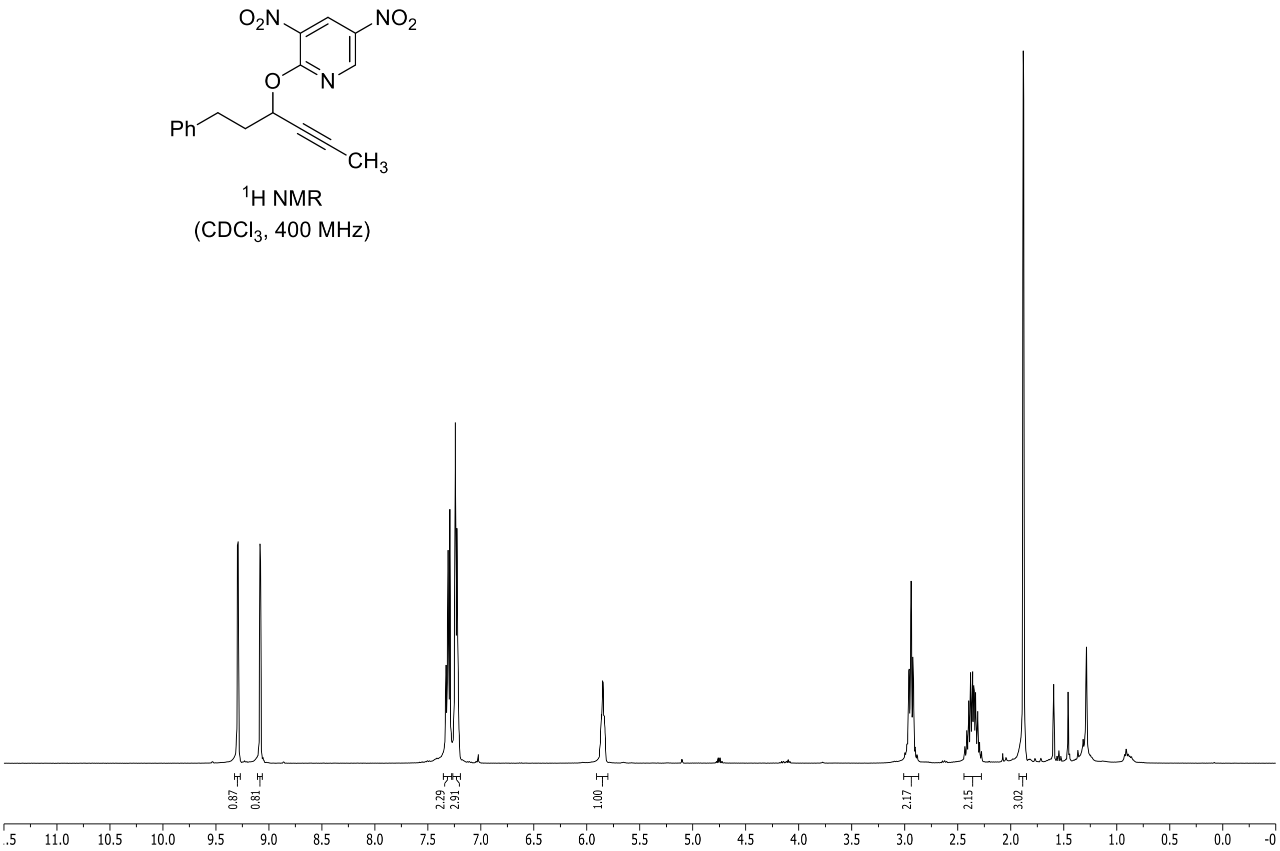
O2N
NO2
O
N
Ph
CH3
1H NMR
(CDCl3, 400 MHz)
0.87
0.81
2.29
2.91
1.00
2.17
2.15
3.02

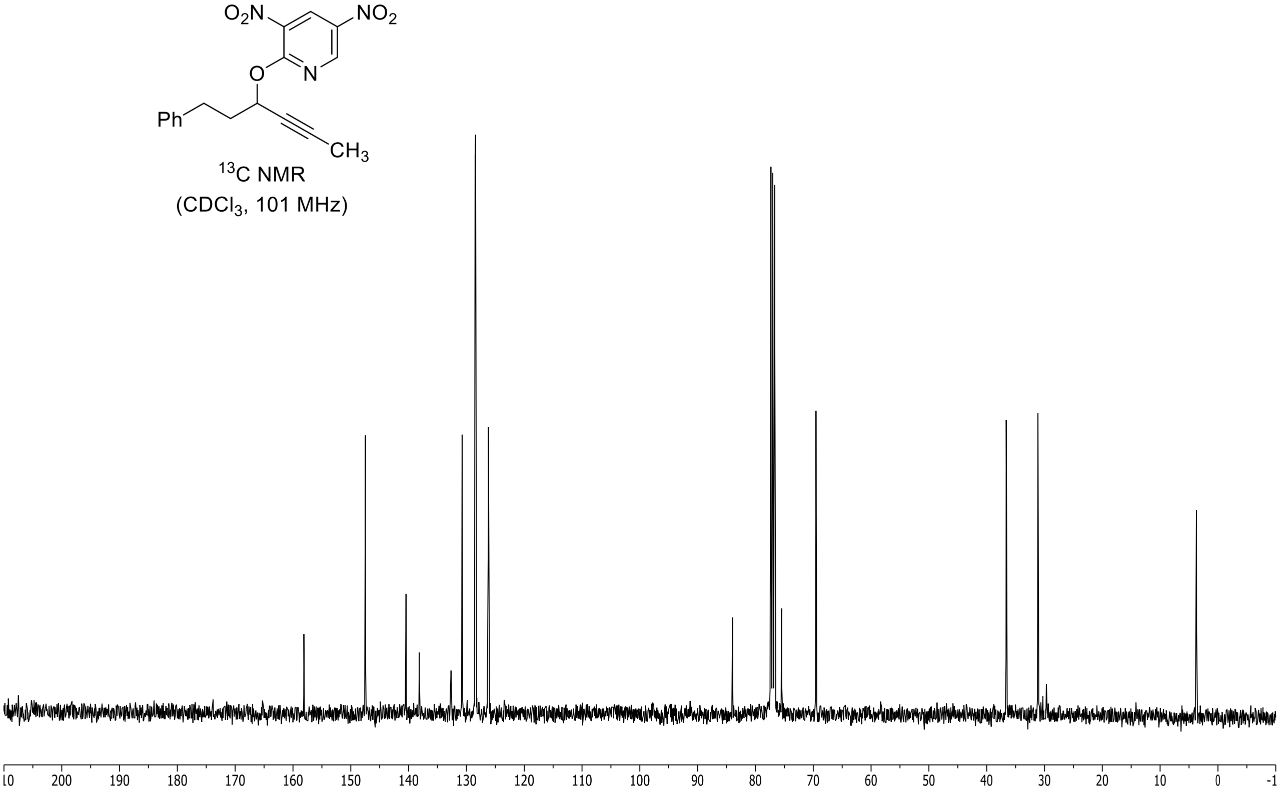
O2N
NO2
O
N
Ph
CH3
13C NMR
(CDCl3, 101 MHz)

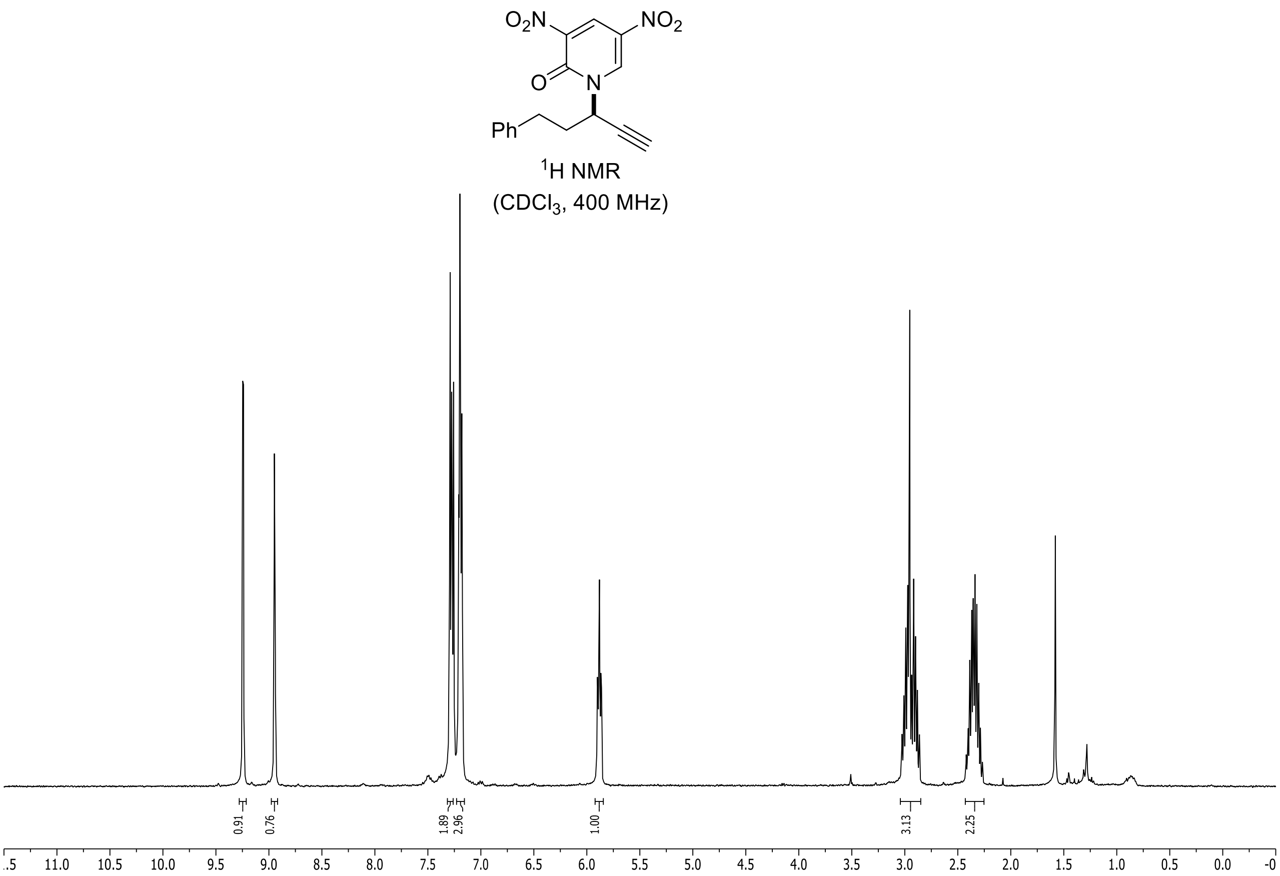
O_2N
NO_2
O
N
Ph
^{1}H NMR
($CDCl_3$, 400 MHz)
0.91
0.76
1.89
2.96
1.00
3.13
2.25

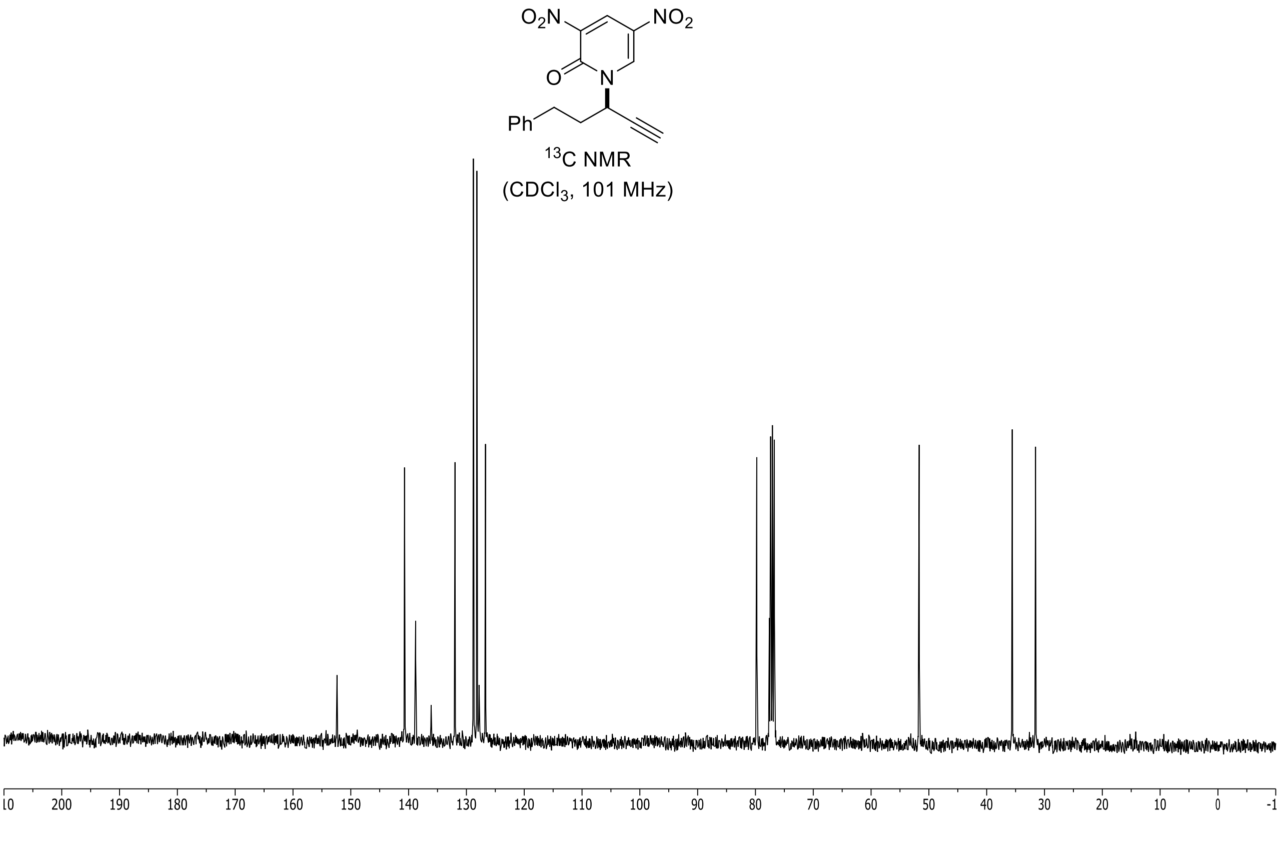
O_2N
NO_2
O
N
Ph
^{13}C NMR
($CDCl_3$, 101 MHz)

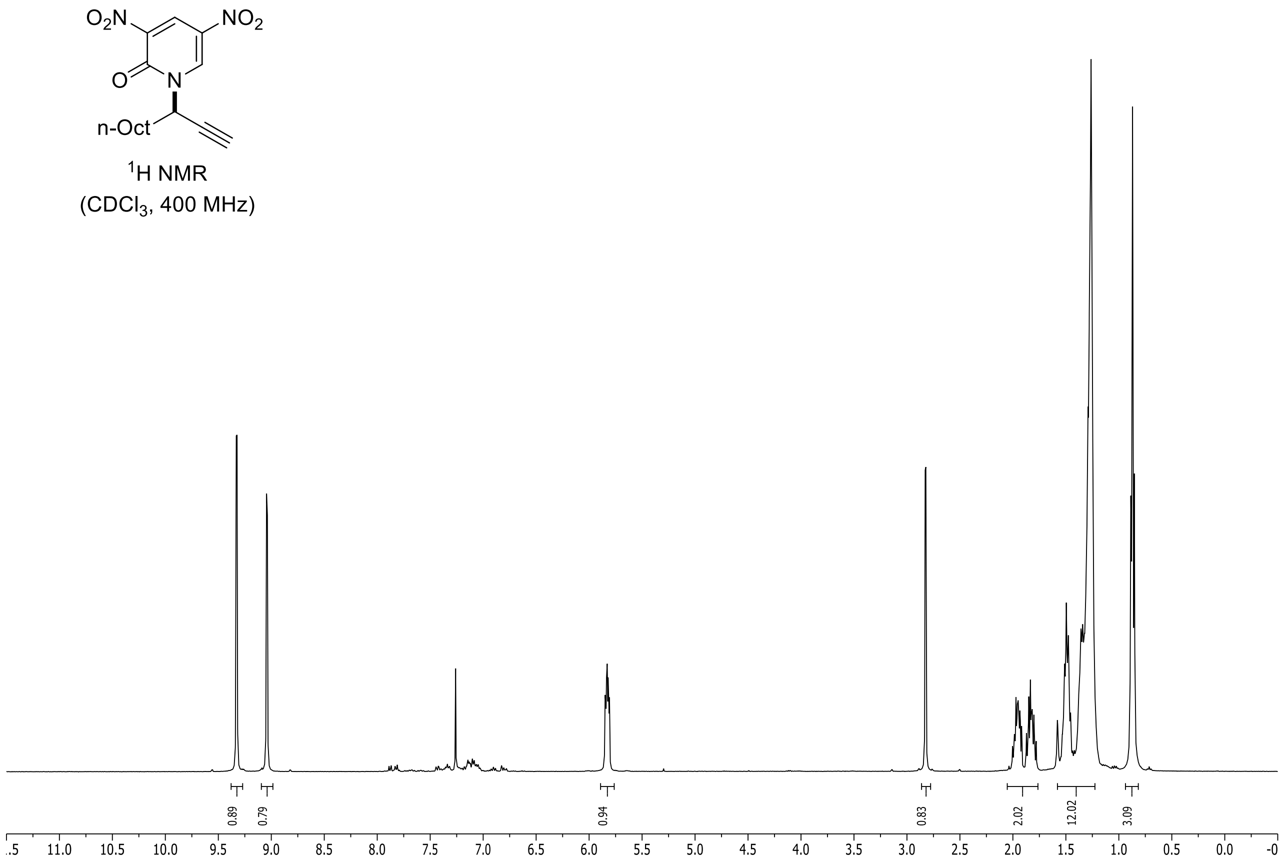
O2N
NO2
O
N
n-Oct
1H NMR
(CDCl3, 400 MHz)
0.89
0.79
0.94
0.83
2.02
12.02
3.09

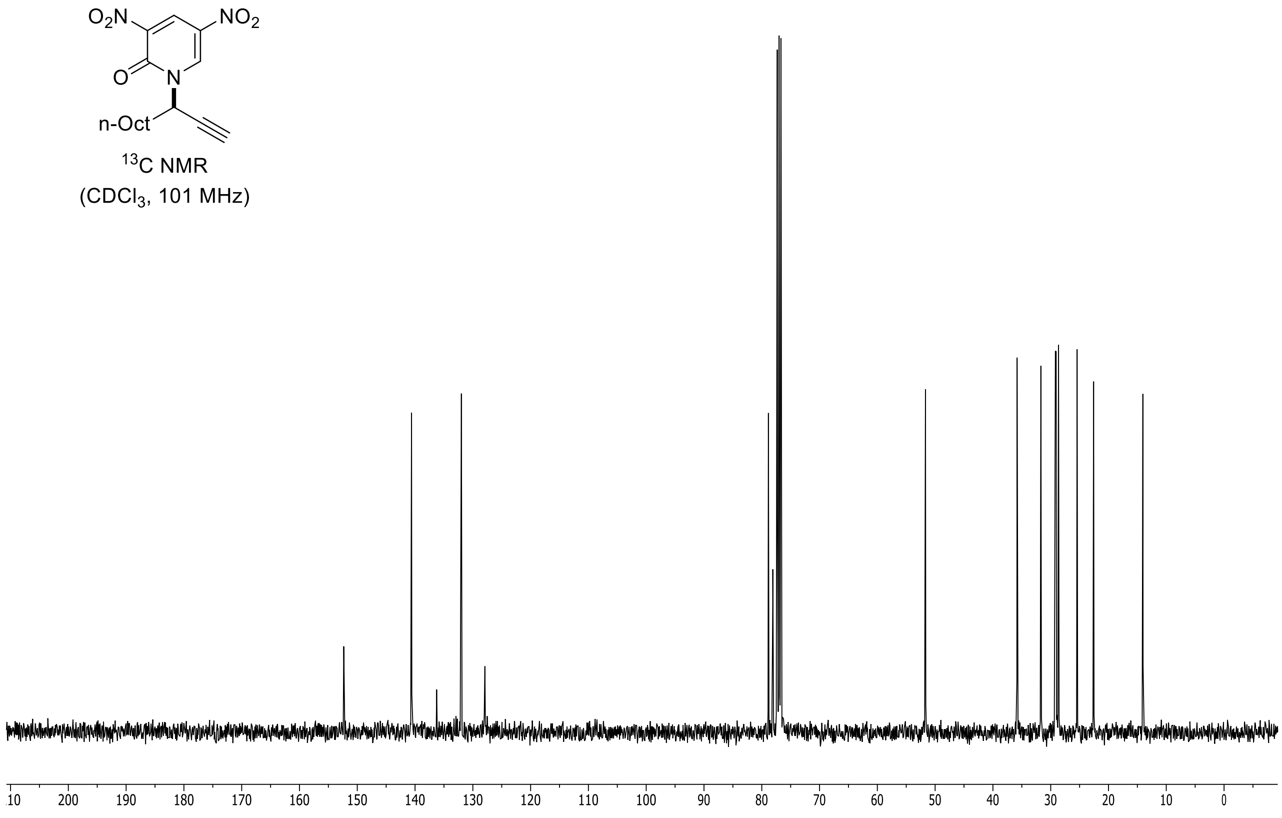
O2N
NO2
O
N
n-Oct
13C NMR
(CDCl3, 101 MHz)

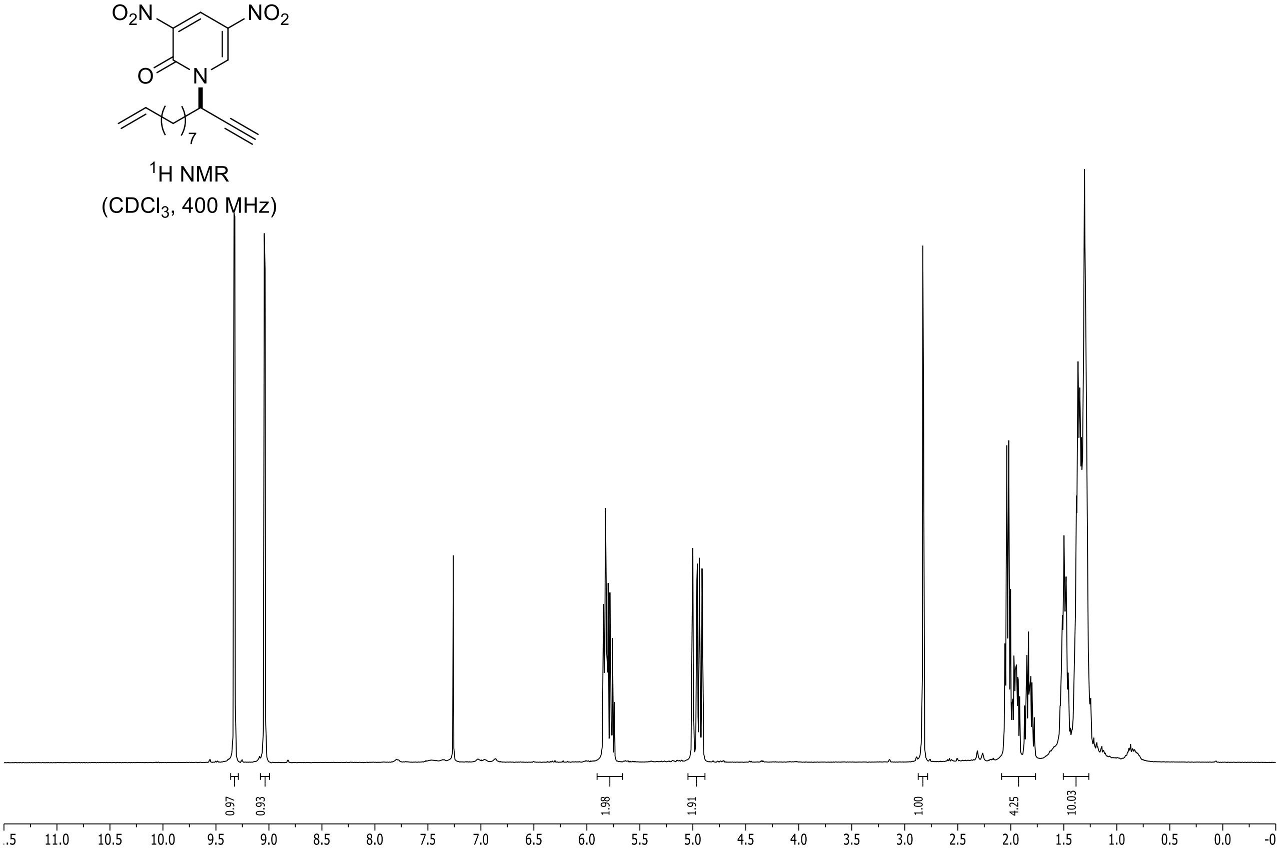
O_2N
NO_2
O
N
7
^{1}H NMR
($CDCl_3$, 400 MHz)

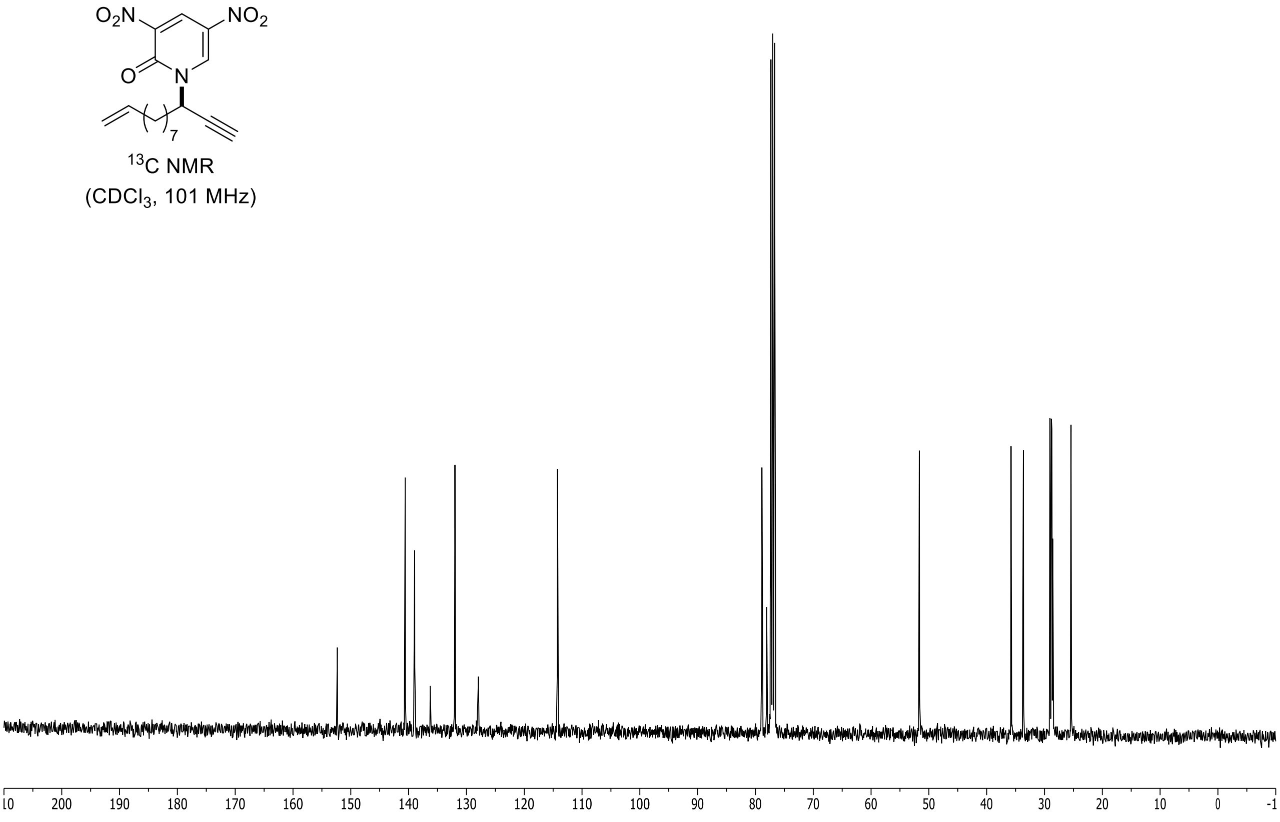
O_2N
NO_2
O
N
7
^{13}C NMR
($CDCl_3$, 101 MHz)

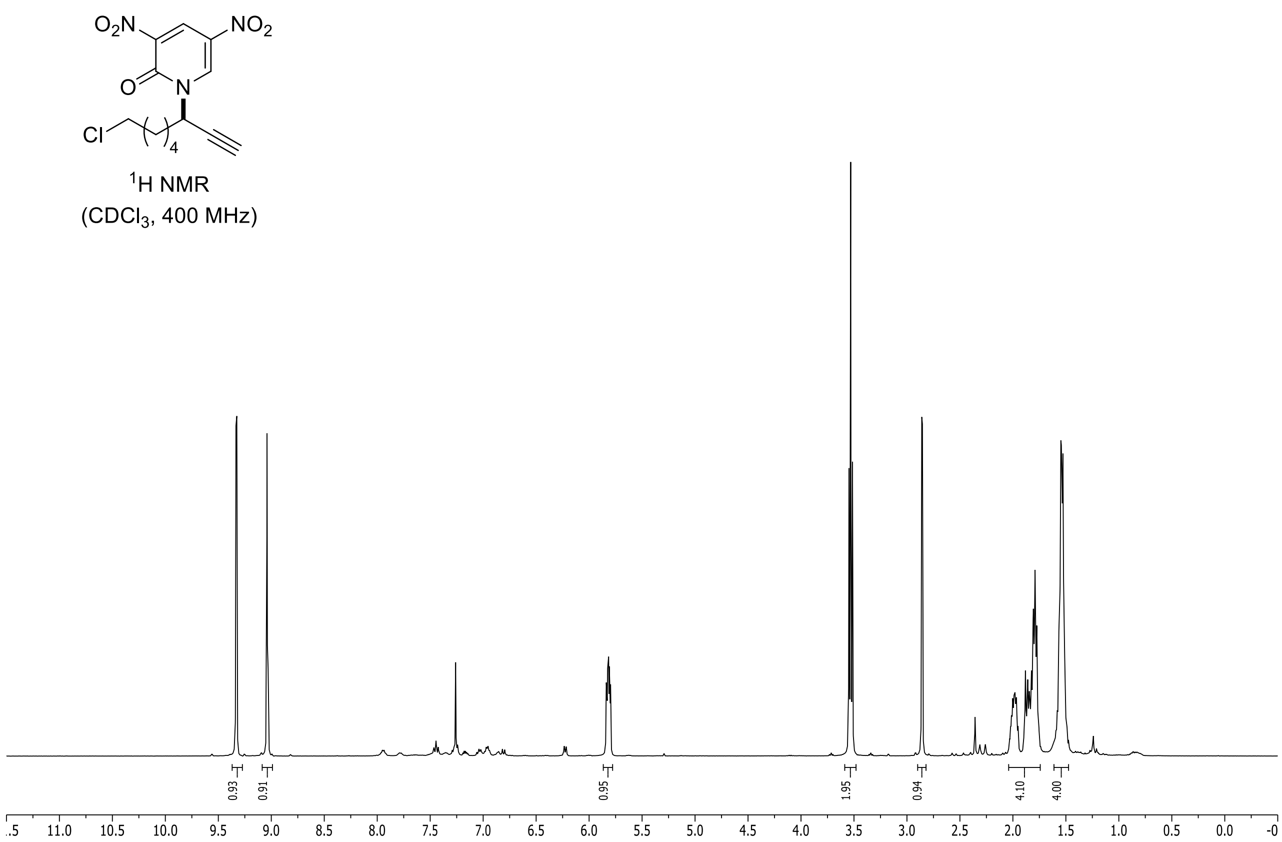

O_2N
NO_2
O
N
Cl
4
^1H NMR
($CDCl_3$, 400 MHz)

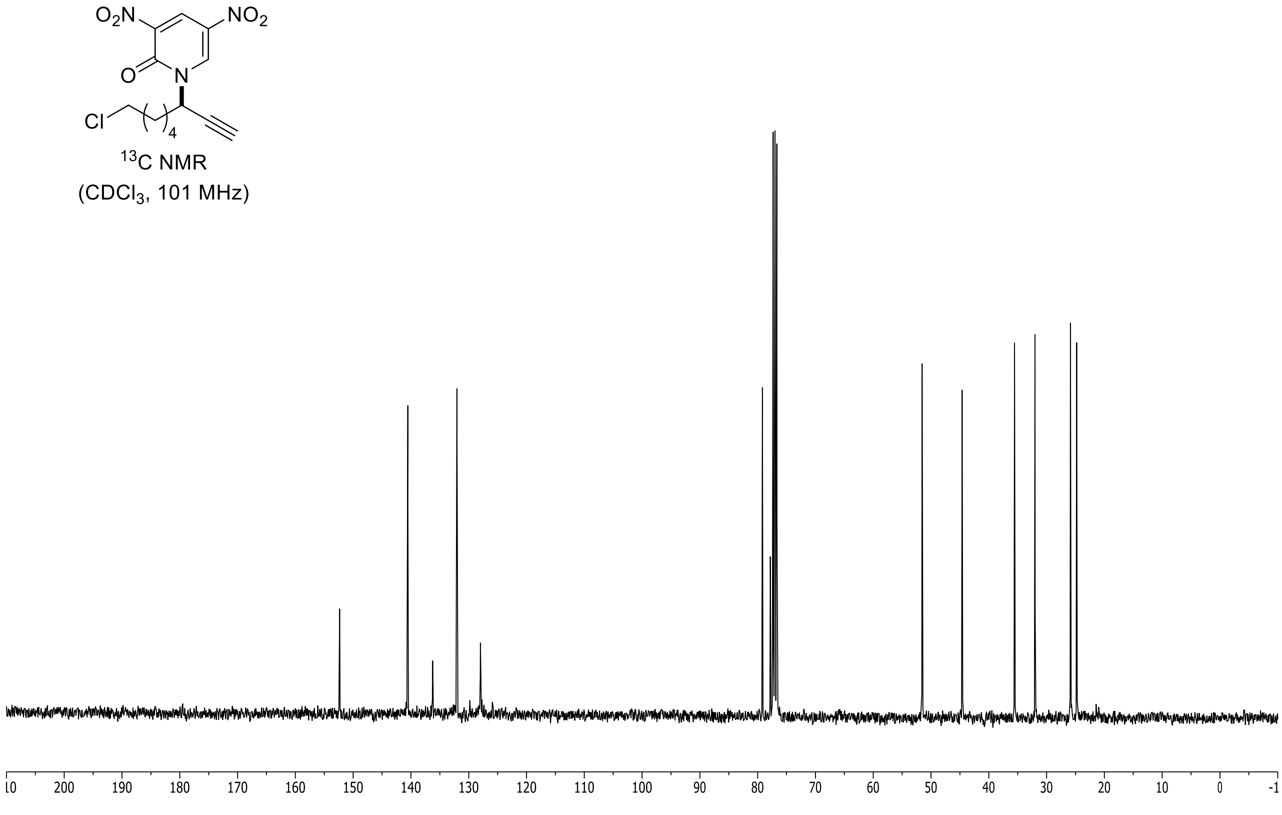

O_2N
NO_2
O
N
Cl
4
^{13}C NMR
($CDCl_3$, 101 MHz)

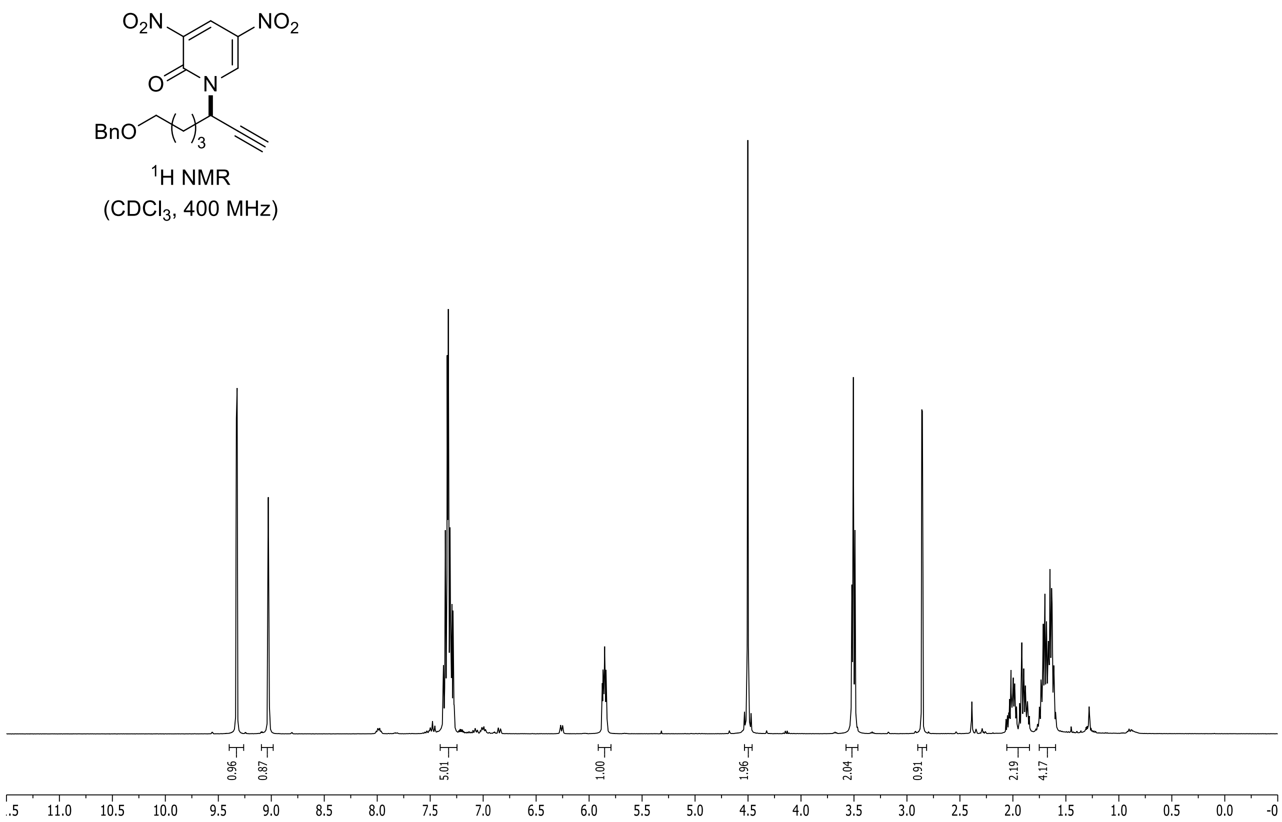
O2N
NO2
O
N
BnO
3
1H NMR
(CDCl3, 400 MHz)
0.96
0.87
5.01
1.00
1.96
2.04
0.91
2.19
4.17

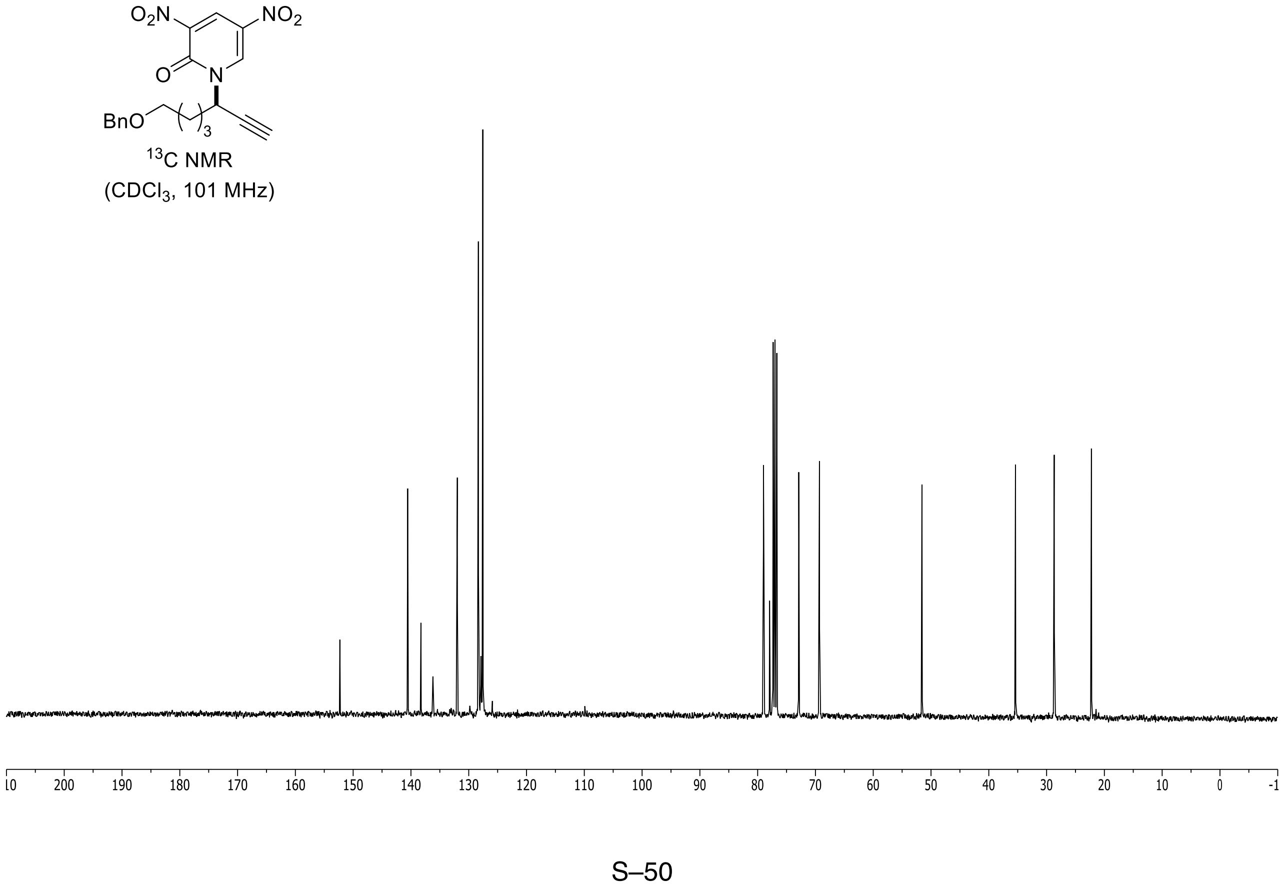
O2N
NO2
O
N
BnO
3
13C NMR
(CDCl3, 101 MHz)

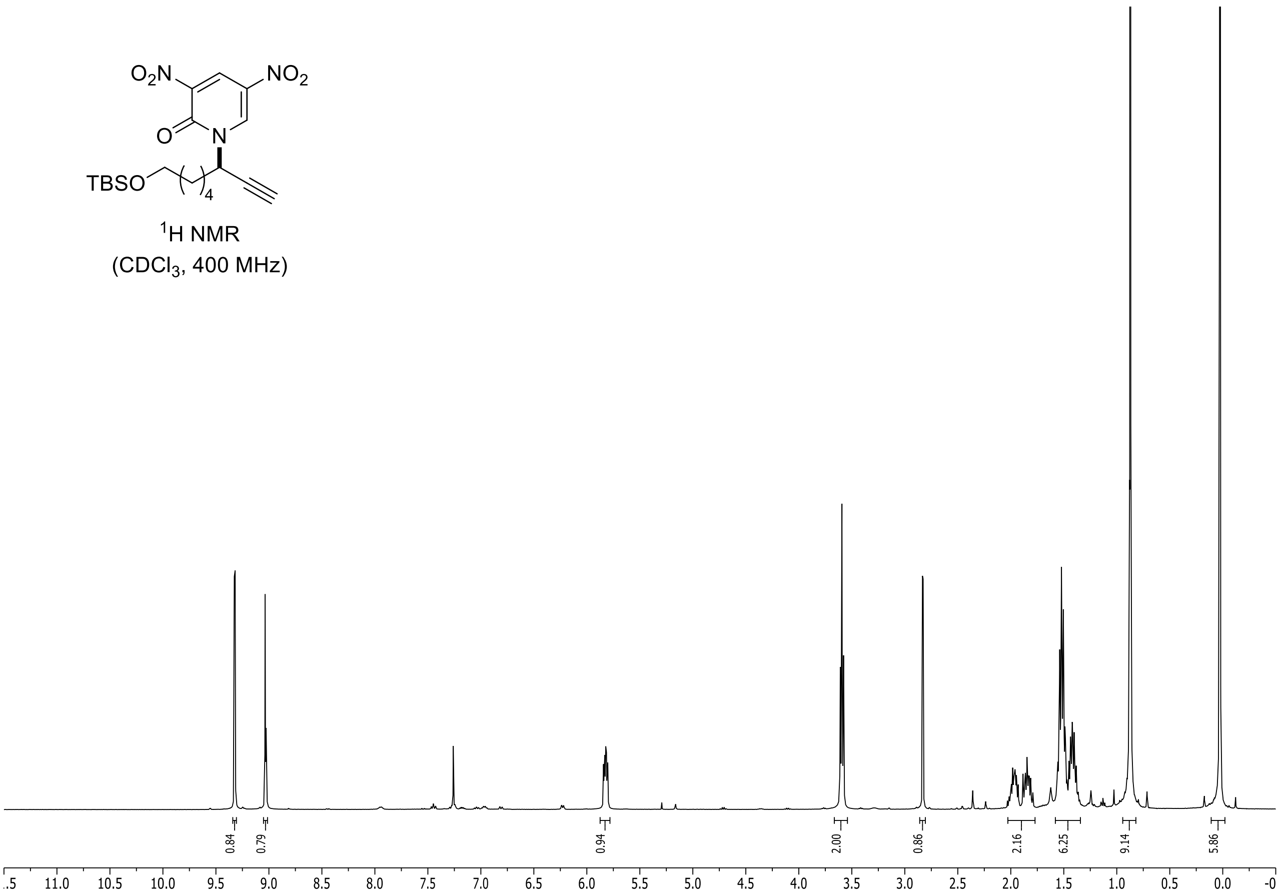
O2N
NO2
O
N
TBSO
4
1H NMR
(CDCl3, 400 MHz)
0.84
0.79
0.94
2.00
0.86
2.16
6.25
9.14
5.86
.5 11.0 10.5 10.0 9.5 9.0 8.5 8.0 7.5 7.0 6.5 6.0 5.5 5.0 4.5 4.0 3.5 3.0 2.5 2.0 1.5 1.0 0.5 0.0 -0

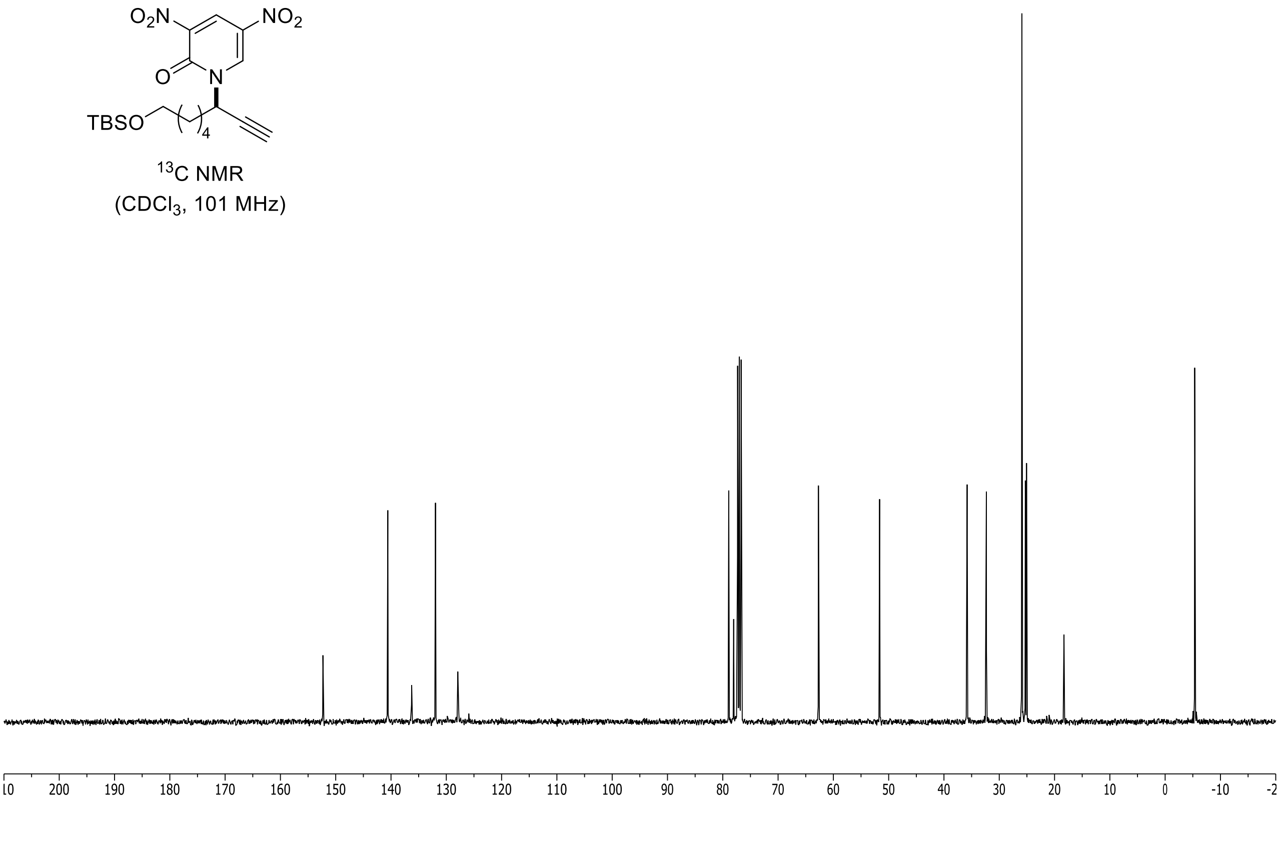
O2N
NO2
O
N
TBSO
4
13C NMR
(CDCl3, 101 MHz)
10 200 190 180 170 160 150 140 130 120 110 100 90 80 70 60 50 40 30 20 10 0 -10 -2

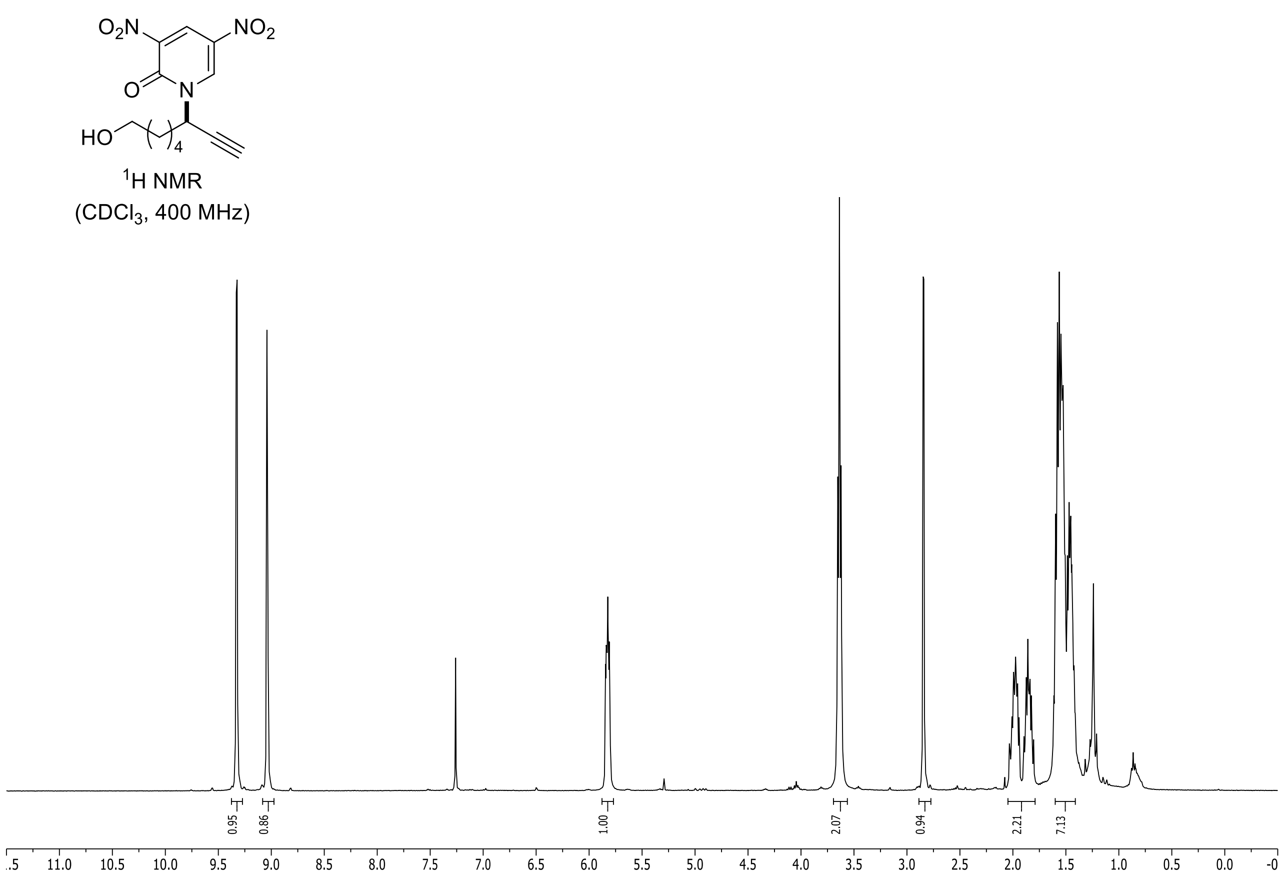
O2N
NO2
O
N
HO
4
1H NMR
(CDCl3, 400 MHz)
0.95
0.86
1.00
2.07
0.94
2.21
7.13
11.0 10.5 10.0 9.5 9.0 8.5 8.0 7.5 7.0 6.5 6.0 5.5 5.0 4.5 4.0 3.5 3.0 2.5 2.0 1.5 1.0 0.5 0.0

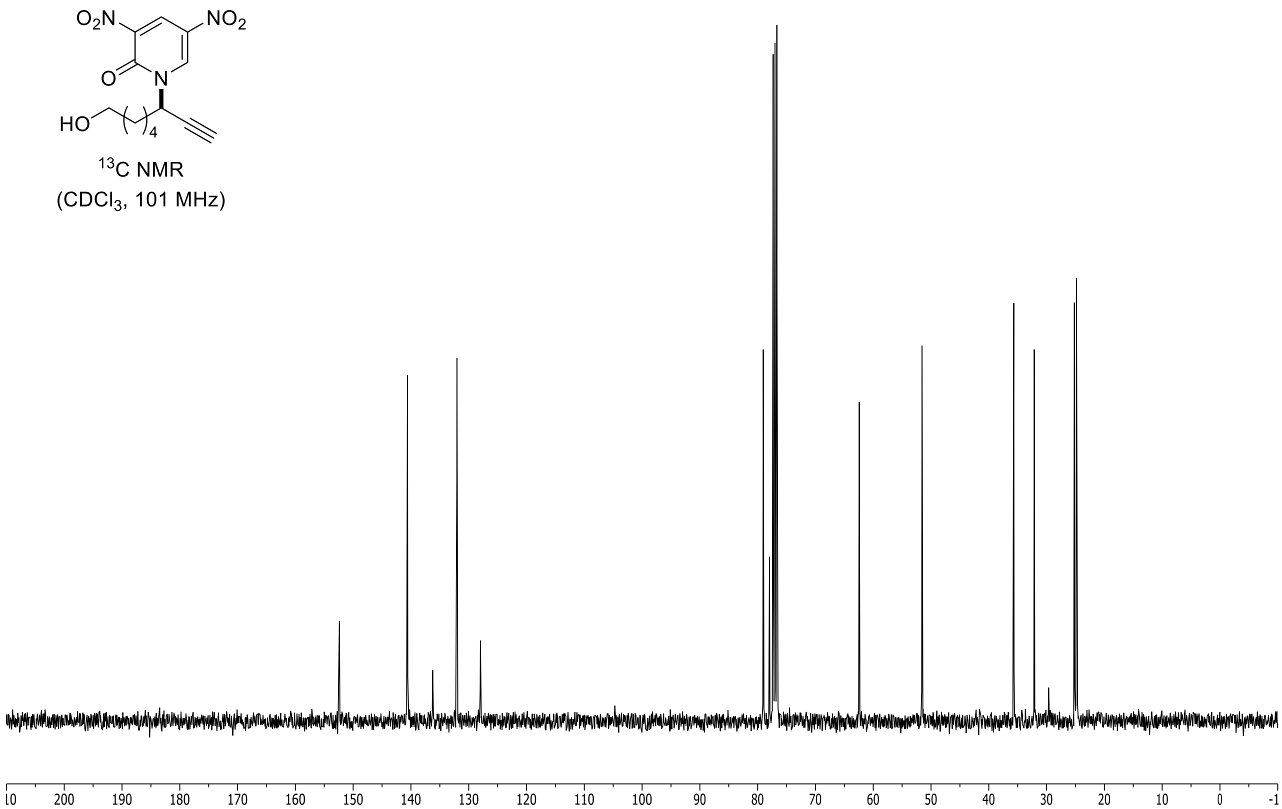
O2N
NO2
O
N
HO
4
13C NMR
(CDCl3, 101 MHz)
200 190 180 170 160 150 140 130 120 110 100 90 80 70 60 50 40 30 20 10 0

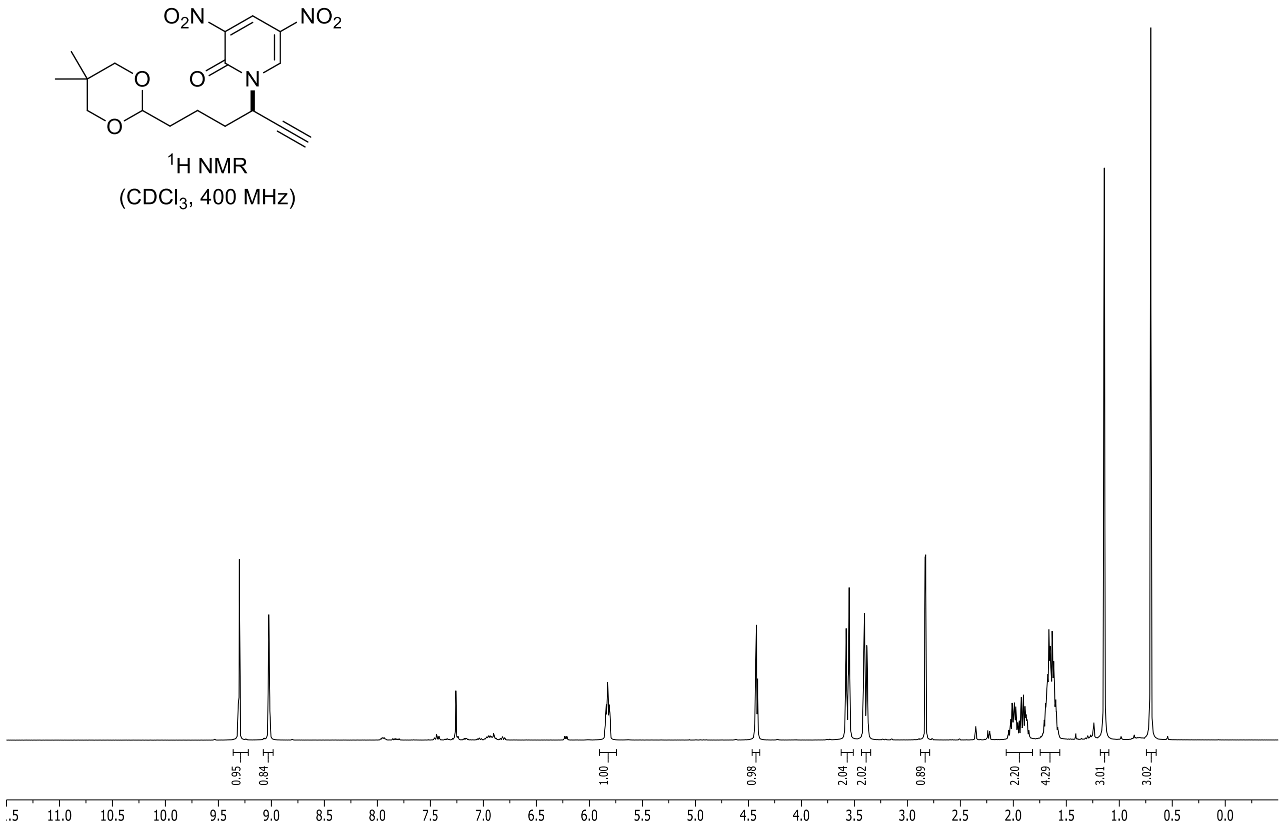
O2N
NO2
O
O
O
N
1H NMR
(CDCl3, 400 MHz)
0.95
0.84
1.00
0.98
2.04
2.02
0.89
2.20
4.29
3.01
3.02

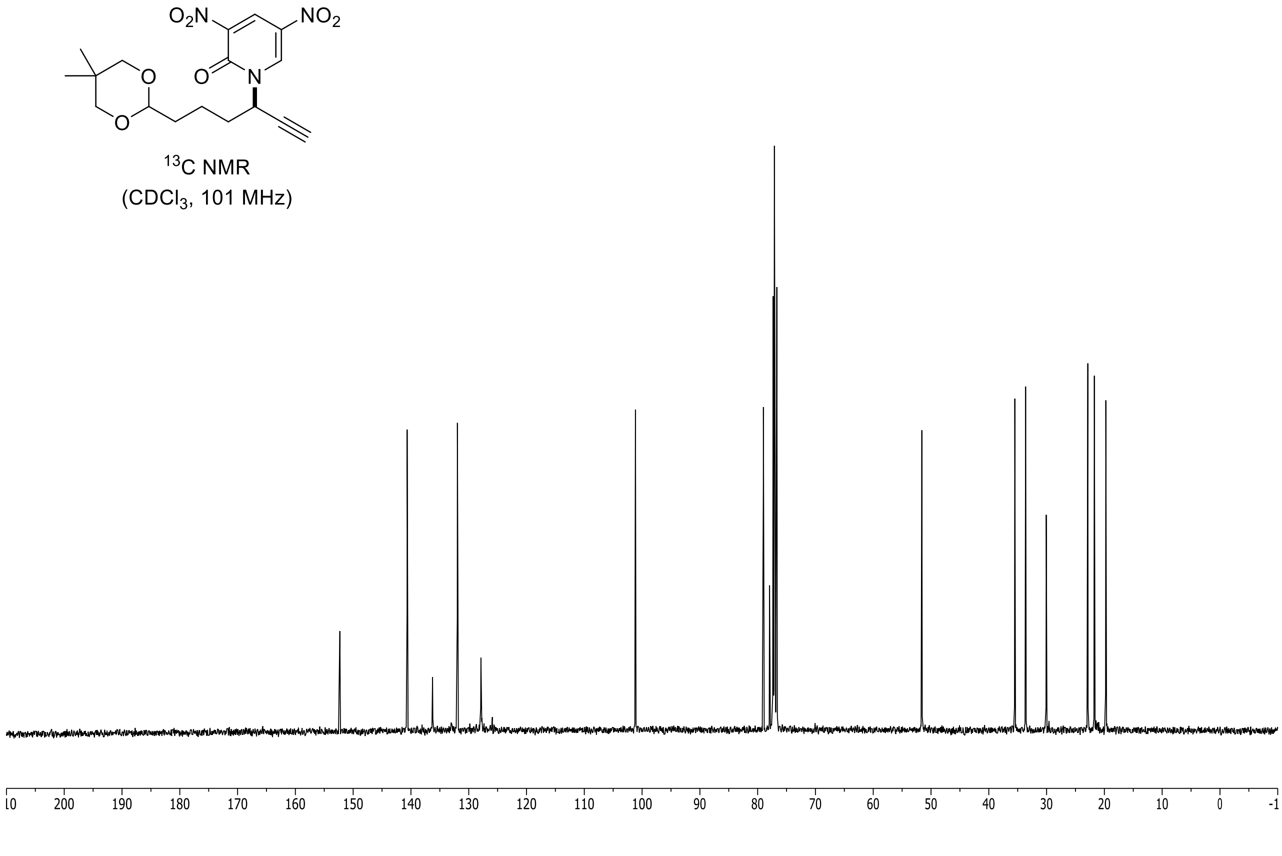
O2N
NO2
O
O
O
N
13C NMR
(CDCl3, 101 MHz)

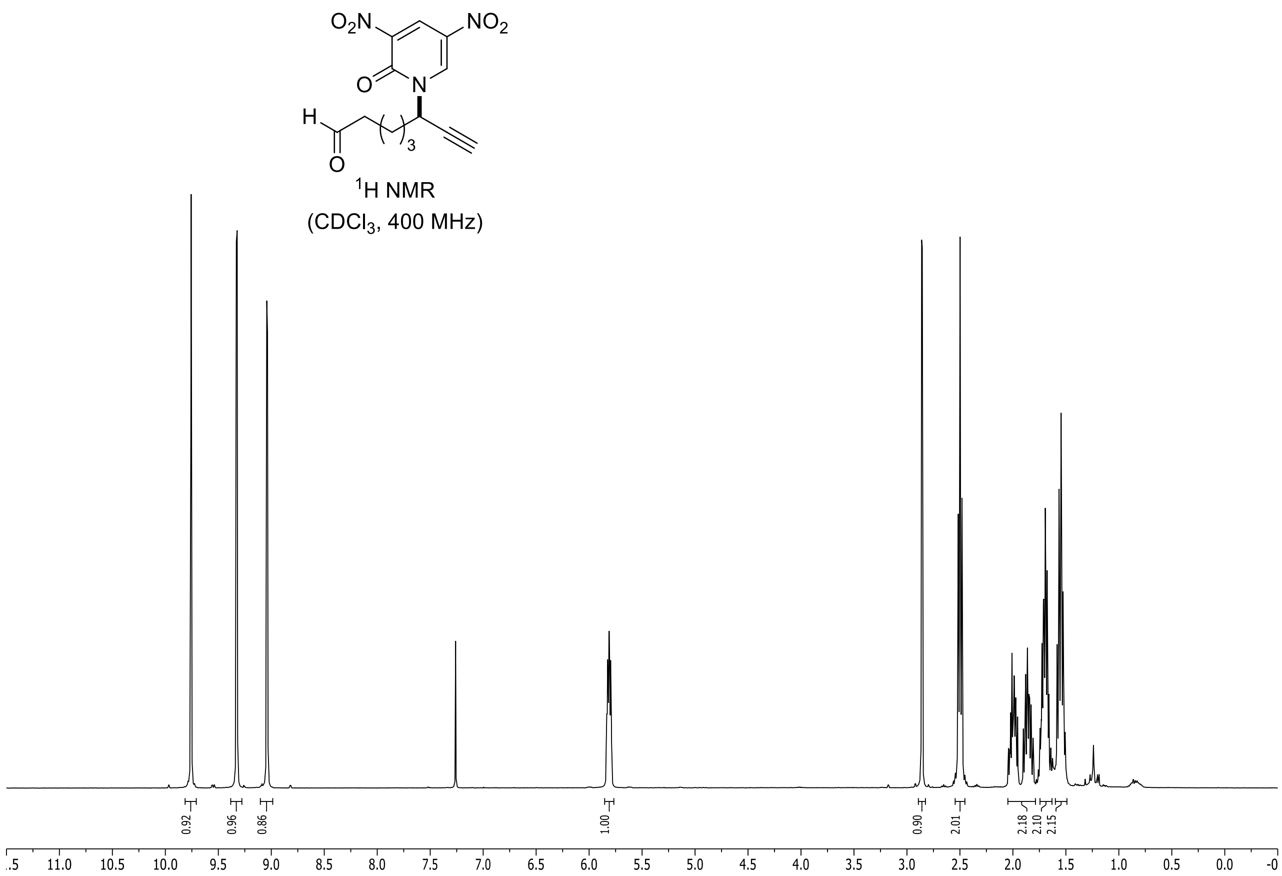
O2N
NO2
O
N
H
O
3
1H NMR
(CDCl3, 400 MHz)
0.92
0.96
0.86
1.00
0.90
2.01
2.18
2.10
2.15
11.0
10.5
10.0
9.5
9.0
8.5
8.0
7.5
7.0
6.5
6.0
5.5
5.0
4.5
4.0
3.5
3.0
2.5
2.0
1.5
1.0
0.5
0.0

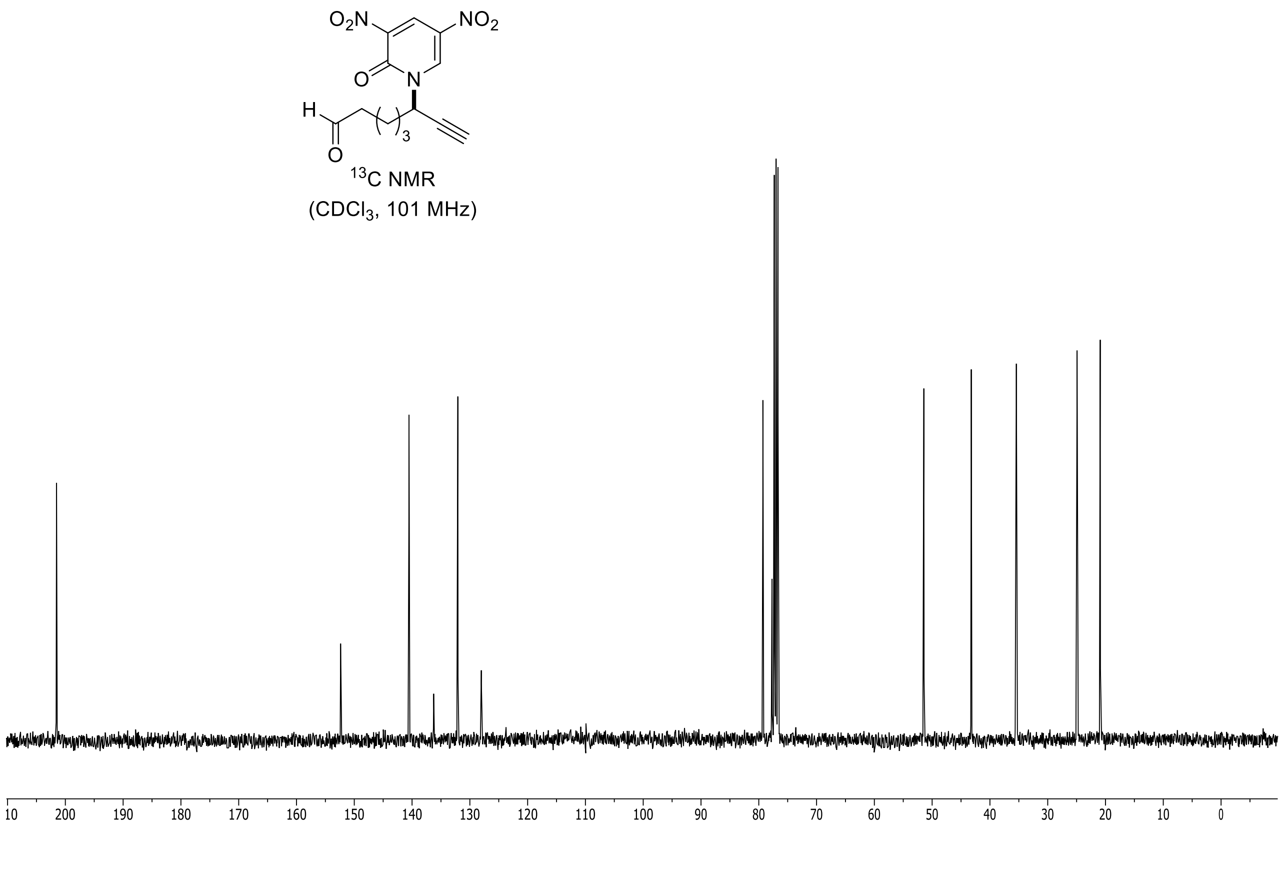
O2N
NO2
O
N
H
O
3
13C NMR
(CDCl3, 101 MHz)
200
190
180
170
160
150
140
130
120
110
100
90
80
70
60
50
40
30
20
10
0

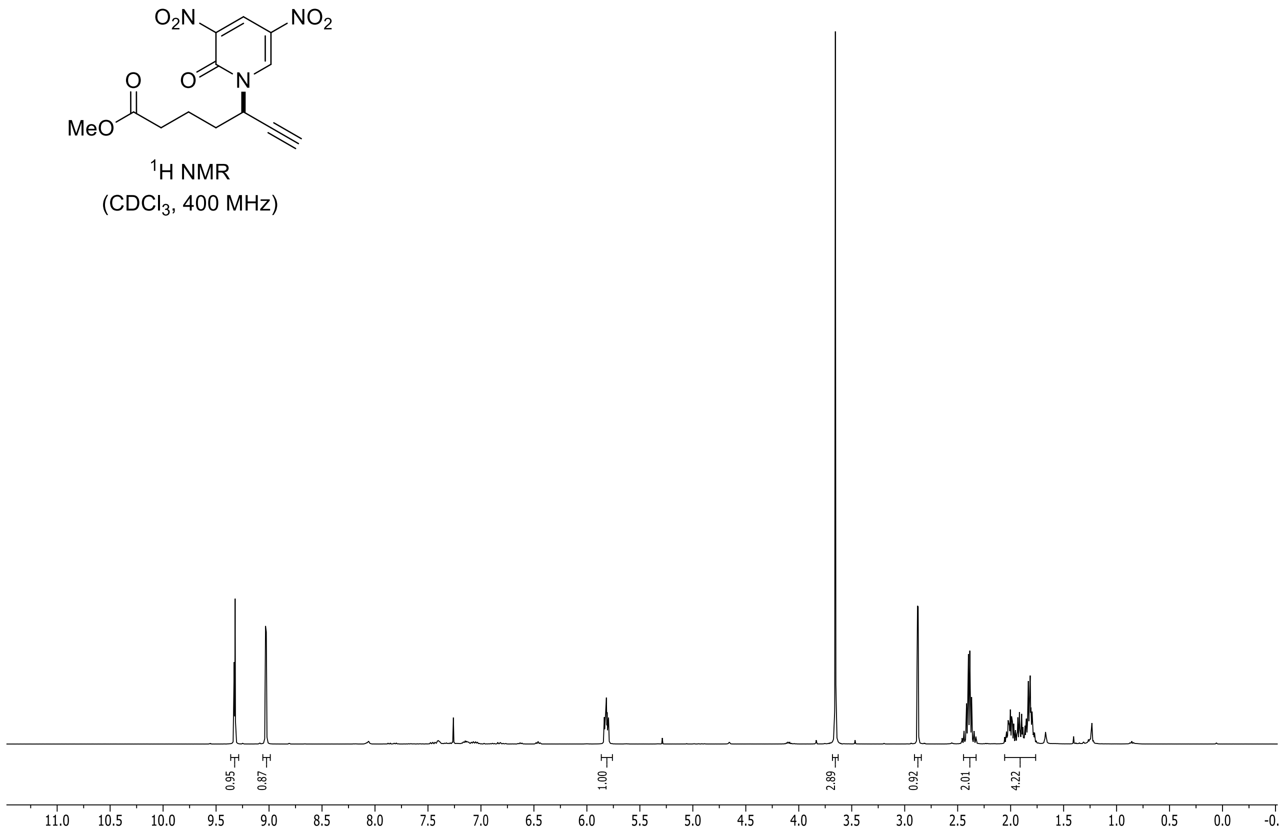
O2N
NO2
O
O
N
MeO
1H NMR
(CDCl3, 400 MHz)
0.95
0.87
1.00
2.89
0.92
2.01
4.22
11.0 10.5 10.0 9.5 9.0 8.5 8.0 7.5 7.0 6.5 6.0 5.5 5.0 4.5 4.0 3.5 3.0 2.5 2.0 1.5 1.0 0.5 0.0

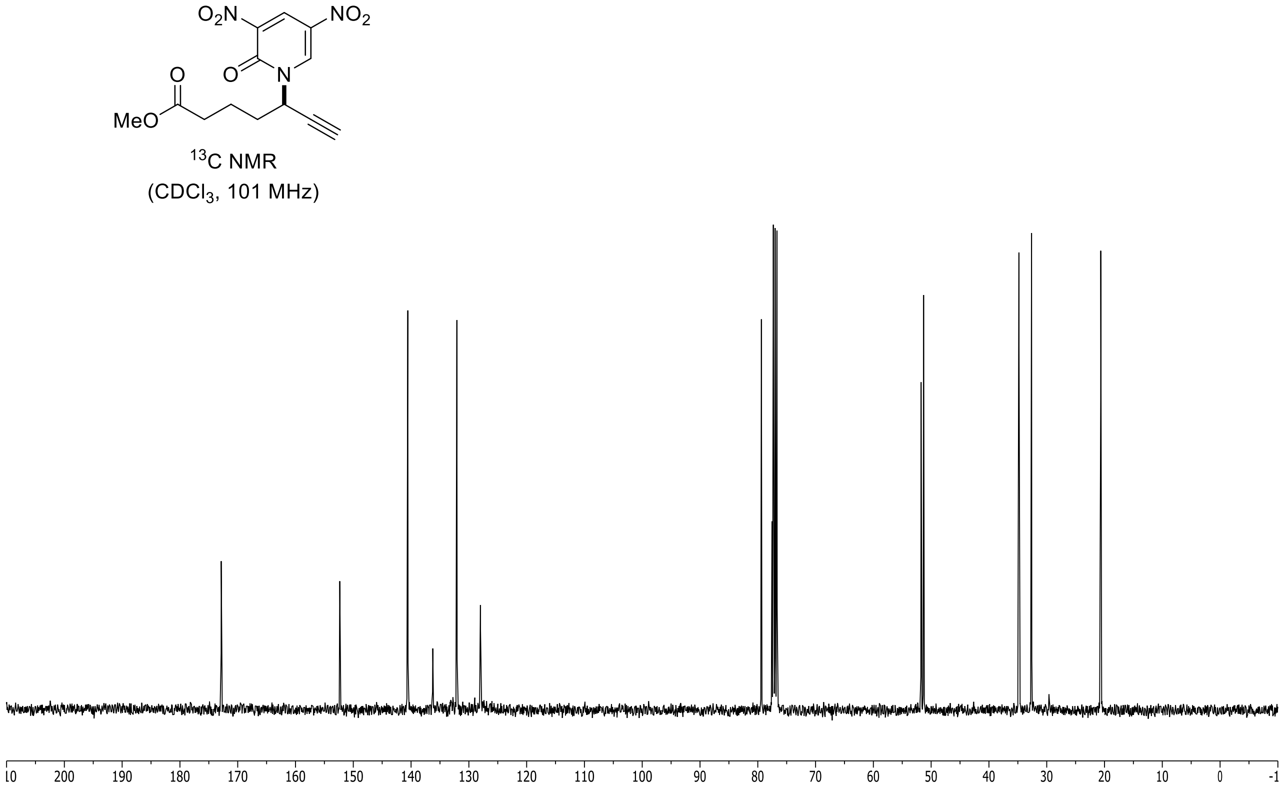
O2N
NO2
O
O
N
MeO
13C NMR
(CDCl3, 101 MHz)
200 190 180 170 160 150 140 130 120 110 100 90 80 70 60 50 40 30 20 10 0

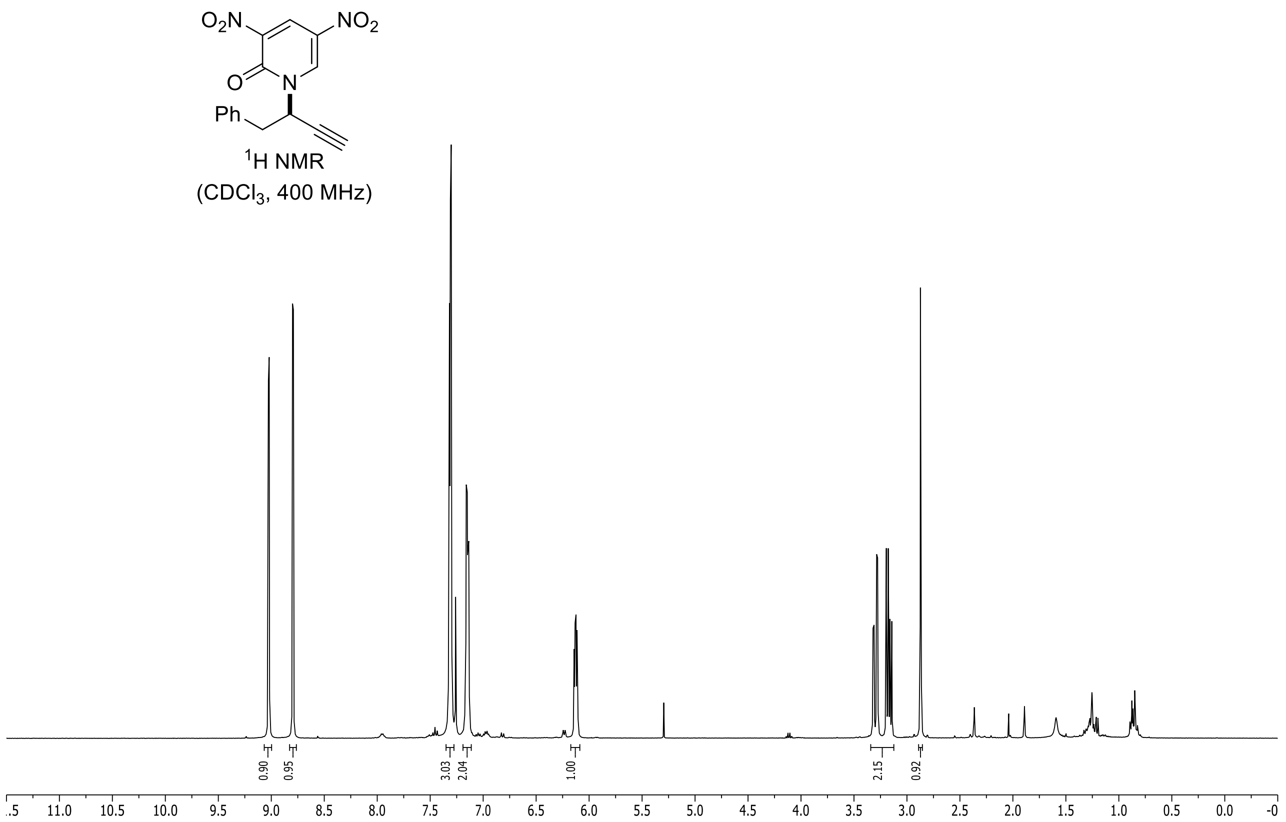

O_2N
NO_2
O
N
Ph
^1H NMR
($CDCl_3$, 400 MHz)
0.90
0.95
3.03
2.04
1.00
2.15
0.92
11.0 10.5 10.0 9.5 9.0 8.5 8.0 7.5 7.0 6.5 6.0 5.5 5.0 4.5 4.0 3.5 3.0 2.5 2.0 1.5 1.0 0.5 0.0

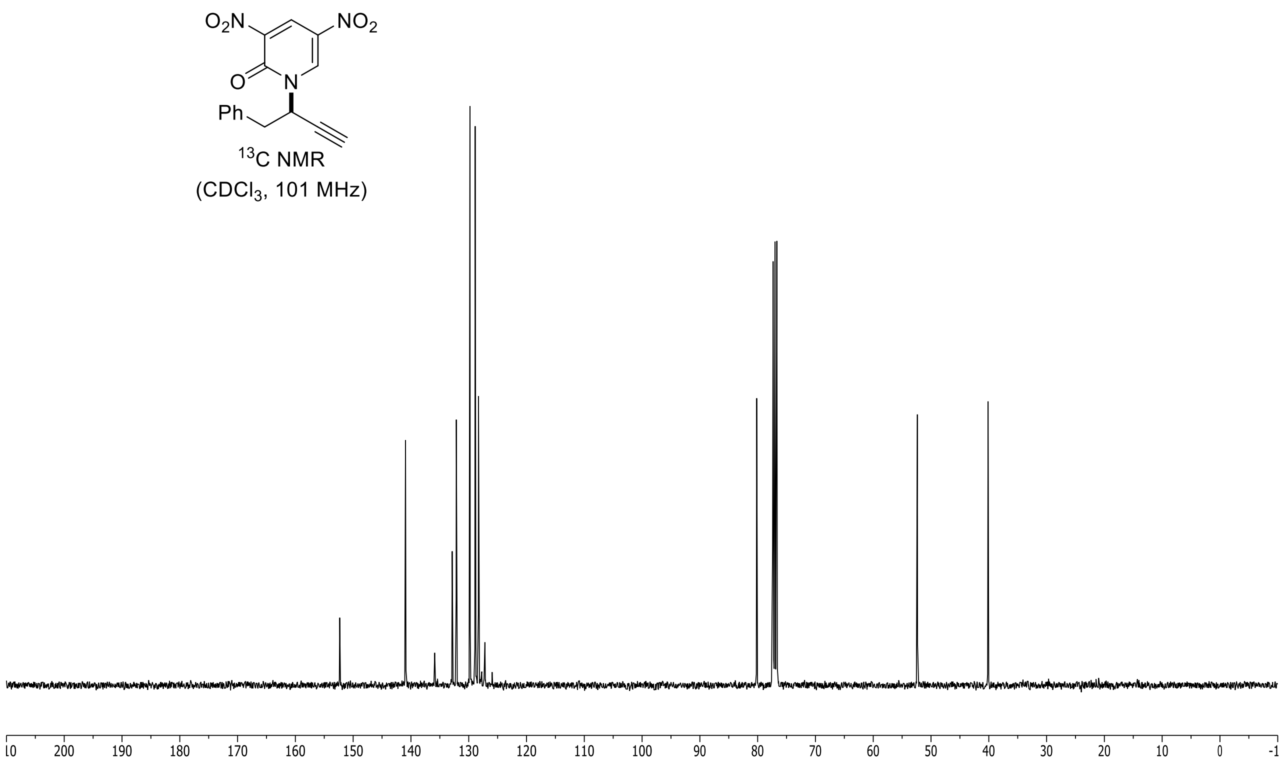

O_2N
NO_2
O
N
Ph
^{13}C NMR
($CDCl_3$, 101 MHz)
200 190 180 170 160 150 140 130 120 110 100 90 80 70 60 50 40 30 20 10 0

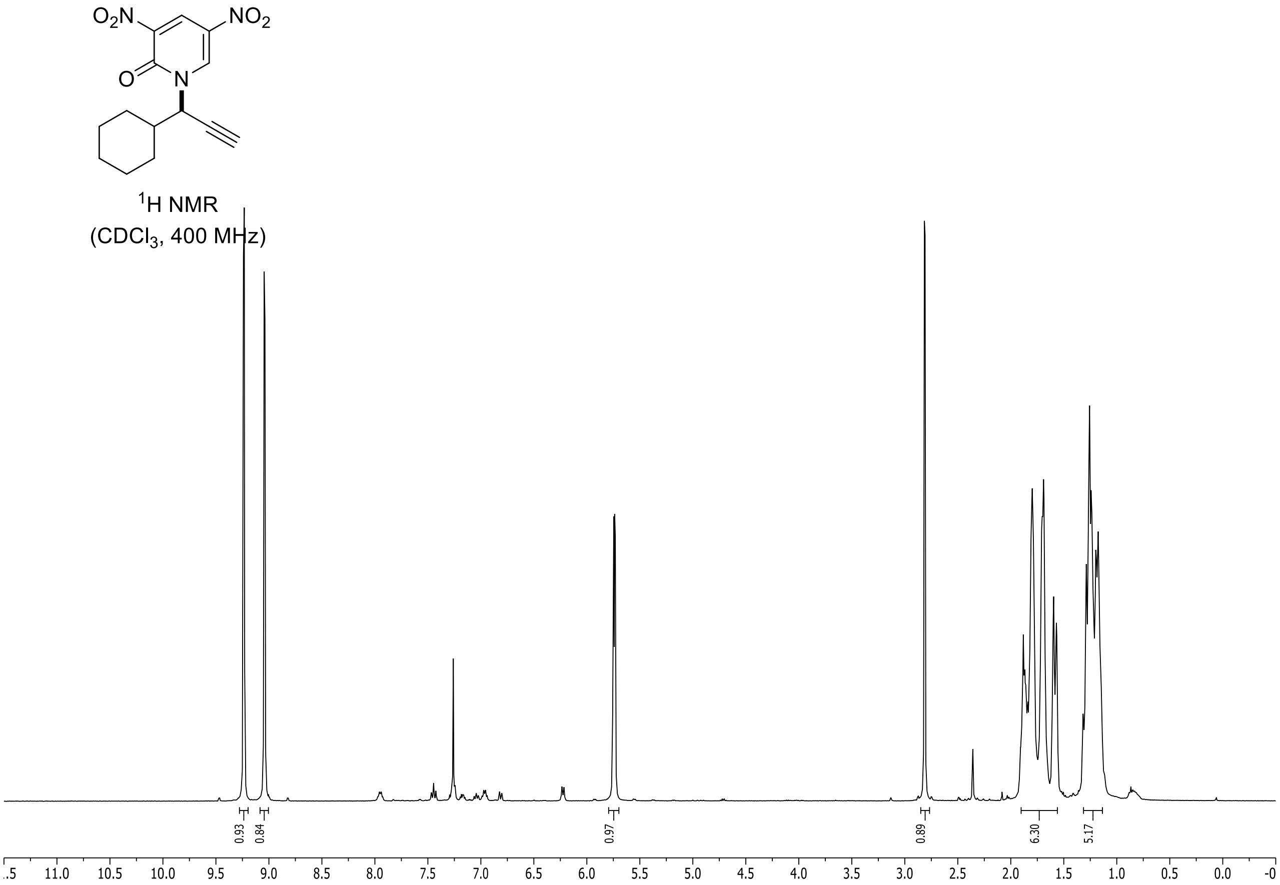
O2N
NO2
O
N
1H NMR
(CDCl3, 400 MHz)
0.93
0.84
0.97
0.89
6.30
5.17
11.0 10.5 10.0 9.5 9.0 8.5 8.0 7.5 7.0 6.5 6.0 5.5 5.0 4.5 4.0 3.5 3.0 2.5 2.0 1.5 1.0 0.5 0.0

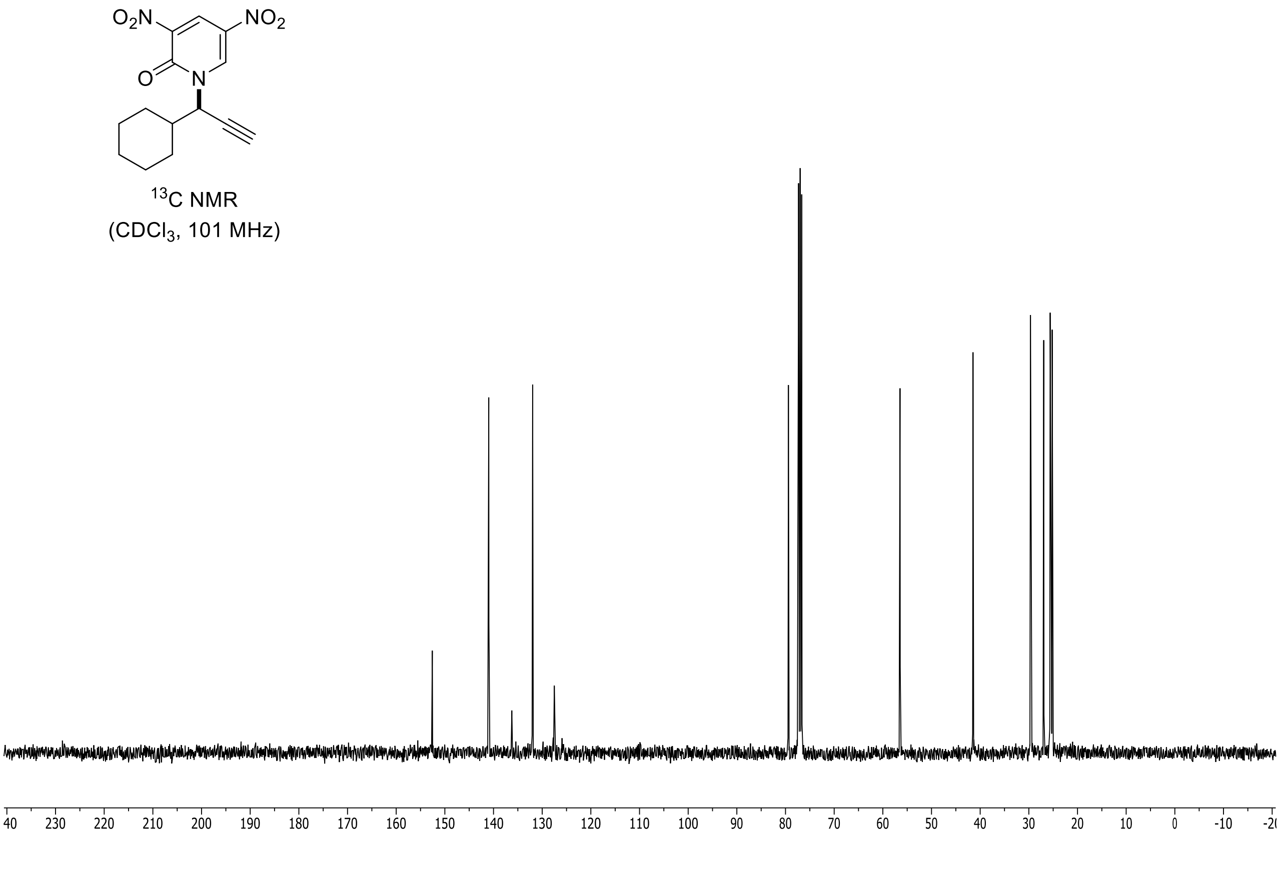
O2N
NO2
O
N
13C NMR
(CDCl3, 101 MHz)
230 220 210 200 190 180 170 160 150 140 130 120 110 100 90 80 70 60 50 40 30 20 10 0 -10

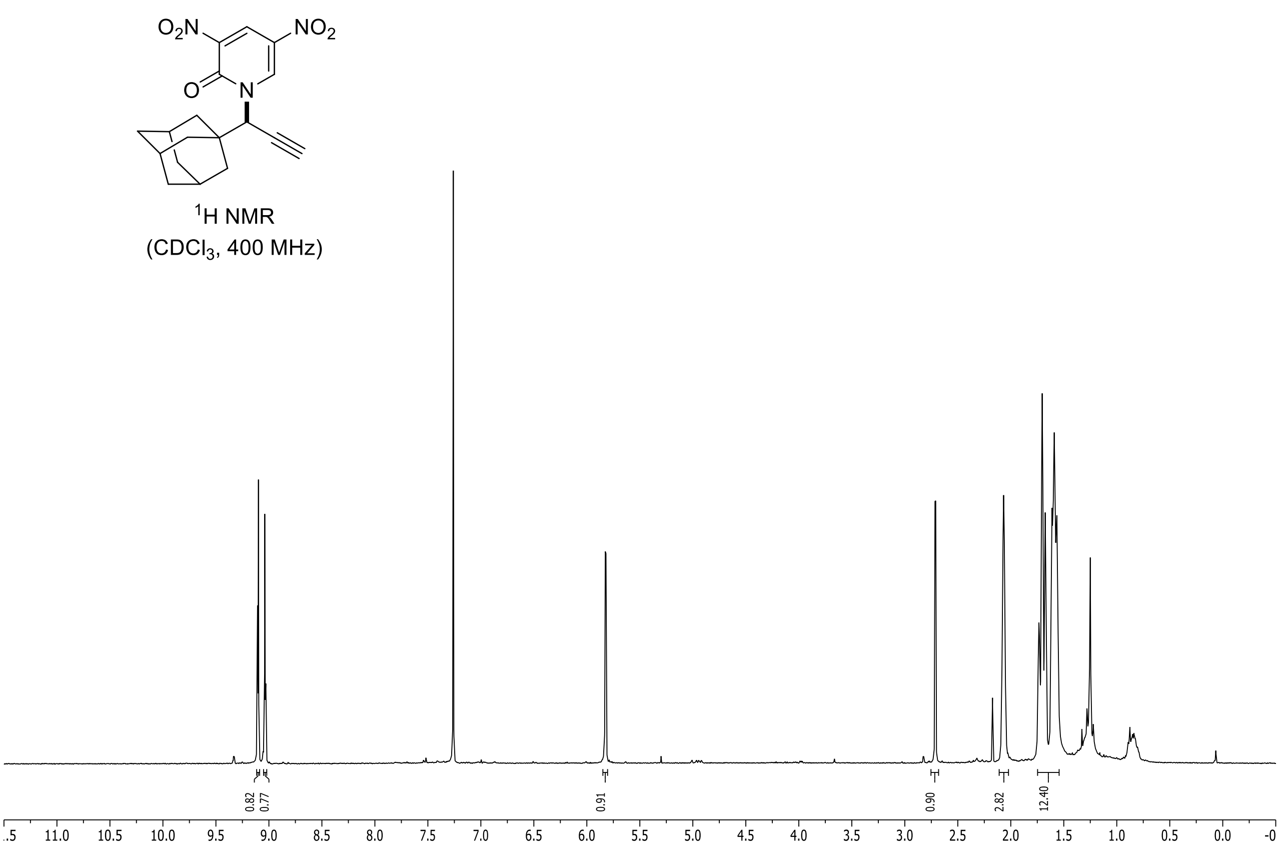
O2N
NO2
O
N
1H NMR
(CDCl3, 400 MHz)
0.82
0.77
0.91
0.90
2.82
12.40

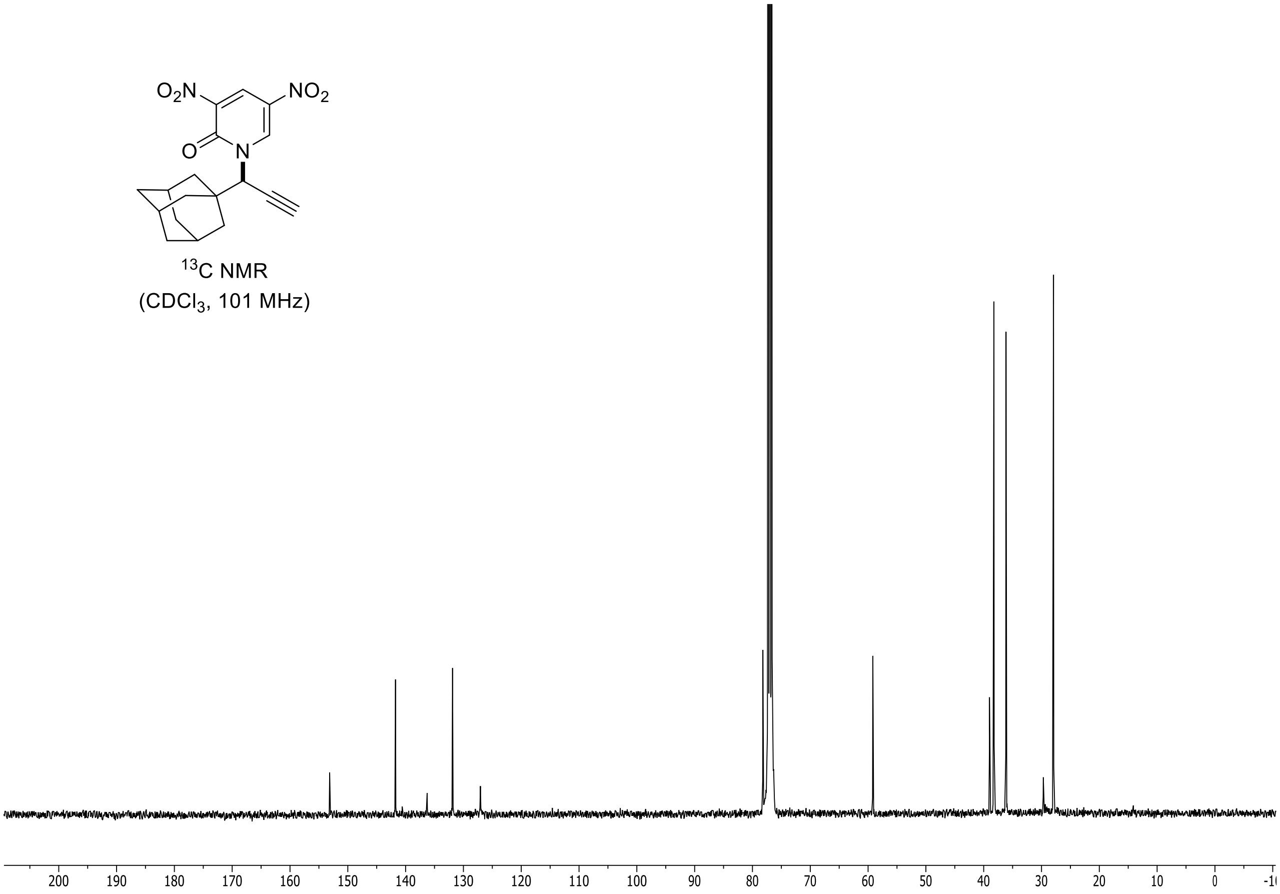
O2N
NO2
O
N
13C NMR
(CDCl3, 101 MHz)

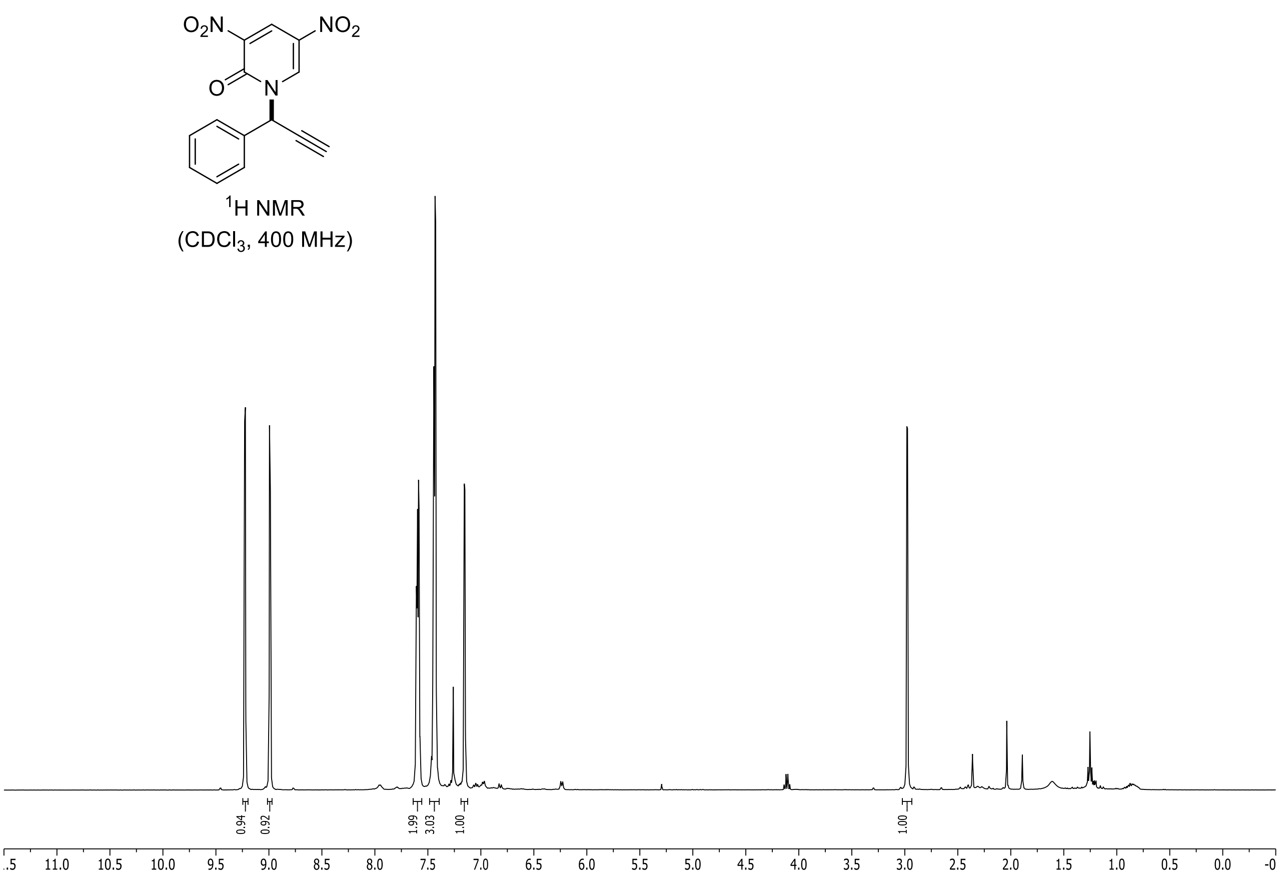

O_2N
NO_2
O
N
^1H NMR
($CDCl_3$, 400 MHz)
0.94
0.92
1.99
3.03
1.00
1.00

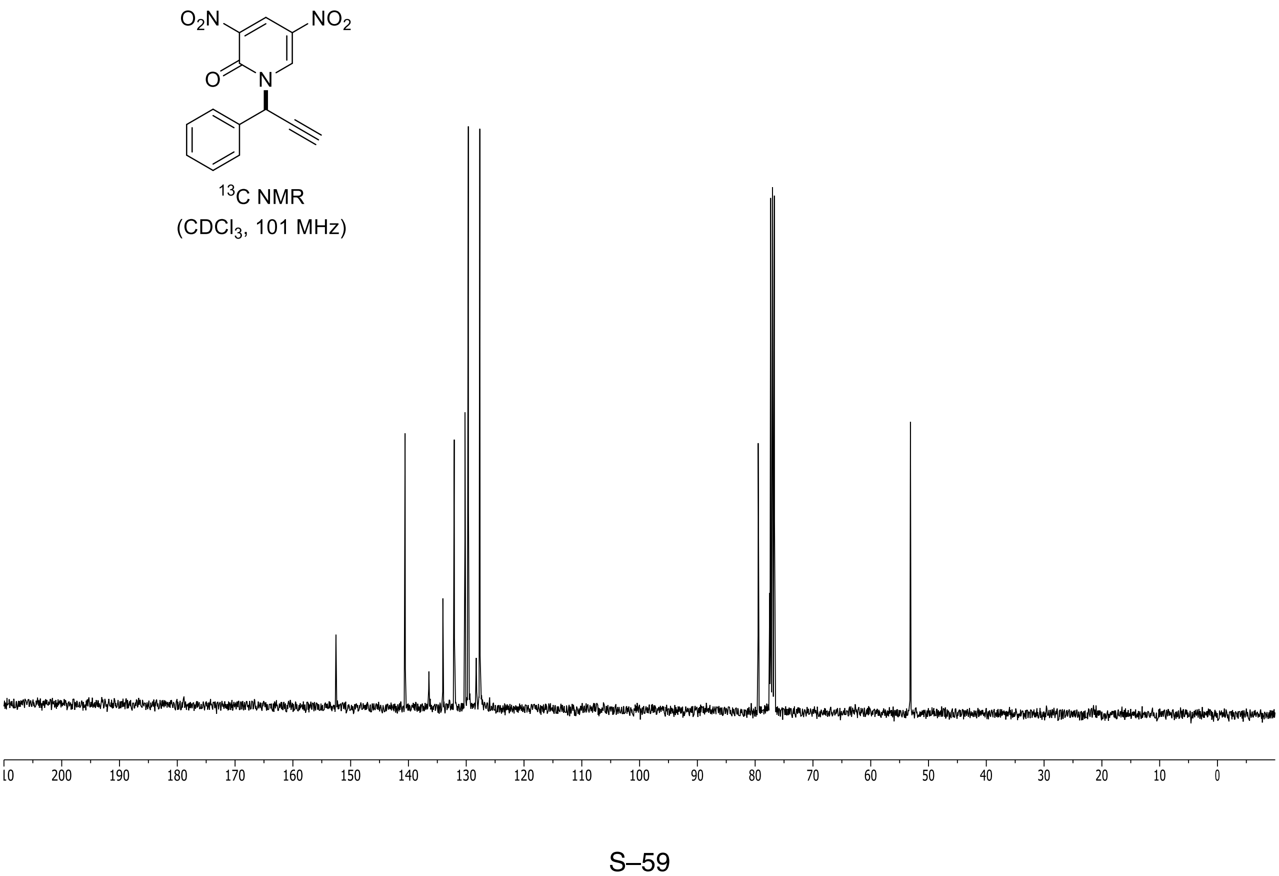

O_2N
NO_2
O
N
^{13}C NMR
($CDCl_3$, 101 MHz)

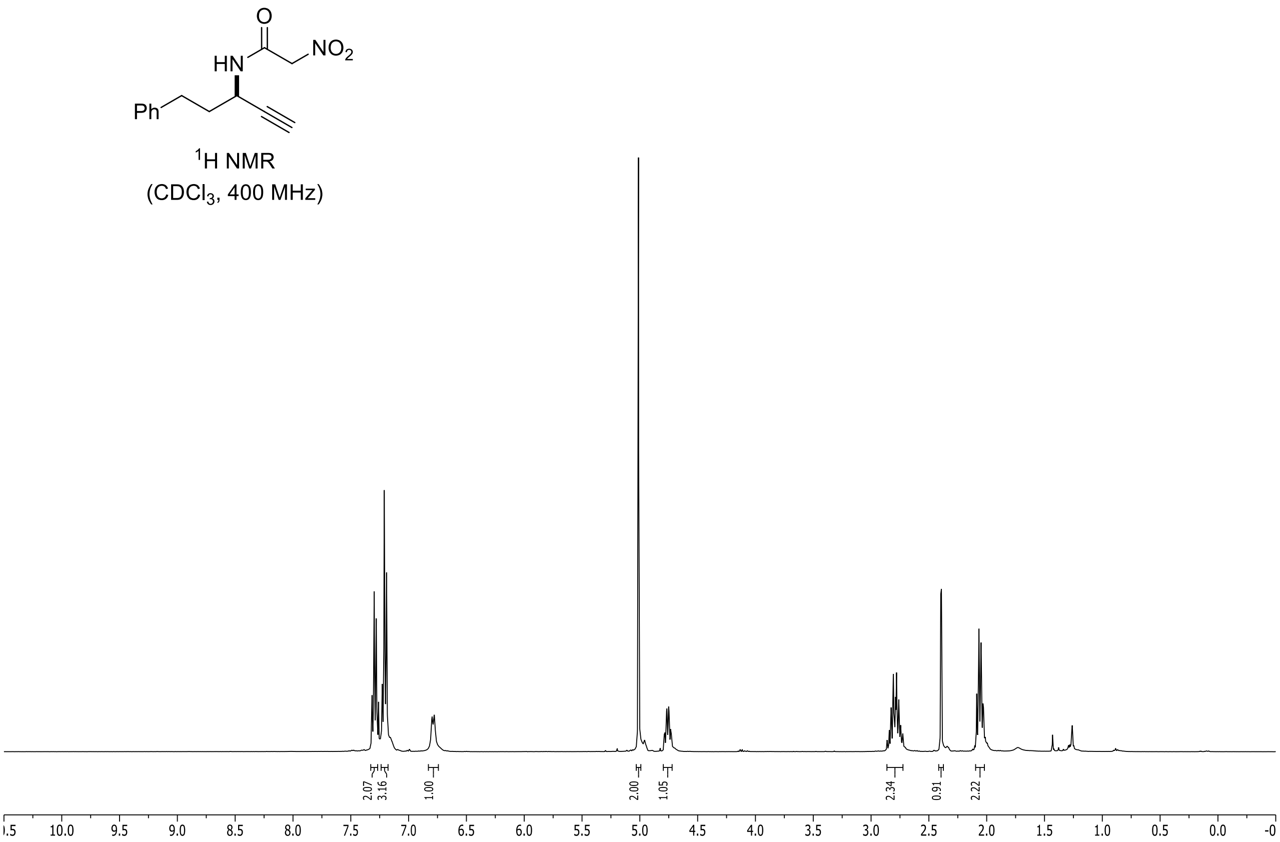

O
HN
NO2
Ph
1H NMR
(CDCl3, 400 MHz)
2.07
3.16
1.00
2.00
1.05
2.34
0.91
2.22
10.0
9.5
9.0
8.5
8.0
7.5
7.0
6.5
6.0
5.5
5.0
4.5
4.0
3.5
3.0
2.5
2.0
1.5
1.0
0.5
0.0

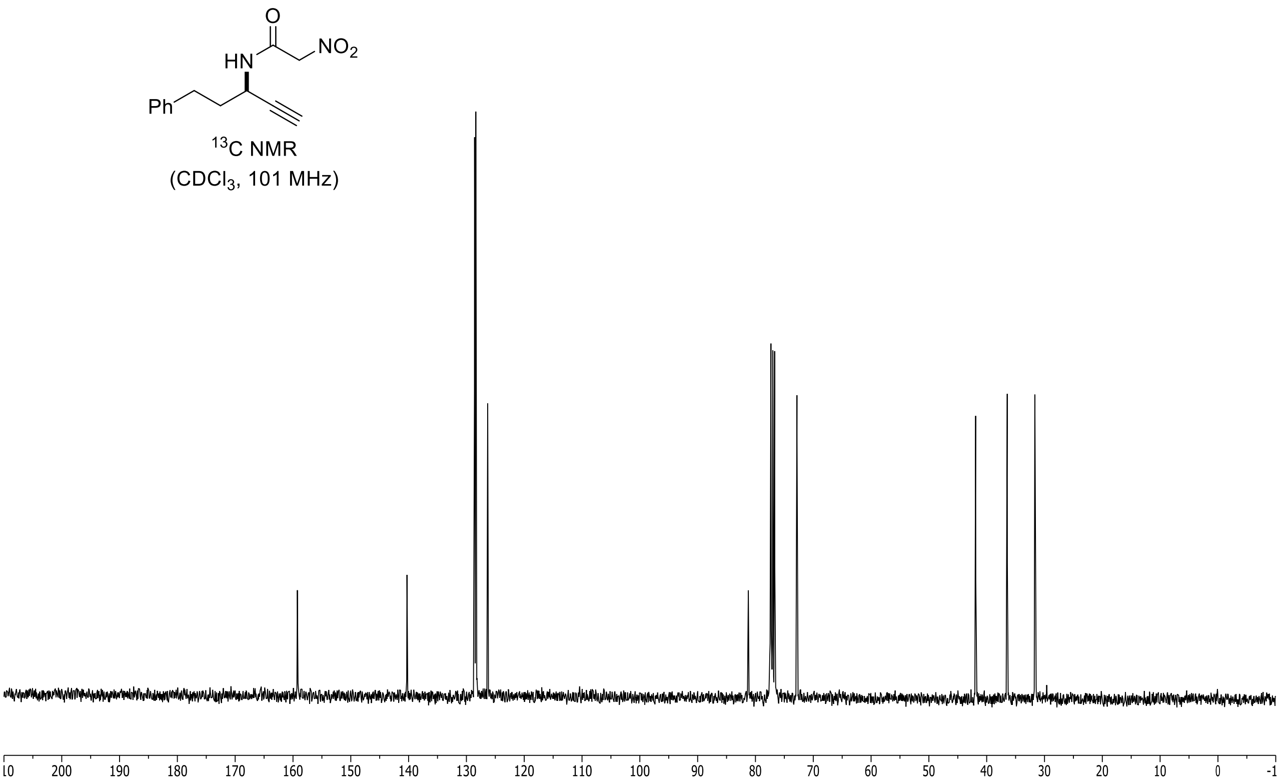

O
HN
NO2
Ph
13C NMR
(CDCl3, 101 MHz)
200
190
180
170
160
150
140
130
120
110
100
90
80
70
60
50
40
30
20
10
0

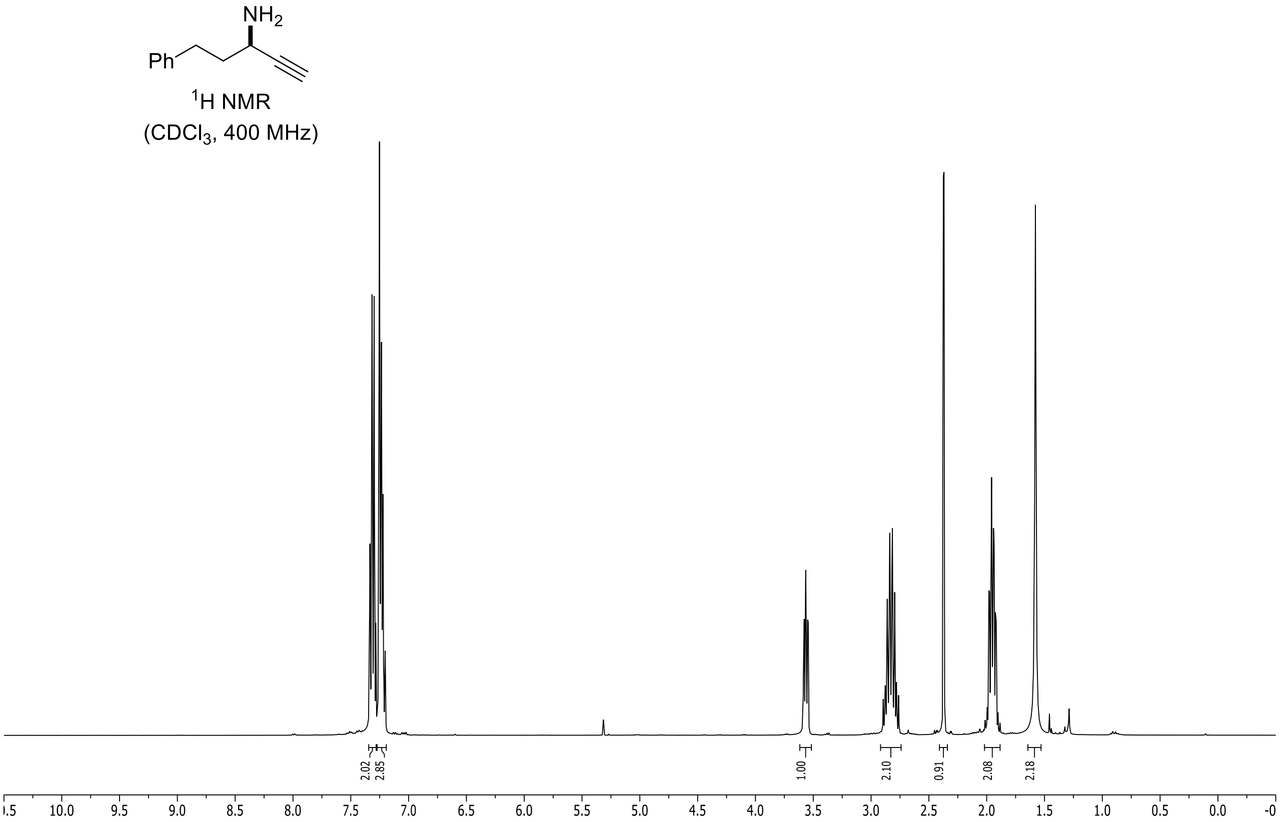
NH2
Ph
1H NMR
(CDCl3, 400 MHz)
2.02
2.85
1.00
2.10
0.91
2.08
2.18
10.0
9.5
9.0
8.5
8.0
7.5
7.0
6.5
6.0
5.5
5.0
4.5
4.0
3.5
3.0
2.5
2.0
1.5
1.0
0.5
0.0

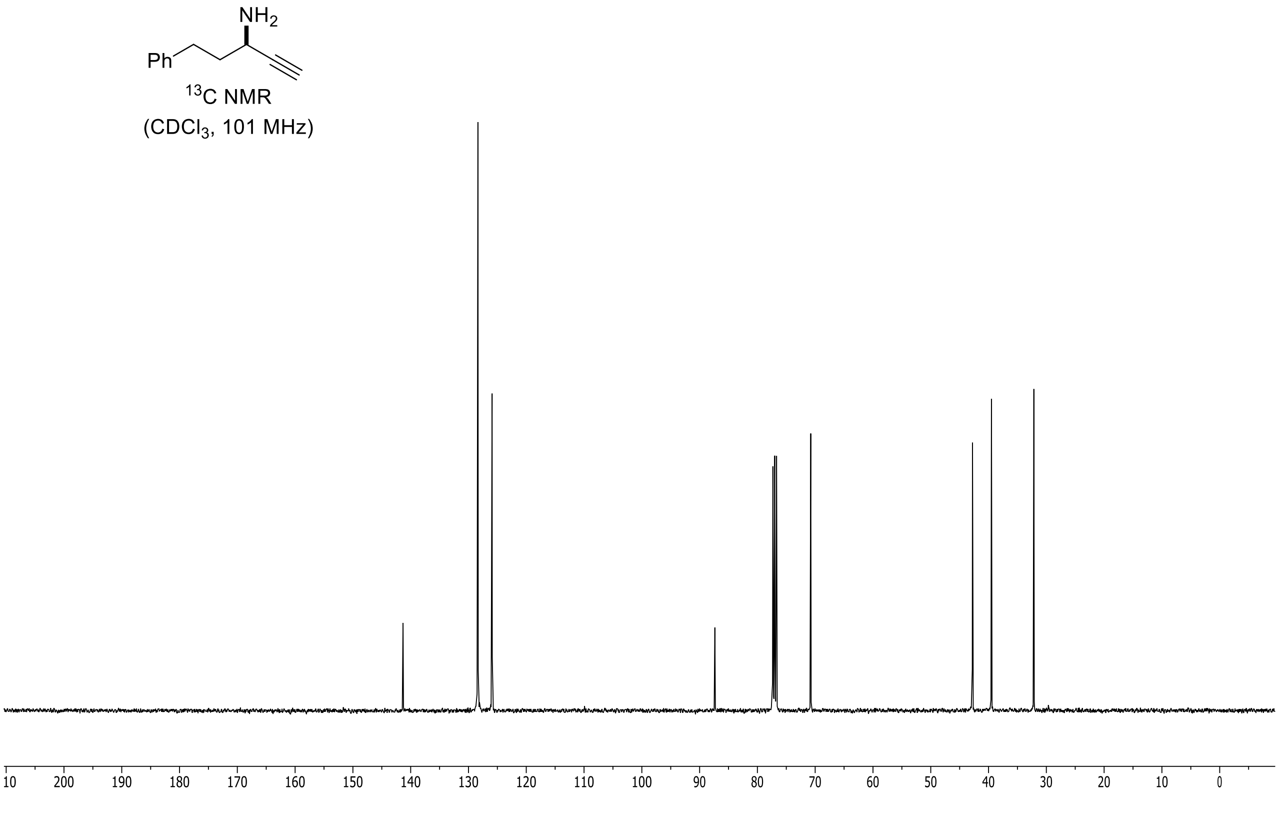
NH2
Ph
13C NMR
(CDCl3, 101 MHz)
200
190
180
170
160
150
140
130
120
110
100
90
80
70
60
50
40
30
20
10
0

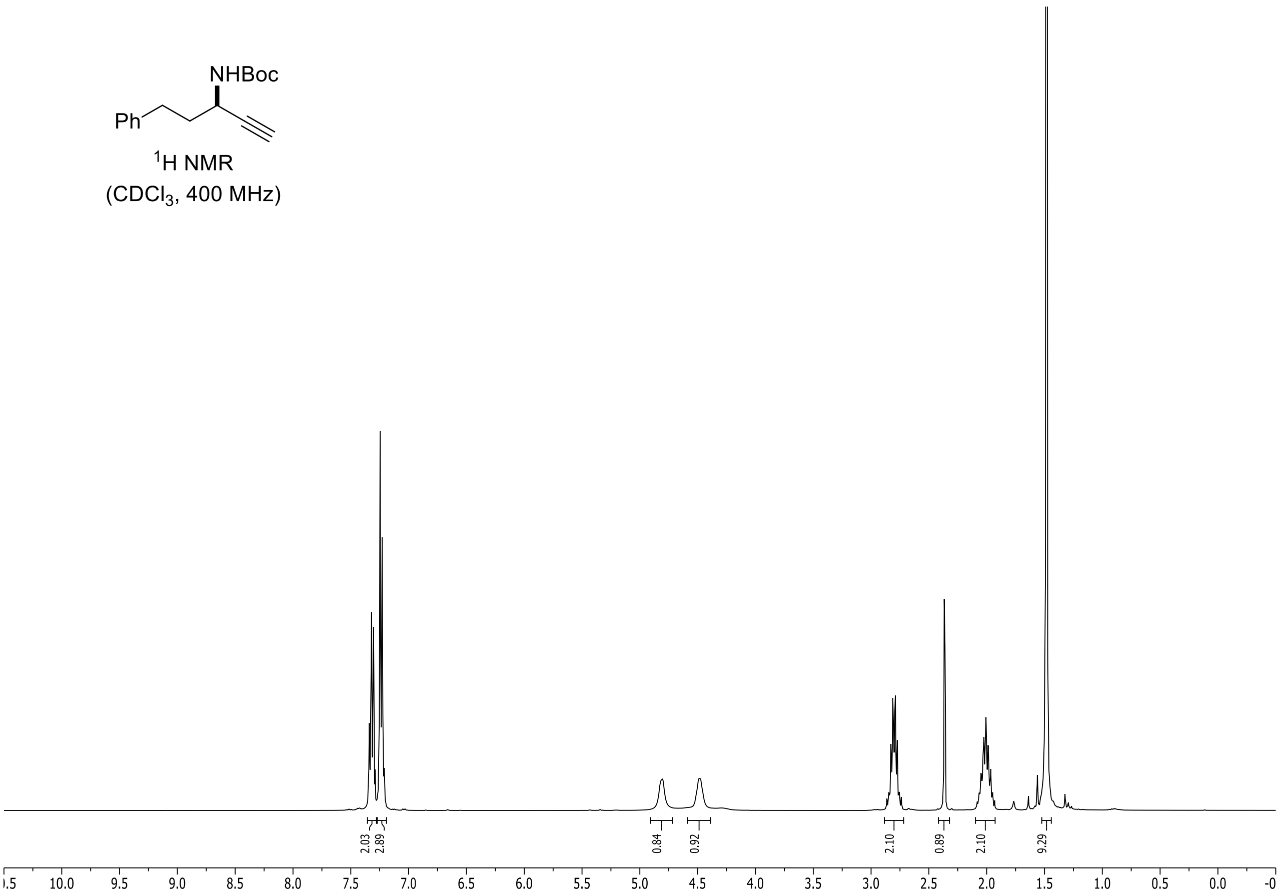
NHBoc
Ph
^{1}H NMR
(CDCl$_3$, 400 MHz)

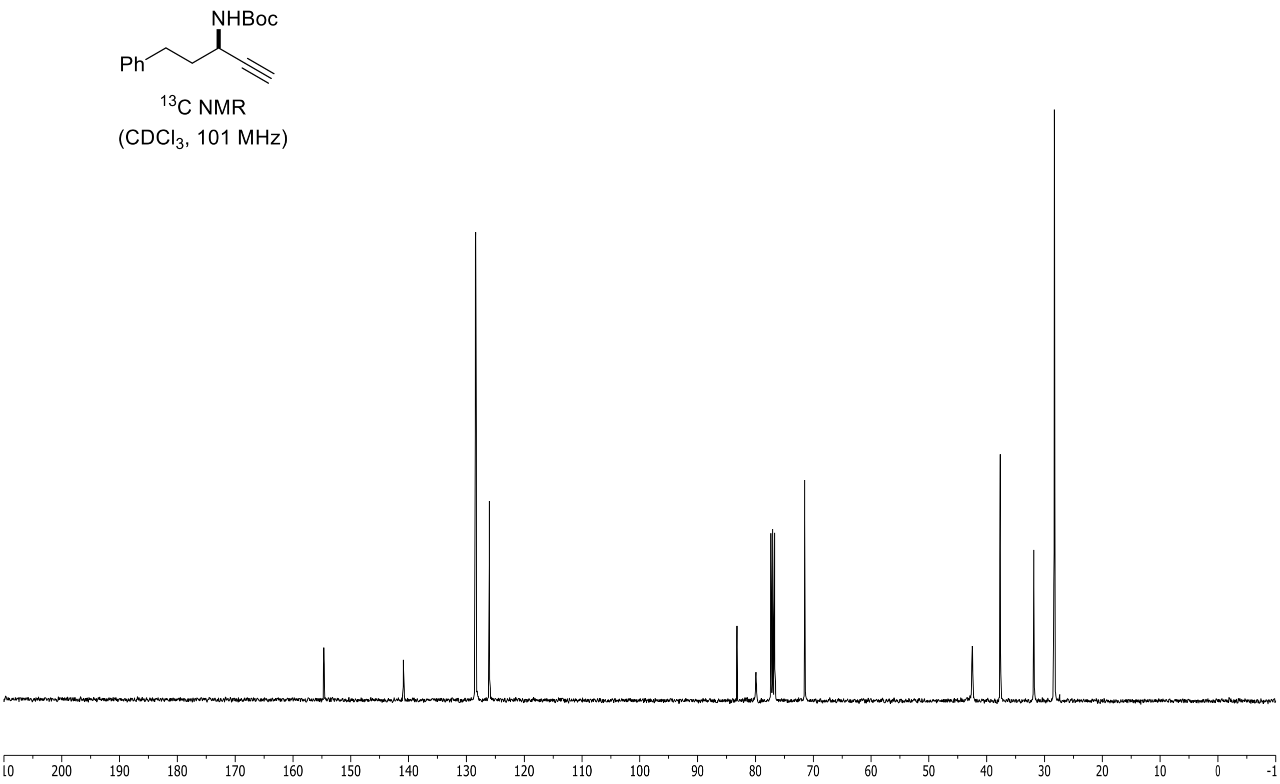
NHBoc
Ph
^{13}C NMR
(CDCl$_3$, 101 MHz)

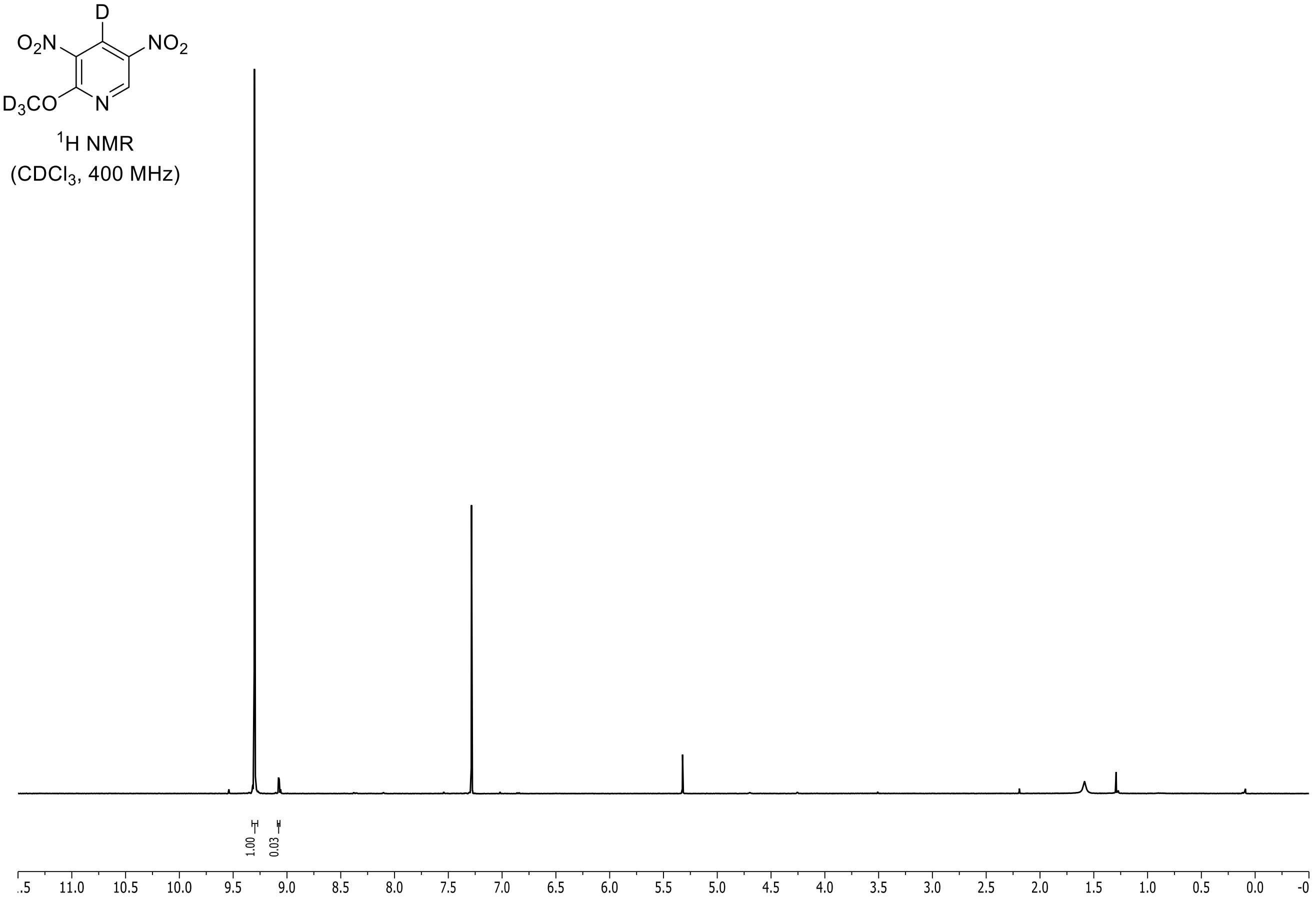
D
O2N
NO2
D3CO
N
1H NMR
(CDCl3, 400 MHz)
1.00
0.03
11.0 10.5 10.0 9.5 9.0 8.5 8.0 7.5 7.0 6.5 6.0 5.5 5.0 4.5 4.0 3.5 3.0 2.5 2.0 1.5 1.0 0.5 0.0

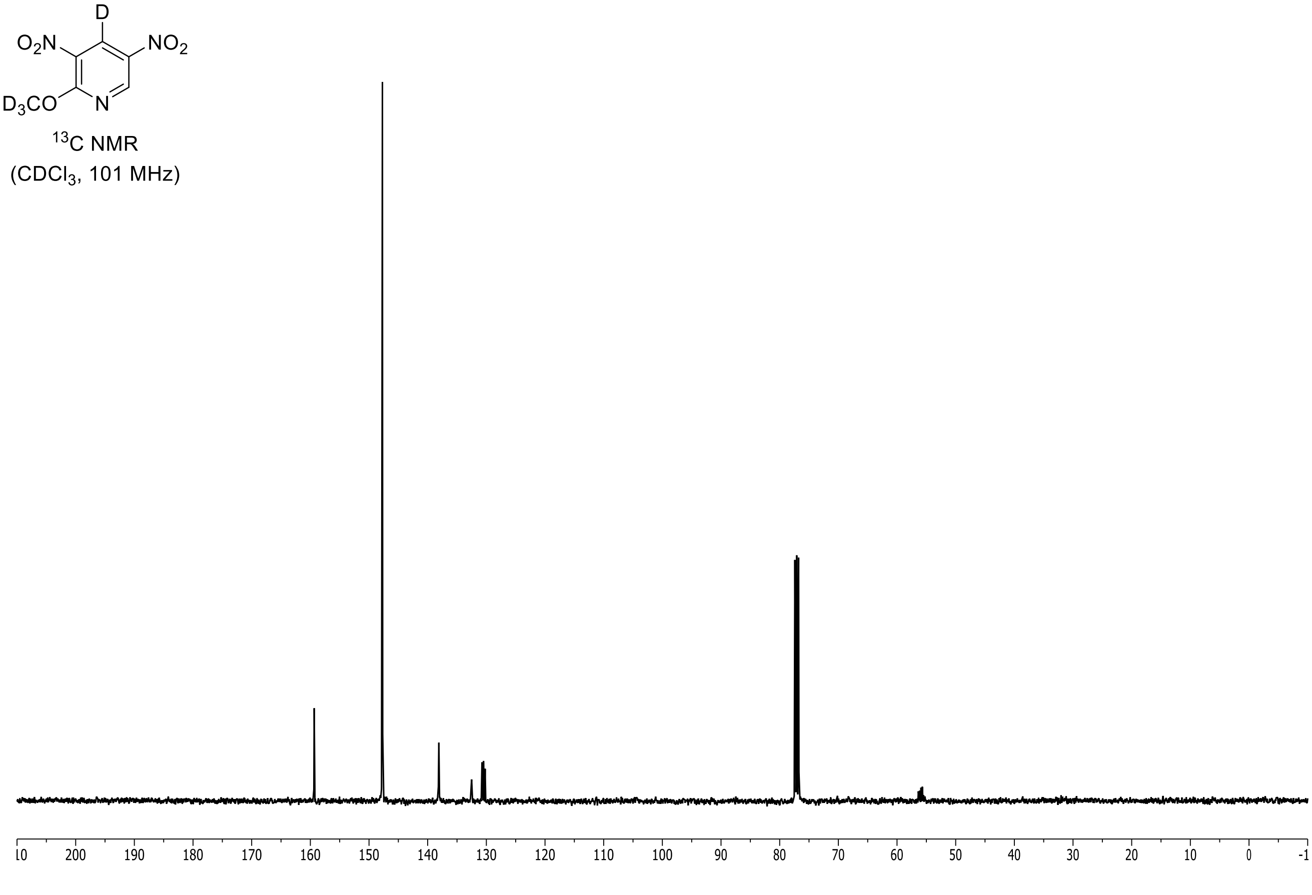
D
O2N
NO2
D3CO
N
13C NMR
(CDCl3, 101 MHz)
200 190 180 170 160 150 140 130 120 110 100 90 80 70 60 50 40 30 20 10 0

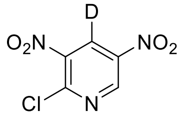

^{1}H NMR
($CDCl_3$, 400 MHz)

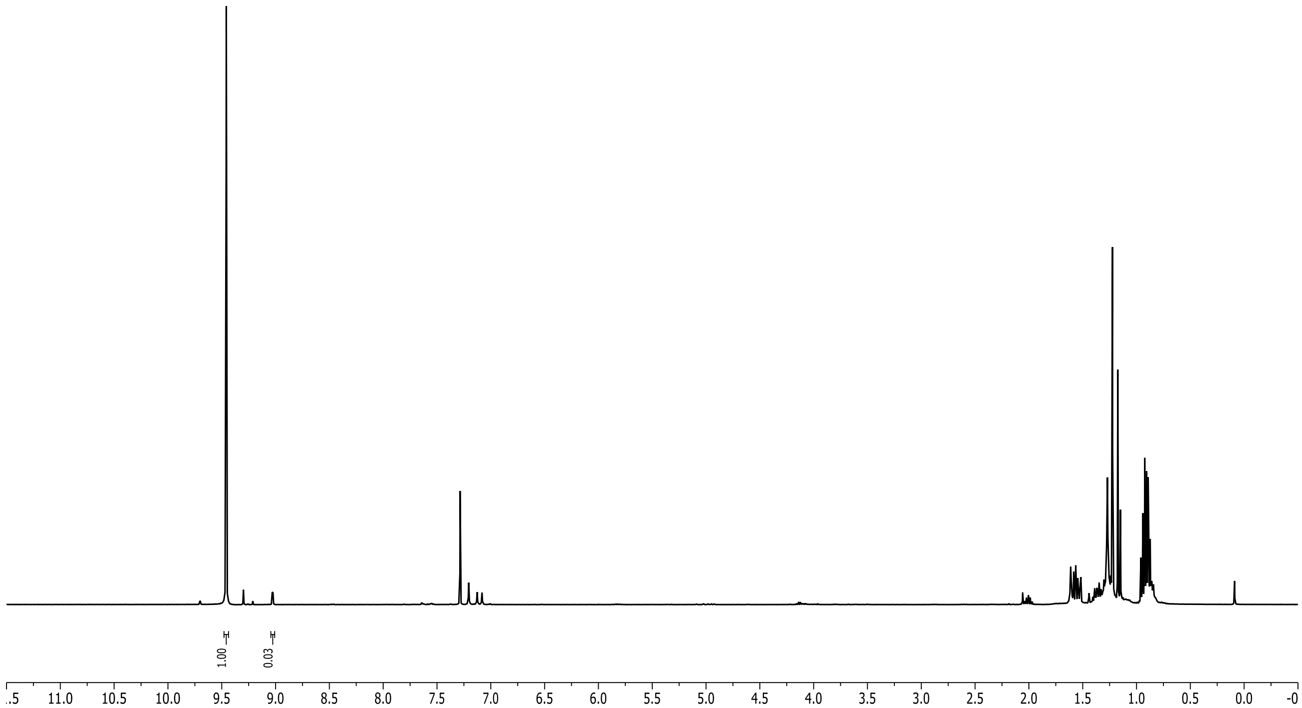

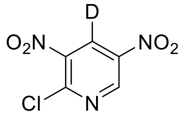

^{13}C NMR
($CDCl_3$, 101 MHz)

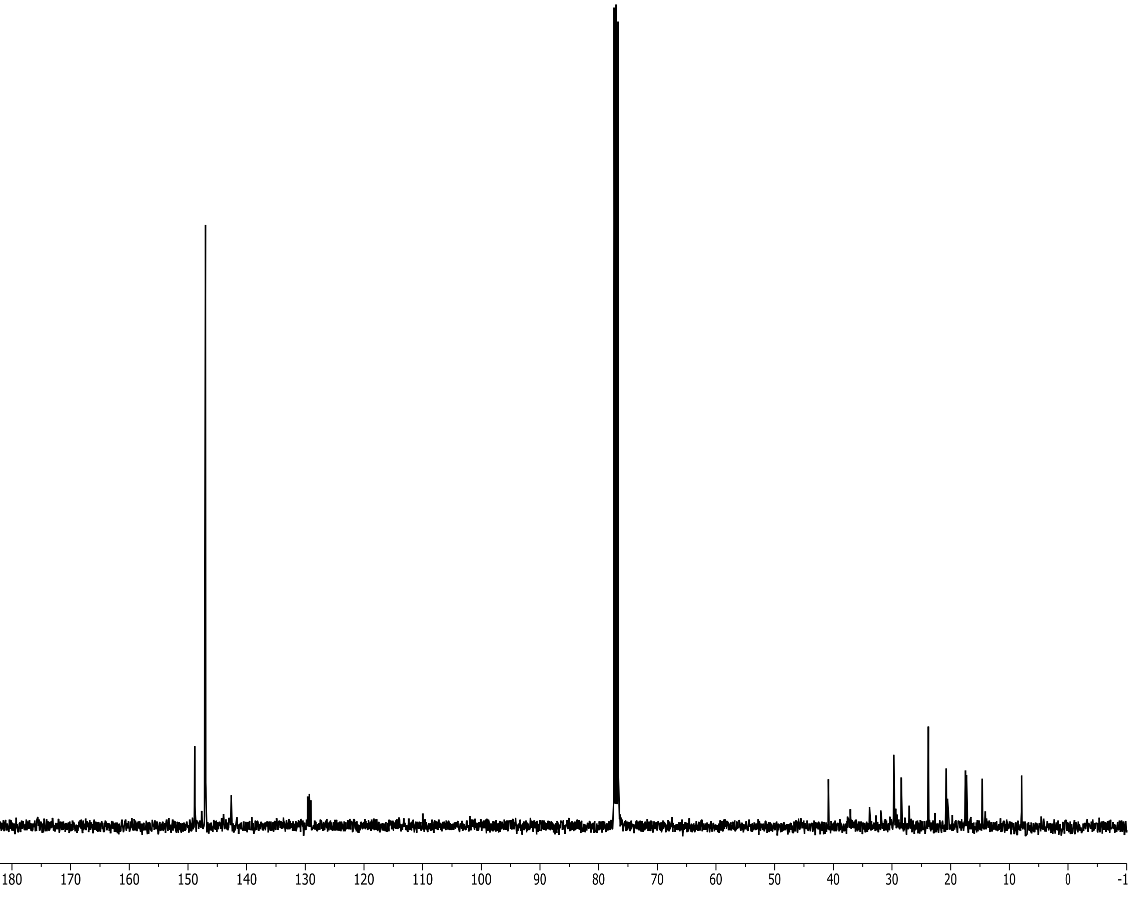

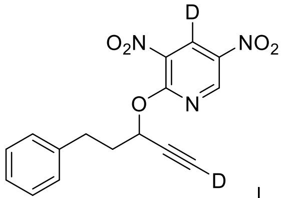

^1H NMR
($CDCl_3$, 400 MHz)

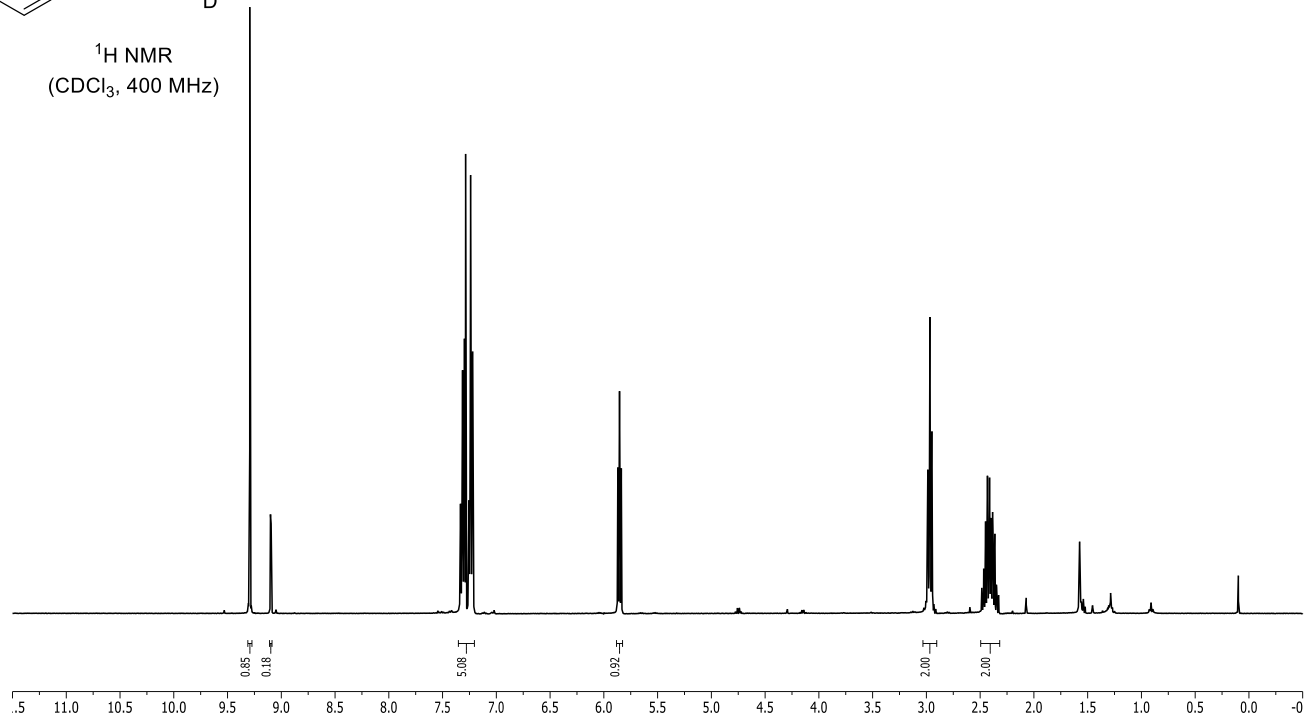

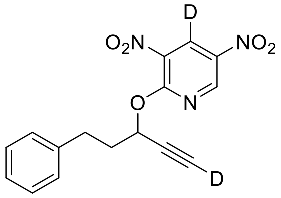

^{13}C NMR
($CDCl_3$, 101 MHz)

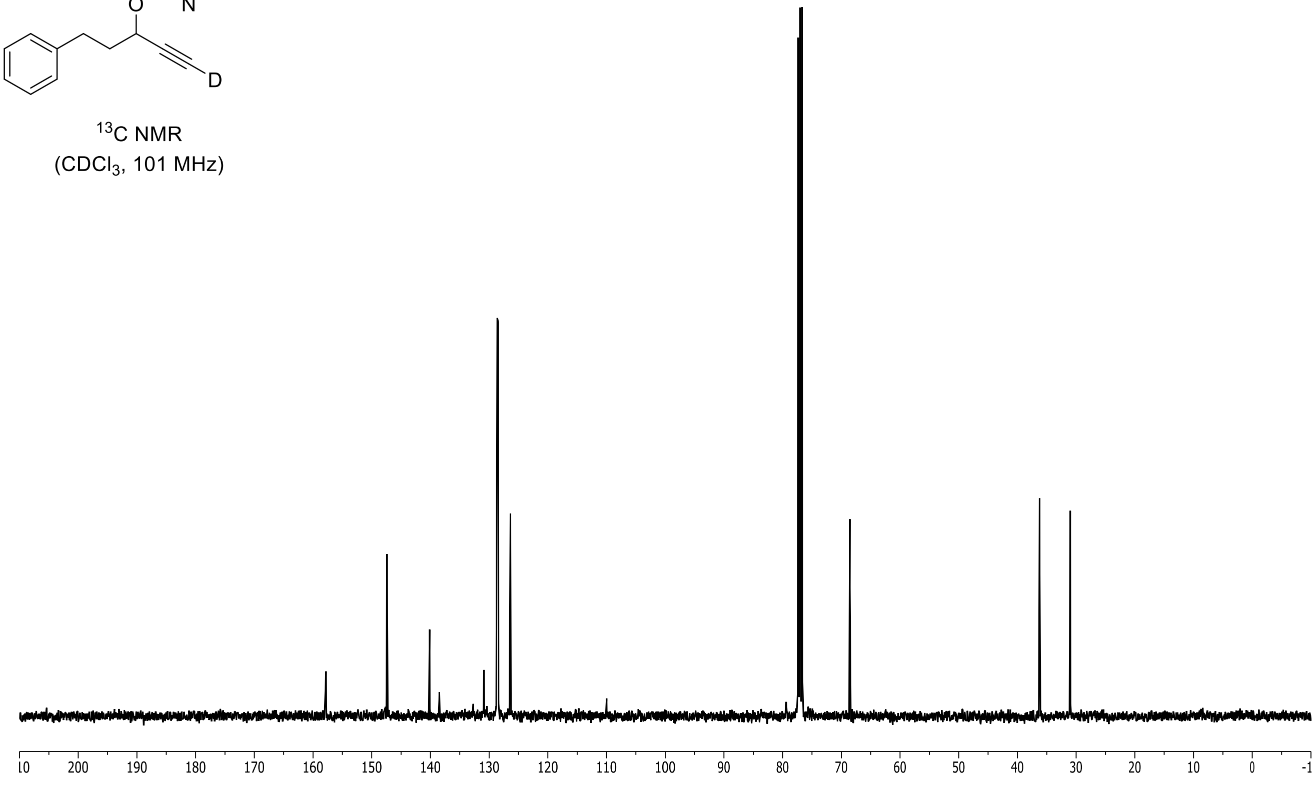

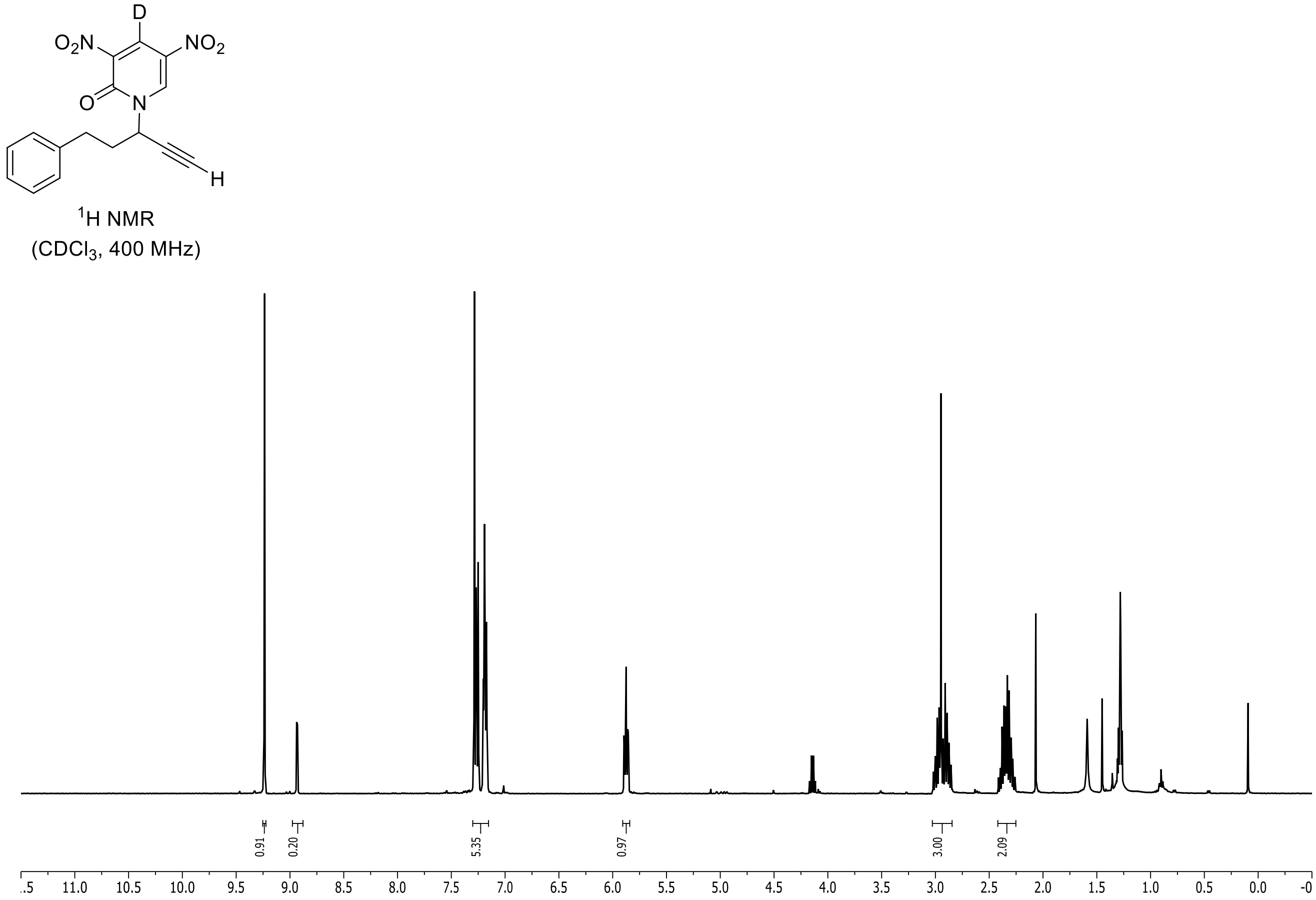
D
O_2N
NO_2
O
N
H
^{1}H NMR
($CDCl_3$, 400 MHz)
0.91
0.20
5.35
0.97
3.00
2.09

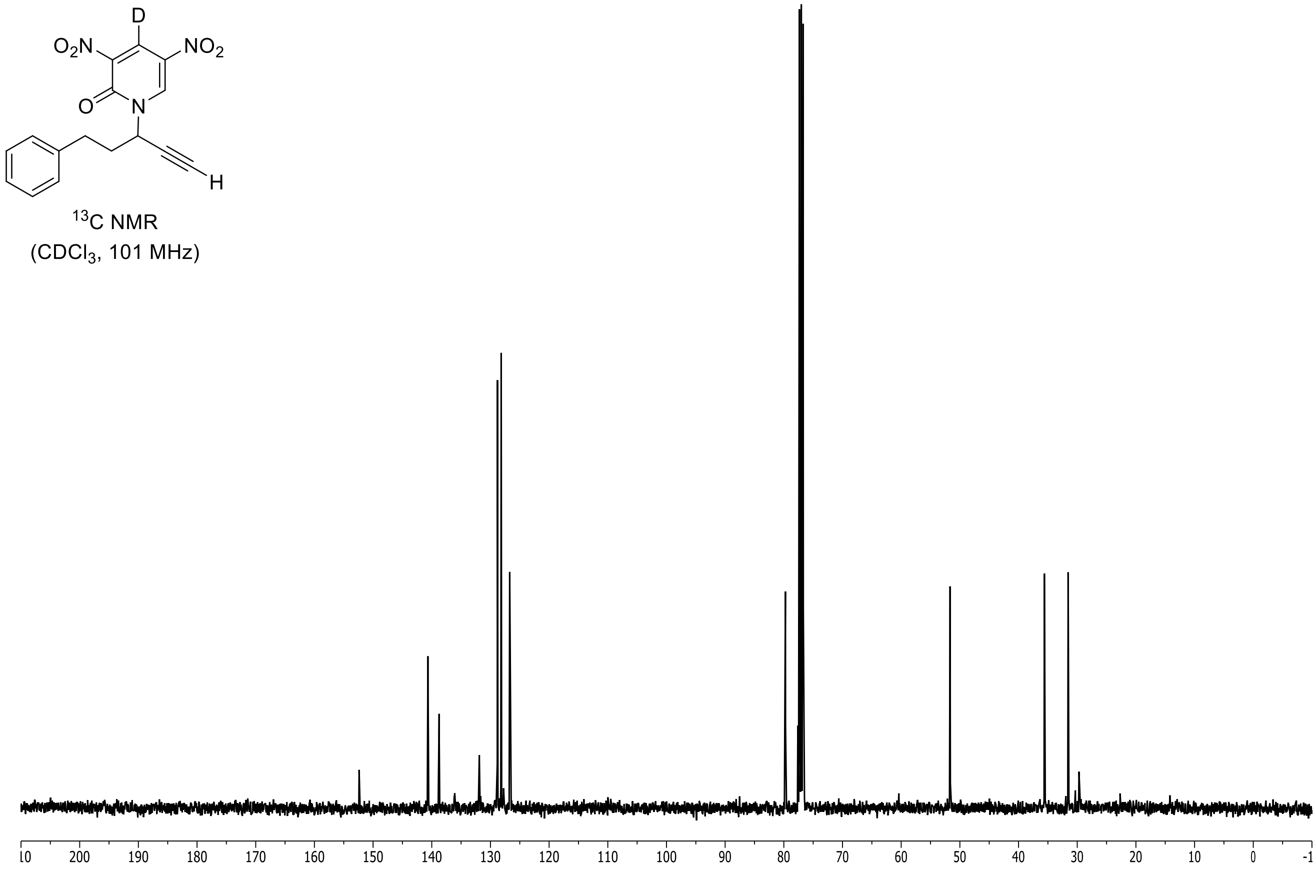
D
O_2N
NO_2
O
N
H
^{13}C NMR
($CDCl_3$, 101 MHz)

9. HPLC Spectra of Enantioenriched Products

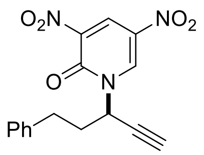

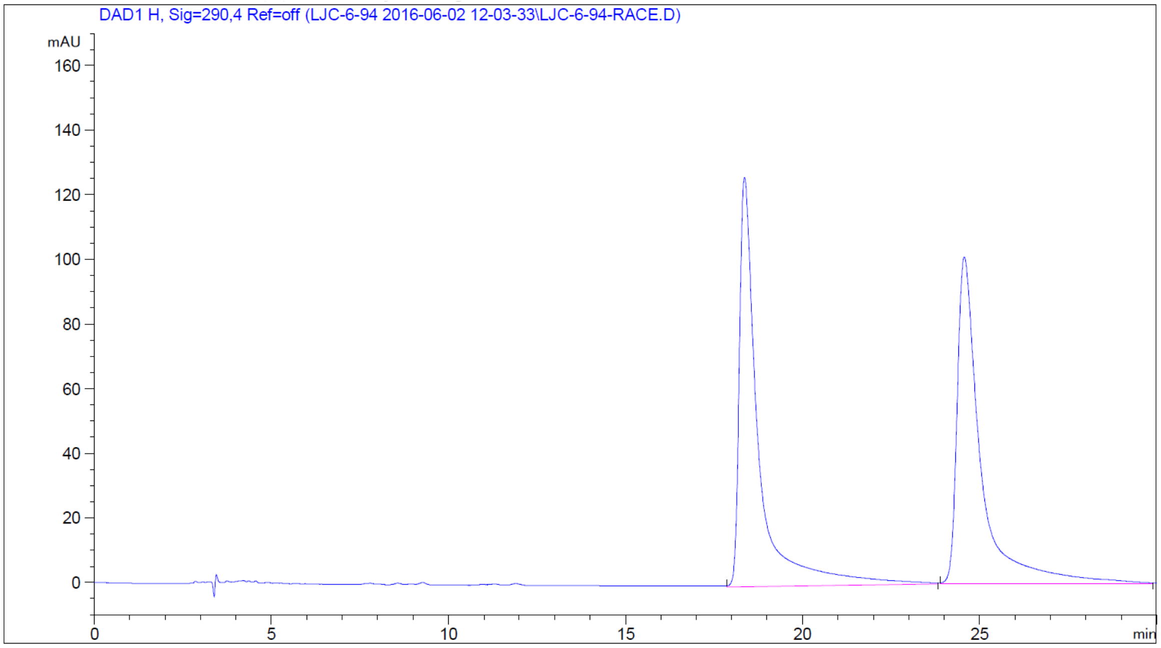

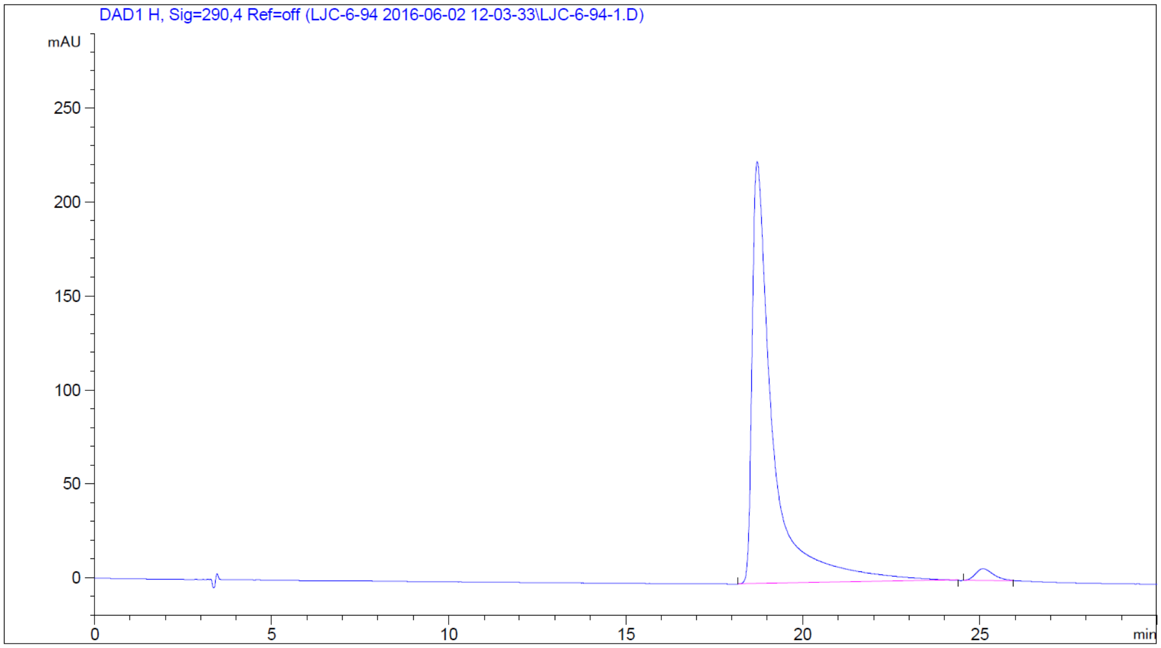

Peak #	RetTime [min]	Type	Width [min]	Area [mAU*s]	Height [mAU]	Area %
1	18.712	MM	0.6754	9105.32617	224.68349	97.6338
2	25.093	BB	0.4978	220.66910	6.25227	2.3662

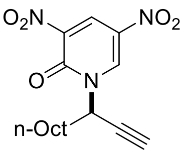

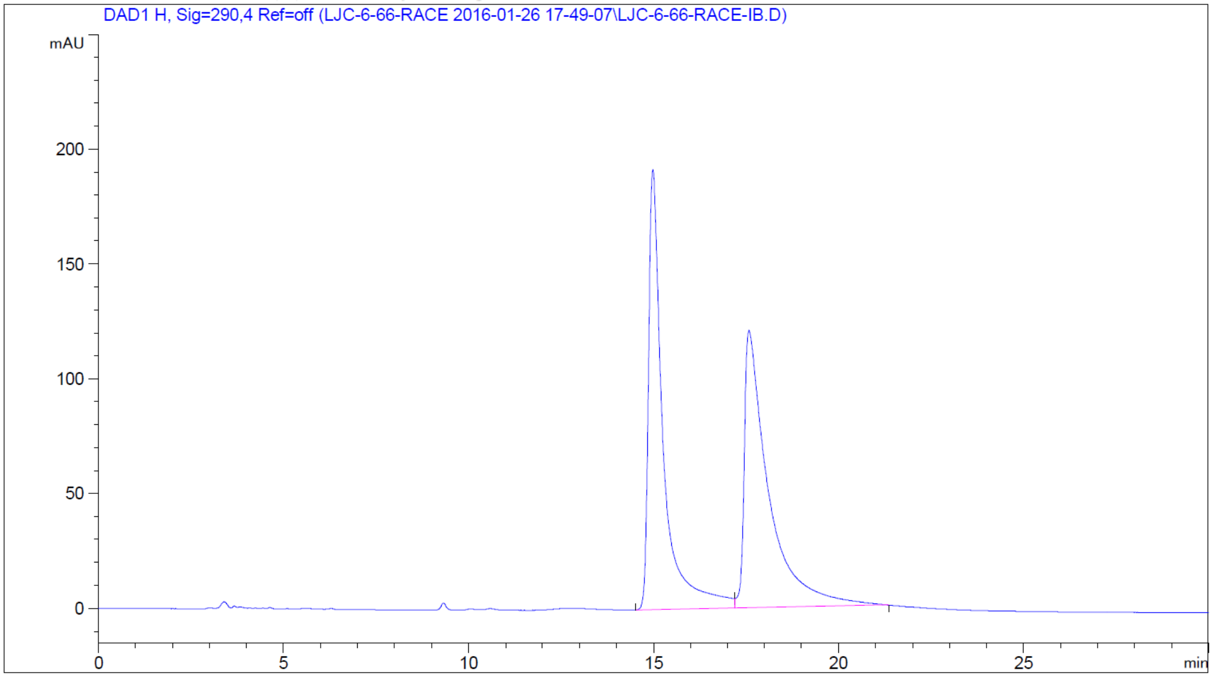

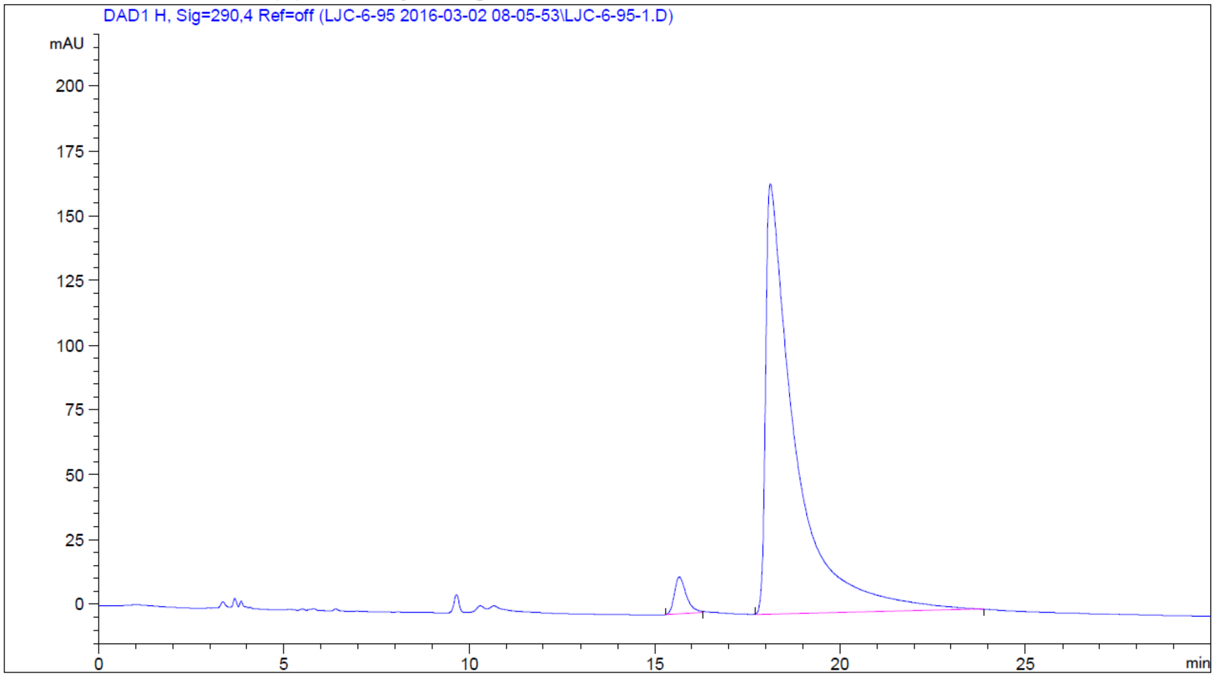

Peak #	RetTime [min]	Type	Width [min]	Area [mAU*s]	Height [mAU]	Area %
1	15.668	BB	0.3375	320.29987	14.28824	3.3718
2	18.124	MM	0.9207	9178.99219	166.16382	96.6282

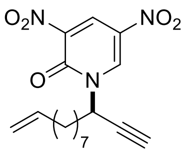

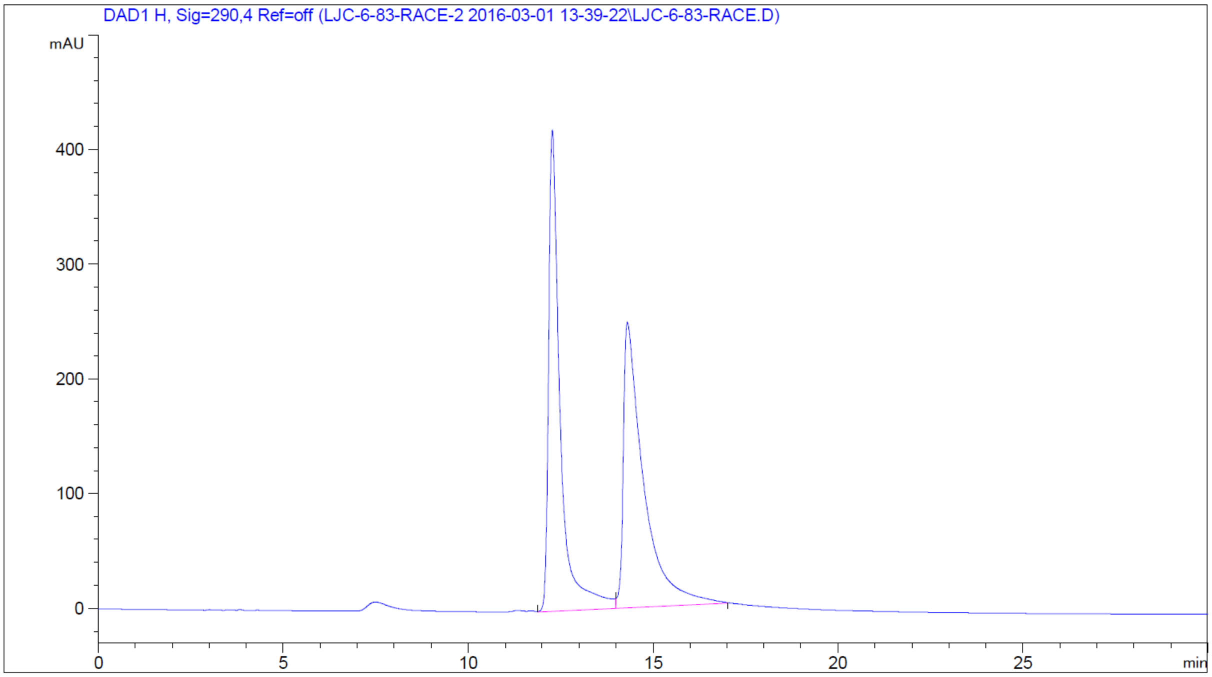

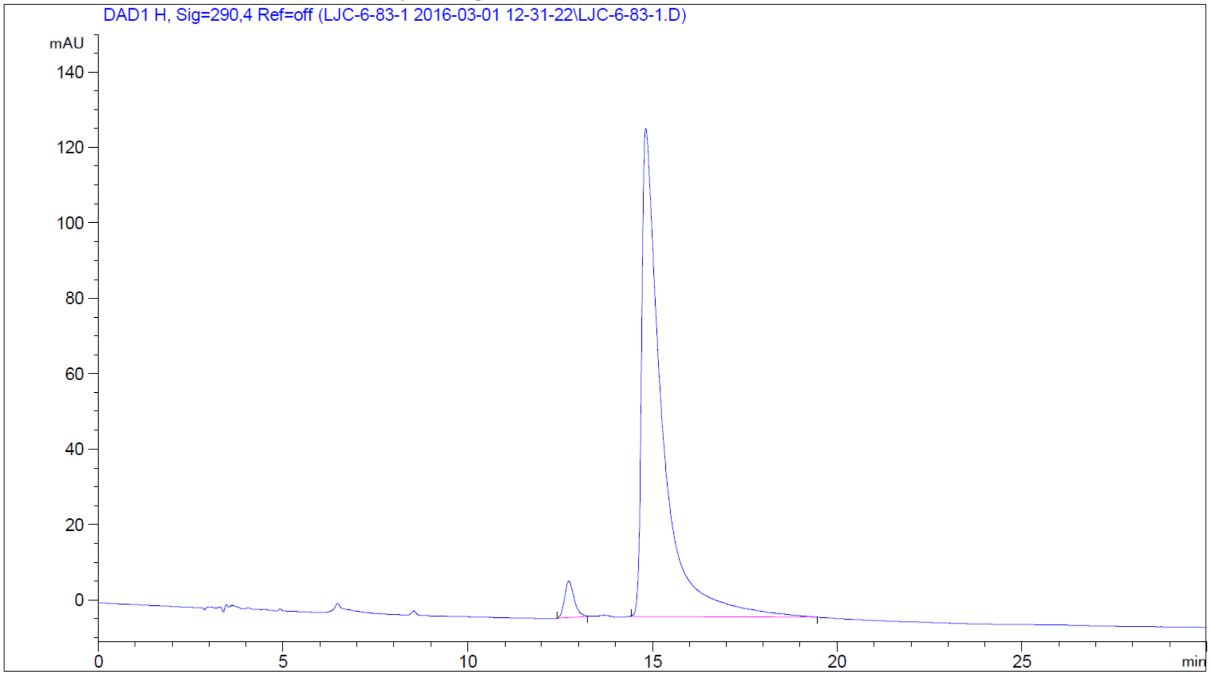

Peak #	RetTime [min]	Type	Width [min]	Area [mAU*s]	Height [mAU]	Area %
1	12.738	BB	0.2755	175.14902	9.66030	3.4183
2	14.823	MM	0.6374	4948.68799	129.39560	96.5817

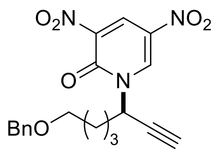

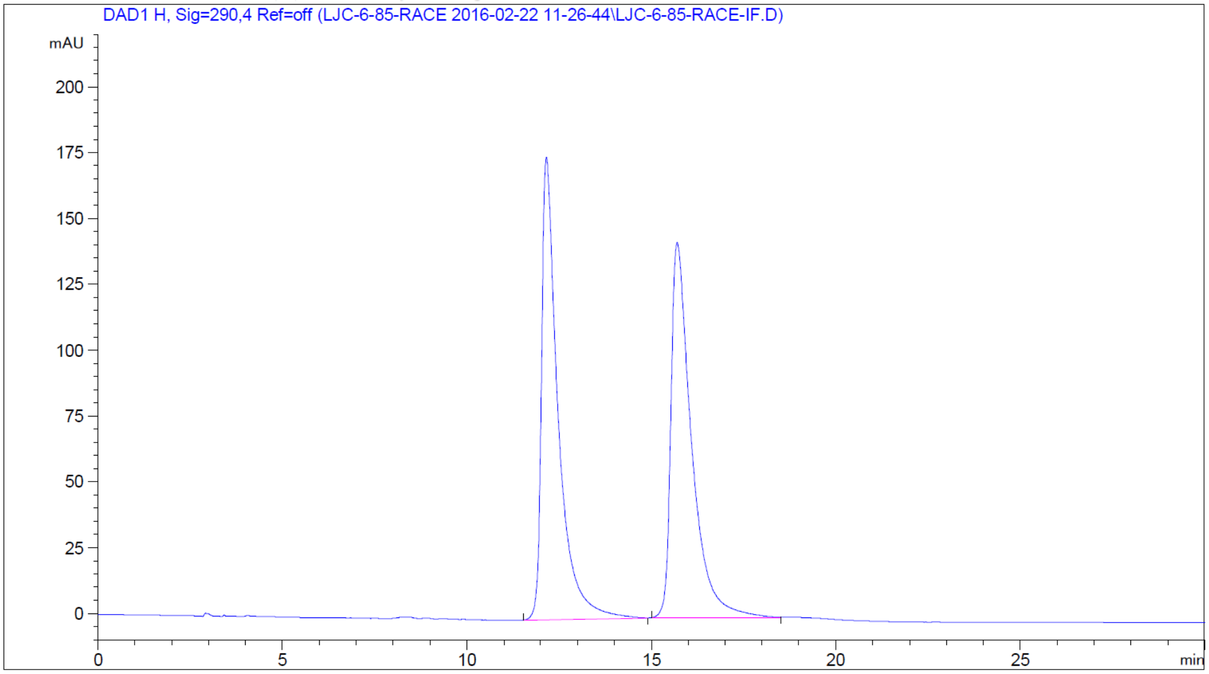

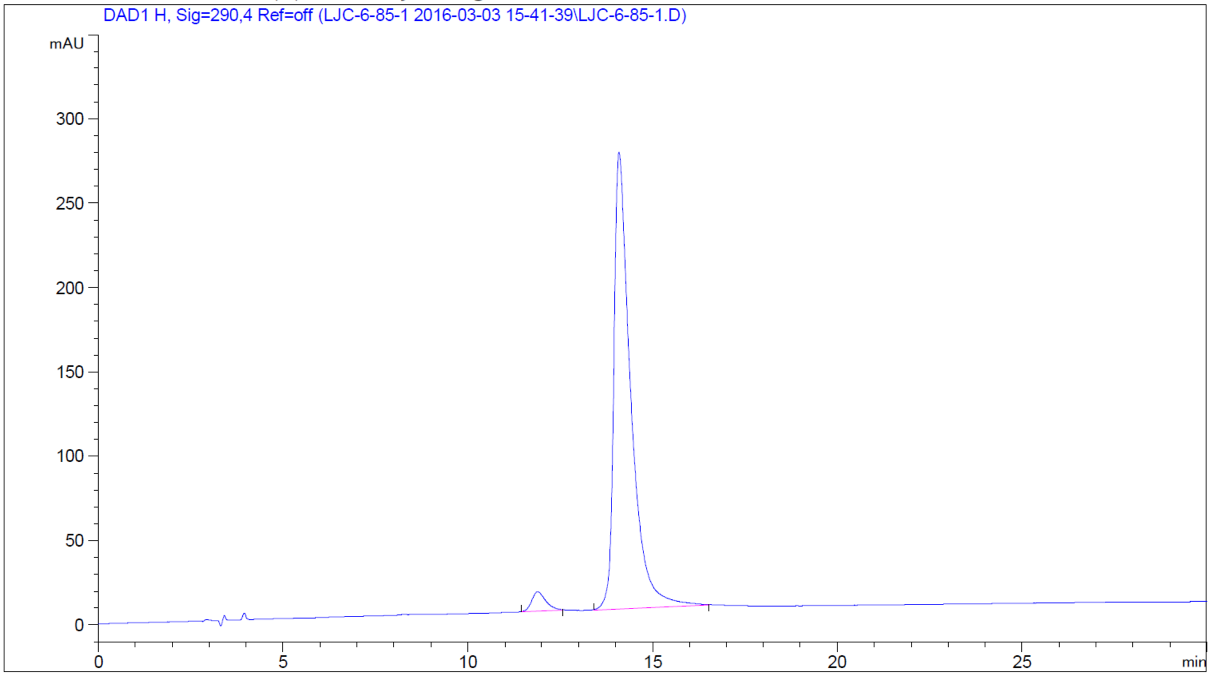

Peak #	RetTime [min]	Type	Width [min]	Area [mAU*s]	Height [mAU]	Area %
1	11.888	BB	0.3974	310.38470	11.55339	3.4810
2	14.090	MM	0.5296	8606.15137	270.84061	96.5190

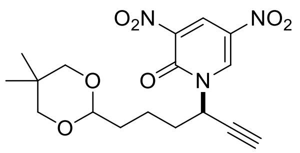

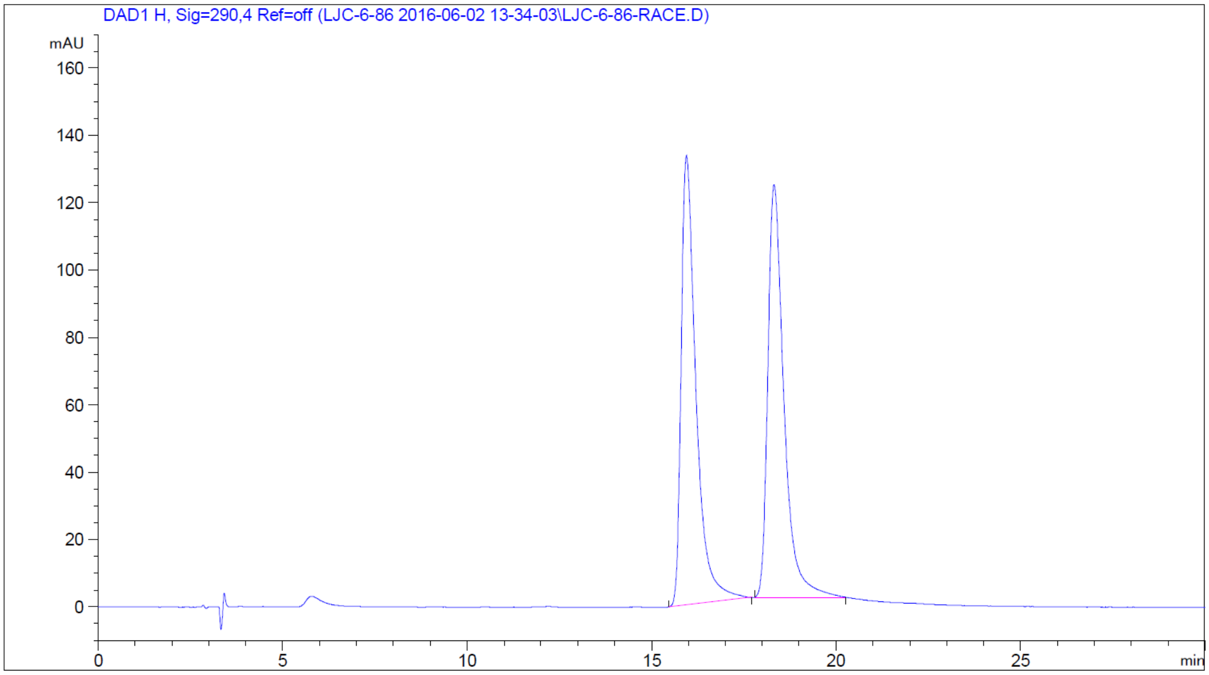

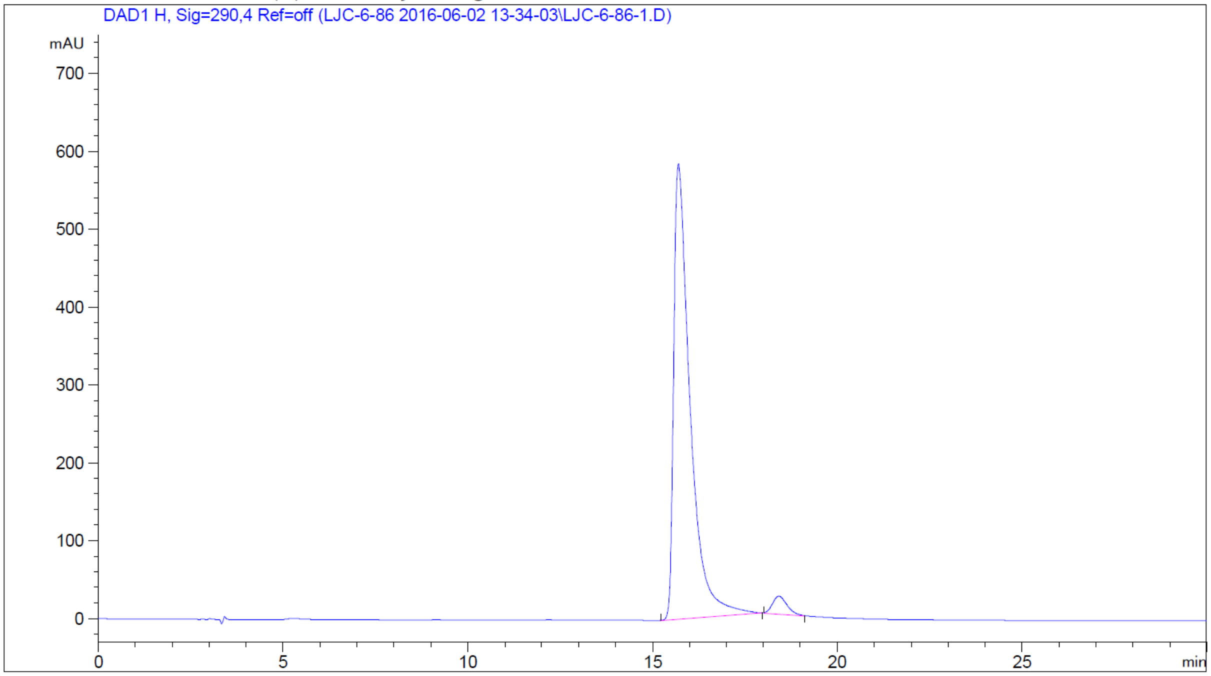

Peak #	RetTime [min]	Type	Width [min]	Area [mAU*s]	Height [mAU]	Area %
1	15.704	MM	0.5124	1.79852e4	585.02887	96.5972
2	18.423	MM	0.4561	633.56268	23.15052	3.4028

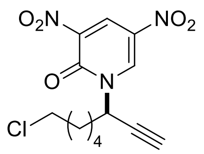

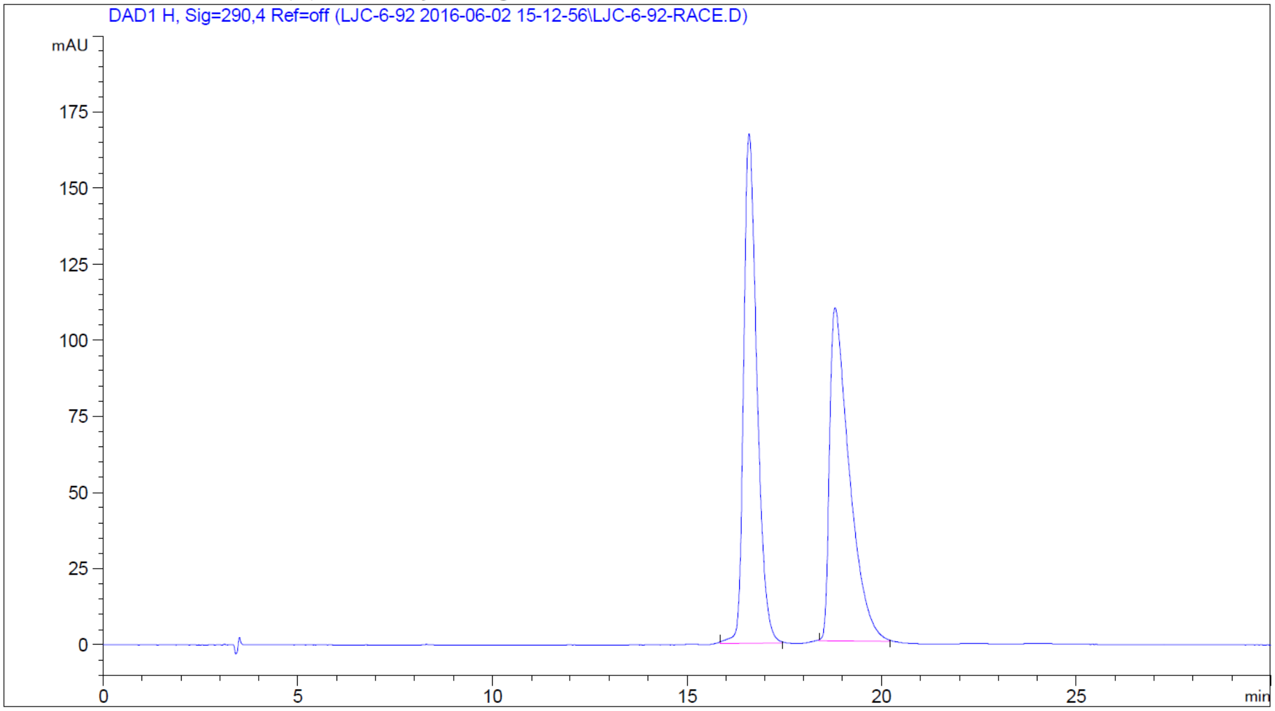

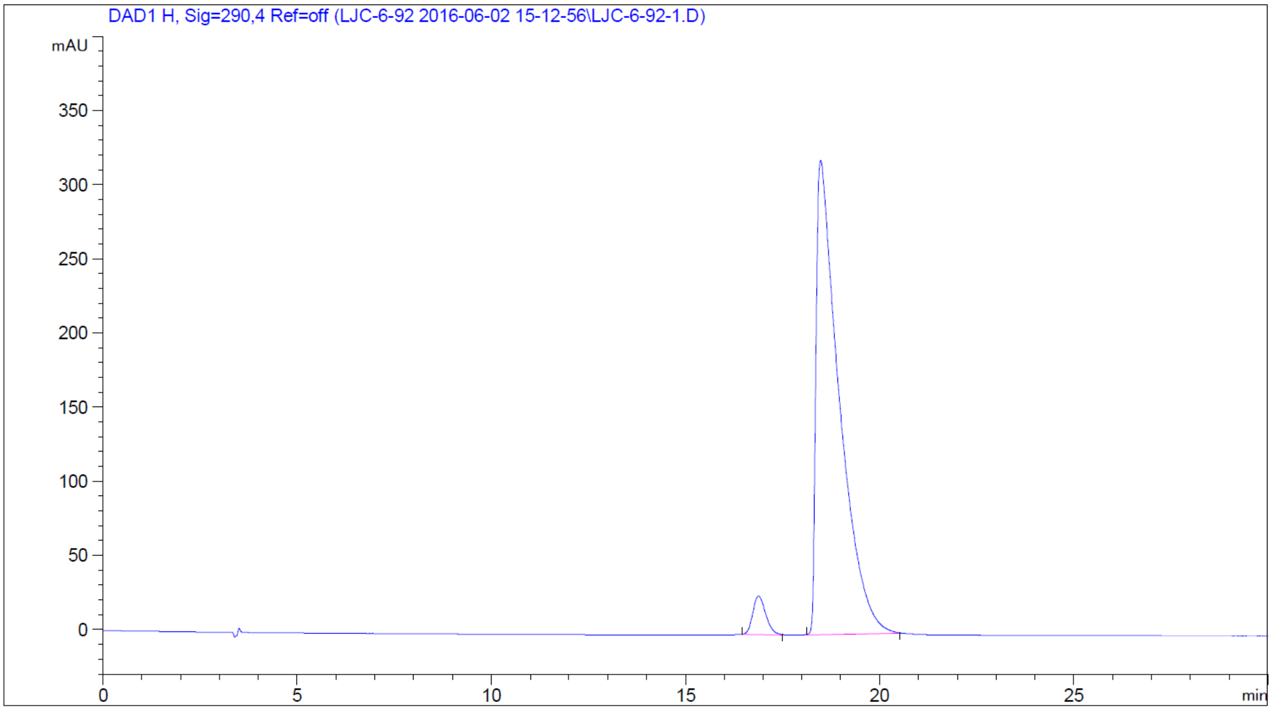

Peak #	RetTime [min]	Type	Width [min]	Area [mAU*s]	Height [mAU]	Area %
1	16.878	BB	0.3559	603.80280	26.09848	4.3605
2	18.479	BB	0.5815	1.32434e4	319.55338	95.6395

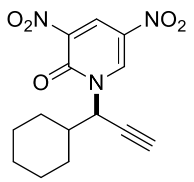

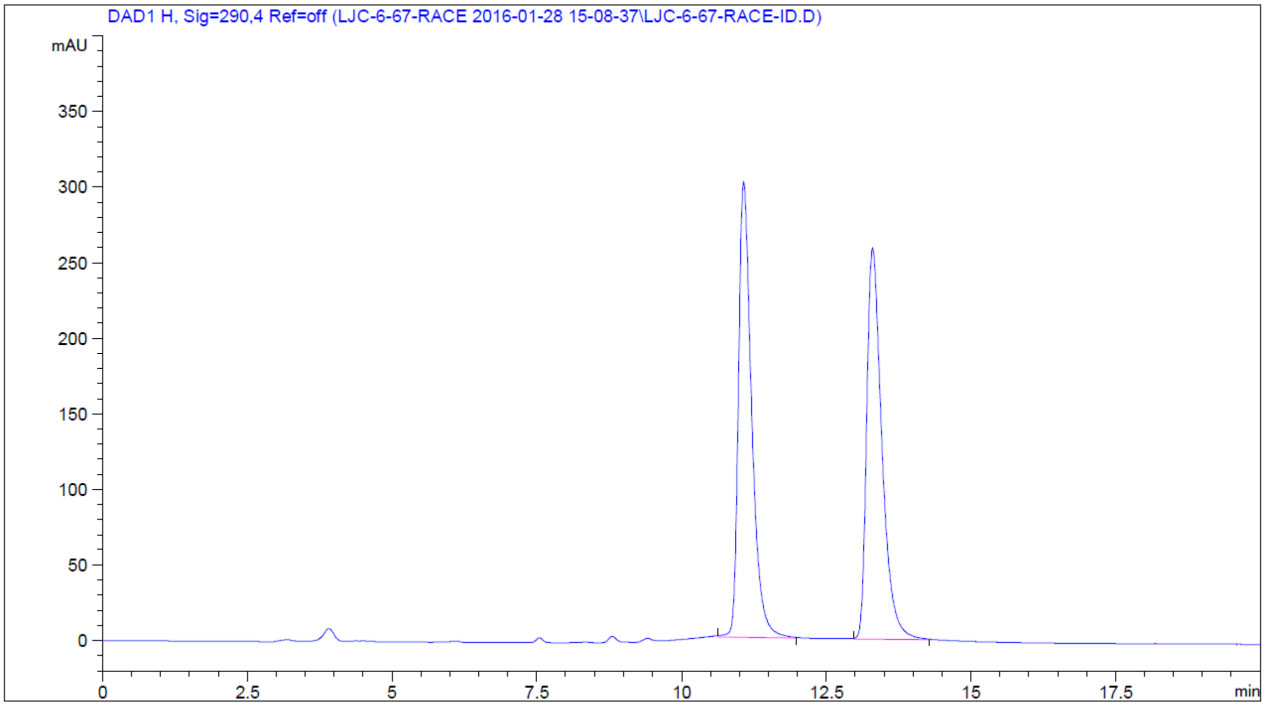

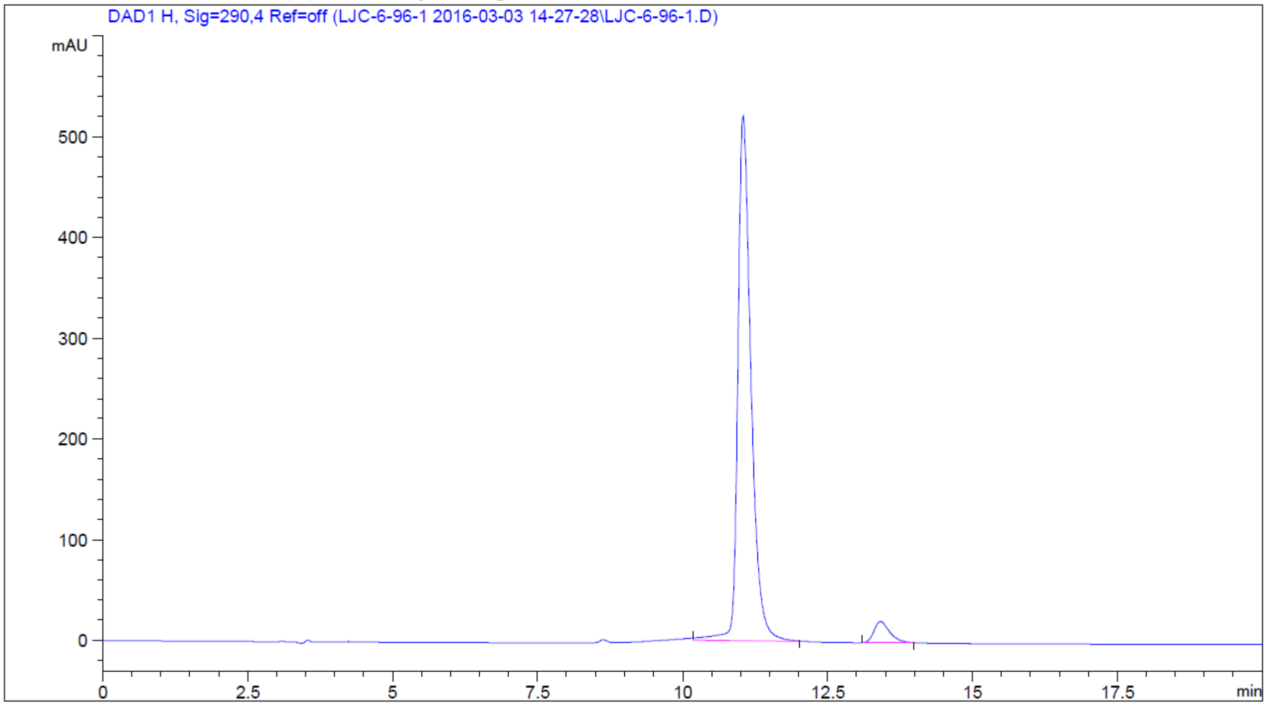

Peak #	RetTime [min]	Type	Width [min]	Area [mAU*s]	Height [mAU]	Area %
1	11.046	BB	0.2391	8268.77539	521.13336	95.3246
2	13.416	BB	0.2916	405.55820	21.16108	4.6754

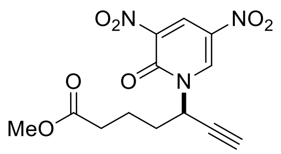

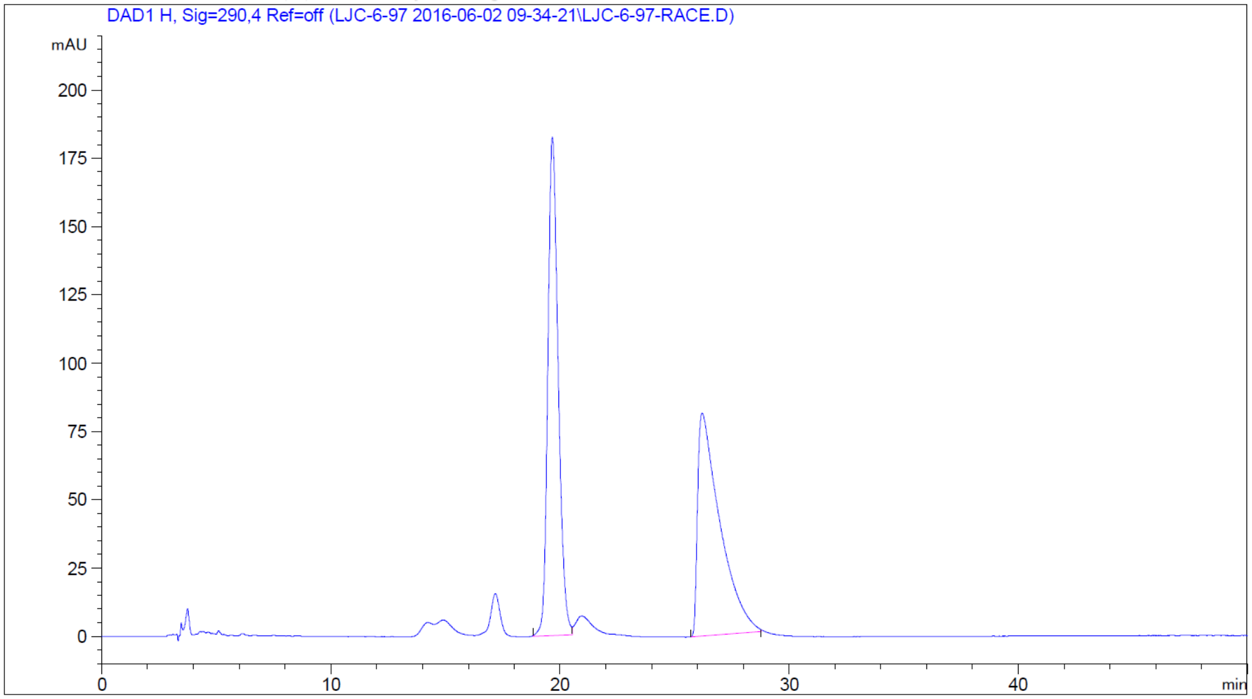

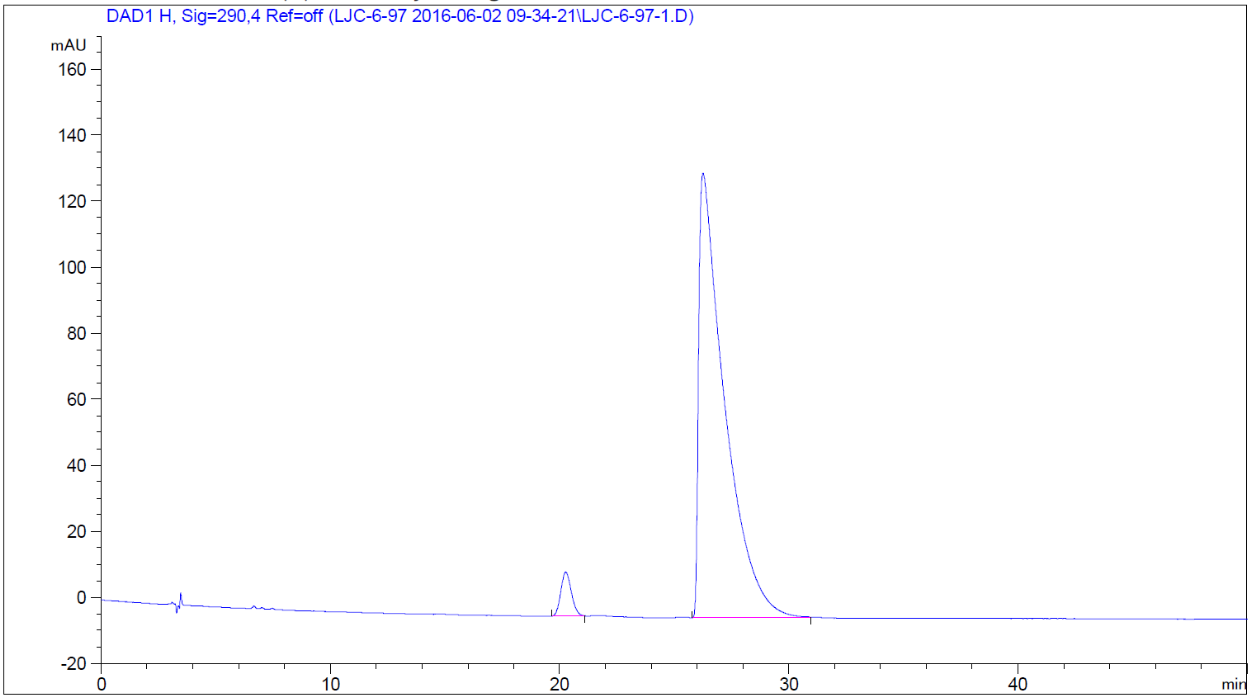

Peak #	RetTime [min]	Type	Width [min]	Area [mAU*s]	Height [mAU]	Area %
1	20.271	BB	0.4927	428.12589	13.33152	3.9025
2	26.261	MM	1.3047	1.05423e4	134.66667	96.0975

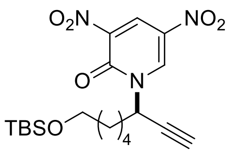

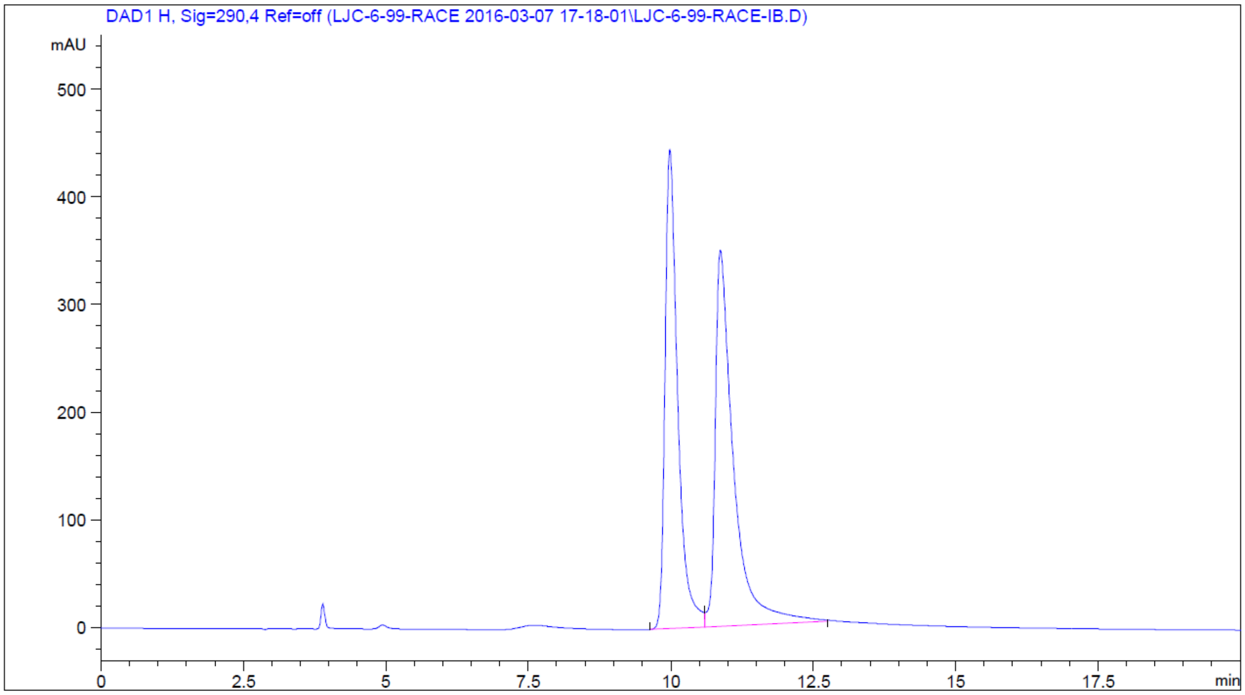

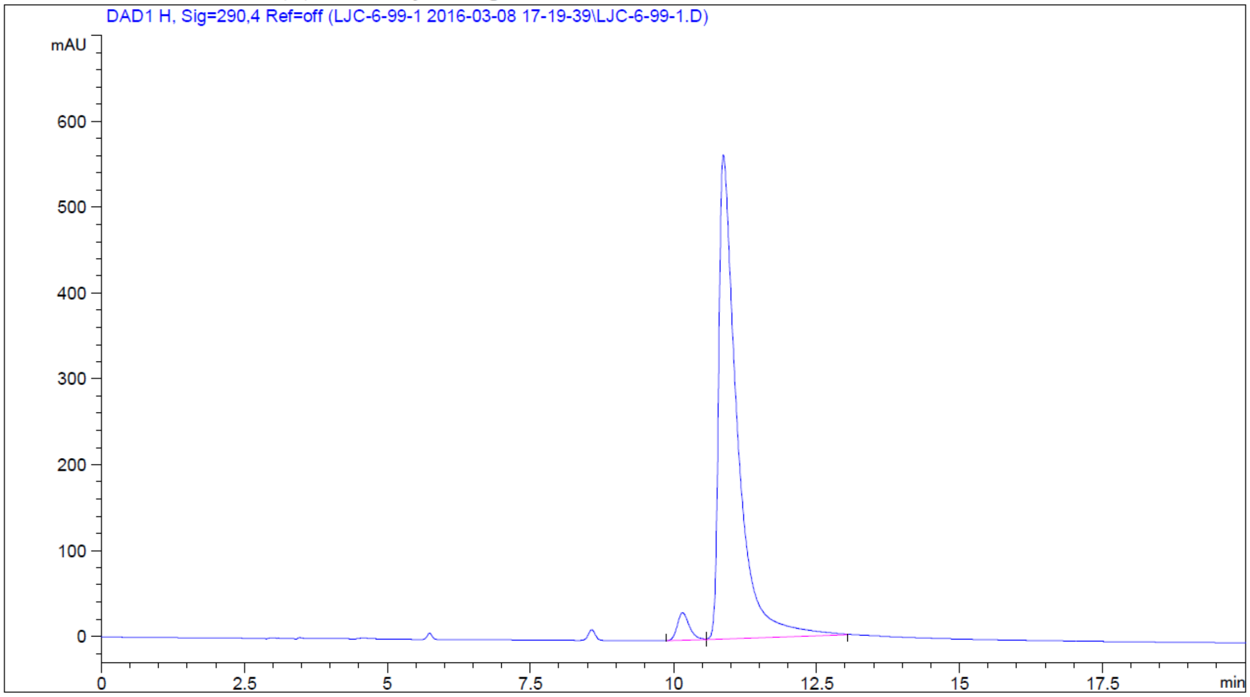

Peak #	RetTime [min]	Type	Width [min]	Area [mAU*s]	Height [mAU]	Area %
1	10.159	BV	0.2199	467.92670	32.13675	3.5688
2	10.873	VB	0.3194	1.26438e4	564.27075	96.4312

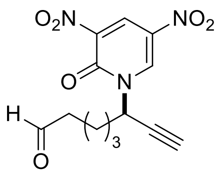

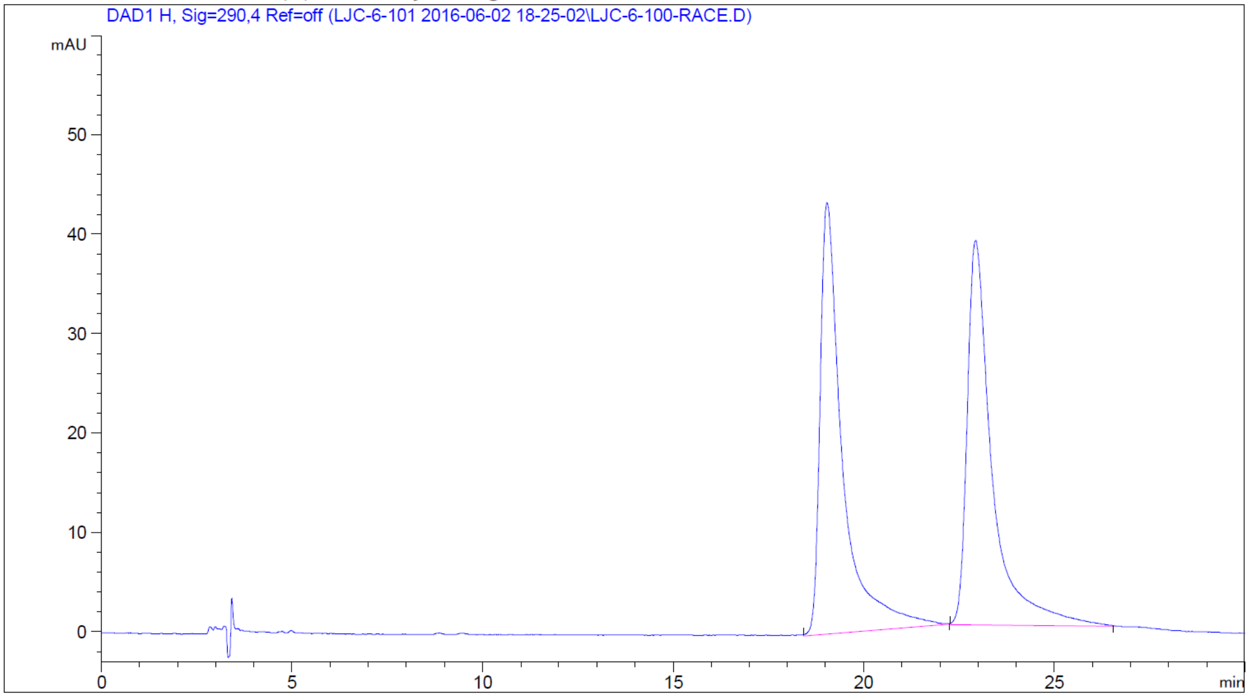

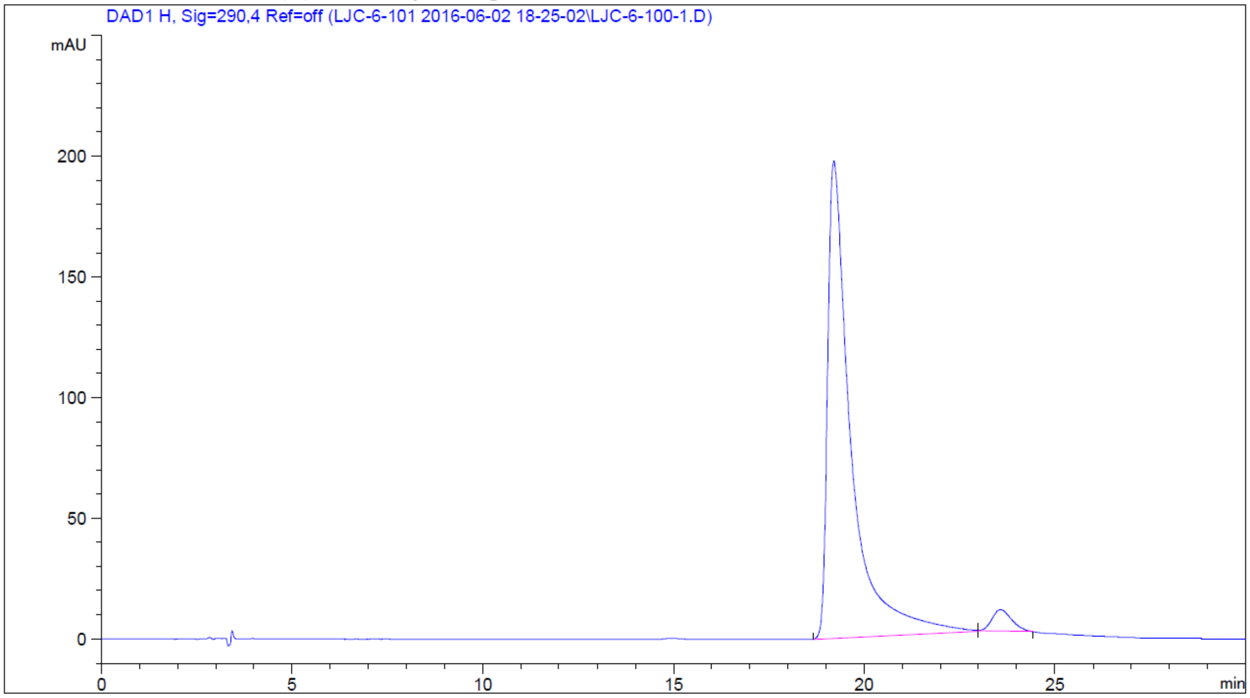

Peak #	RetTime [min]	Type	Width [min]	Area [mAU*s]	Height [mAU]	Area %
1	19.208	MM	0.7121	8452.66895	197.84544	96.3730
2	23.584	MM	0.5963	318.11768	8.89184	3.6270

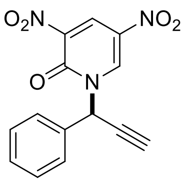

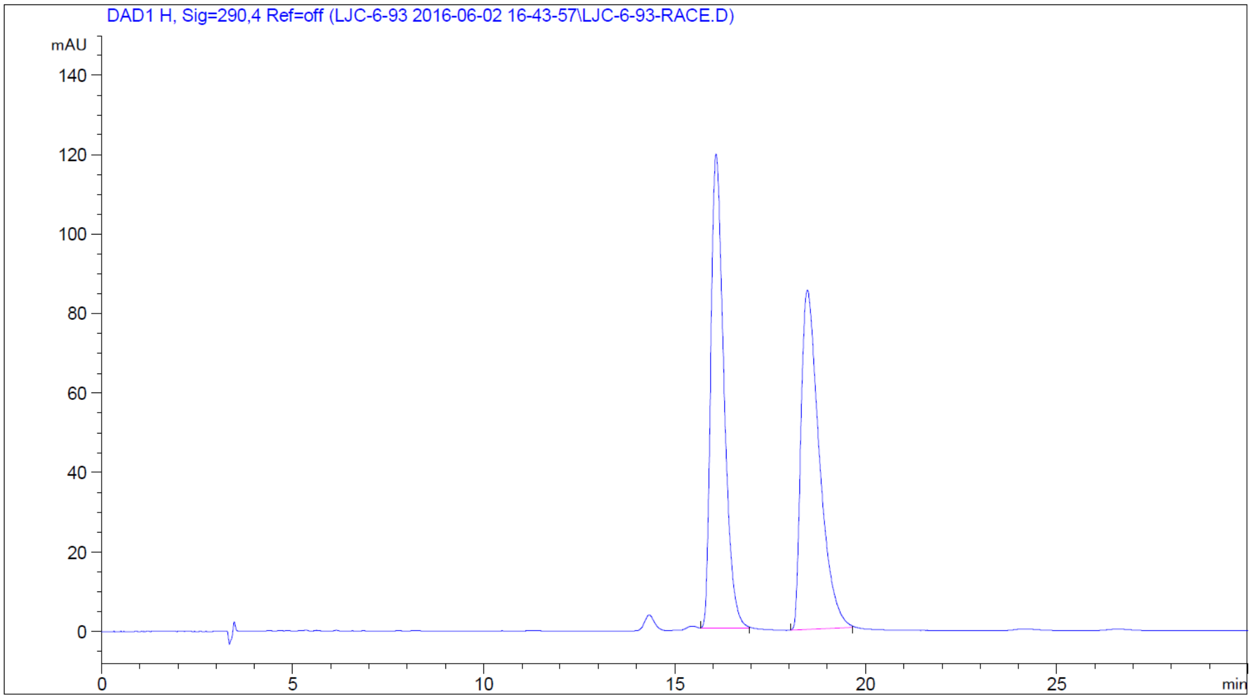

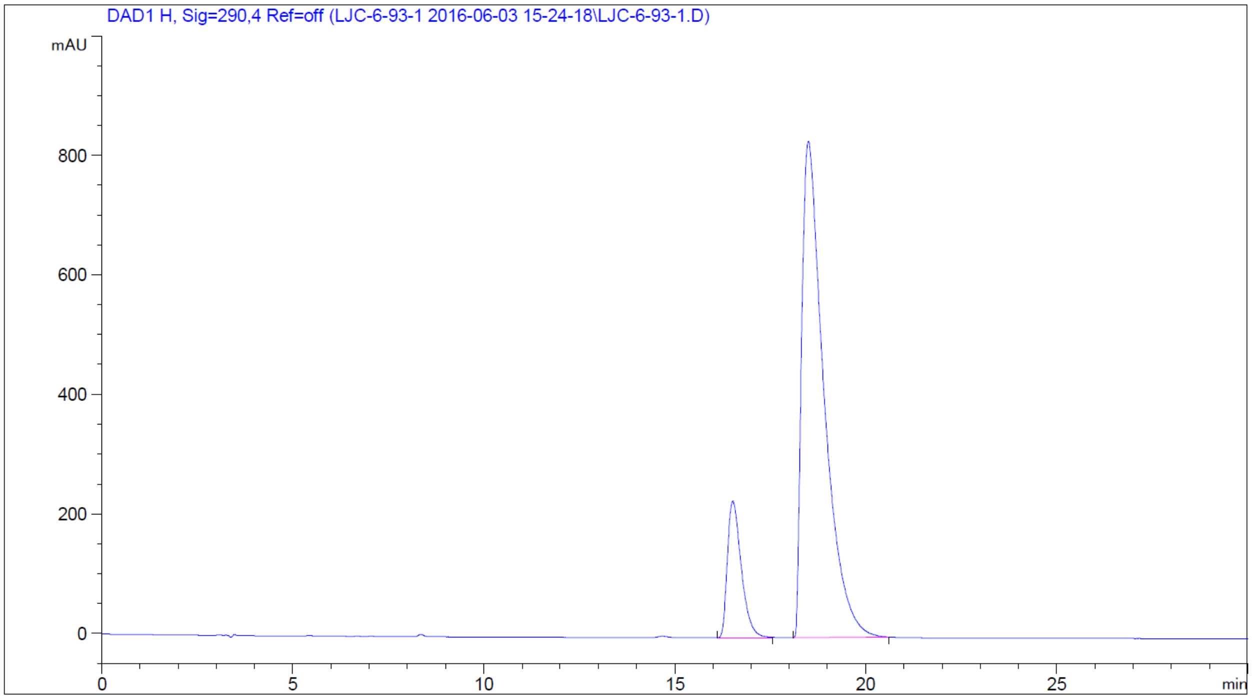

Peak #	RetTime [min]	Type	Width [min]	Area [mAU*s]	Height [mAU]	Area %
1	16.520	BB	0.3894	5789.56738	228.66458	14.6815
2	18.500	BB	0.6066	3.36449e4	830.50861	85.3185

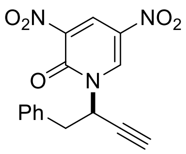

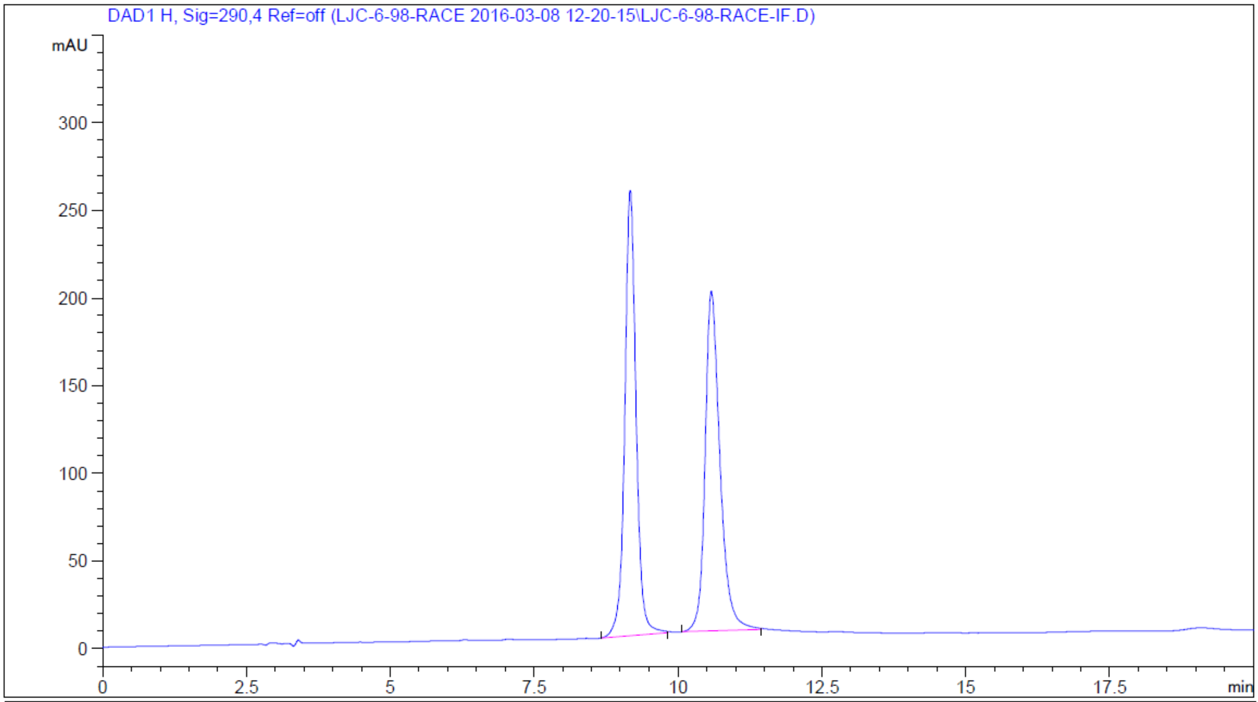

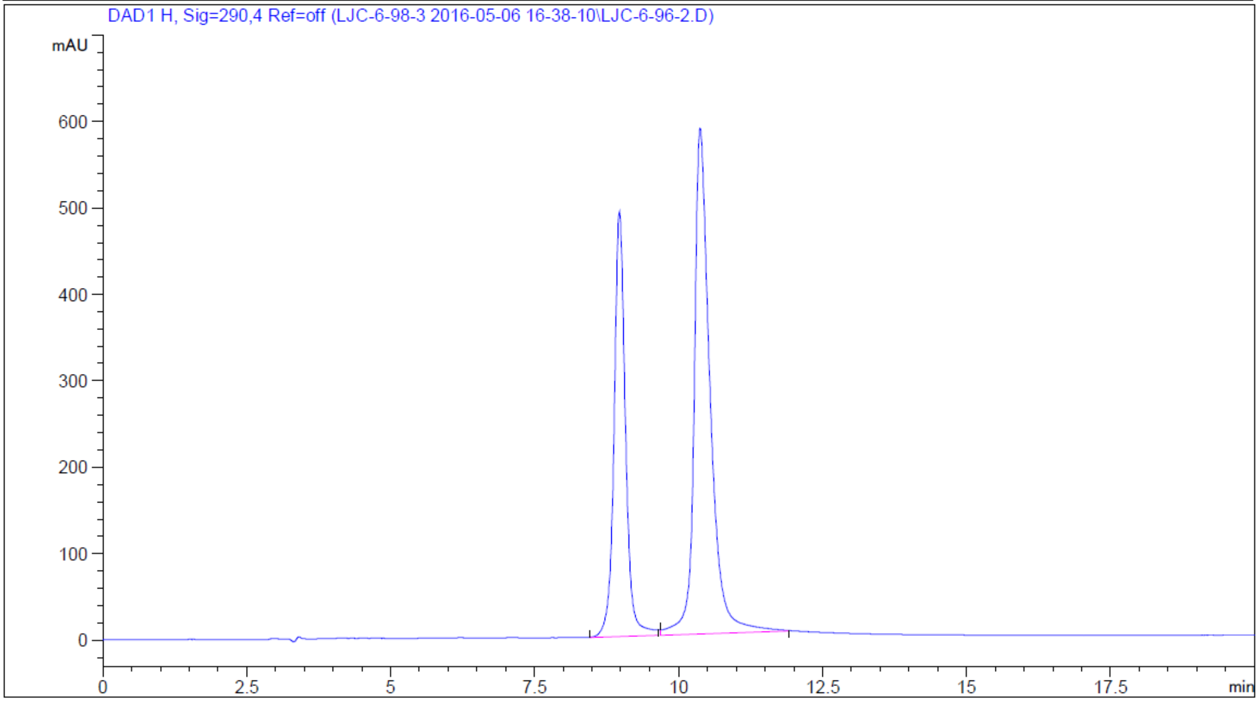

Peak #	RetTime [min]	Type	Width [min]	Area [mAU*s]	Height [mAU]	Area %
1	8.976	BB	0.2067	6779.25098	492.06491	37.5089
2	10.377	BB	0.2812	1.12944e4	585.02814	62.4911

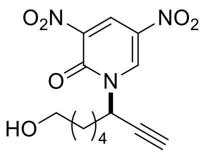

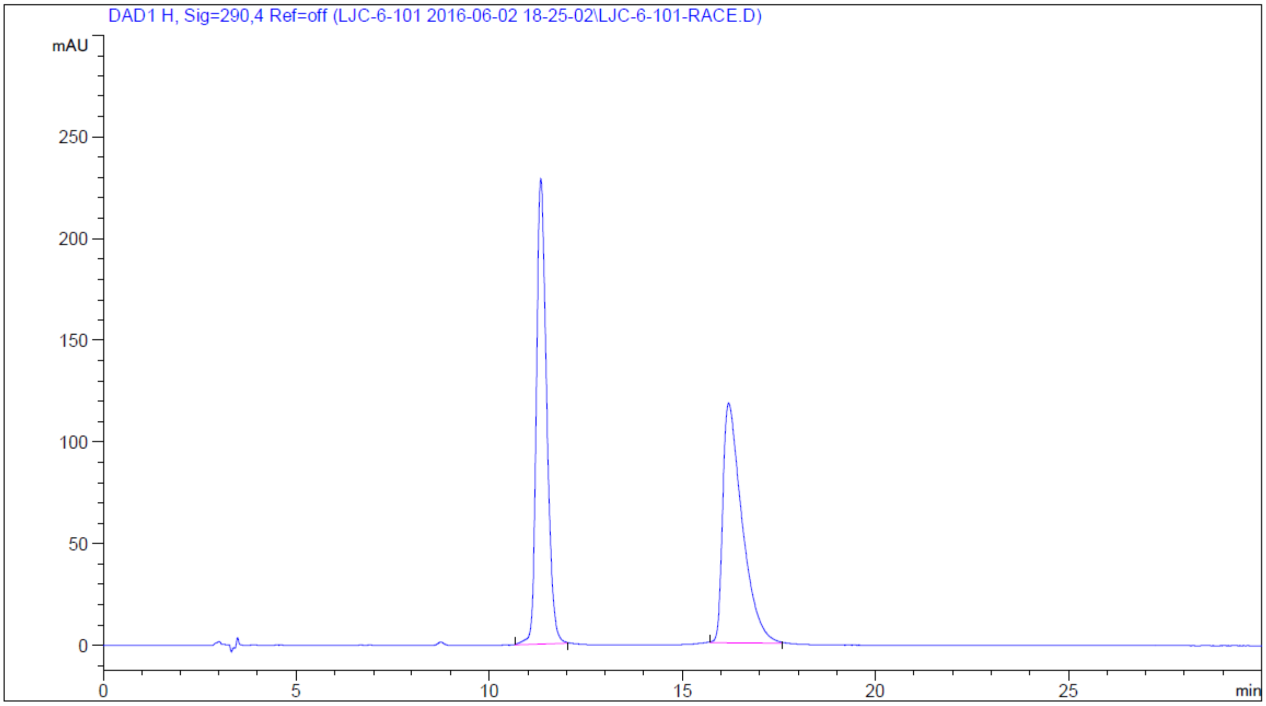

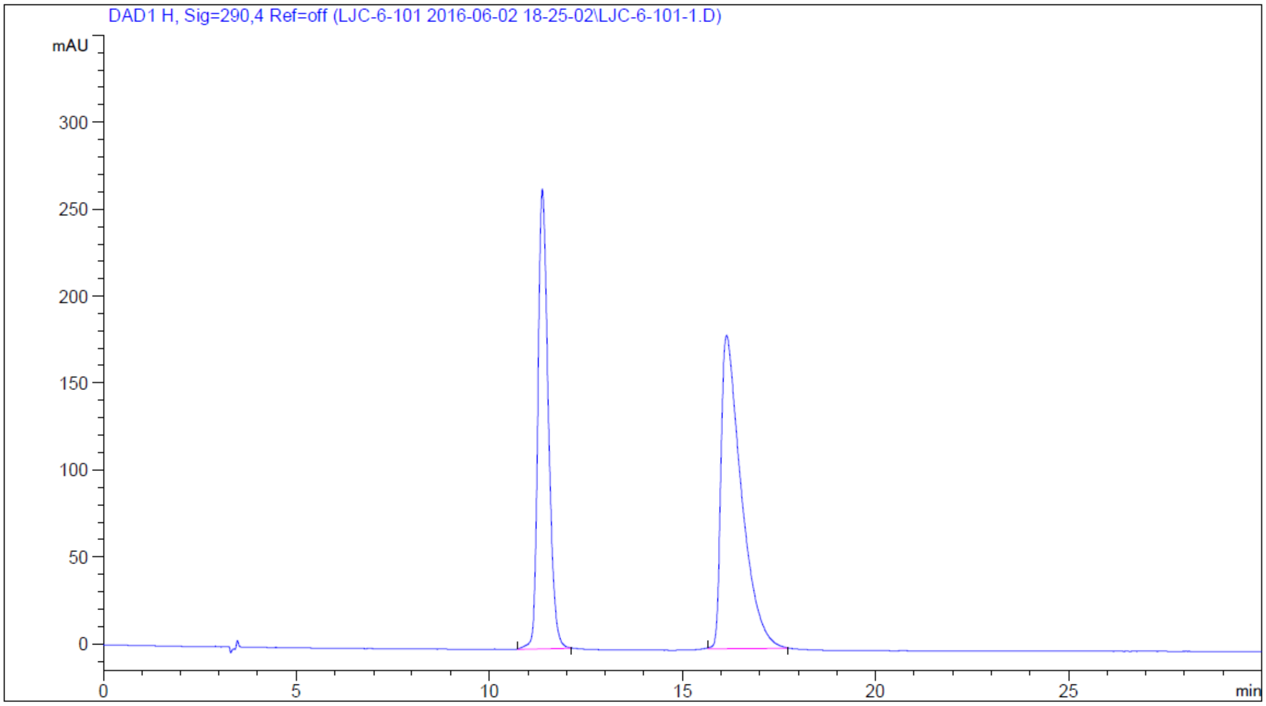

Peak #	RetTime [min]	Type	Width [min]	Area [mAU*s]	Height [mAU]	Area %
1	11.376	BB	0.2851	4929.37305	264.88956	42.7894
2	16.149	BB	0.5370	6590.69580	180.06691	57.2106

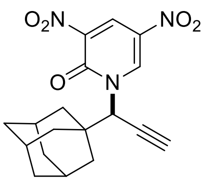

O2N
NO2
O
N

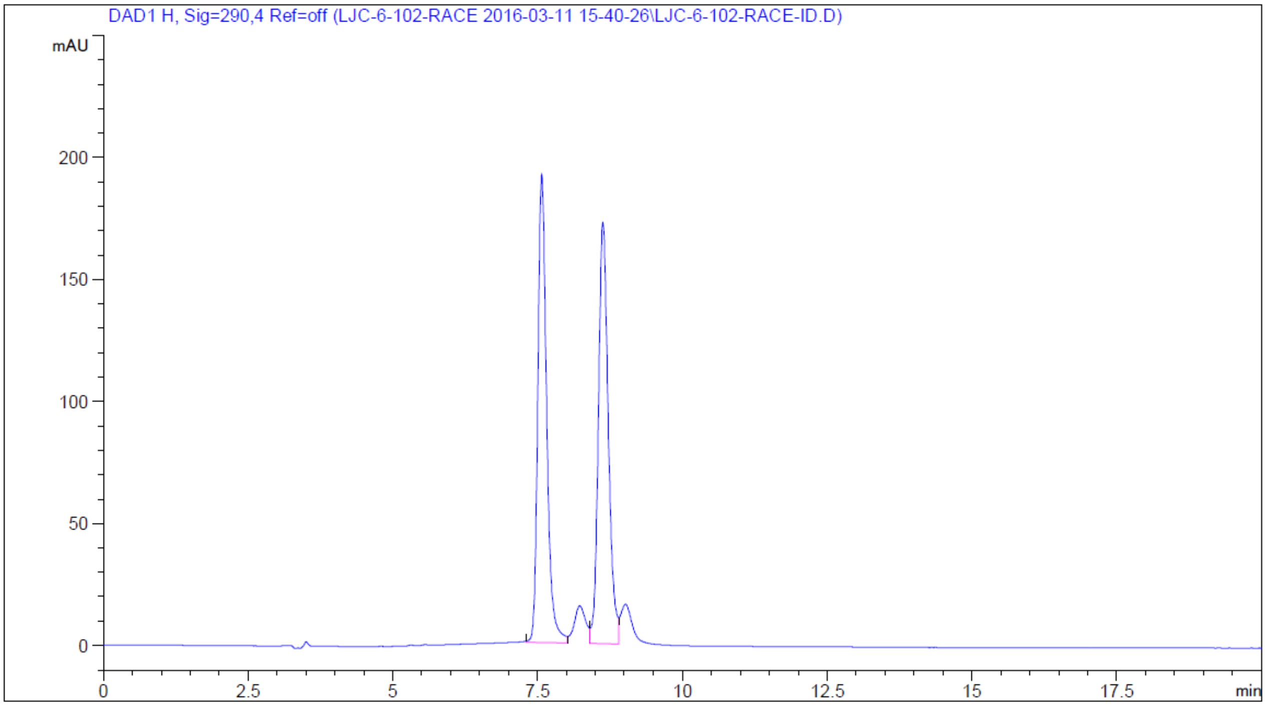

DAD1 H, Sig=290,4 Ref=off (LJC-6-102-RACE 2016-03-11 15-40-26\LJC-6-102-RACE-ID.D)
mAU
200
150
100
50
0
0
2.5
5
7.5
10
12.5
15
17.5
min

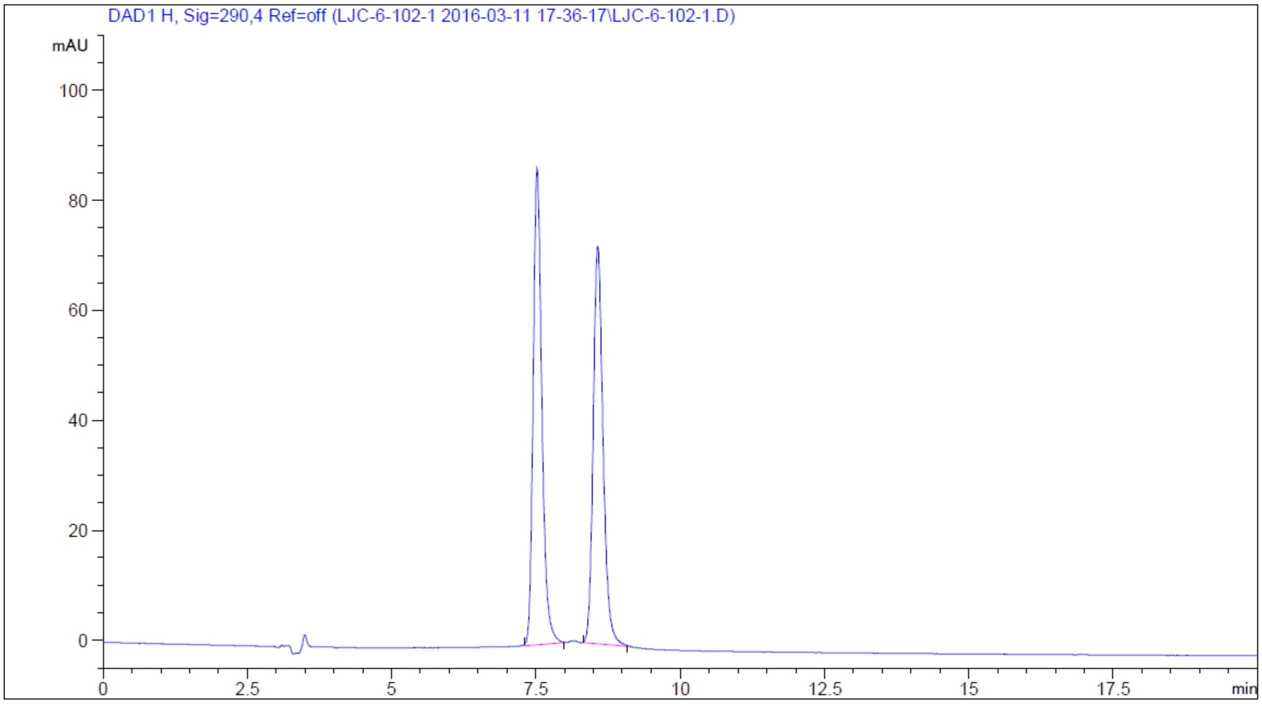

DAD1 H, Sig=290,4 Ref=off (LJC-6-102-1 2016-03-11 17-36-17\LJC-6-102-1.D)
mAU
100
80
60
40
20
0
0
2.5
5
7.5
10
12.5
15
17.5
min

Peak #	RetTime [min]	Type	Width [min]	Area [mAU*s]	Height [mAU]	Area %
1	7.524	BB	0.1599	905.54102	86.66437	51.3623
2	8.575	BB	0.1806	857.50452	72.20493	48.6377

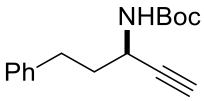

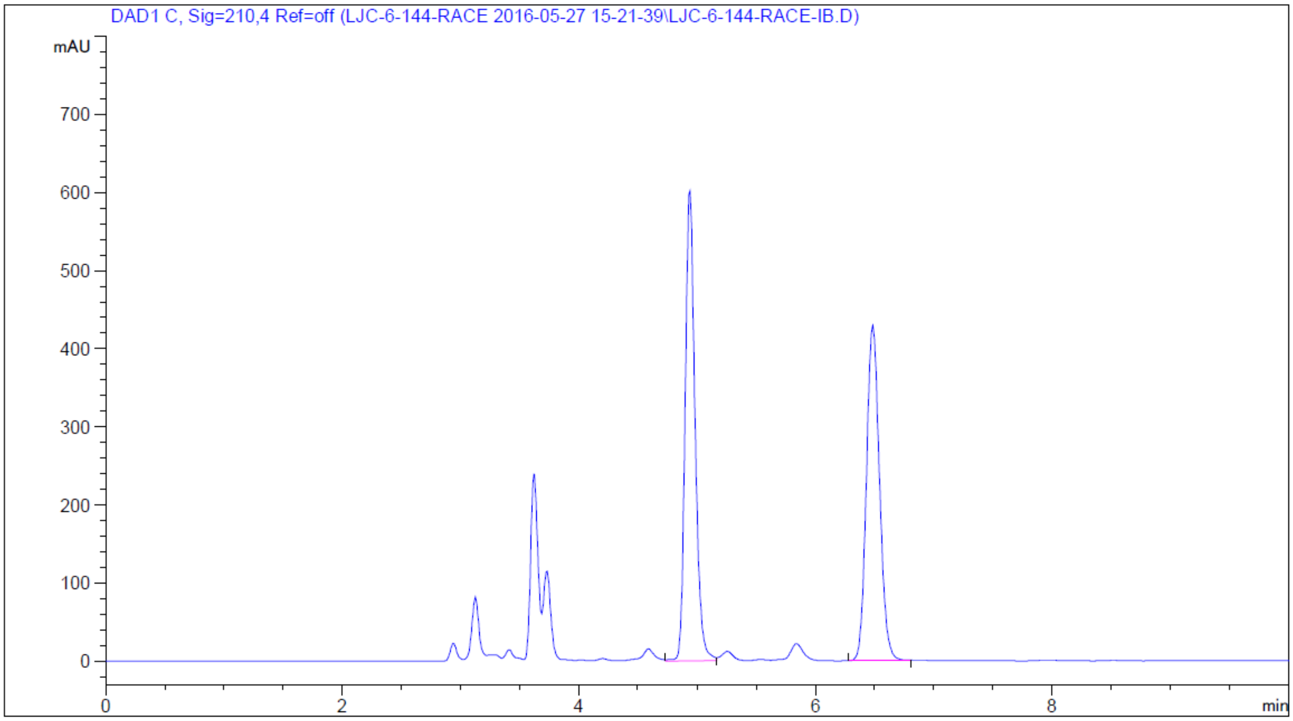

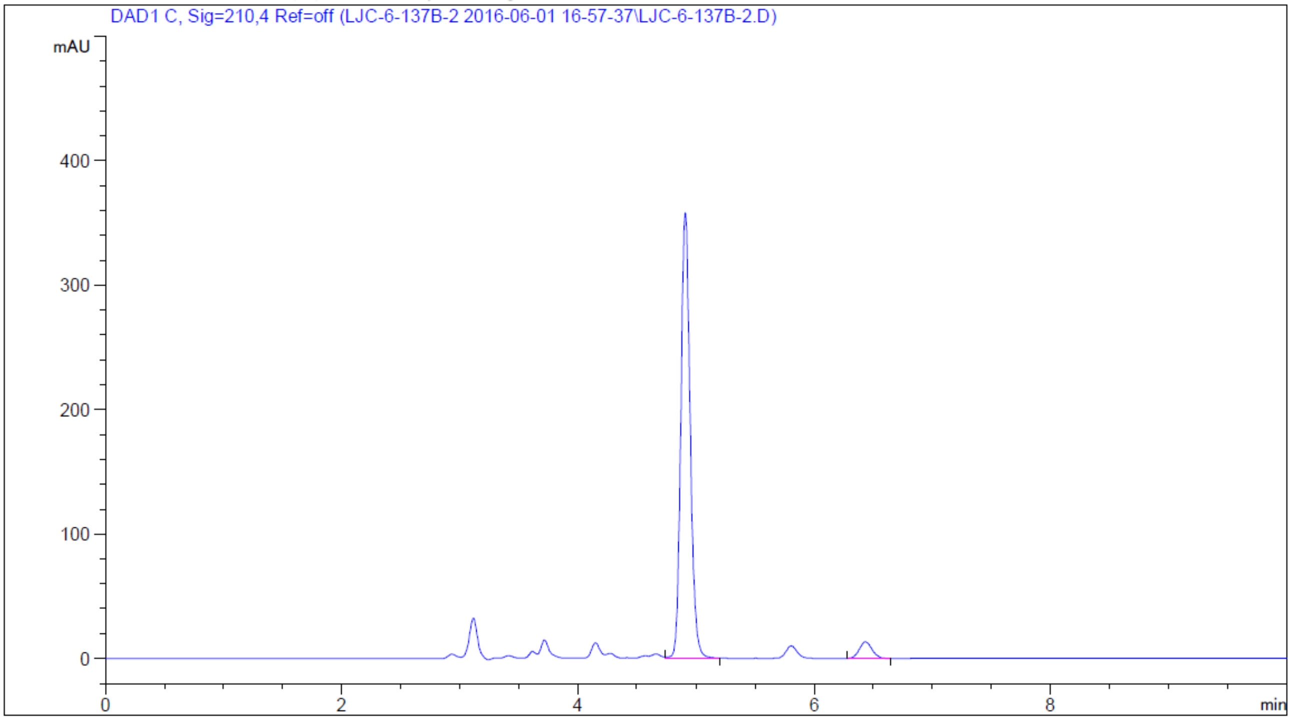

Peak #	RetTime [min]	Type	Width [min]	Area [mAU*s]	Height [mAU]	Area %
1	4.909	VB	0.0841	1975.58911	359.31079	95.0899
2	6.435	BB	0.1159	102.01166	13.54233	4.9101
